# Synthesis and Application of Two-Photon Active Fluorescent Rhodol Dyes for Antibody Conjugation and *In Vitro* Cell Imaging

**DOI:** 10.1021/acsomega.3c01796

**Published:** 2023-06-14

**Authors:** Dénes Szepesi Kovács, Balázs Chiovini, Dalma Müller, Estilla Zsófia Tóth, Anna Fülöp, Péter Ábrányi-Balogh, Lucia Wittner, György Várady, Ödön Farkas, Gábor Turczel, Gergely Katona, Balázs Győrffy, György Miklós Keserű, Zoltán Mucsi, Balázs J. Rózsa, Ervin Kovács

**Affiliations:** † Medicinal Chemistry Research Group, Research Centre for Natural Sciences, H-1117 Budapest, Hungary; ‡ Department of Organic Chemistry and Technology, Budapest University of Technology and Economics, H-1111 Budapest, Hungary; § National Laboratory for Drug Research and Development, H-1117 Budapest, Hungary; ∥ Faculty of Information Technology and Bionics, Pázmány Péter Catholic University, H-1444 Budapest, Hungary; ⊥ Oncology Biomarker Research Group, Research Centre for Natural Sciences, H-1117 Budapest, Hungary; # Department of Bioinformatics, Semmelweis University, H-1094 Budapest, Hungary; ∇ Semmelweis University Doctoral School, H-1085 Budapest Hungary; ○ Integrative Neuroscience Research Group, Research Centre for Natural Sciences, H-1117 Budapest, Hungary; ◆ Femtonics Ltd., H-1094 Budapest, Hungary; ¶ Molecular Cell Biology Research Group, Research Centre for Natural Sciences, H-1117 Budapest, Hungary; & Department of Organic Chemistry, Eötvös Loránd University, H-1117 Budapest, Hungary; ● NMR Research Laboratory, Research Centre for Natural Sciences, H-1117 Budapest, Hungary; ◊ Department of Pediatrics, Semmelweis University, H-1094 Budapest, Hungary; ▲ Brain Vision Center, H-1094 Budapest, Hungary; □ Faculty of Materials and Chemical Sciences, University of Miskolc, Miskolc H-3515, Hungary; ^ Laboratory of 3D Functional Network and Dendritic Imaging, Institute of Experimental Medicine, H-1083 Budapest, Hungary; ¢ Polymer Chemistry and Physics Research Group, Research Centre for Natural Sciences, H-1117 Budapest, Hungary

## Abstract

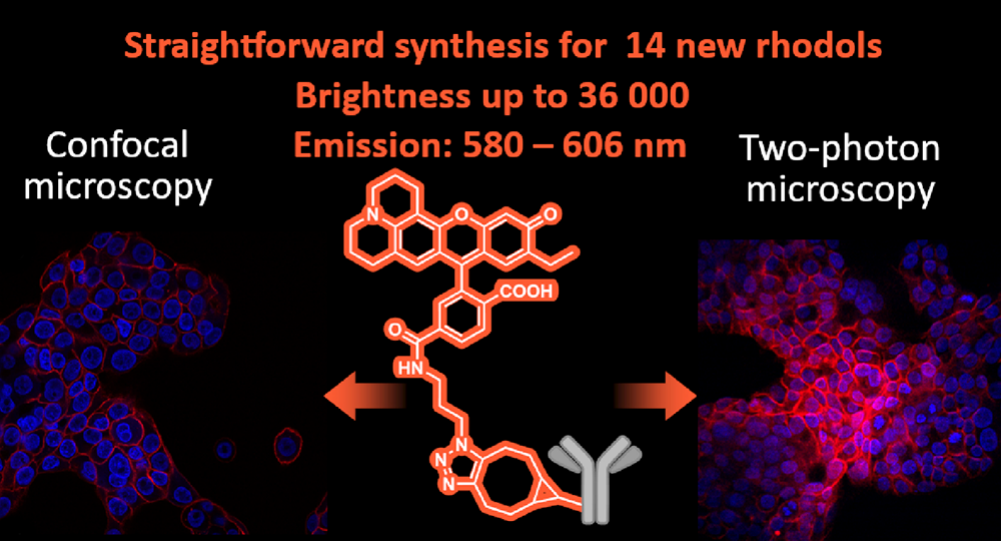

A novel family of julolidine-containing fluorescent rhodols equipped with a wide variety of substituents was synthesized in a versatile two-step process. The prepared compounds were fully characterized and exhibited excellent fluorescence properties for microscopy imaging. The best candidate was conjugated to the therapeutic antibody trastuzumab through a copper-free strain-promoted azide-alkyne click reaction. The rhodol-labeled antibody was successfully applied for *in vitro* confocal and two-photon microscopy imaging of Her2+ cells.

## Introduction

Rapid and reliable identification of malignant tumor cells is crucial in cancer histological diagnosis. To achieve this goal, one of the most efficient and straightforward techniques is the use of specific antibodies that label exclusively the targeted tumor cells with high selectivity. This labeling usually visualizes the tumor cells for an imaging technique and distinguishes them from the healthy population; therefore, *in vitro* or *in vivo* methods based on antibody conjugation are a focus of research interest.^1,2^ Due to these reasons, there is a continuous demand for novel fluorescent dyes exhibiting specific emission maxima, higher photostability, better quantum yield, and significantly larger Stokes shift.^3^

**Figure 1:**
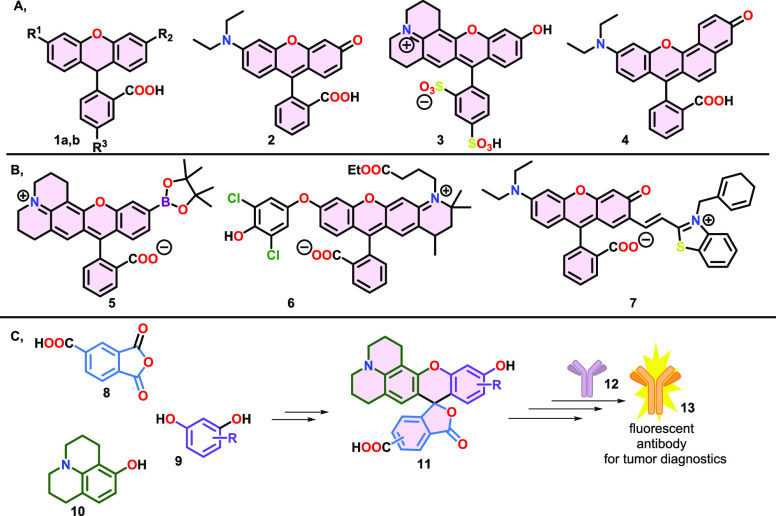
(A) Selected examples for previously published fluorophores (**1a**: TAMRA, R^1^ = NMe_2_, R^2^ = NMe_2_, R^3^ = COOH; **1b**: fluorescein, R^1^ = OH, R^2^ = O, R^3^ = H). (B) Rhodol chemodosimeters used in *in vitro* studies. (C) Synthetic route and proposed application of the dyes synthesized in this work.

Rhodamines, like tetramethylrhodamine (TAMRA, **1a**) and fluorescein (**1b**), are widely used fluorophores (laser dyes, fluorescent probes, and chemosensors) because of their excellent photostability and photophysical properties (Figure 1A).^4,5^ The family of rhodols (**2**) is less prevailing and structurally similar based on the same xanthene scaffold. Rhodols usually have small Stokes shift (ca. 20–25 nm), which need to be increased for better detection. First-generation rhodols (**2**) were synthetized containing a diethylamino group on one side of the xanthene core (Figure 1A). However, there was still some room for improvement in fluorescence properties, and therefore, on the one hand, an electron-donating julolidine core^6−10^ was incorporated into next-generation compounds (**3**) that inhibits the internal rotation of the amino group, decreasing the twisted intramolecular charge transfer (TICT). On the other hand, in some cases, the π-system was extended (**4**) to fine-tune the emission.^11−14^ Furthermore, rhodols were also used as fluorescent probes for inorganic (H_2_O_2_ and HOCl) (**5** and **6**) or glutathione-detecting probes (**7**). The latter compound (**7**) was also applied in biological systems (Figure 1B).^15−17^

Specific fluorescently labeled proteins became effective tools to study the mechanism of complex biological systems.^18,19^ In tumor diagnostics, dye-labeled antibodies have great importance due to their combined unique selectivity and detectability.^1,2,5,20,21^ Nowadays, biorthogonal and click chemistry (*e.g*., azide-alkyne cycloaddition) offers a site-selective and simple labeling method; therefore, most of the fluorescent dyes are equipped with a functional group available for these reactions.^22−25^

Although the access to fluorescence or confocal microscopy is more general than that for two-photon fluorescence microscopy (2P microscopy), using 2P active fluorophores in imaging processes has many advantages. 2P excitation fluorescence imaging that provides thin optical sectioning has enabled a more precise quantification during analysis by restricting out-of-focus excitation (and thus emission). As out-of-focus fluorescence is never generated, no pinhole is required in the microscope detection path, resulting in an increase in the efficiency of fluorescence collection that makes a huge advantage and motivation to develop fluorescent dyes with 2P cross section. Moreover, excitation can be reached at longer wavelengths, the auto-fluorescence is negligible, and due to the lower energy of photons, less photobleaching is experienced.^26,27^ Finally, it is important to note that cancer cell behavior can be examined *in vivo* together with the surrounding tissue, *e.g*., non-tumorous, but tumor-associated stromal cells.^28^ Combining all of the advantages of 2P imaging, this technique is more and more widespread in cell imaging and the number of 2P studies in the diagnostic field is increasing, justifying the development of 2P active and specific fluorescent dyes.^29−31^

In light of these aspects, as a continuation of our interest in novel fluorescent dyes,^32−36^ two-photon active compounds,^35,37^ and antibody modification,^25^ we aimed to develop new rhodol derivatives with increased 2P cross section, large Stokes shift, and high photostability. After investigating the photophysical properties of the library, the best candidate of this set of dyes was cross-linked to the therapeutic antibody trastuzumab, and the dye-labeled antibody was investigated *in vitro* (Figure 1C) for the specific recognition of Her2+ cancer cells by confocal and 2P microscopy.

## Results and Discussion

### Synthesis of a Library of New Rhodol Fluorophores

**Scheme 1:**
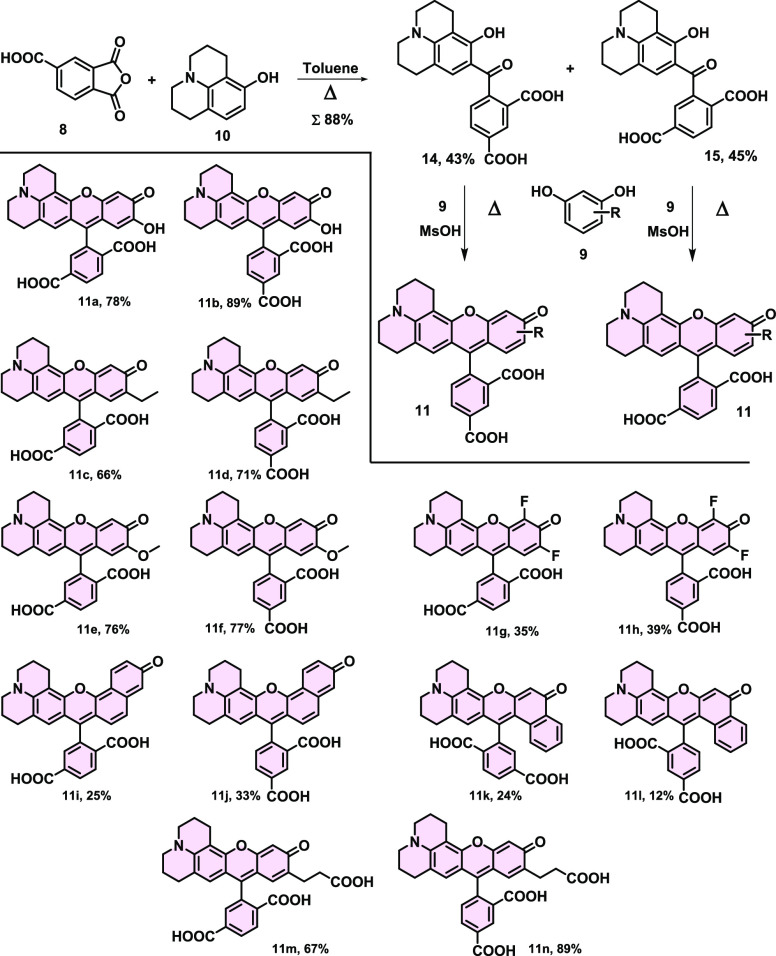
Two-Step Synthesis of Rhodol Derivatives (**11**) from Phthalic Anhydride Derivative (**8**), 8-Hydroxyjulolidine (**10**), and Various Phenol Derivatives (**9**) and the Structure of the Synthetized Compounds

The julolidine-decorated rhodols were synthesized in a two-step synthetic route. At first, the Friedel Craft acylation of 8-hydroxyjulolidine (**10**) using 1,2,4-benzenetricarboxylic anhydride (**8**) resulted in the mixture of ketones **14** and **15**, which were separated successfully by preparative HPLC. Then, both intermediates **14** and **15** were transformed to the desired products **11a**–**11n** using various resorcinol derivatives (**9**) in an acid-catalyzed condensation with up to 89% yields (Scheme 1).

### Photophysical Development of the New Fluorophores

**Scheme 2:**
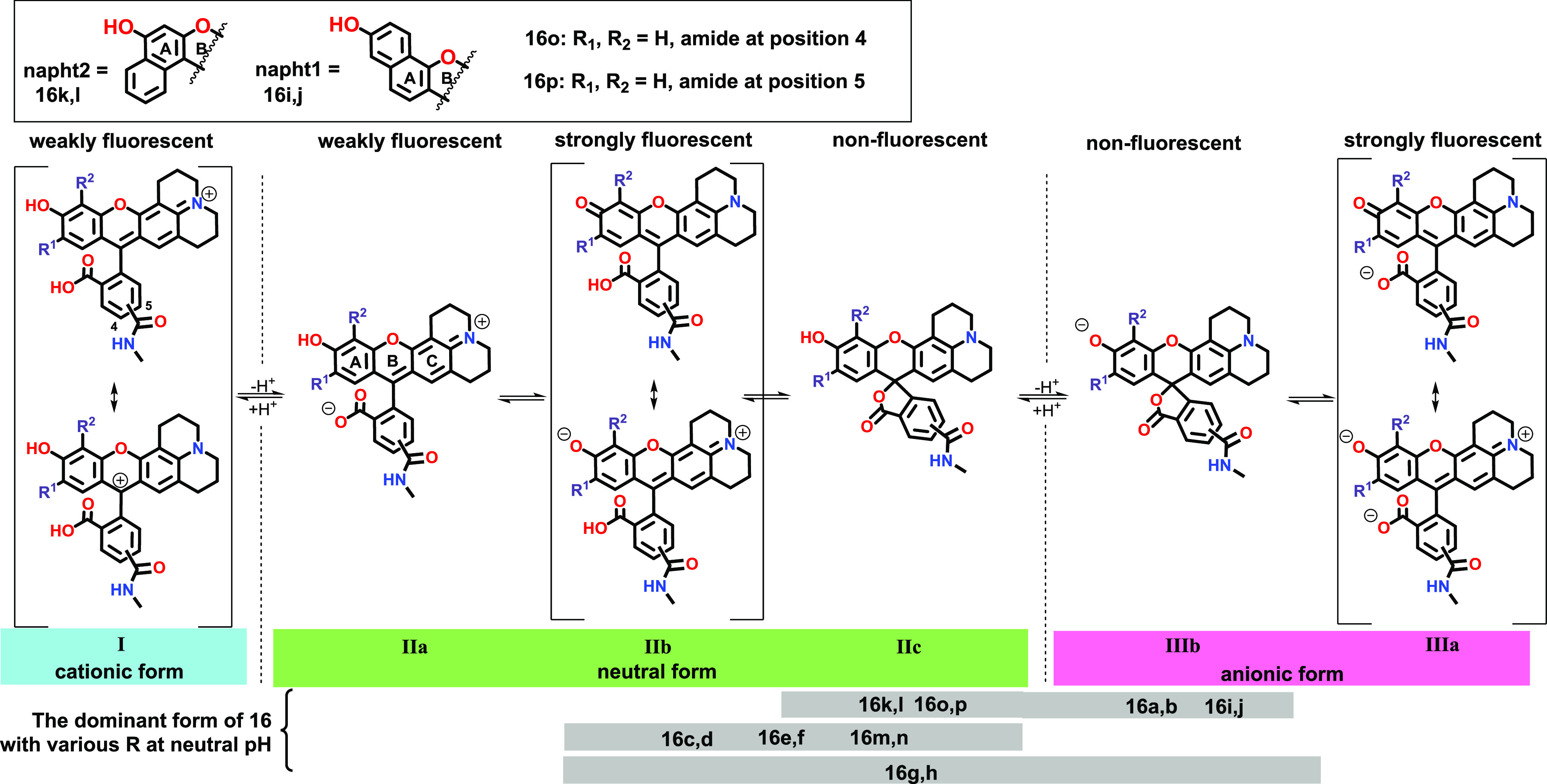
Protonation and Ring Closure Equilibria of Compounds **16a**–**16n** for Computational Study Computed at the B3LYP/6-31G(d,p)//PCM(water) level of theory. Below is the most possible distribution of the rhodol derivatives.

The spectroscopical properties of the rhodol carboxylic acids (**11a**–11**n**) have also been investigated in detail. Molar absorption coefficient, quantum yield, and brightness were determined (Table 1 and Table S1). We have also measured the 2P cross section and the emission intensity (Table 1). In most of the cases (**11a**–**11h** and **11k**–**11n**), the excitation maxima (λ_exc_^max^) are located in the range of visible green light (542–551 nm) between the emission range of the common fluorescein and rhodamine dyes. It could be concluded that small changes in the structure did influence neither the excitation nor the emission significantly (571–606 nm). As expected, **11i** and **11j** with an extended aromatic skeleton showed higher absorbance maxima (λ_abs_^max^ = 570 nm, Table S1). The emission maxima of **11i** and **11j** were detected in the range of yellow and orange light (603–606 nm), close to that of the rhodamine derivatives. The Stokes shifts for the members of the rhodol library are typically 30–41 nm, which is significantly larger than for that of the widely used rhodamines (the Stokes shift of **1a** is only 23 nm; Table 1, entry 15). These advantages could be exploited in imaging experiments as the overlap of the absorption and emission spectra is negligible, allowing more comfortable selection of optical filters. Moreover, the recorded brightness is higher for **11a**, **11d**, and **11m** (Table 1, entries 1, 4, and 13) than the brightness of the frequently used 5-TAMRA (**1a**, 31,554 M^–1^ cm^–1^ in Table 1, entry 15). In summary, the photophysical properties predict good applicability of these new dyes. In some cases (**11c**, **11e**, **11h**, **11i**, **11j**, and **11l** in Table 1, entries 3, 5, 8–10, and 12), molar extinction coefficients and quantum yields were lower, decreasing the brightness of the compound. This could be explained by the well-known ring-chain tautomerism between the lactone and the free acid form of the rhodol along with the changes of pH^38^ and tuned by the substituents (Scheme 2). To explore the ring opening and closing equilibrium, we have investigated the pH dependence of the absorbance and fluorescence of **11d**. The decreasing pH caused a significant drop in the fluorescence intensity; particularly, below pH = 6.4, the intensity decreased to 5% at 577 nm (Figure S49). This is in concordance with the presumption that under acidic conditions, the lactone form is more stable,^39^ while the anionic carboxylate supporting the electron gradient increasing the corresponding wavelengths is present under alkaline conditions. In the case of the excitation maxima using 2P techniques, the 2P properties are similar for all the members of the library. In particular, the excitation maxima could be achieved in the range of 830–850 nm; however, the intensity was generally in the range of 10–150 a.u. (Table 1). The **11d** derivative was selected for further development based on its photospectroscopic properties.

**Table 1:** Single-Photon Characterization of the Novel Rhodols (**11a**–**11n**) and the Extensively Used 5-TAMRA in HEPES (10 μM, pH = 7.4)

		single-photon properties					two-photon properties	
#	compound	λ_exc_^max^ [nm]	λ_em_^max^ [nm]	Δλ [nm]	*B* [M^–1^ cm^–1^]		λ_exc_^max^ [nm]^a^	intensity [a.u.]^+^
1	**11a**	546	585	37	32,678		845	46.0
2	**11b**	547	589	40	19,021		845	96.4
3	**11c**	542	571	28	18,834		845	128.5
4	**11d**	542	577	34	35,663		845	151.7
5	**11e**	547	582	34	18,712		845	59.4
6	**11f**	547	586	38	28,912		845	51.4
7	**11g**	551	580	27	19,153		855	69.0
8	**11h**	550	584	32	7955		845	2.0
9	**11i**	568	606	36	526		855	4.8
10	**11j**	568	603	33	673		855	7.0
11	**11k**	551	592	40	22,142		845	23.2
12	**11l**	551	594	41	18,574		845	28.8
13	**11m**	540	573	30	36,473		835	31.0
14	**11n**	543	579	35	31,055		845	11.8
15	5-TAMRA	551	574	24	31,554		855	104

a λ_exc_^max^: wavelength of the maximum of excitation spectrum. λ_em_^max^: wavelength of the maximum of emission spectrum. Δλ: Stokes shift calculated by λ_em_^max^ – λ_exc_^max^. *B* is brightness (*B* = ε·Φ). For more details, see Table S1 and experimental section in the Supporting Information.

### Theoretical Calculations to Explain the Differences in Fluorescence Properties

In the course of the computational study, the fluorophore was modeled as the R,^1^R^2^-functionalized methyl amide derivative (**16a**–**16n**, corresponding to **11a**–**11n**, and in addition, the non-substituted derivatives as R,^1^R^2^ = H in **16o** and **16p** (see Scheme 2 and Table S3a), mimicking the antibody–fluorophore conjugate (**13**). Both 4- and 5-carboxamide isomers (series derived from **14** and **15**, respectively) were considered. The rhodol scaffold, bearing a positive charge, participates in complex deprotonation and isomerization equilibria (Scheme 2). The various species (forms I, II, and III) exhibit different fluorescence properties, and their appearance in the solution depends on their thermodynamic stability, indicated by the computed Δ*G* values (Table S2). Here, we studied only the substituent effect on this equilibrium to estimate the relationship between the distribution of the tautomeric forms and fluorescence intensities. Form IIb is responsible for the high fluorescence intensity with overall neutral charge, so in our scope, we looked for a derivative, where form IIb has the lowest Δ*G* value, suggesting that it is the most preferred form.

Interestingly, the position of the carboxamide functional group on the phenyl ring has no significant effect on the computed result (ΔΔ*G* is less than 2 kJ mol^–1^), so we focused on the functional groups decorating ring A of the xanthene. Considering a protonation equilibrium with water molecules (Figure S55), in general, at neutral pH, form IIb is more stable than form I (ranges from +5 to +42 kJ mol^–1^) and, except **16k**–**16n**, form IIb is more stable than form IIIb (ranges from +4 to +20 kJ mol^–1^, Table S2). For R^1^ = R^2^ = F (**16g** and **16h**), form IIb and form IIIb are quite close to each other (ΔΔ*H* less than 4 kJ mol^–1^). In the cases of **16k** and **16l** (R = napht2) and **16m** and **16n**, (R = −C_2_H_4_COOH), form IIIb is the most stable. Since we are looking for neutral structures, these compounds are not suggested for conjugation based on theoretical assumptions.

In the next section, we compare only the different form II species. Among the substituents, the OH functional group (**16a** and **16b**) prefers mostly form IIb by ca. 40 kJ mol^–1^, which is due to the strong and stabilizing hydrogen bond between O^–^ and the neighboring OH group. This also refers to the increased acidity of the rhodol OH. Compounds **16i** and **16j** (R = napht1) favor the ring closed forms IIc and IIIb, which are not fluorescent in accordance with the experimental findings, so these are also excluded from the selection. The ethyl (**16c** and **16d**), carboxyethyl (**16m** and **16n**), and methoxy (**16e** and **16f**) substituents, however, exhibit favorable distribution for the fluorescent form IIb. In conclusion, both theory and experiments suggest that the last six derivatives can be promising candidates for antibody conjugation.

Finally, to demonstrate the ability of the selected rhodol **11d** for biological application, we have investigated the photostability in HEPES buffer by exciting continuously using a 520 nm LED light source (5 and 10 W). The original fluorescence intensity decreased to 50% after 15 min in the case of 5 W and after 10 min in the case of 10 W irradiation (Figure S50). The rates of bleaching were acceptable, considering an imaging process that usually requires sequential excitation for just a couple of minutes and lower than the commonly used fluorescein. Furthermore, investigating the solvent effect on the photophysical properties of **11d** (Figure S51) in a series of solvents, we have observed decreased absorbance and fluorescence intensity for apolar-aprotic solvents (toluene, dioxane, and tetrahydrofuran). In the case of slightly polar and protic ethanol, we detected a significant increase in the absorbance and two times higher fluorescence intensity compared to HEPES buffer. Also, there is a slight hypsochromic effect in EtOH compared to buffer, DCM, and acetonitrile. Considering the emission wavelength, the aqueous buffer seems to be an appropriate medium, confirming the usability in biological investigations. In aqueous medium, suppressed aggregation quenching is often relevant;^3,26,40^ however, in the case of **11d**, there is no significant loss of intensity with and without SDS in HEPES buffer (Figure S52). Furthermore, chemical stability tests over 24 h in aqueous solutions using various buffers (pH 3 to 11; Figure S53) also proved the excellent applicability of the selected candidate **11d**.

### Antibody Conjugation and Microscopy Imaging

**Scheme 3:**
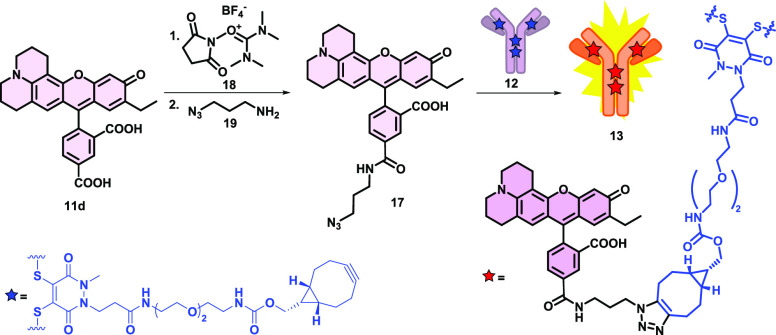
Transformation of **11d** Acid with a Simple and Fast Method to NHS active ester Followed by the Introduction of the Azide Functional Group and the Use of **17** in Click Reaction to Produce the Antibody–Fluorophore Conjugate **13**

After having these promising results in hand, we transformed **11d** to **17** azide, enabling azide-alkyne click reaction. Knowing that antibodies target specific and unique cancer cells and deliver their fluorescent^1^ or cytotoxic payloads^41^ with high accuracy, we have conjugated **17** to the human IgG trastuzumab having four cyclooctynyl harbors (created after literature procedures, shown in Scheme 3),^42^ resulting in a potential diagnostical tracer **13** for imaging of Her2+ cells.^43^

At first, an activated^44^ NHS ester of **11d** was prepared with TSTU (**18**), followed by the smooth acylation of 3-azidopropane-1-amine (**19**), resulting in **17**. Then, we were able to label the antibody (**12**) with the fluorescent dye in a copper-free strain-promoted azide-alkyne click reaction at room temperature. The antibody–fluorophore conjugate **13** was characterized by UV/Vis spectroscopy to determine the fluorophore–antibody ratio (FAR). With the use of the Lambert–Beer equation, the ideal FAR = 4 value was confirmed (Table S2). The homogeneity of the conjugate was determined by SDS-PAGE that showed high (95%) homogeneity after the modification steps (Figure S53). Investigating the gel under UV light (366 nm), the fluorescent spot of the antibody–fluorophore conjugate could be seen by the naked eye (Figure S54).

**Figure 2:**
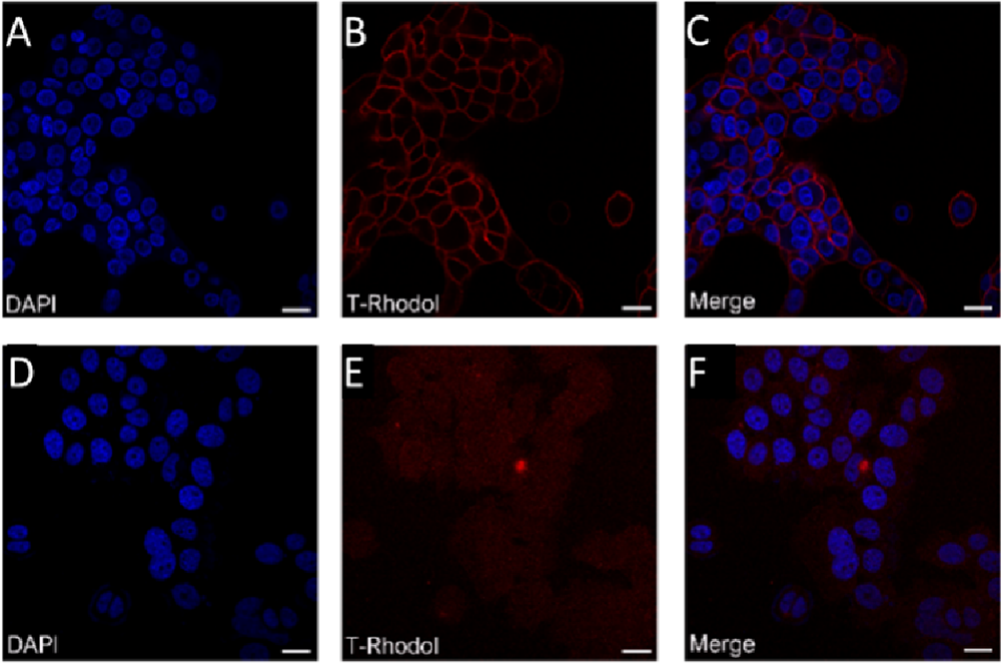
Confocal microscopy images of NCI-N87 (Her2+) (A–C) and MCF-7 (Her2−) (D–F) cells treated with **13** fluorophore–antibody conjugate. (A) DAPI cell nucleus labeling of NCI-N87 cells. (B) **13** conjugate labeling of NCI-N87 cells. (C) Merged image of DAPI and **13** conjugate labeling of NCI-N87 cells. (D) DAPI cell nucleus labeling of MCF-7 cells. (E) **13** conjugate labeling of MCF-7 cells. (F) Merged image of DAPI and **13** conjugate labeling of MCF-7 cells. Scale bars: 10 μm. (A, C, D) Excitation: 405 nm; emission: 456 nm. (B, E, F) Excitation: 543 nm; emission: 687 nm.

Having the antibody–fluorophore conjugate (**13**) in hand, first, we examined the selectivity of the conjugates in flow cytometry using living cells. We treated NCI-N87 cells overexpressing Her2 receptor and MCF7 Her2-negative cells with **13** conjugate and observed the same selectivity as the native antibody^45,46^ and its pyridazinone conjugates,^42^ indicating that the conjugates might be useful tools also on living cells (Figure S55). Second, cell sections of the same cell lines were treated with **13** (Figure 2). Confocal microscopy showed no membrane labeling for Her2-negative MCF-7 cells, while in the case of the Her2+ cell line, the membrane labeling (red, Figure 2B) was significant next to the signal of DAPI (blue, Figure 2A).

**Figure 3:**
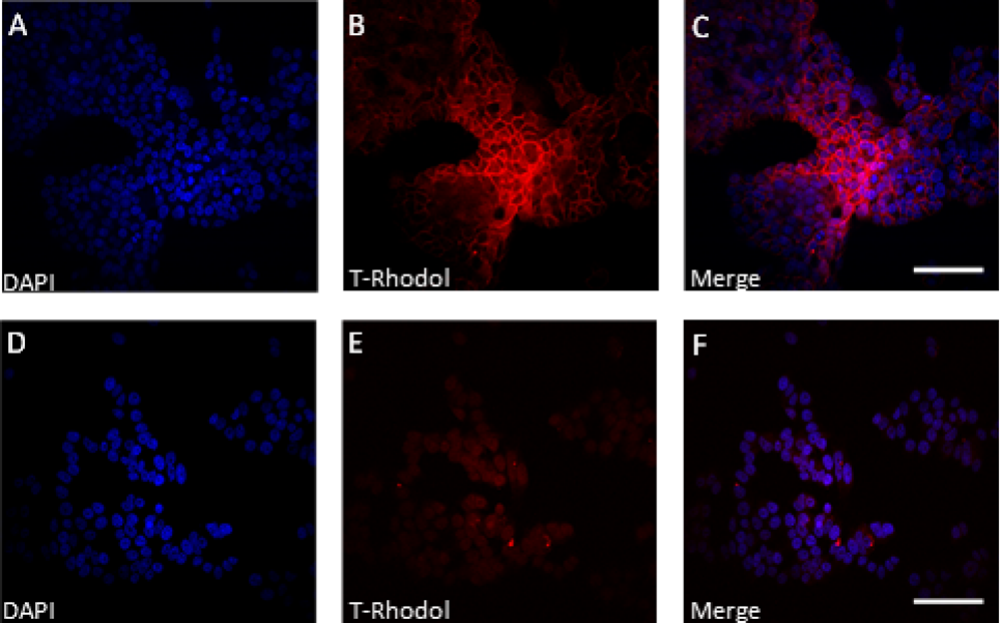
Artificially recolored two-photon images of **13** antibody conjugate-treated Her2+ and Her2– cells. (A) DAPI labeling of NCI-N87 cells excited at 750 nm (PMT filter: 490–550 nm). (B) Fluorescence of **13** antibody conjugate on NCI-N87 cells excited at 840 nm (PMT filter: 570–640 nm). (C) Merged image of NCI-N87 cells. (D) DAPI labeling of MCF-7 cells excited at 750 nm (PMT filter: 490–550 nm). (E) Image of **13** conjugate-treated MCF-7 cells excited at 840 nm (PMT filter: 570–640 nm). (F) Merged image of MCF-7. Scale bars: 100 μm. For camera images, see Figures S56–S58.

We examined the cell section with 2P microscopy as well, observing the analogue labeling pattern (Figure 3). Membrane labeling is clearly visible on the merged picture (Figure 3C) using the red fluorescence signal (Figure 3A) on the surface of NCI-N87 cells (Her2+) and GFP fluorescence images (Figure 3B) of the same (Her2+) cells similar to using confocal techniques (Figure 2C). In the case of MCF-7 (Her2−) cells, only autofluorescence was detected on the same wavelength (Figure 2D–F). Therefore, we confirmed that novel rhodol **11d** is also an effective tool for 2P microscopy imaging and the antibody–fluorophore conjugate retained its selectivity examined on two cell lines.

## Conclusions

In conclusion, we have synthesized a library of next-generation rhodol fluorophores with improved fluorescence properties compared to 5-TAMRA, particularly better Stokes shift, and increased brightness. The new dyes exhibited specific emission wavelength, excellent brightness, applicability in confocal as well as in 2P microscopy, and good photostability for imaging in aqueous buffer. In the case of some derivatives, the decreased fluorescence could be explained by ring-chain tautomerism supported by theoretical calculations. The dye with the best properties was equipped with an azide functional group and linked to cyclooctyne-derived trastuzumab. Finally, this fluorophore–antibody conjugate was proven to be efficient to selectively label Her2-positive NCI-N87 cells in both confocal and 2P microscopy imaging of cells designated as trastuzumab targets.

## Supporting Information Available

Experimental procedures and compound characterization (Figures S1–S55), 2P measurements with camera images (Figures S56–S58), structure and data of computed water clusters (Figure S59), FAR determination (Table S2), and theoretical data (Tables S3 and S4) (PDF)
