# Supplementary material for: Synthesis and Application of Two-Photon Active Fluorescent Rhodol Dyes for Antibody Conjugation and In Vitro Cell Imaging
Source: ACS Omega. 2023 Jun 14;8(25):22836–43. doi: 10.1021/acsomega.3c01796 (PMC10308389; doi:10.1021/acsomega.3c01796)
Supplement: Supplementary file 1 — ao3c01796_si_001.pdf [file ao3c01796_si_001.pdf]

Synthesis and application of two-photon active fluorescent rhodol dyes for antibody conjugation and in vitro cell imaging

Dénes Szepesi Kovács,[a,b,c,‡] Balázs Chiovini,[d] Dalma Müller,[e,f,g] Estilla Zsófia Tóth,[c,g,h] Anna Fülöp,[i] Péter Ábrányi-Balogh,[a,b,c] Lucia Wittner,[c,h] György Várady,[j] Ödön Farkas,[k] Gábor Turczel,[l] Gergely Katona,[d] Balázs Győrffy,[c,e,f,m] György Miklós Keserű,[a,b,c]* Zoltán Mucsi,[n,o]* Balázs J. Rózsa,[d,n,p]* Ervin Kovács[i,q,*,‡]

[a] Medicinal Chemistry Research Group, Research Centre for Natural Sciences, H-1117 Budapest, Hungary;
[b] Department of Organic Chemistry and Technology, Budapest University of Technology and Economics, H-1111 Budapest, Hungary
[c] National Laboratory for Drug Research and Development, H-1117 Budapest, Hungary
[d] Faculty of Information Technology and Bionics, Pázmány Péter Catholic University, H-1444 Budapest, Hungary
[e] Oncology Biomarker Research Group, Research Centre for Natural Sciences, H-1117 Budapest, Hungary
[f] Department of Bioinformatics, Semmelweis University, H-1094, Budapest, Hungary
[g] Semmelweis University Doctoral School, H-1085 Budapest Hungary
[h] Integrative Neuroscience Research Group, Research Centre for Natural Sciences, H-1117 Budapest, Hungary
[i] Femtonics Ltd. H-1094 Budapest, Hungary
[j] Molecular Cell Biology Research Group, Research Centre for Natural Sciences, H-1117 Budapest, Hungary
[k] Department of Organic Chemistry, Eötvös Loránd University, H-1117 Budapest, Hungary
[l] NMR Research Laboratory, Research Centre for Natural Sciences, H-1117 Budapest, Hungary
[m] Department of Pediatrics, Semmelweis University, H-1094, Budapest, Hungary
[n] Brain Vision Center, H-1094 Budapest, Hungary
[o] Faculty of Materials and Chemical Sciences, University of Miskolc, Miskolc H-3515, Hungary
[p] Laboratory of 3D Functional Network and Dendritic Imaging, Institute of Experimental Medicine, H-1083 Budapest, Hungary
[q] Polymer Chemistry and Physics Research Group, Research Centre for Natural Sciences, H-1117 Budapest, Hungary;
‡These authors contributed equally.

Table of contents

General 2
Synthesis of **14**,**15** benzophenones and rhodols **11a-n** and azide **17**. 3
SDS-PAGE and densitometry 4
Photophysical measurements 5
Absorption and emission data of the synthetized novel compounds **11a–n** and 5-TAMRA. 6
  Trastuzumab reconjugation with DBPD-CO 7
  Click reaction of **17** and T-CO conjugate (**12**) resulting fluorescent conjugate (**13**) 7
  Cell lines and culture conditions 7
  Flow cytometry 7
  Immunocytochemistry 8
  In vitro two-photon imaging 8
Chemical characterization of **15, 16**, rhodol derivatives **11a-n** and **14** 10
Structure determination of **11f** rhodol was carried out using 2D NMR measurements........ 37
Two-photon cross section of the relevant rhodol derivatives 40
The pH dependent of absorption and emission spectra of **11d**........ 40
Photostability measurement of **11d** dye........ 40
Solvent dependence of **11d** rhodol derivative 41
Aggregation studies of **11d** in aqueous media 41
pH stability studies of **11d**........ 42
SDS-PAGE results of **13** conjugate 42
Flow cytometry measurements of cells without or treated with **13** antibody conjugate 42
Absorbance measurement of rhodol-trastuzumab conjugate (**13**) 43
Two-photon measurements of cells without or treated with **13** antibody conjugate. 44
Computed enthalpy and Gibbs free energy changes, the coordinates of the optimized structures.46
References 146

General

All the chemical reagents, solvents were purchased from Merck/Sigma Aldrich, ThermoFisher and VWR International and used as received. NMR solvents were purchased from Eurisotop. Trastuzumab antibody was from Genentech Inc.

Reactions were monitored by LC-MS (Shimazu MS2020, Supelco Ascentis, 2.0 × 50 mm, 2.1 µm C18 column; injection of 1 µl; 5–98% MeCN/H_2O, linear gradient, with constant 0.1% v/v TFA additive; run of 6 min; flow of 0.8 ml min^{-1}; ESI; positive ion mode). Reaction products were purified by gradient elution preparative HPLC (HPLC Gilson 333 instrument, UV detector 220 nm) on a Phenomenex Gemini C18, 250×50.00 mm; 10 µm, 110A column using 0.2% v/v TFA in water and acetonitrile as the mobile phase components.

^{1}H NMR and ^{13}C NMR spectra were recorded in DMSO-d_6 solution at room temperature, on a Varian Unity Inova 500 spectrometer (at 500 MHz and 126 MHz for ^{1}H and ^{13}C/APT NMR spectra, respectively), and on a Varian Unity Inova 300 spectrometer (at 300 MHz and 75 MHz for ^{1}H and ^{13}C NMR spectra, respectively). All chemical shifts are quoted in parts per million (ppm), measured from the center of the signal except in the case of multiplets, which are quoted as a range. ^{1}H NMR and ^{13}C chemical shifts are referenced to the residual solvent peak of $(CD_3)_2SO$ (^{1}H referenced to 2.50 ppm and ^{13}C referenced to 39.52 ppm) or to TMS as the internal standard. Coupling constants are given with an accuracy of 0.1 Hz. Splitting patterns are abbreviated as follows: singlet (s), doublet (d), triplet (t), quartet (q), multiplet (m), broad singlet (bs) and combinations thereof. Assignment of spectra was aided by 2D NMR spectroscopy (^{1}H-^{13}C HSQC and HMBC).

HRMS analyses were performed on high-resolution measurements were performed on a Sciex TripleTOF 5600+ high resolution tandem mass spectrometer equipped with DuoSpray ion source. Electrospray ionization was applied in positive ion detection mode. Samples were dissolved in acetonitrile and flow injected into acetonitrile: water 50/50 flow. The flow rate was 0.2 mL/min. The resolution of the mass spectrometer was 35 000.

The two-photon absorption cross sections (TPCSs) were determined with the two-photon excited fluorescence (TPEF) method.[1] The measurements were performed using an inverted two-photon microscope (FemtoSmart2D, Femtonics), equipped with a XLUMPFLN20XW Olympus objective (numerical aperture; NA = 1.0) and a tuneable high-power Ti:Sapphire laser (Coherent Chameleon Discovery COM5, wavelength of the excitation light is between 700 nm and 1040 nm). The incident light source was focused into at capillary filled with either the sample or the reference solution (Rhodamine 6G in MeOH[2]) and integrated fluorescence emission was detected in a wavelength window from 570 to 640 nm (green channel of the

microscope). The power of the laser source was kept constant at 30 mW. TPCS at each excitation wavelength was calculated according to the following equation:

$$TPCS_{\mathrm{s}} = TPCS_{\mathrm{ref}} \cdot \frac{A_{\mathrm{sam}} \cdot c_{\mathrm{ref}} \cdot a_{\mathrm{ref}} \cdot n_{\mathrm{D,ref}}}{A_{\mathrm{ref}} \cdot c_{\mathrm{sam}} \cdot a_{\mathrm{sam}} \cdot n_{\mathrm{D,sam}}}$$

where A is the mean TPEF emission intensity, c is the dye concentration, n_D is the refractive index of the solvent measured at the sodium D-line; a is a variable derived from one-photon emission measurements calculated as the integral of one-photon emission spectrum from 570 to 640 nm divided by the total one-photon emission spectrum integral. Lower indices *ref* and *s* refer to the reference compound and the sample, respectively.

All theoretical calculations computations were carried out with the Gaussian16 program package (G16)[3], using standard convergence criteria for the gradients of the root mean square (RMS) Force, Maximum Force, RMS displacement and maximum displacement vectors (3.0×10^{-4}, 4.5×10^{-4}, 1.2×10^{-3} and 1.8×10^{-3}). Computations were carried out at B3LYP level of theory[4] by the basis set of 6-31G(d,p). The vibrational frequencies were computed at the same levels of theory, in order to confirm properly all structures as residing at minima on their potential energy hypersurfaces (PESs). Thermodynamic functions U, H, G and S were computed at 398.15 K. Beside the vacuum calculations, the IEFPCM method was also applied to model the solvent effect, by using the default settings of G16, setting the $\varepsilon = 78.3553$ for water[5].

Synthesis of **14**,**15** benzophenones and rhodols **11a-n** and azide **17**.

*Synthesis of benzophenone **14** and **15**, the key intermediers of rhodol derivative*[6]

Julolidin-8-ol (1.987 g, 10.50 mmol, 1 equivalent) and 1,2,4-benzenetricarboxylic anhydride (2.017 g, 10.50 mmol, 1 equivalent) were suspended in dry toluene (150 mL) and stirred at 110 °C for 20 hours. Dark precipitate formation was observed during the reaction, the solution became brownish in the end of the reaction. The mixture with precipitate was evaporated under reduced pressure. The yellowish-brown solid residue (4.60 g) was purified by preparative HPLC (water–acetonitrile–0.1% TFA, using the gradient method). After purification, the fractions were lyophilized. The products (**14**, 1.71 g, yield 43%; **15**, 1.81 g, yield 45%) were isolated as a yellow powder. The spectroscopic properties of the synthetized **14** and **15** are in good agreement with the previous publications.[6]

*Synthesis of rhodol derivatives **11a-n**.*

Benzophenone **15** or **16** (190.7 mg, 0.50 mmol, 1 equivalent) and phenol/naphthol (1.00 mmol, 2 equivalent) was dissolved in methanesulfonic acid (1.0 mL) and stirred at 100 °C for 30-60 minutes, followed by HPLC-MS. Dark red solution was formed. (Methanol avoided.). The

mixture was cooled down to room temperature, diluted with acetonitrile (3 mL), then carefully 2 mL of distilled water followed by 0.9 mL triethylamine was added using an icy bath. The pH ~4-6 was set. The dark red solution was directly purified by preparative HPLC (water–acetonitrile–0.1% TFA, using the gradient method). After purification, the fractions were lyophilized. The product (**11**) was isolated as a dark red powder.

*Synthesis of **14** rhodol with azide function.*

11d (50.0 mg, 0.103 mmol) was dissolved in 1 mL dry DMF, N,N-diisopropylethylamine (0.27 mL, 1.55 mmol, 15 equivalent) was added then cooled to 0 °C. TSTU (46.7 mg 0.155 mmol, 1.5 equivalent) in 1 mL dry DMF was added then stirred at room temperature for two hours. (LCMS: conversion>98%, some bis succinated product also formed). 10 equivalents of 3-azidopropylamine (104 mg, 1.03 mmol) were added, then stirred overnight. After 20 hours reaction time, based on LC-MS measurements, small amount of product **17** was formed. Additional 10 equivalent of 3-azidopropylamine (104 mg, 1.03 mmol) was added, stirred for 30 minutes. Based on LCMS the conversion was almost 100%, the desired product was formed. The mixture was evaporated under reduced pressure. The dark red solid residue was purified by preparative HPLC (water–acetonitrile–0.1% TFA, using the gradient method). After purification, the fractions were lyophilized. The desired product 4-((3-azidopropyl)carbamoyl)-2-(11-ethyl-12-oxo-2,3,6,7-tetrahydro-1H,5H,12H-chromeno[2,3-f]pyrido[3,2,1-ij]quinolin-9-yl)benzoic acid (**17**) was isolated as red solid (32 mg, 65%).

SDS-PAGE and densitometry

Non-reducing glycine-SDS-PAGE at 10% acrylamide running were performed following standard lab procedures. A 4% stacking gel was used and a broad-range MW marker (4.6–300 kDa, ProSieve QuadColor Protein Marker, Lonza) was co-run to estimate protein weights. Samples (10 μL at 5 μM) were mixed with loading buffer (3 μL, composition for 6×SDS: 1 g SDS, 3 mL glycerol, 6 mL 0.5 M Tris buffer pH = 6.8; 2 mg Coomassie-blue R250 in 10 mL), heated at 65 °C for 5minutes. Samples were subsequently loaded into the wells in a volume of 13 μL. All gels were run at constant 200 mA for 45 minutes. Gels were stained using a Coomassie stain (0.12 g Coomassie-blue G-250, 0.10 g Coomassie-blue R-250, 500 mL MeOH, 400 mL distilled water, 100 mL acetic acid), after washing it was rested at room temperature for 16 h in water-ethanol mixture. Then the gels were imaged using a HP Laserjet 1132 MFP scanner at 600 dpi. Images were saved under default brightness, contrast, and gamma settings. Densitometry was performed using ImageJ. Background subtraction was achieved using the built-in plugin with a rolling ball radius of 30, sliding paraboloid, and smoothing. Brightness and contrast settings were auto adjusted within the software.

Photophysical measurements

Absorbance measurements were carried out on a Jasco V-750 Spectrophotometer (standard cell quartz cuvette with 1 cm light path length, 1 nm bandwidth, 400 nm/min recording speed) operating at 21 °C. We measured the absorbance spectra from 220 nm to 750 nm. The UV/Vis absorbance measurements of trastuzumab conjugate were carried out on SpectraMax iD5 Multi-Mode Microplate Reader (Molecular Devices; San Jose, CA). Before the measurements buffer exchange was performed six times with Sartorius Vivaspin 500 10000 MWCO at 15000 g for 10 minutes for each time. Sample buffer was used as blank for baseline correction with extinction coefficients; ε_{280}= 232677 $M^{-1}cm^{-1}$ and ε_{543}= 0 $M^{-1}cm^{-1}$ for trastuzumab (in the equation T); ε_{280}= 32267 $M^{-1}cm^{-1}$for ε_{543}= 52923 $M^{-1}cm^{-1}$ for 5-((3-azidopropyl)carbamoyl)-2-(11-ethyl-12-oxo-2,3,6,7-tetrahydro-1*H*,5*H*,12*H*-chromeno[2,3-*f*]pyrido[3,2,1-*ij*]quinolin-9-yl)benzoic acid (**17**) measured in water/BBS. FAR values were calculated from Lambert-Beer equation for absorbance at 280 and 543 nm.

$$A_{280\,nm} = \varepsilon_{\mathbf{11d},280\,nm} \cdot l \cdot c_{\mathbf{11d}} + \varepsilon_{T,280\,nm} \cdot l \cdot c_T$$

$$A_{543\,nm} = \varepsilon_{\mathbf{11d},543\,nm} \cdot l \cdot c_{\mathbf{11d}} + \varepsilon_{T,543\,nm} \cdot l \cdot c_T$$

The fluorescence measurements were carried out on a Jasco FP8300 spectrofluorometer, in a standard cell quartz cuvette with 1 cm light path length. The widths of the excitation slit and the emission slit were both set to 2.5 nm with the scanning speed at 1000 nm/min. Pure solvents were used as blank correction. We used HEPES pH=7.4 buffer as solvent if not otherwise mentioned, and the concentration was 1 μM. We measured the absorbance spectra from 250 nm to 700 nm. The excitement spectra were recorded between 250 nm and 700 nm. For the emission spectra, we excited the sample at the excitation maximum, and recorded it from excitation maximum plus 10 nm to 800 nm.

The quantum yields were determined by recording the fluorescence spectra of a series of different concentrations. The gradient of the integrated fluorescence intensities plotted against absorbance at the excitation wavelength was used for the calculation of the quantum yields:

$$\Phi_x = \Phi_{St} \cdot \left(\frac{Grad_x}{Grad_{St}}\right) \cdot \left(\frac{\eta_x^2}{\eta_{St}^2}\right)$$

The **11a-n** compounds were excited at 500 nm; Rhodamine B was used as reference (Φ_F^{EtOH}=0.65). During the computations we used refraction coefficients from literature[6].

The solvent screen measurement was carried out with 5 μM solutions of the **11d** rhodol in acetonitrile, 1,4-dioxane, water, toluene, dichloromethane, tetrahydrofuran, ethanol, and ethyl acetate.

The photostability measurement was carried out with 5 μM solutions of compound **11d** in HEPES. We used 10W, 540 nm emitting diode, and measured the emission spectrum in every hour for 4 hours. During the photophysical measurements the solutions were excited at 380 nm.

Absorption and emission data of the synthetized novel compounds **11a–n** and 5-TAMRA.

Table S1. Detailed spectroscopic properties of the synthesized rhodols with julolidine moiety (**11a–n**) in HEPES (5 μM, pH = 7.4). Extension of Table 1 of the main text.

**Flourophore**		**Absorption**		**Excitation**	**Emission**	**Stokes shift**[a]	**QY**	**Brightness**[b]
		λ_{abs}^{max} [nm]	ε (λ_{abs}^{max}) [$M^{-1}cm^{-1}$]	λ_{exc}^{max} [nm]	λ_{em}^{max} [nm]	Δλ [nm]	Φ_F [-]	B [$M^{-1}cm^{-1}$]
**11a**	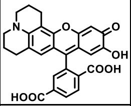	548	93 480	546	585	37	0.35	32 678
**11b**	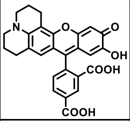	549	60 623	547	589	40	0.31	19 021
**11c**	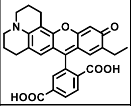	543	19 856	542	571	28	0.20	3 995
**11d**	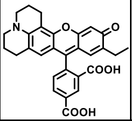	543	71 176	542	577	34	0.50	35 663
**11e**	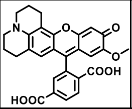	548	25 180	547	582	34	0.72	18 712
**11f**	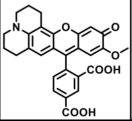	548	60 023	547	586	38	0.48	28 912
**11g**	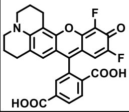	553	54 069	551	580	27	0.35	19 153
**11h**	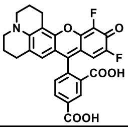	552	58 205	550	584	32	0.14	7 955
**11i**	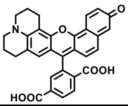	570	16 924	568	606	36	0.03	526
**11j**	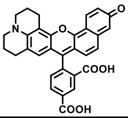	570	19 020	568	603	33	0.03	673
**11k**	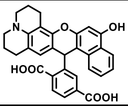	552	44 927	551	592	40	0.49	22 142
**11l**	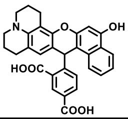	553	49 360	551	594	41	0.38	18 574
**11m**	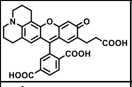	543	79 643	540	573	30	0.46	36 473
**11n**	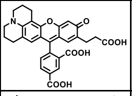	544	66 076	543	579	35	0.47	31 055
**5-TAMRA**	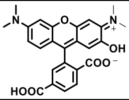	550	76 960	551	574	24	0.41	31 554

[a]Stokes shifts are provided as wavelength difference, $\Delta\lambda_{max} = \lambda_{max}(EM) - \lambda_{max}(EX)$. [b]Brightness values are defined as the product of the extinction coefficient (*ε*) and the fluorescence quantum yield (*QY*).

Trastuzumab reconjugation with DBPD-CO

It was prepared by following literature procedure.[7] The resulting T-CO conjugate (**18**) was buffer exchanged using Vivaspin500 10 kDa MWCO membrane filter twice. At the last step the retentate was further purified from the small molecular reactants with ZebaSpin 7 kDa MWCO columns. The conjugate was investigated with UV/Vis absorbance measurement and non-reducing SDS-PAGE (see for example at Figure 2,). The DAR was calculated from the absorbance spectra and it is resulted DAR=4.

*Click reaction of **17** and T-CO conjugate (**12**) resulting fluorescent conjugate (**13**)*

To 13 µL 59.77. µM T-CO (**S1**) solution (0.00078 µmol) 0.39 µL 20 mM **17** rhodol (0.0078 µmol, 10 equiv.) was added. The reaction mixture was gently shaken overnight. The next morning the buffer was removed using Vivaspin500 10 kDa MWCO membrane filter twice. At the last step the retentate was further purified from the small molecular reactants with ZebaSpin 7 kDa MWCO columns. The **19** conjugate was investigated with UV/Vis absorbance measurement (Figure S49.) and non-reducing SDS-PAGE. The average FAR determined from UV gives about 4.

$$A_{280\,nm} = \varepsilon_{\mathbf{14},280\,nm} \cdot l \cdot c_{\mathbf{11d}} + \varepsilon_{T,280\,nm} \cdot l \cdot c_T$$

$$A_{543\,nm} = \varepsilon_{\mathbf{14},543\,nm} \cdot l \cdot c_{\mathbf{11d}} + \varepsilon_{T,543\,nm} \cdot l \cdot c_T$$

$$0.0497 = 32267\ M^{-1}cm^{-1} \cdot 0.05\ cm \cdot c_{\mathbf{11d}} + 232677\ M^{-1}cm^{-1} \cdot 0.05\ cm \cdot c_T$$

$$0.0303 = 52923\ M^{-1}cm^{-1} \cdot 0.05\ cm \cdot c_{\mathbf{11d}} + 0\ M^{-1}cm^{-1} \cdot 0.05\ cm \cdot c_T$$

$$c_T = 1.5\ \mu M$$

$$c_T = 1.5\ \mu M;\ c_{\mathbf{11d}} = 6.0\ \mu M; FAR = {}^{c_{\mathbf{11d}}}/_{c_T} = 4.05$$

Cell lines and culture conditions

MCF-7 (HER2- human breast adenocarcinoma), NCI-N87-GFP (HER2+ human gastric carcinoma), and KBR- TurboRFP602S (HER2+ human breast cancer) cell lines were cultured in sterile culture flasks at 37 °C in a humidified atmosphere with 5% CO_2 in an incubator. The cells were cultured in DMEM-F12 (Dulbecco's Modified Eagle's Medium, Nutrient Mixture F12; Gibco, Waltham, Massachusetts, USA), supplemented with 10% FBS (Fetal Bovine Serum; Euroclone, Pero, Italy), 100 units/mL penicillin, 100 mg/mL streptomycin, and 0.25 µg/mL amphotericin B (Lonza, Basel, Switzerland).

All work with the cell lines were performed in a laminar flow biosafety cabinet.

Flow cytometry

Samples were analyzed using an Attune NxT flow cytometer (ThermoFisher Scientific). Trastuzumab fluorescence (BL1-H) was measured with 488 nm excitation and 530/30 nm

emission, **13** fluorescence (YL1-H) was measured with 561 nm excitation and 585/16 nm emission. Analysis was performed using Attune NxT 3.1.2 software.

Preparing labelled cells with T-Rhodol (**13**) for FACS

Human breast cancer cell lines NCI-N87 GFP (HER2 positive) and MCF-7 (HER2 negative) were applied. The cells were cultured in DMEM-F12 medium (Gibco) supplemented with 10% foetal bovine serum (EuroClone), 100 units/mL penicillin, 100 mg/mL streptomycin, and 0.25 µg/mL amphotericin B (Lonza). Both cell lines were maintained at 37 °C in a humidified incubator with 5% CO_2.

Cultured cells were harvested using 0.25% trypsin in 2.21 mM EDTA (Corning) and washed with washing buffer (pH=7.4 PBS buffer containing 1% FBS). Cells were put 1.5 mL microtubes and stained with 10 µg/mL of the trastuzumab conjugate (**13**) (the antibody was diluted in 3% bovine serum albumin in PBS). The cells were incubated in 200 µL staining volume for 30 minutes at room temperature, then washed three times in 200 µL washing buffer. The cells were also stained with 100-fold diluted secondary antibody (anti-human IgG FITC) for 1 hour at room temperature, then washed three times in washing buffer. Finally, cells were suspended in 500 µL washing buffer followed by flow cytometry analysis.

Immunocytochemistry

Glass coverslips were sterilized in 90% ethanol, and after drying they were placed in 6 well cell culture plates (Starstedt, Nümbrech, Germany). Cells were suspended using 0.25% trypsin in 2.21 mM EDTA (Corning, Manassas, VA, USA) and washed with PBS (Gibco, Waltham, Massachusetts, USA supplemented with 1% FBS). Approximately 200 000 cells were placed in each well in 1ml of culture medium. After 24 hours of incubation at 37 °C, the cells were washed 3 times with PBS (Gibco, Waltham, Massachusetts, USA) and then fixed in 4% paraformaldehyde (Thermo Scientific, Waltham, Massachusetts, USA) for 10 minutes. After three PBS washes, we blocked the non-specific protein binding sites with 30 minutes of BSA (Sigma, St. Louis, Missouri, USA) incubation. The cells were incubated with the dye-conjugated primary antibody (**13**) in 20 µg/mL concentration for 1 hour. After three PBS washes the samples were mounted with Vectashield-DAPI (for nucleus staining, Vector, Newark, California, USA). The samples were investigated with both a Zeiss LSM 710 confocal microscope (Zeiss, Jena, Germany) and 3D AO laser-scanning two-photon microscope (Femto3D Atlas, Femtonics, Hungary).

In vitro two-photon imaging

Nci-rohodol positive cells (Her2+), Nci-rhodol negative cells (Her2+) and Mcf7-rhodol positive cells (Her2+) in culture were measured with in vitro 2-photon microscopy. After the visualization of the cell culture surface with CCD camera two-photon images were performed. All two-photon experiments were made with an AO laser-scanning microscope

(Femto3D Atlas, Femtonics, Hungary). Femtosecond laser pulses were provided by a Mai Tai HP laser (Spectra Physics) at 840 nm and 750 nm wavelength. The pulse length was set to 100 fs below the objective using the motorized four-prism sequence of the ATLAS microscope (4DBCU unit, Femtonics).

We used a 16× Nikon CFI LWD Plan Fluorite Objective water-immersion objective lens with 0.8 NA and 3 mm WD (N16XLWD-PF). Images were recorded in red and green channels. Maximum intensitiy projection of Her2+ cells were performed from 9 plane. The resolution of the images (776 by 1000 pixels) was 0.48 μm. The excitation was delivered to the sample, and the fluorescence signal was collected, using an XLUMPlanFI20×/1.0 lens (Olympus, 20×, NA 1.0). The fluorescence data separated using dichroic mirrors (700dcxru, Chroma Technology) and delivered to GaAsP photomultiplier tubes (H7422P-40-MOD, Hamamatsu). Images were recorded in red and green channels (red:605/70, green: 520/60). Fluorescence information was calculated using the built-in analysis tools in the acquisition software (MES, Femtonics).

The 2-photon sensitivity of the different dyes was measured similarly to the above. The dyes were filled into a borosilicate glass capillary and then adjusted to the focal point under the microscope objective. After that, we took a high-resolution two-photon image of the dye-filled capillary. Images were recorded in red and green channels. Afterwards, the fluorescence values (number of emitted photons) of the different dyes were measured at different wavelengths (700-900 nm). Fluorescence information was calculated using the built-in analysis tools in the acquisition software (MES, Femtonics) and ImageJ Fiji program.

Chemical characterization of **15, 16**, rhodol derivatives **11a-n** and **14**

*2-(11-Hydroxy-12-oxo-2,3,6,7-tetrahydro-1H,5H,12H-chromeno[2,3-f]pyrido[3,2,1-ij]quinolin-9-yl)terephthalic acid (**11a**)*

^{1}H NMR (500 MHz, DMSO-d_6) δ 8.30 – 8.21 (m, 2H, 2×ArH), 7.82 (s, 1H, ArH), 6.86 (s, 1H, ArH), 6.62 (s, 1H, ArH), 6.16 (s, 1H, ArH), 3.44 (d, J = 14.9 Hz, 2H, CH_2), 3.41 – 3.32 (m, 2H, CH_2), 3.03 – 2.81 (m, 2H, CH_2), 2.60 (s, 2H, CH_2), 2.03 – 1.86 (m, 2H, CH_2), 1.84 – 1.72 (m, 2H, CH_2) ppm; ^{13}C NMR (126 MHz, DMSO-d_6) δ 166.5, 116.4, 151.0, 150.1, 147.8, 135.0, 134.7, 131.5, 131.1, 130.9, 125.2, 125.1, 124.2, 114.2, 113.09, 113.07, 107.50, 107.47, 105.01, 104.96, 102.6, 50.7, 50.2, 27.2, 20.5, 19.9, 19.6 ppm. HRMS: calcd. for $C_{27}H_{22}NO_7$: 472.1396, $[M+H]^+$ found: 472.1392.

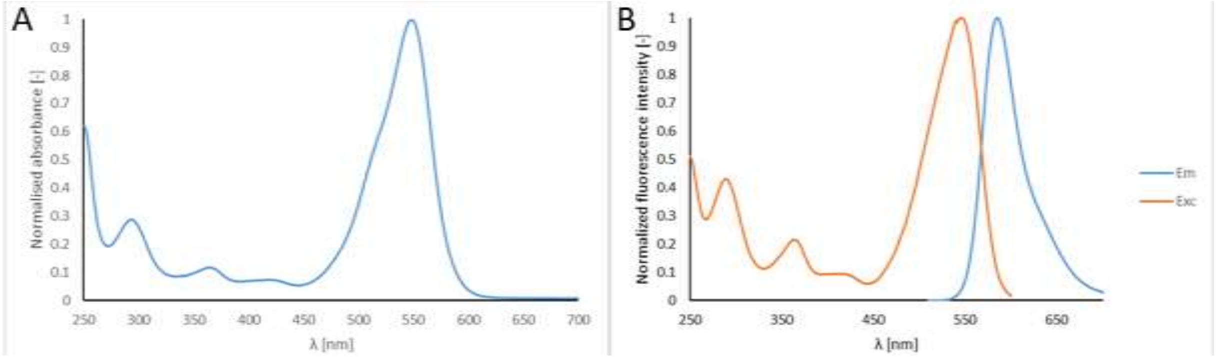

Figure S1. (A) Normalized absorption, (B) excitation (orange) and fluorescence emission spectra (blue) of **11a** in HEPES buffer (c= 5 μM, pH = 7.4).

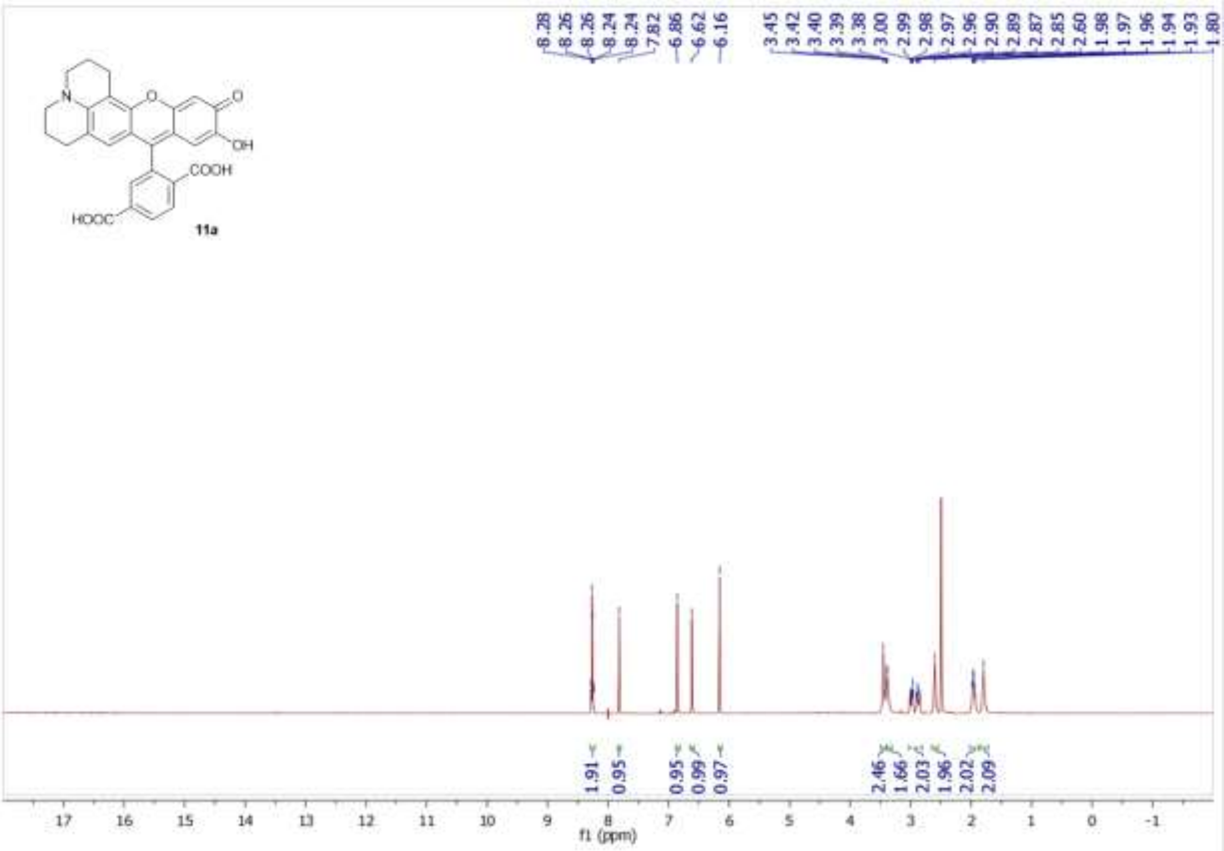

Figure S2. ^{1}H NMR spectrum of **11a** recorded at 500 MHz in DMSO-d_6.

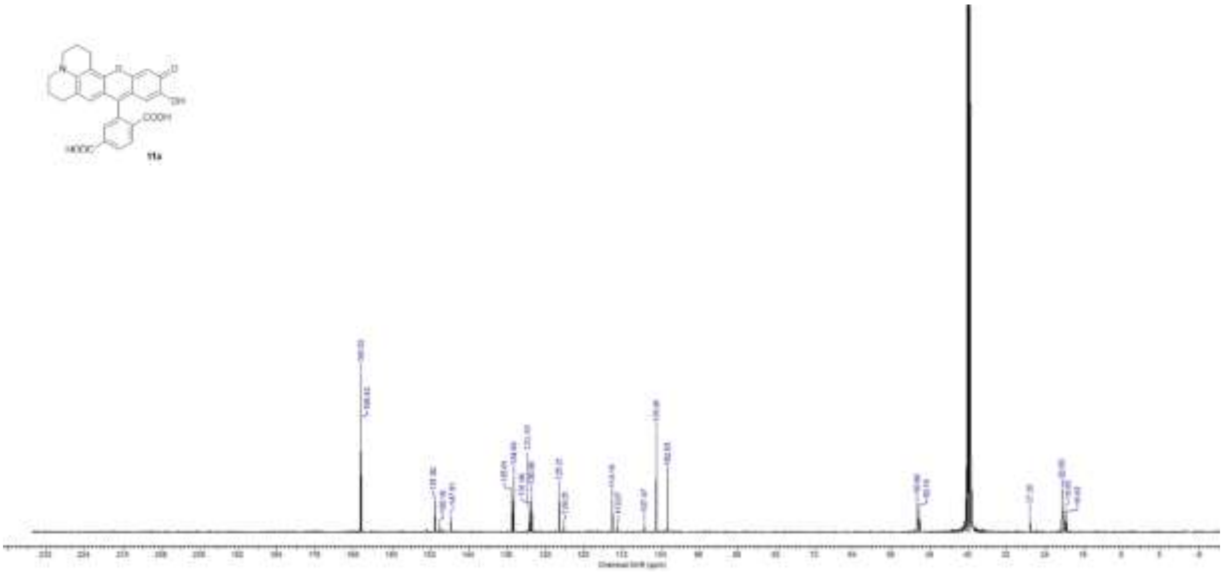

Figure S3. ^{13}C NMR spectrum of **11a** recorded at 126 MHz in DMSO-*d6*.

*4-(11-Hydroxy-12-oxo-2,3,6,7-tetrahydro-1H,5H,12H-chromeno[2,3-f]pyrido[3,2,1-ij]quinolin-9-yl)isophthalic acid (**11b**)*

^{1}H NMR (500 MHz, DMSO-d_6) δ 8.73 (s, 1H, ArH), 8.35 (d, *J* = 7.8 Hz, 1H, ArH), 7.55 (d, *J* = 7.9 Hz, 1H, ArH), 7.16 (s, 1H, ArH), 6.78 (s, 1H, ArH), 6.37 (s, 1H, ArH), 3.63 – 3.47 (m, *J* = 18.8 Hz, 4H, 2×CH_2), 3.06 – 2.89 (m, 2H, CH_2), 2.73 – 2.64 (m, 2H, CH_2), 2.06 – 1.91 (m, 2H, CH_2), 1.91 – 1.77 (m, 2H, CH_2) ppm; ^{13}C NMR (126 MHz, DMSO-d_6) *δ* 166.6, 166.0, 159.1, 158.7, 158.5, 152.3, 152.2, 151.1, 146.6, 138.6, 133.5, 133.0, 132.0, 131.8, 131.4, 126.7, 125.9, 114.8, 114.5, 109.7, 105.1, 102.9, 51.2, 50.7, 27.2, 20.3, 19.7, 19.4 ppm. HRMS: calcd. for $C_{27}H_{22}NO_7$: 472.1390, $[M+H]^+$ found: 472.1387.

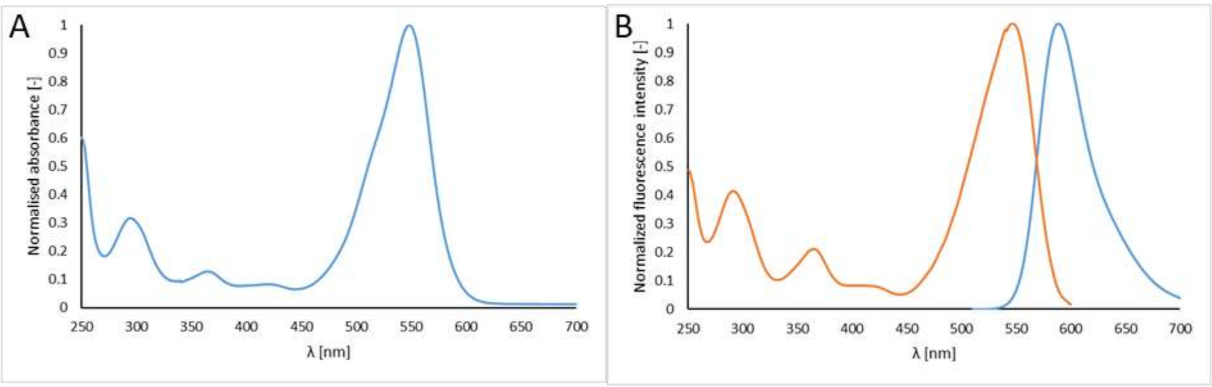

Figure S4. (A) Normalized absorption, (B) excitation (orange) and fluorescence emission spectra (blue) of **11b** in HEPES buffer (c= 5 µM, pH = 7.4).

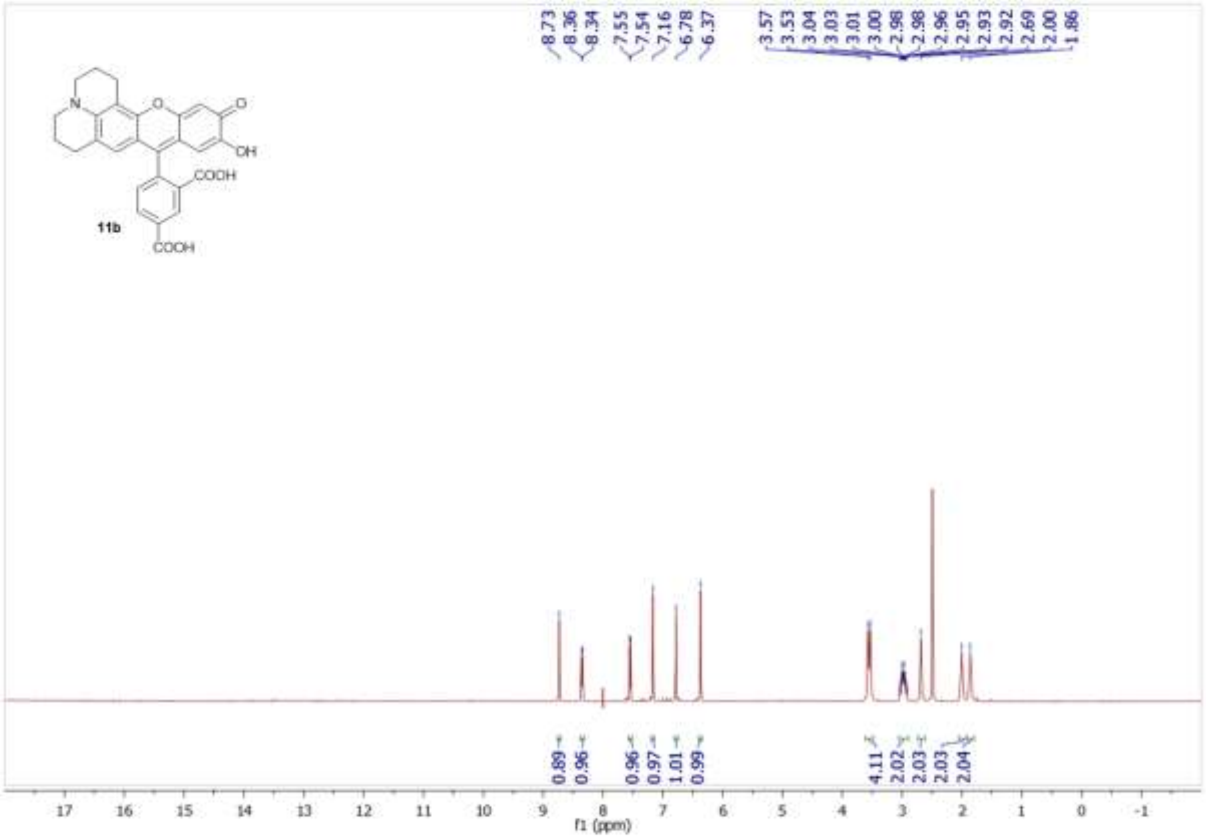

Figure S5. ^{1}H NMR spectrum of **11b** recorded at 500 MHz in DMSO-d_6.

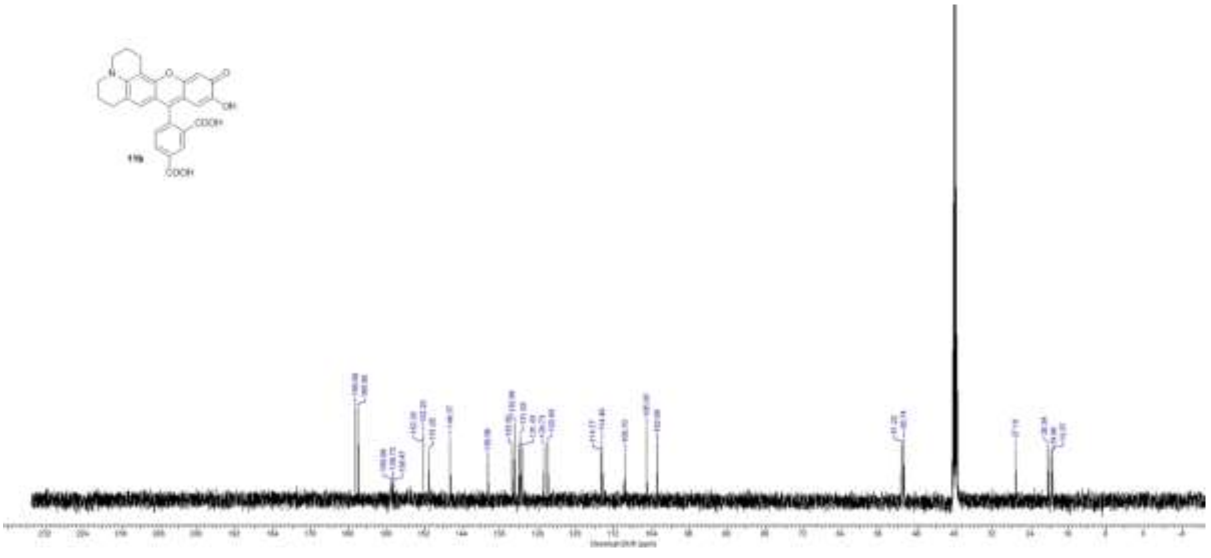

Figure S6. ^{13}C NMR spectrum of **11b** in DMSO-d_6 recorded at 126 MHz.

*2-(11-Ethyl-12-oxo-2,3,6,7-tetrahydro-1H,5H,12H-chromeno[2,3-f]pyrido[3,2,1-ij]quinolin-9-yl)terephthalic acid (**11c**)*

^{1}H NMR (400 MHz, DMSO-d_6) δ 8.31 – 8.26 (m, J = 1.0 Hz, 2H, 2×ArH), 7.85 (s, 1H, ArH), 7.15 – 7.08 (m, 1H, ArH), 6.80 (s, 1H, ArH), 6.74 – 6.69 (m, 1H, ArH), 3.61 – 3.49 (m, 4H, 2×CH_2), 3.03 – 2.97 (m, 4H, 2×CH_2), 2.71 – 2.64 (m, 2H, CH_2), 2.07 – 1.99 (m, J = 13.8 Hz, 2H, CH_2), 1.92 – 1.82 (m, J = 13.1 Hz, 2H, CH_2), 1.00 (t, J = 7.4 Hz, 3H, CH_3) ppm; ^{13}C NMR cannot be measured because of solubility problems. HRMS: calcd. for $C_{29}H_{26}NO_6$: 484.1754, $[M+H]^+$ found: 484.1758.

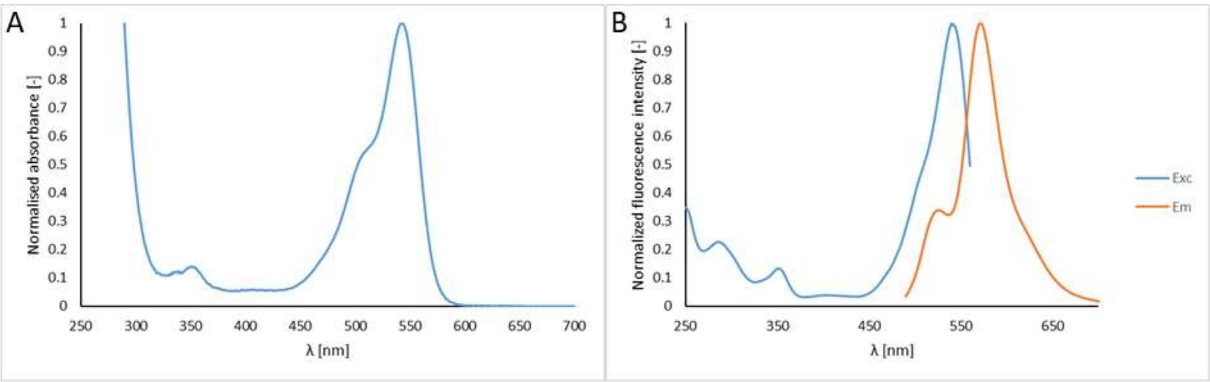

Figure S7. (A) Normalized absorption, (B) excitation (blue) and fluorescence emission spectra (orange) of **11c** in HEPES buffer (c= 5 µM, pH = 7.4).

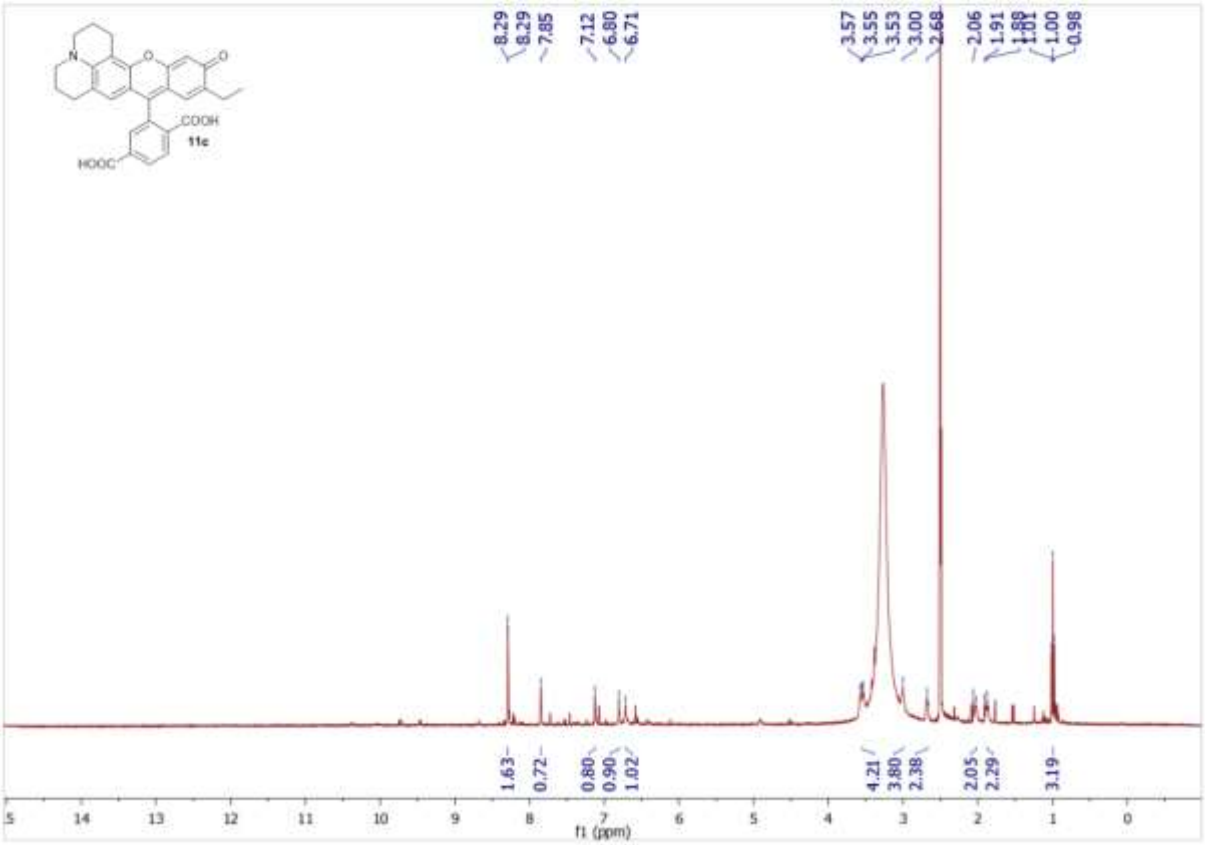

Figure S8. ^{1}H NMR spectrum of **11c** recorded at 400 MHz in DMSO-d_6.

*4-(11-Ethyl-12-oxo-2,3,6,7-tetrahydro-1H,5H,12H-chromeno[2,3-f]pyrido[3,2,1-ij]quinolin-9-yl)isophthalic acid (**11d**)*

^{1}H NMR (500 MHz, DMSO-d_6) δ 8.68 (s, 1H, ArH), 8.33 (d, J = 7.8 Hz, 1H, ArH), 7.53 (d, J = 7.9 Hz, 1H, ArH), 7.14 (s, 1H, ArH), 6.82 (s, 1H, ArH), 6.73 (s, 1H, ArH), 3.62 – 3.48 (m, 4H, 2×CH_2), 3.06 – 2.91 (m, 2H, CH_2), 2.72 – 2.62 (m, 2H, CH_2), 2.48 – 2.42 (m, 2H, CH_2), 2.07 – 1.93 (m, 2H, CH_2), 1.93 – 1.78 (m, 2H, CH_2), 0.99 (t, J = 7.4 Hz, 3H, CH_3) ppm; ^{13}C NMR (126 MHz, DMSO-d_6) δ 166.6, 166.3, 154.1, 152.1, 144.4, 133.8, 133.0, 132.1, 131.6, 131.4, 130.9, 128.2, 126.4, 123.8, 119.5, 118.1, 113.7, 105.6, 102.0, 51.3, 50.8, 27.1, 22.9, 20.4, 19.8, 19.5, 14.2 ppm. HRMS: calcd. for $C_{29}H_{26}NO_6$: 484.1754, $[M+H]^+$ found: 484.1757.

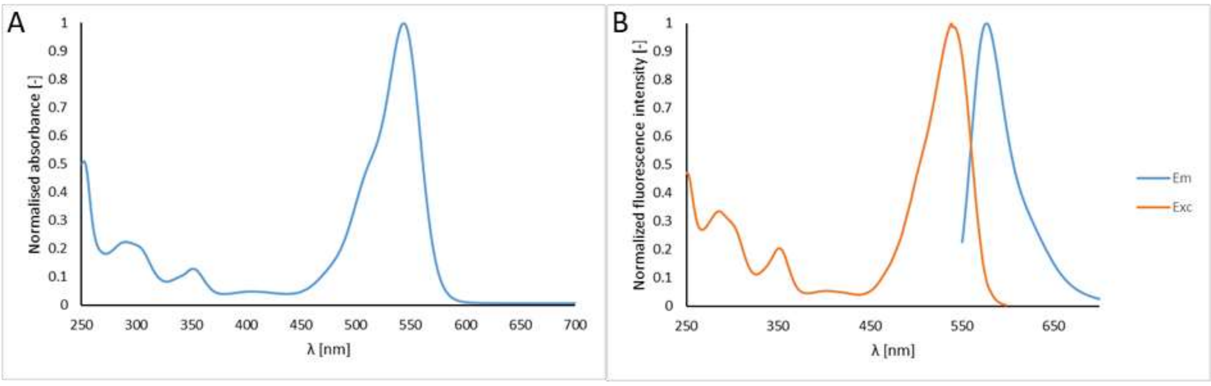

Figure S9. (A) Normalized absorption, (B) excitation (blue) and fluorescence emission spectra (orange) of **11d** in HEPES buffer (c= 5 µM, pH = 7.4).

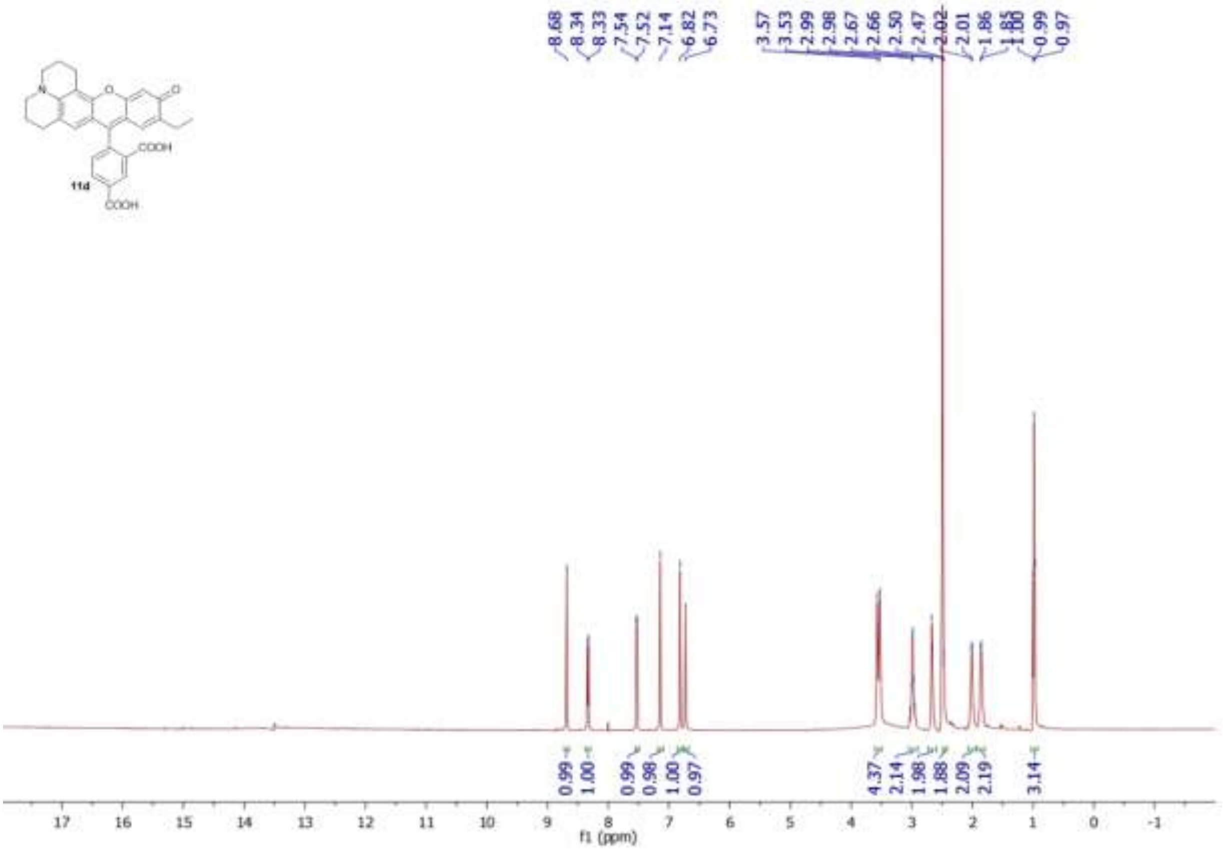

Figure S10. ^{1}H NMR spectrum of **11d** recorded at 500 MHz in DMSO-d_6.

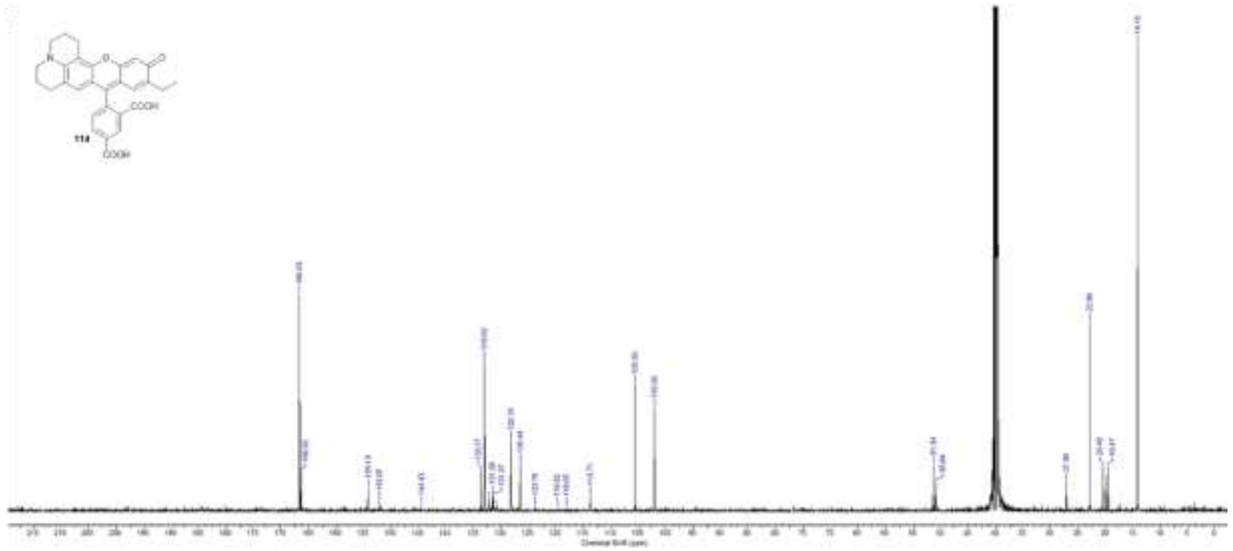

Figure S11. ^{13}C NMR spectrum of **11d** in DMSO-d_6 recorded at 126 MHz.

*2-(11-Methoxy-12-oxo-2,3,6,7-tetrahydro-1H,5H,12H-chromeno[2,3-f]pyrido[3,2,1-ij]quinolin-9-yl)terephthalic acid (**11e**)*

^{1}H NMR (500 MHz, DMSO-d_6) δ 8.32 – 8.31 (m, 2H, 2×ArH), 7.89 (s, 1H, ArH), 7.26 (s, 1H, ArH), 6.81 (s, 1H, ArH), 6.38 (s, 1H, ArH), 3.63 – 3.53 (m, 7H, 2×CH_2, OCH_3), 3.09 – 2.96 (m, 2H, CH_2), 2.74 – 2.67 (m, 2H, CH_2), 2.06 – 1.98 (m, 2H, CH_2), 1.91 – 1.82 (m, 2H, CH_2) ppm; ^{13}C NMR (126 MHz, DMSO-d_6) *δ* 166.0, 165.9, 158.1, 157.9, 157.4, 152.4, 152.1, 151.0, 134.6, 134.3, 131.3, 130.9, 130.8, 127.9, 126.9, 125.7, 125.0, 122.2, 115.2, 113.9, 106.8, 104.7, 102.7, 55.9, 50.9, 50.4, 26.7, 21.1, 19.7, 19.1, 18.8 ppm. HRMS: calcd. for $C_{28}H_{24}NO_7$: 486.1547, $[M+H]^+$ found: 486.1550.

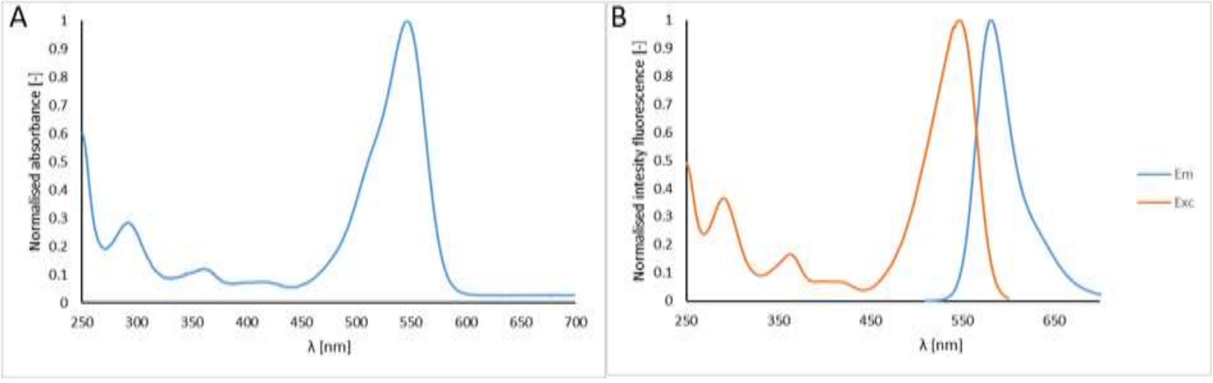

Figure S12. (A) Normalized absorption, (B) excitation (blue) and fluorescence emission spectra (orange) of **11e** in HEPES buffer (c= 5 µM, pH = 7.4).

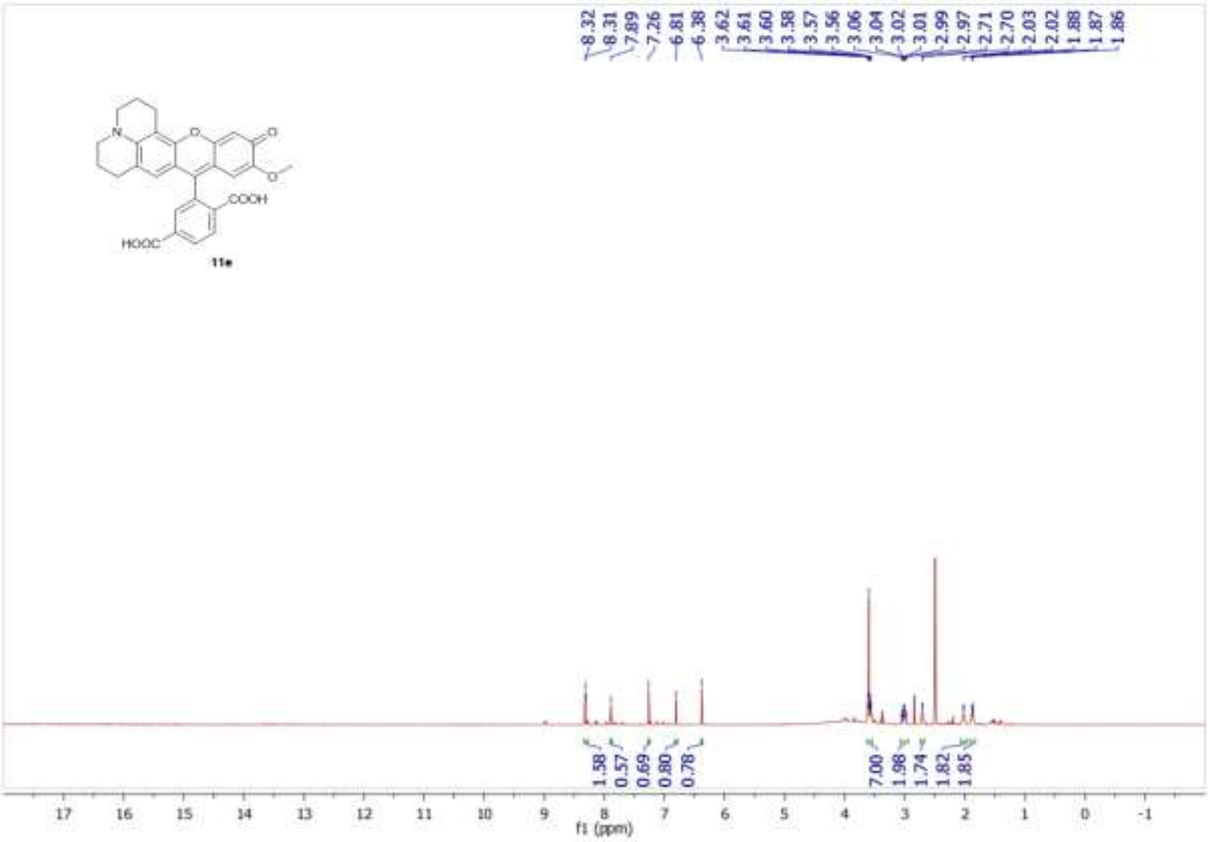

Figure S13. ^{1}H NMR spectrum of **11e** recorded at 500 MHz in DMSO-d_6.

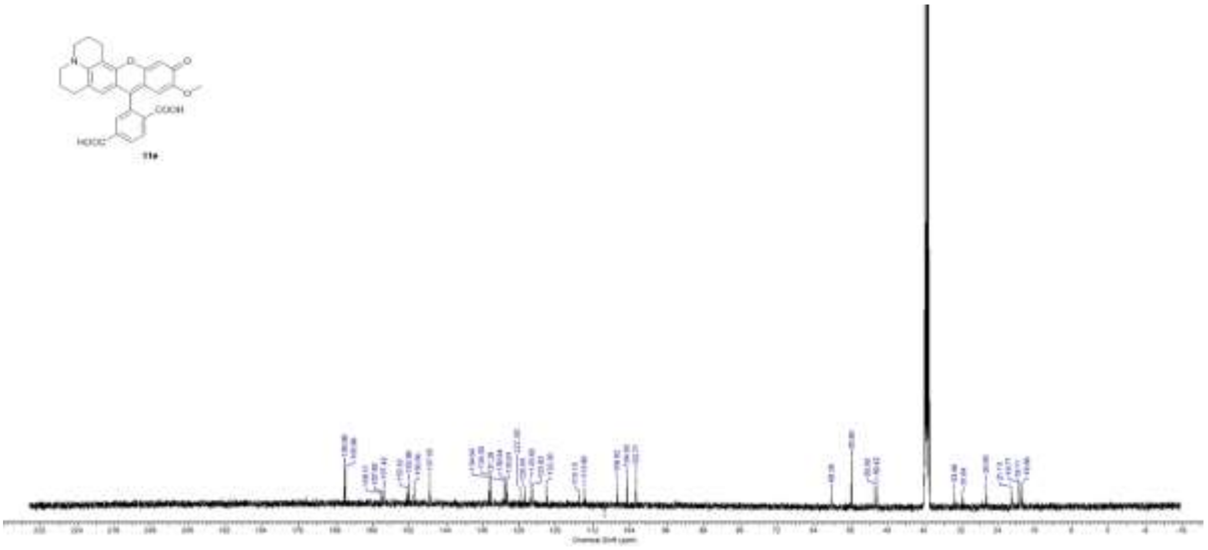

Figure S14. ^{13}C NMR spectrum of **11e** in DMSO-d_6 recorded at 126 MHz.

*4-(11-Methoxy-12-oxo-2,3,6,7-tetrahydro-1H,5H,12H-chromeno[2,3-f]pyrido[3,2,1-ij]quinolin-9-yl)isophthalic acid (**11f**)*

^{1}H NMR (500 MHz, DMSO-d_6) δ 8.71 (d, J = 0.8 Hz, 1H, ArH), 8.37 – 8.32 (m, 1H, ArH), 7.56 (d, J = 7.9 Hz, 1H, ArH), 7.20 (d, J = 6.0 Hz, 1H, ArH), 6.76 (s, 1H, ArH), 6.35 (s, 1H, ArH), 3.60 – 3.50 (m, 7H, 2×CH_2, OCH_3), 3.07 – 2.91 (m, 2H, CH_2), 2.69 (t, J = 5.9 Hz, 2H, CH_2), 2.00 (dd, J = 11.1, 5.3 Hz, 2H, CH_2), 1.91 – 1.79 (m, 2H, CH_2) pmm; ^{13}C NMR (126 MHz, DMSO-d_6) δ 166.6, 166.2, 159.1, 158.6, 158.3, 152.3, 152.1, 151.7, 148.4, 133.6, 133.0, 132.0, 131.9, 131.3, 126.8, 126.0, 114.8, 113.9, 107.1, 105.2, 103.3, 56.4, 51.3, 50.8, 27.2, 20.4, 19.7, 19.4 ppm. HRMS: calcd. for $C_{28}H_{24}NO_7$: 486.1547, $[M+H]^+$ found: 486.1556.

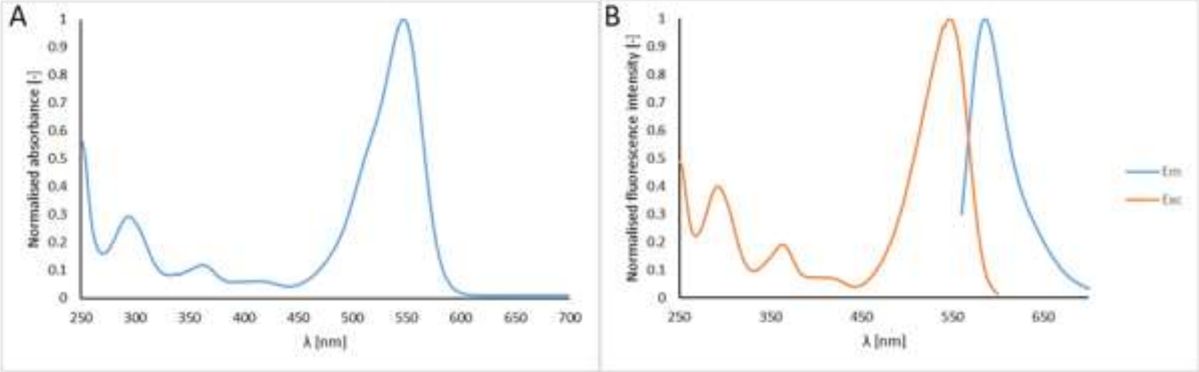

Figure S15. (A) Normalized absorption, (B) excitation (blue) and fluorescence emission spectra (orange) of **11f** in HEPES buffer (c= 5 µM, pH = 7.4).

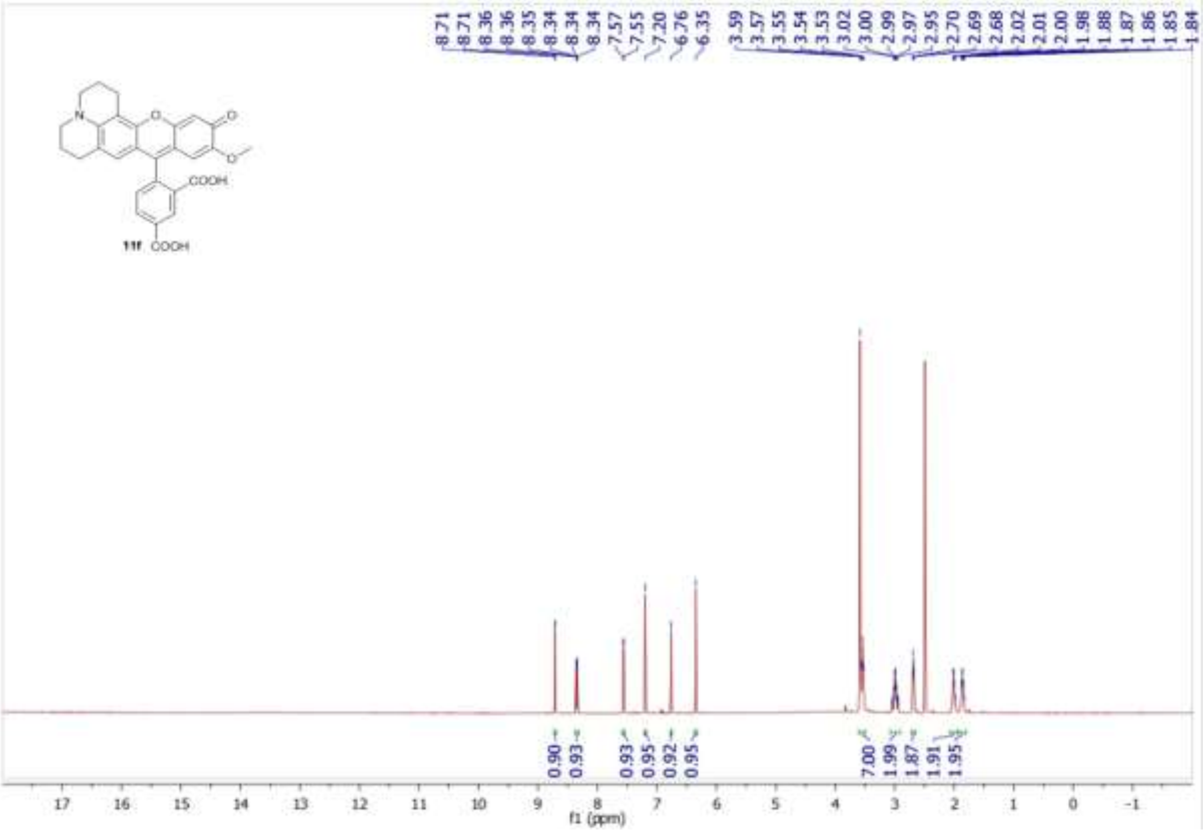

Figure S16. ^{1}H NMR spectrum of **11f** recorded at 500 MHz in DMSO-d_6.

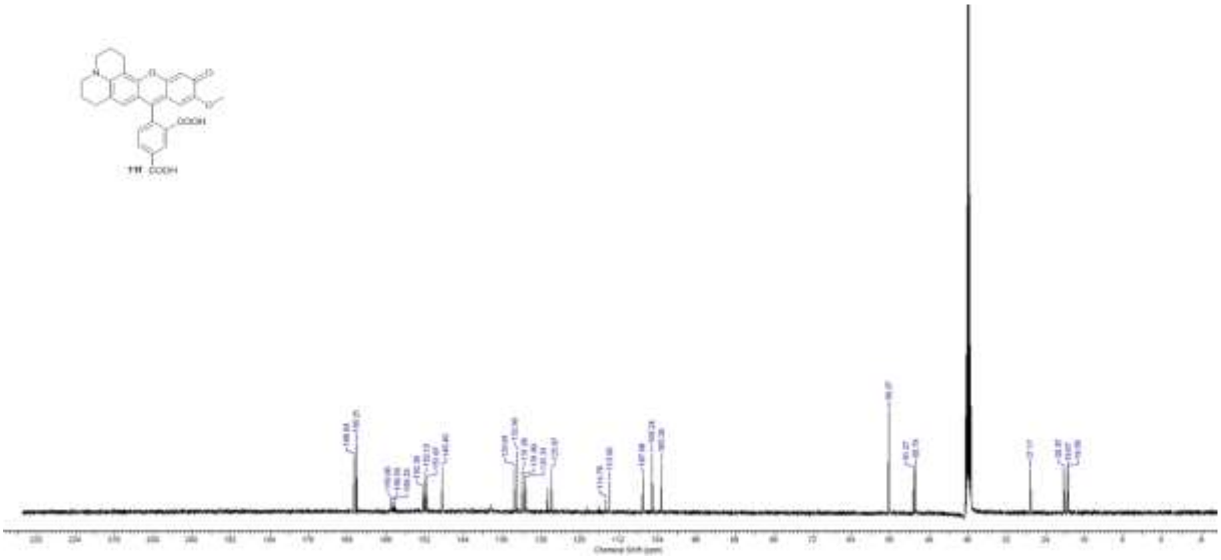

Figure S17. ^{13}C NMR spectrum of **11f** in DMSO-d_6 recorded at 126 MHz.

*2-(11,13-Difluoro-12-oxo-2,3,6,7-tetrahydro-1H,5H,12H-chromeno[2,3-f]pyrido[3,2,1-ij]quinolin-9-yl)terephthalic acid (**11g**)*

^{1}H NMR (300 MHz, DMSO-d_6) δ 8.26 (s, 2H, 2×ArH), 7.83 (s, 1H, ArH), 6.67 – 6.52 (m, 2H, ArH), 3.59 – 3.39 (m, 4H, 2×CH_2), 3.01 – 2.89 (m, 2H, 2×CH_2), 2.68 – 2.57 (m, 2H, CH_2), 2.06 – 1.94 (m, 2H, CH_2), 1.90 – 1.79 (m, 2H, CH_2) ppm; ^{13}C NMR (75 MHz, DMSO-d_6) *δ* 166.52, 166.46, 158.9, 150.6, 145.9, 139.6, 139.5, 134.9, 134.7, 131.3, 125.9, 124.6, 109.9, 108.5, 108.2, 105.9, 50.9, 50.4, 27.1, 20.5, 19.9, 19.6 ppm. HRMS: calcd. for $C_{27}H_{20}F_2NO_6$: 492.1253, $[M+H]^+$ found: 492.1264.

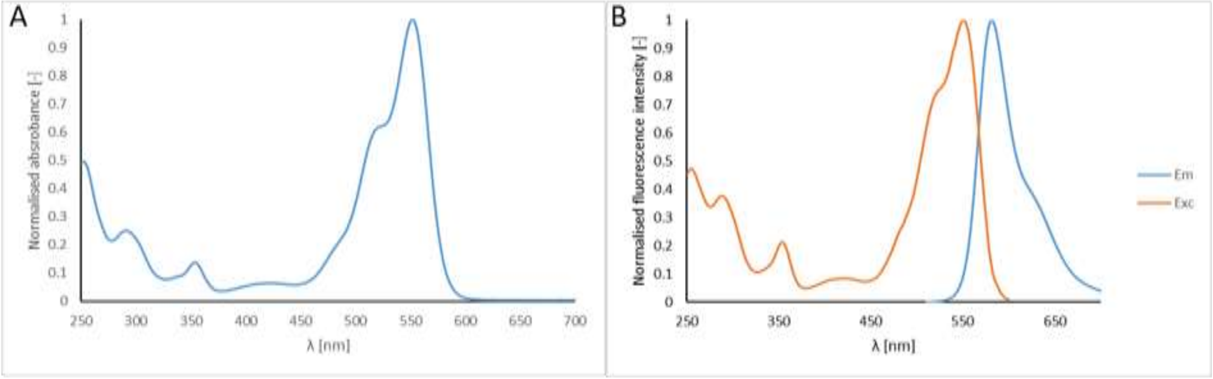

Figure S18. (A) Normalized absorption, (B) excitation (blue) and fluorescence emission spectra (orange) of **11g** in HEPES buffer (c= 5 µM, pH = 7.4).

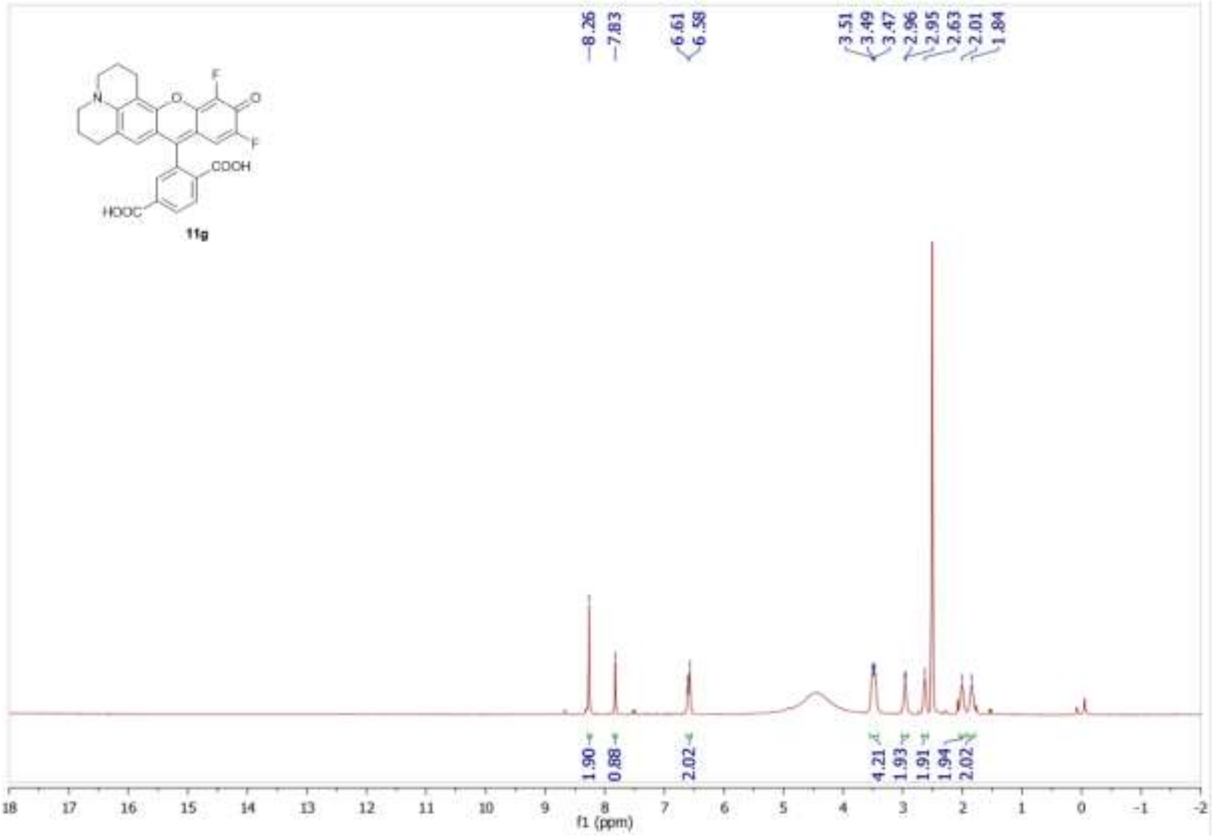

Figure S19. ^{1}H NMR spectrum of **11g** recorded at 300 MHz in DMSO-d_6.

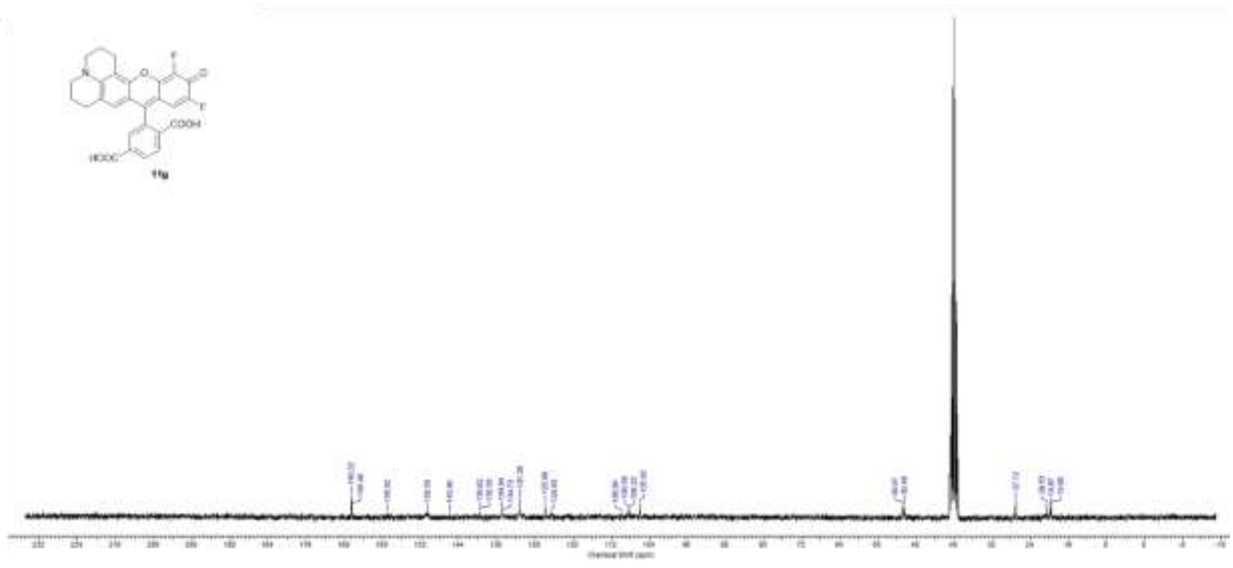

Figure S20. ^{13}C NMR spectrum of **11g** in DMSO-d_6 recorded at 75 MHz.

4-(11,13-Difluoro-12-oxo-2,3,6,7-tetrahydro-1*H*,5*H*,12*H*-chromeno[2,3-*f*]pyrido[3,2,1-*ij*]quinolin-9-yl)isophthalic acid (**11h**)

^{1}H NMR (500 MHz, DMSO-d_6) δ 8.64 (s, 1H, ArH), 8.29 (dd, J = 7.9, 1.7 Hz, 1H, ArH), 7.49 (d, J = 7.9 Hz, 1H, ArH), 6.53 (d, J = 11.3 Hz, 2H, 2×ArH), 3.43 (d, J = 20.2 Hz, 4H, 2×CH_2), 3.00 – 2.86 (m, 2H, CH_2), 2.61 (dd, J = 12.8, 7.2 Hz, 2H, CH_2), 2.03 – 1.91 (m, 2H, CH_2), 1.87 – 1.76 (m, 2H, CH_2) ppm; ^{13}C NMR (126 MHz, DMSO-d_6) δ 166.6, 166.3, 150.1, 142.4, 142.3, 140.5, 140.4, 133.7, 132.9, 131.5, 126.2, 125.6, 123.1, 108.9, 108.8, 108.2, 108.1, 105.9, 105.8, 50.6, 50.1, 27.2, 20.7, 20.0, 19.8 ppm. HRMS: calcd. for $C_{27}H_{20}F_2NO_6$: 492.1253, $[M+H]^+$ found: 492.1247.

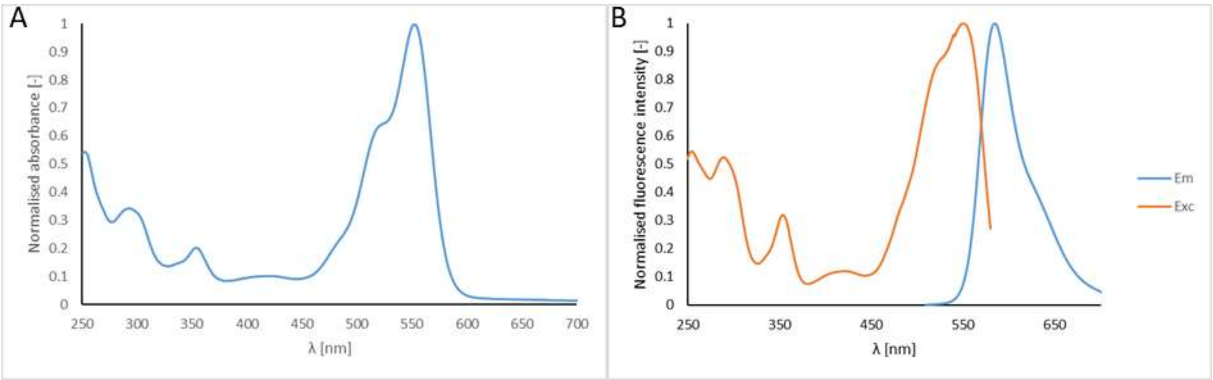

Figure S21. (A) Normalized absorption, (B) excitation (blue) and fluorescence emission spectra (orange) of **11h** in HEPES buffer (c= 5 µM, pH = 7.4).

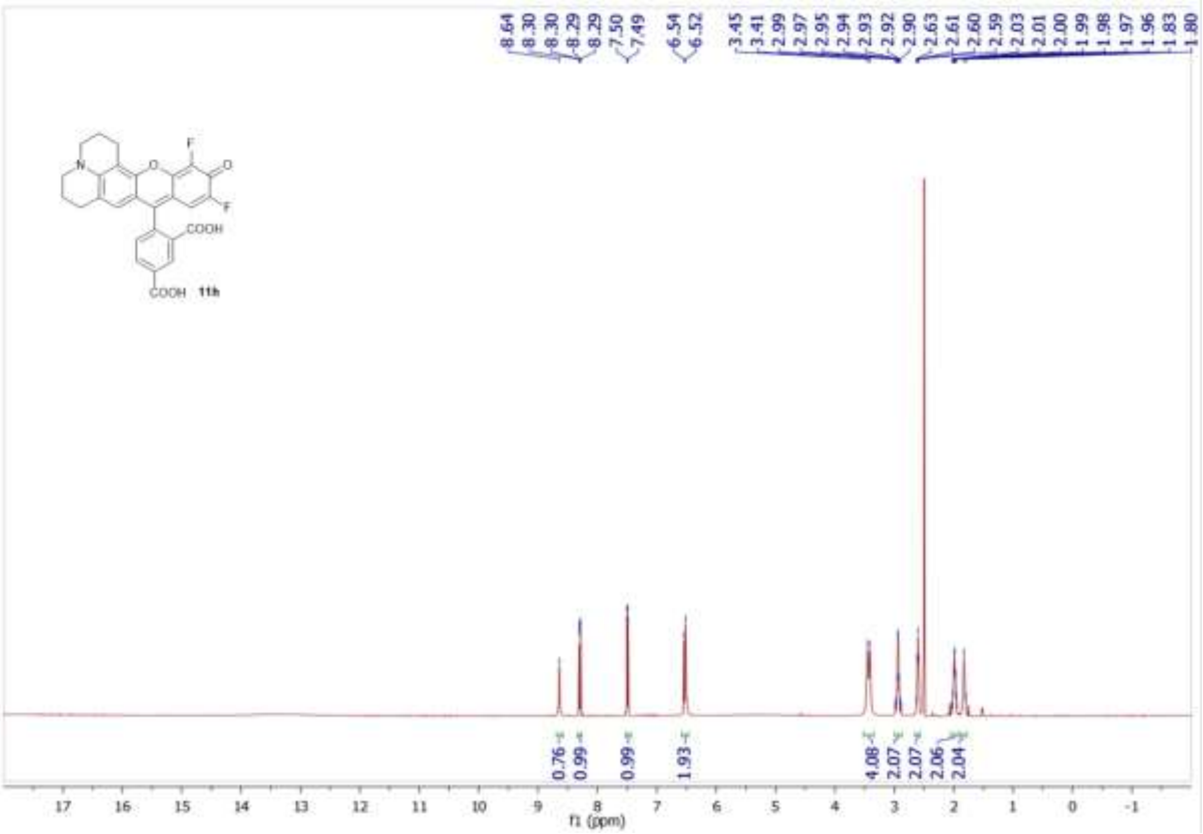

Figure S22. ^{1}H NMR spectrum of **11h** recorded at 500 MHz in DMSO-d_6.

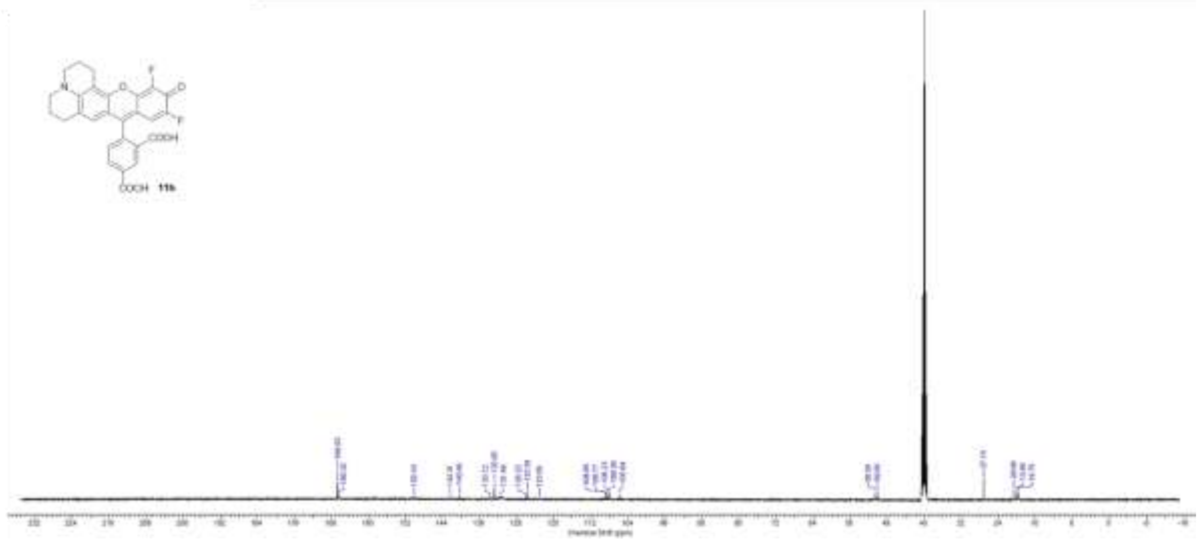

Figure S23. ^{13}C NMR spectrum of **11h** in DMSO-d_6 recorded at 126 MHz.

*2-(13-Oxo-2,3,6,7-tetrahydro-1H,5H,13H-benzo[7,8]chromeno[2,3-f]pyrido[3,2,1-ij]quinolin-9-yl)terephthalic acid (**11i**)*

^{1}H NMR (500 MHz, DMSO-d_6) δ 8.74 (s, 1H, ArH), 8.53 (d, *J* = 9.0 Hz, 1H, ArH), 8.36 (dd, *J* = 12.9, 11.9 Hz, 1H, ArH), 7.59 (dd, *J* = 20.0, 8.4 Hz, 2H, 2×ArH), 7.35 (d, *J* = 9.1 Hz, 1H, ArH), 7.29 (s, 1H, ArH), 6.90 (d, *J* = 8.9 Hz, 1H, ArH), 6.84 (s, 1H, ArH), 3.59 (d, *J* = 19.5 Hz, 4H, 2×CH_2), 3.08 (t, *J* = 5.9 Hz, 2H, CH_2), 2.73 (s, 2H, CH_2), 2.07 (d, *J* = 9.5 Hz, 2H, CH_2), 1.95 – 1.83 (m, 2H, CH_2) ppm; ^{13}C NMR (126 MHz, DMSO-d_6) *δ* 166.6, 166.3, 160.9, 158.6, 158.4, 158.1, 151.8, 151.2, 138.6, 134.0, 133.2, 131.6, 131.4, 130.8, 126.2, 125.6, 125.2, 123.5, 120.7, 118.4, 116.3, 116.1, 111.0, 105.9, 51.5, 51.0, 27.1, 20.3, 19.7, 19.4 ppm. HRMS: calcd. for $C_{31}H_{24}NO_6$: 506.1598, $[M+H]^+$ found: 506.1592.

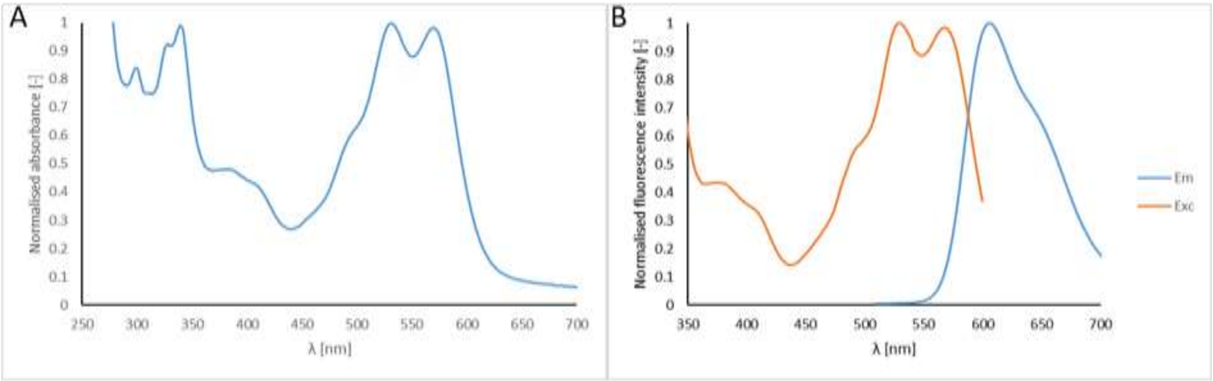

Figure S24. (A) Normalized absorption, (B) excitation (blue) and fluorescence emission spectra (orange) of **11i** in HEPES buffer (c= 5 µM, pH = 7.4).

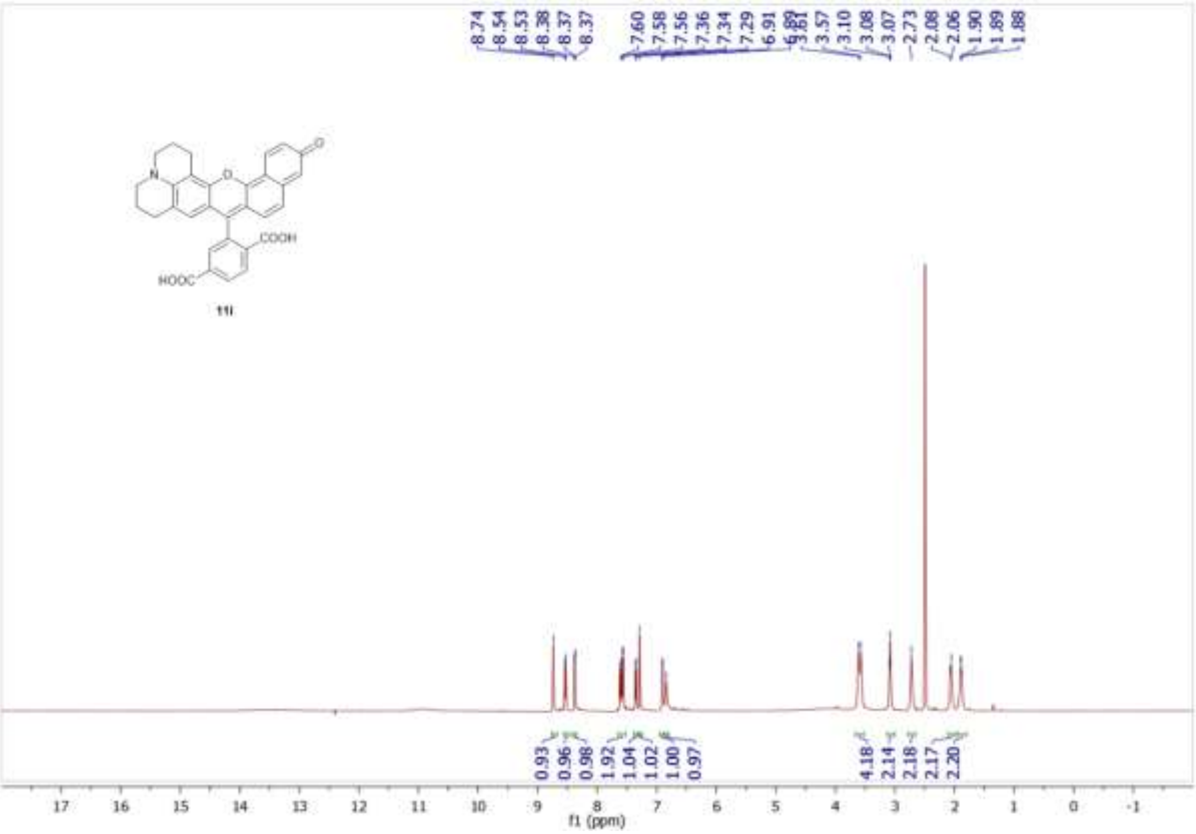

Figure S25. ^{1}H NMR spectrum of **11i** recorded at 500 MHz in DMSO-d_6.

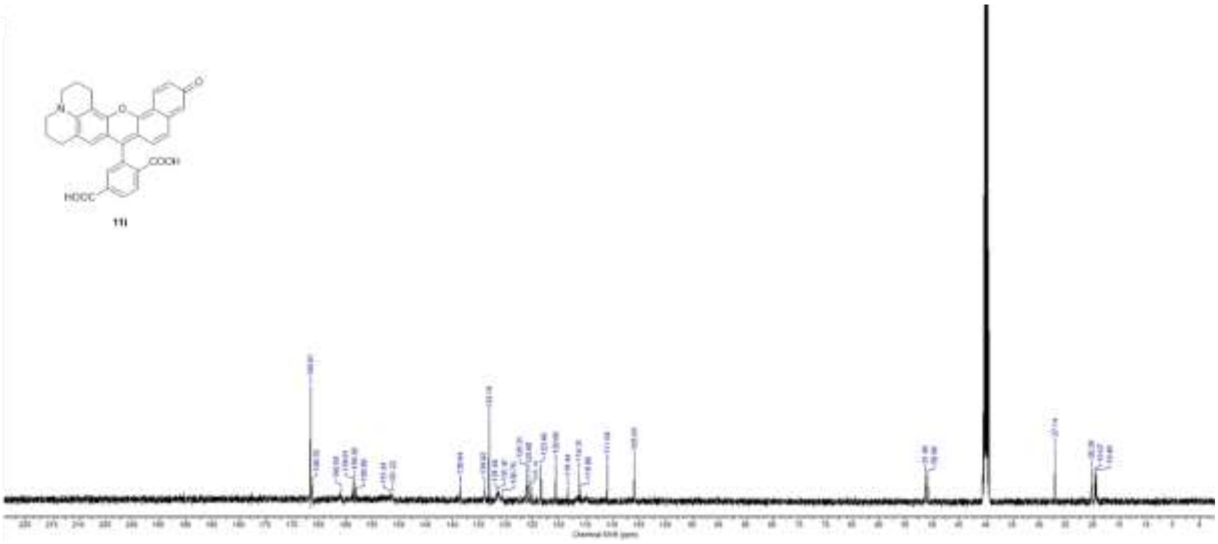

Figure S26. ^{13}C NMR spectrum of **11i** in DMSO-d_6 recorded at 126 MHz.

*4-(13-Oxo-2,3,6,7-tetrahydro-1H,5H,13H-benzo[7,8]chromeno[2,3-f]pyrido[3,2,1-ij]quinolin-9-yl)isophthalic acid (**11j**)*

^{1}H NMR (500 MHz, DMSO-d_6) δ 8.59 (d, *J* = 8.9 Hz, 1H, ArH), 8.32 (q, *J* = 8.2 Hz, 2H, 2×ArH), 7.95 (s, 1H, ArH), 7.89 (s, 1H, ArH), 7.61 (d, *J* = 8.8 Hz, 1H, ArH), 7.40 (dd, *J* = 9.1, 1.9 Hz, 1H, ArH), 7.29 (s, 1H, ArH), 6.89 (d, *J* = 8.7 Hz, 1H, ArH), 6.82 (d, *J* = 8.7 Hz, 1H, ArH), 3.65 – 3.52 (m, 4H, 2×CH_2), 3.20 – 3.10 (m, 2H, CH_2), 2.74 – 2.70 (m, 2H, CH_2), 2.13 – 2.02 (m, 2H, CH_2), 1.93 – 1.84 (m, 2H, CH_2) ppm; ^{13}C NMR (126 MHz, DMSO-d_6) *δ* 166.0, 160.2, 157.7, 150.9, 140.6, 138.0, 134.9, 130.9, 125.6, 125.1, 124.5, 123.1, 120.1, 115.97, 115.95, 110.5, 105.5, 50.8, 50.4, 26.7, 19.9, 19.3, 19.0 ppm. HRMS: calcd. for $C_{31}H_{24}NO_6$: 506.1598, $[M+H]^+$ found: 506.1607.

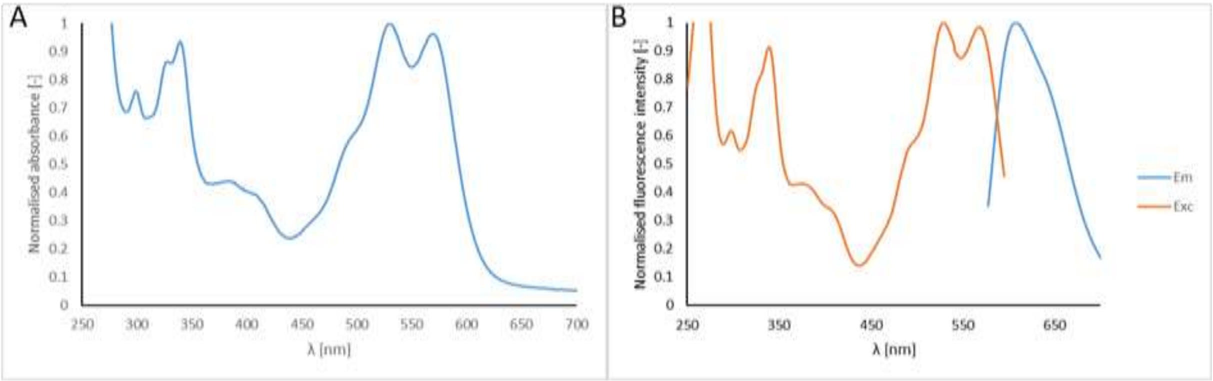

Figure S27. (A) Normalized absorption, (B) excitation (blue) and fluorescence emission spectra (orange) of **11j** in HEPES buffer (c= 5 μM, pH = 7.4).

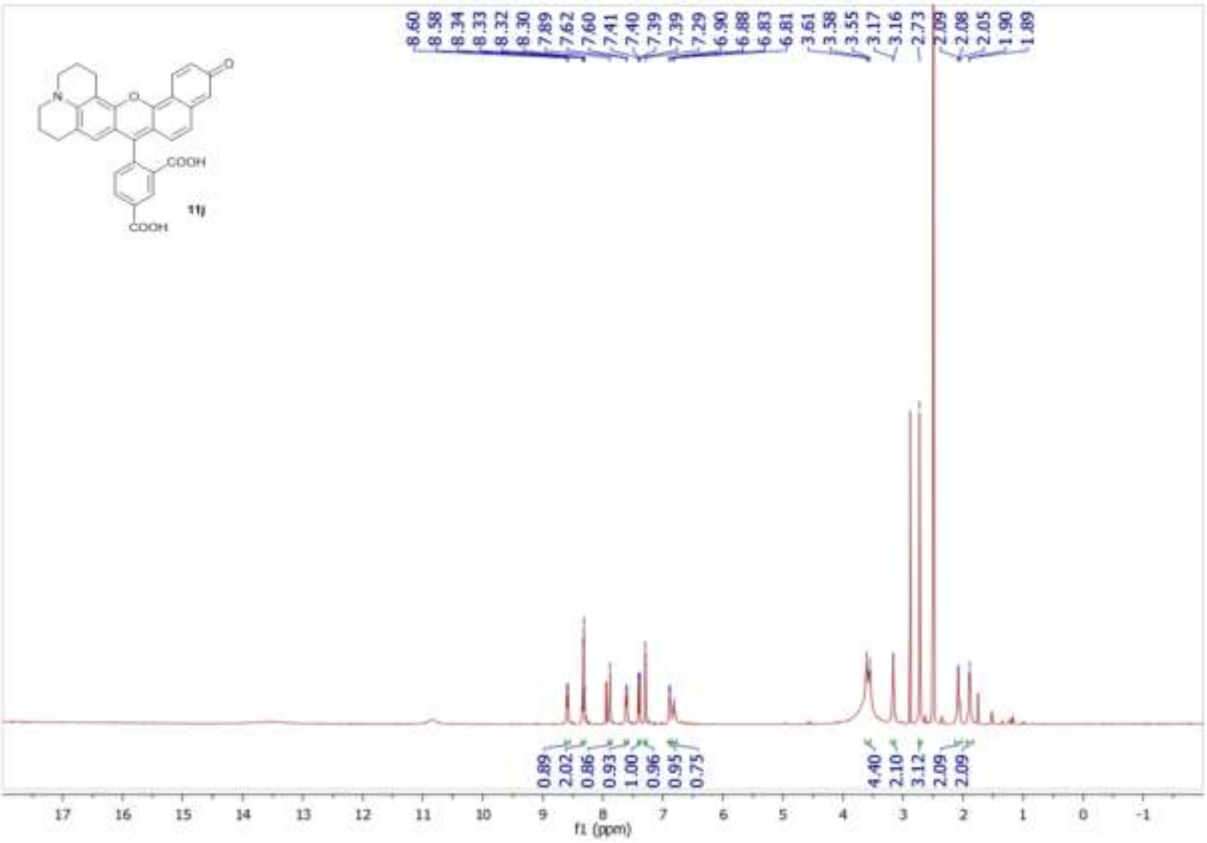

Figure S28. ^{1}H NMR spectrum of **11j** recorded at 500 MHz in DMSO-d_6.

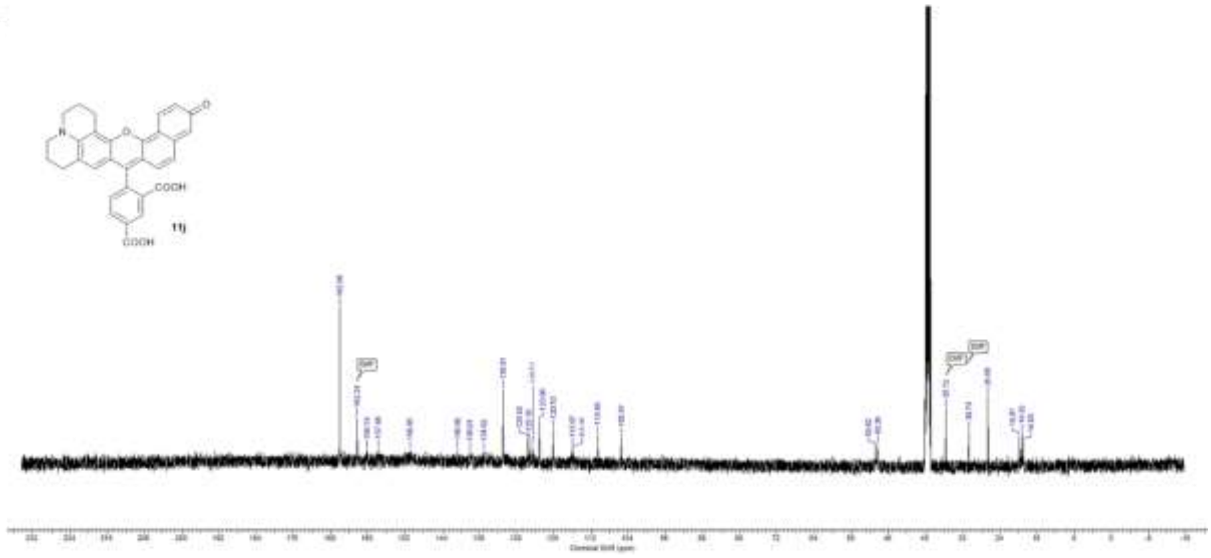

Figure S29. ^{13}C NMR spectrum of **11j** in DMSO-d_6 recorded at 126 MHz.

*2-(14-Hydroxy-2,3,6,7-tetrahydro-1H,5H,9H-benzo[5,6]chromeno[2,3-f]pyrido[3,2,1-ij]quinolin-9-yl)terephthalic acid (**11k**)*

^{1}H NMR (500 MHz, DMSO-d_6) δ 8.78 (d, J = 1.3 Hz, 1H, ArH), 8.36 – 8.28 (m, 2H, 2×ArH), 7.48 (t, J = 7.3 Hz, 2H, 2×ArH), 7.33 – 7.28 (m, 1H, ArH), 6.90 (d, J = 8.4 Hz, 2H, 2×ArH), 6.40 (s, 1H, ArH), 3.50 – 3.40 (m, 4H, 2×CH_2), 3.05 – 2.91 (m, 2H, CH_2), 2.61 (dd, J = 11.5, 5.4 Hz, 2H, CH_2), 2.00 (dq, J = 12.1, 6.0 Hz, 2H, CH_2), 1.88 – 1.78 (m, 2H, CH_2) ppm; ^{13}C NMR (126 MHz, DMSO-d_6) δ 166.6, 166.2, 158.5, 158.3, 150.0, 133.6, 132.7, 132.3, 131.3, 130.4, 130.2, 126.8, 125.9, 125.1, 125.0, 104.8, 100.8, 50.6, 50.1, 27.4, 20.7, 19.8, 19.7 ppm. HRMS: calcd. for $C_{31}H_{26}NO_6$: 506.1598, $[M+H]^+$ found: 506.1598.

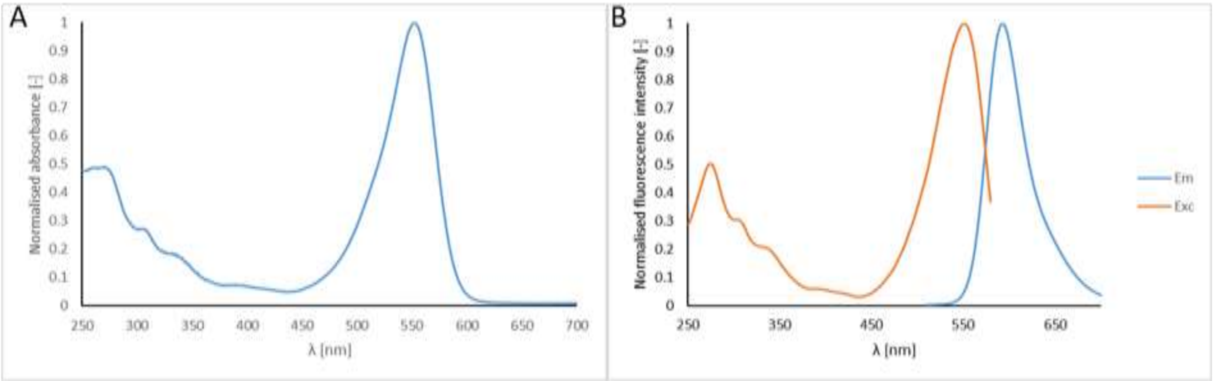

Figure S30. (A) Normalized absorption, (B) excitation (blue) and fluorescence emission spectra (orange) of **11k** in HEPES buffer (c= 5 μM, pH = 7.4).

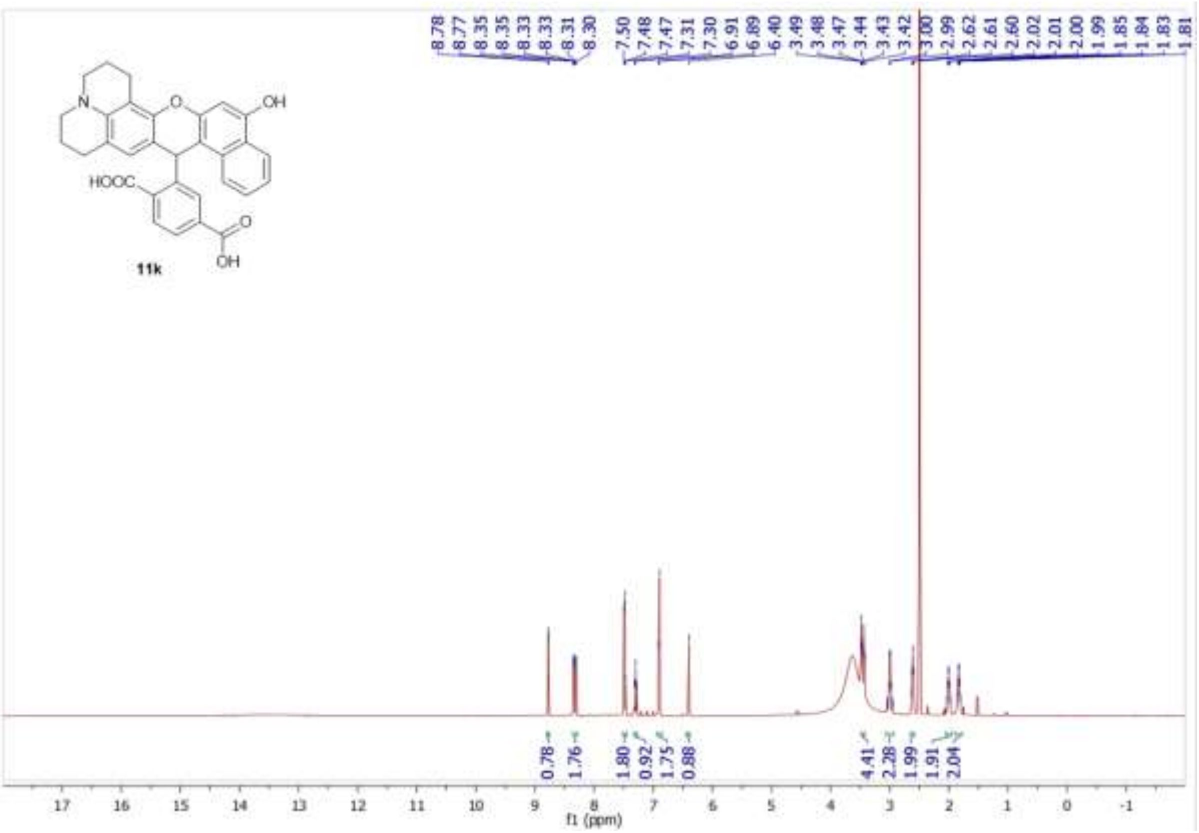

Figure S31. ^{1}H NMR spectrum of **11k** recorded at 500 MHz in DMSO-d_6.

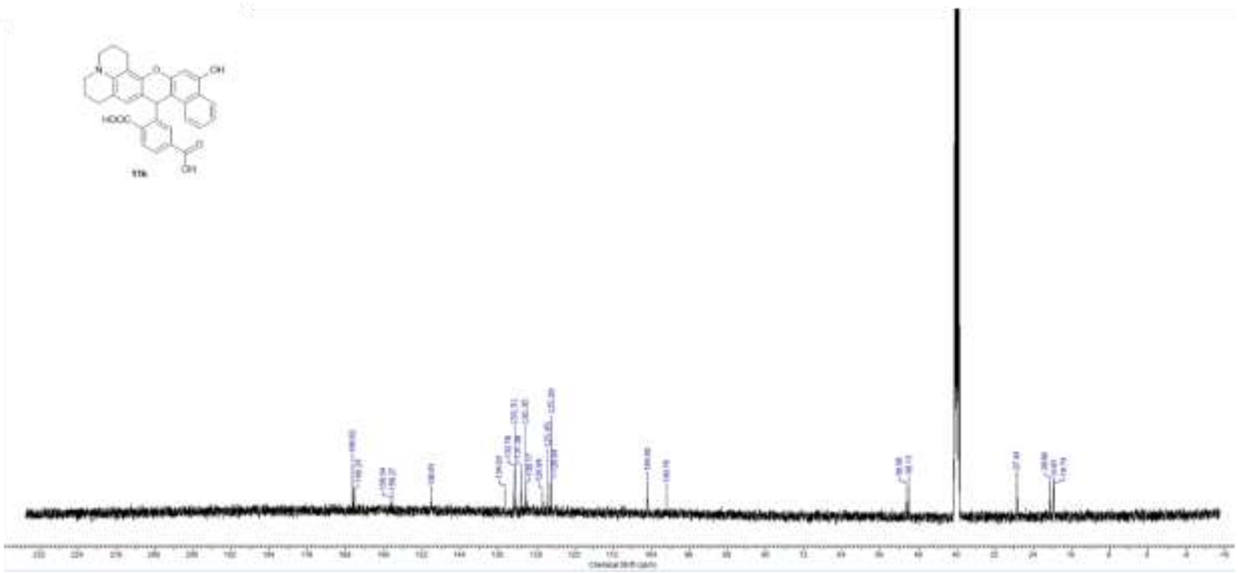

Figure S32. ^{13}C NMR spectrum of **11k** in DMSO-d_6 recorded at 126 MHz.

*4-(14-Hydroxy-2,3,6,7-tetrahydro-1H,5H,9H-benzo[5,6]chromeno[2,3-f]pyrido[3,2,1-ij]quinolin-9-yl)isophthalic acid (**11l**)*

^{1}H NMR (300 MHz, DMSO-d_6) δ 8.78 (d, J = 1.6 Hz, 1H, ArH), 8.39 – 8.26 (m, 2H, 2×ArH), 7.55 – 7.44 (m, 2H, 2×ArH), 7.37 – 7.27 (m, 1H, ArH), 7.02 (s, 1H, ArH), 6.92 (d, J = 8.6 Hz, 1H, ArH), 6.46 (s, 1H, ArH), 3.54 – 3.39 (m, 4H, 2×CH_2), 3.08 – 2.88 (m, 2H, CH_2), 2.62 (s, 2H, CH_2), 2.08 – 1.70 (m, 4H, 2×CH_2) ppm; ^{13}C NMR (75 MHz, DMSO-d_6) δ 166.6, 166.2, 159.7, 150.7, 150.4, 142.6, 134.6, 132.8, 132.4, 131.3, 131.1, 130.3, 127.0, 126.9, 125.8, 125.2, 125.0, 124.9, 113.8, 110.2, 104.7, 100.4, 50.7, 50.3, 27.4, 20.5, 19.7, 19.6 ppm. HRMS: calcd. for $C_{31}H_{26}NO_6$: 506.1598, $[M+H]^+$ found: 506.1593.

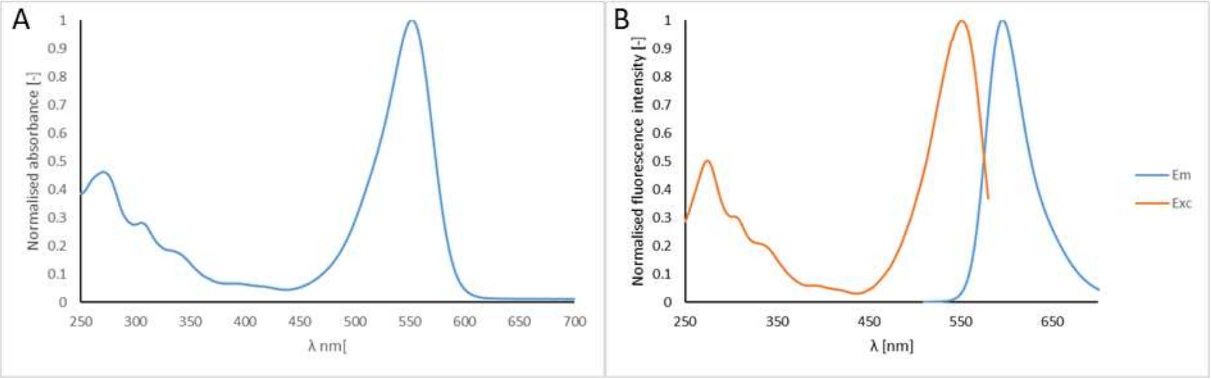

Figure S33. (A) Normalized absorption, (B) excitation (blue) and fluorescence emission spectra (orange) of **11l** in HEPES buffer (c= 5 µM, pH = 7.4).

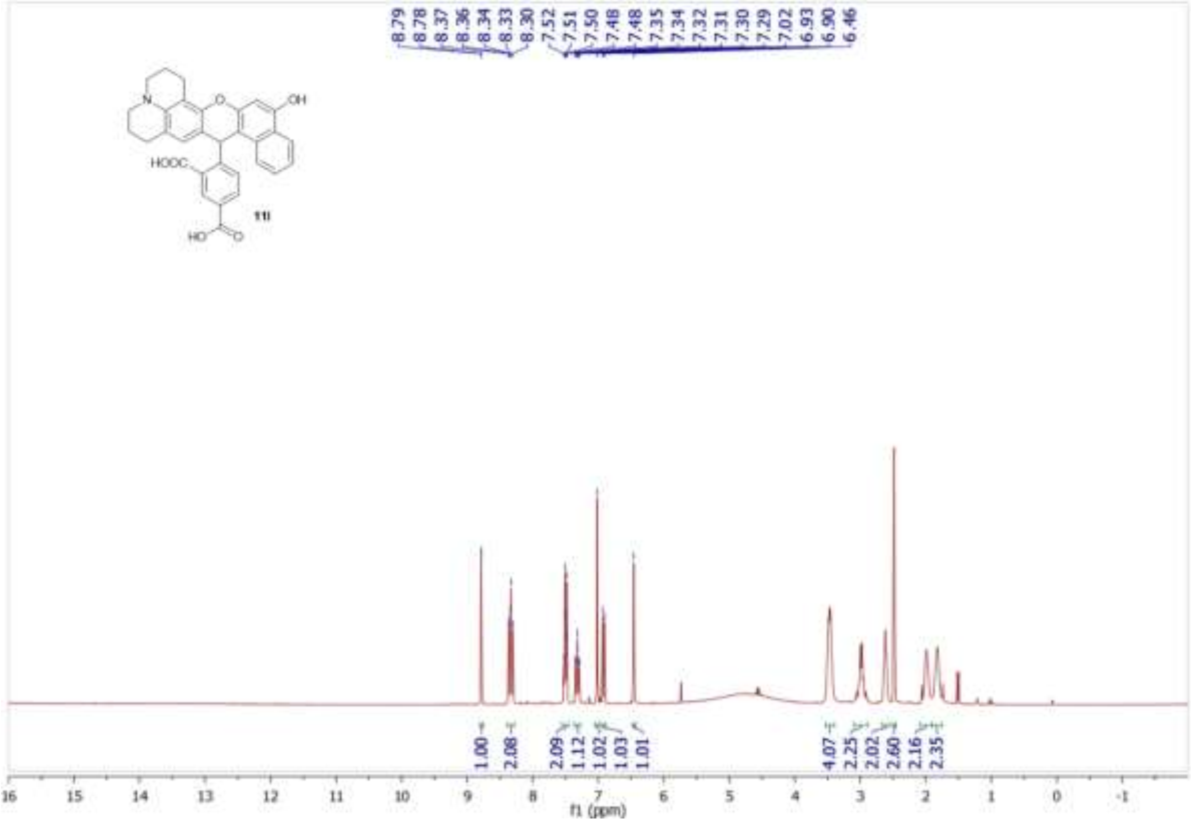

Figure S34. ^{1}H NMR spectrum of **11l** recorded at 300 MHz in DMSO-d_6.

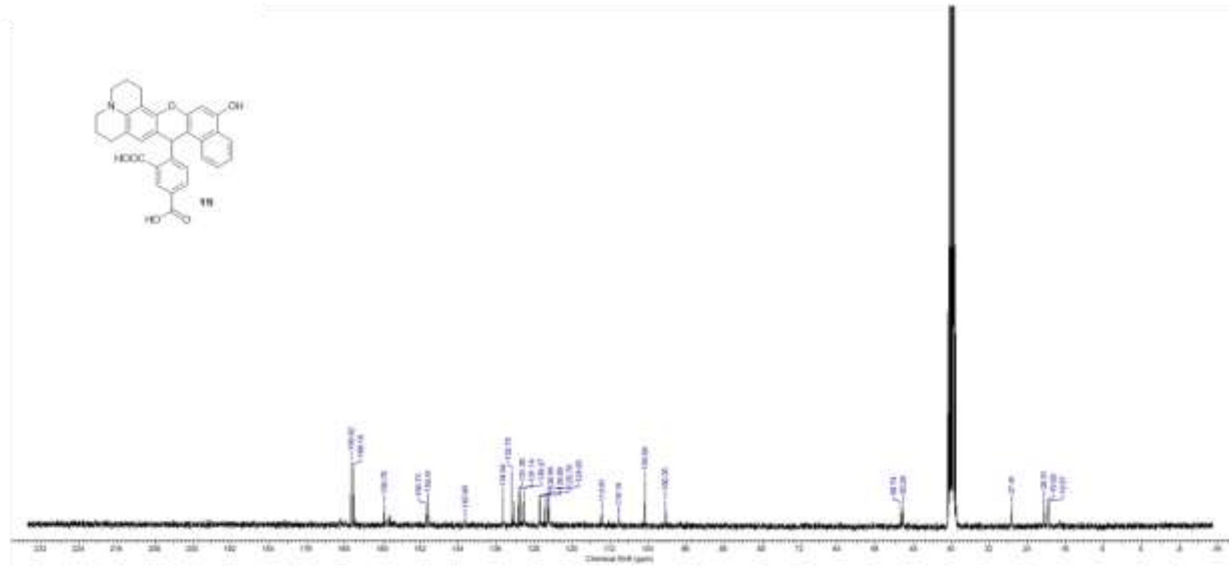

Figure S35. ^{13}C NMR spectrum of **11l** in DMSO-d_6 recorded at 75 MHz.

*2-(11-(2-Carboxyethyl)-12-oxo-2,3,6,7-tetrahydro-1H,5H,12H-chromeno[2,3-f]pyrido[3,2,1-ij]quinolin-9-yl)terephthalic acid (**11m**)*

^{1}H NMR (500 MHz, DMSO-d_6) δ 8.26 – 8.22 (m, 1H, ArH), 8.19 (d, *J* = 8.1 Hz, 1H, ArH), 7.70 (s, 1H, ArH), 6.87 (s, 1H, ArH), 6.67 (s, 1H, ArH), 6.39 (s, 1H, ArH), 3.41 – 3.27 (m, 4H, 2×CH_2), 2.94 – 2.86 (m, 2H, CH_2), 2.61 (tt, *J* = 10.9, 5.3 Hz, 2H, CH_2), 2.58 – 2.52 (m, 2H, CH_2), 2.32 (t, *J* = 7.7 Hz, 2H, CH_2), 2.01 – 1.92 (m, 2H, CH_2), 1.83 – 1.75 (m, 2H, CH_2) ppm; ^{13}C NMR (126 MHz, DMSO-d_6) *δ* 174.1, 167.3, 166.6, 158.5, 158.3, 156.0, 153.2, 150.1, 148.9, 136.0, 132.8, 131.2, 128.9, 128.2, 127.9, 125.5, 122.4, 111.8, 106.3, 102.3, 50.5, 50.0, 33.6, 27.1, 25.4, 20.9, 20.3, 20.2 ppm. HRMS: calcd. for $C_{30}H_{26}NO_8$: 528.1652, $[M+H]^+$ found: 528.1665.

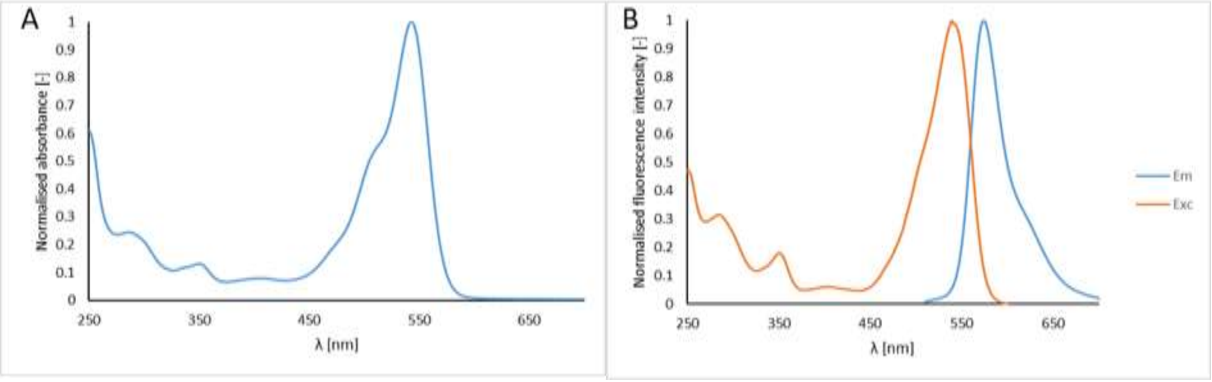

Figure S36. (A) Normalized absorption (B) excitation (orange) and fluorescence emission spectra (blue) (B) of **11m** in HEPES buffer (c= 5 μM, pH = 7.4).

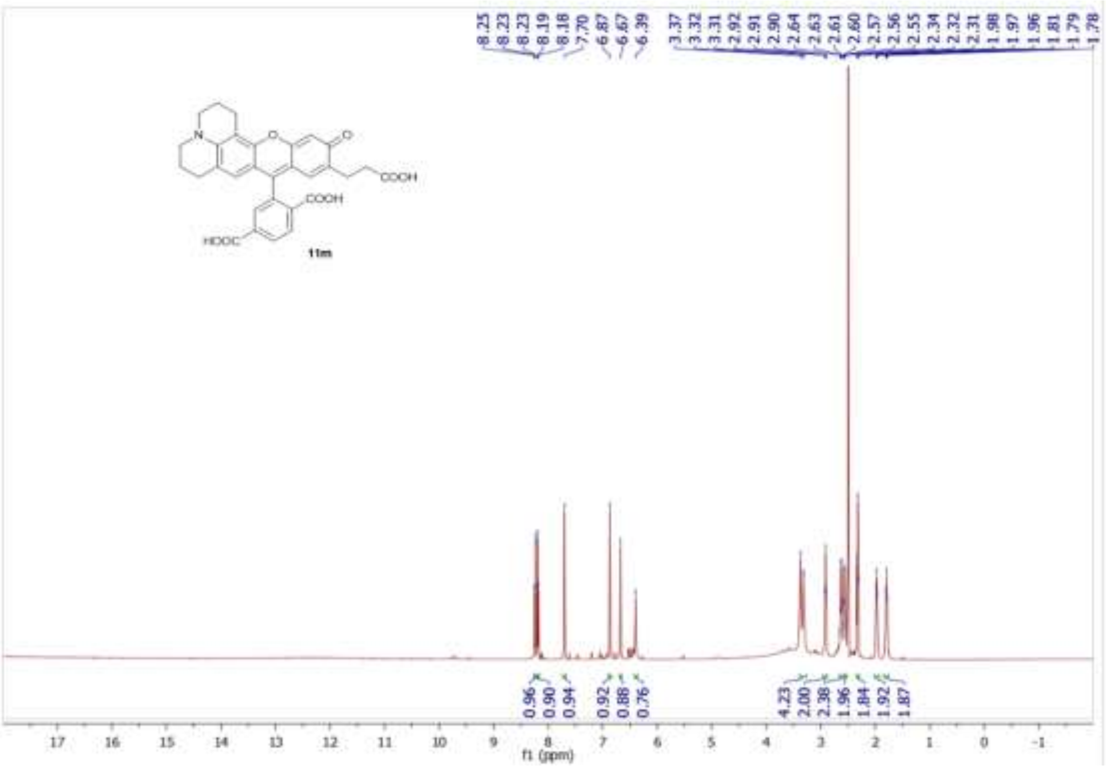

Figure S37. ^{1}H NMR spectrum of **11m** recorded at 500 MHz in DMSO-d_6.

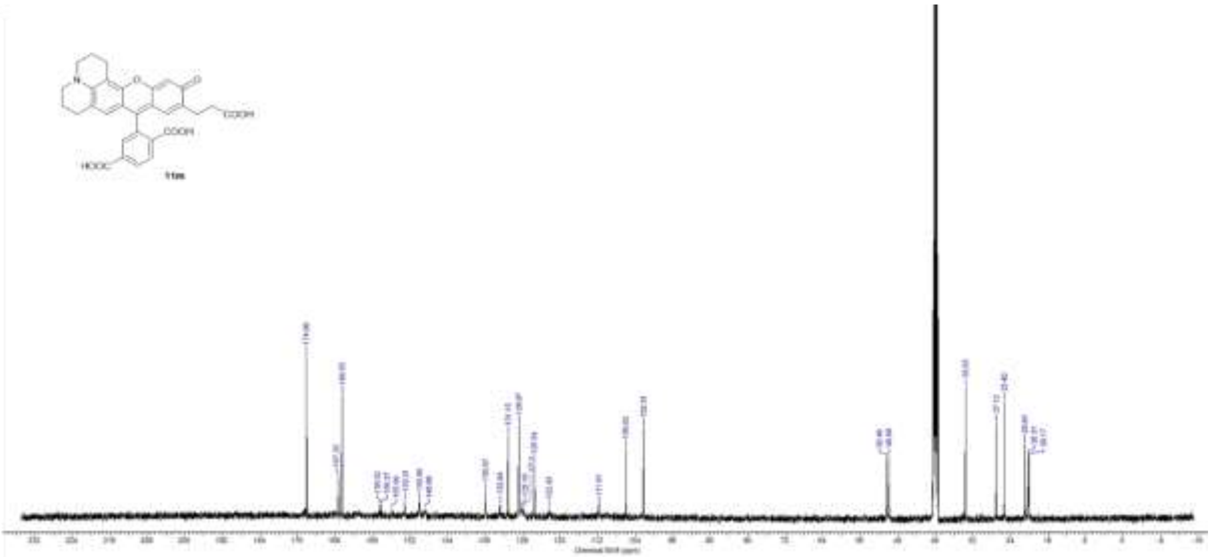

Figure S38. ^{13}C NMR spectrum of **11m** in DMSO-d_6 recorded at 126 MHz.

*4-(11-(2-Carboxyethyl)-12-oxo-2,3,6,7-tetrahydro-1H,5H,12H-chromeno[2,3-f]pyrido[3,2,1-ij]quinolin-9-yl)isophthalic acid (**11n**)*

^{1}H NMR (300 MHz, DMSO-d_6) δ 8.72 – 8.66 (m, 1H, ArH), 8.34 (d, *J* = 8.0 Hz, 1H, ArH), 7.51 (d, *J* = 7.9 Hz, 1H, ArH), 7.14 (s, 1H, ArH), 6.90 (s, 1H, ArH), 6.70 (s, 1H, ArH), 3.61 – 3.47 (m, 4H, 2×CH_2), 3.04 – 2.95 (m, 2H, CH_2), 2.76 – 2.62 (m, 4H, 2×CH_2), 2.35 (t, *J* = 17.0, 9.8 Hz, 2H, CH_2), 2.05 – 1.96 (m, 2H, CH_2), 1.91 – 1.80 (m, 2H, CH_2) ppm; ^{13}C NMR (75 MHz, DMSO-d_6) *δ* 174.0, 166.9, 166.3, 164.3, 158.7, 158.3, 154.2, 152.4, 151.9, 133.9, 133.0, 131.3, 130.6, 129.4, 128.9, 126.4, 126.1, 114.0, 113.6, 105.7, 102.1, 51.3, 50.8, 33.6, 31.1, 27.1, 25.3, 20.4, 19.8, 19.5 ppm. HRMS: calcd. for $C_{30}H_{26}NO_8$: 528.1652, $[M+H]^+$ found: 528.1658.

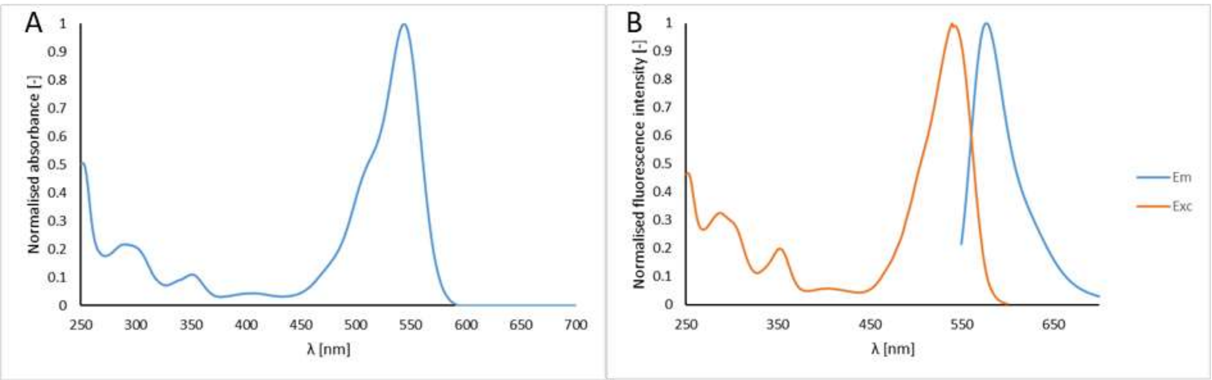

Figure S39. Normalized (A) Absorption, (B) excitation (orange) and fluorescence emission (blue) spectra of **11n** in HEPES buffer (c= 5 µM, pH = 7.4).

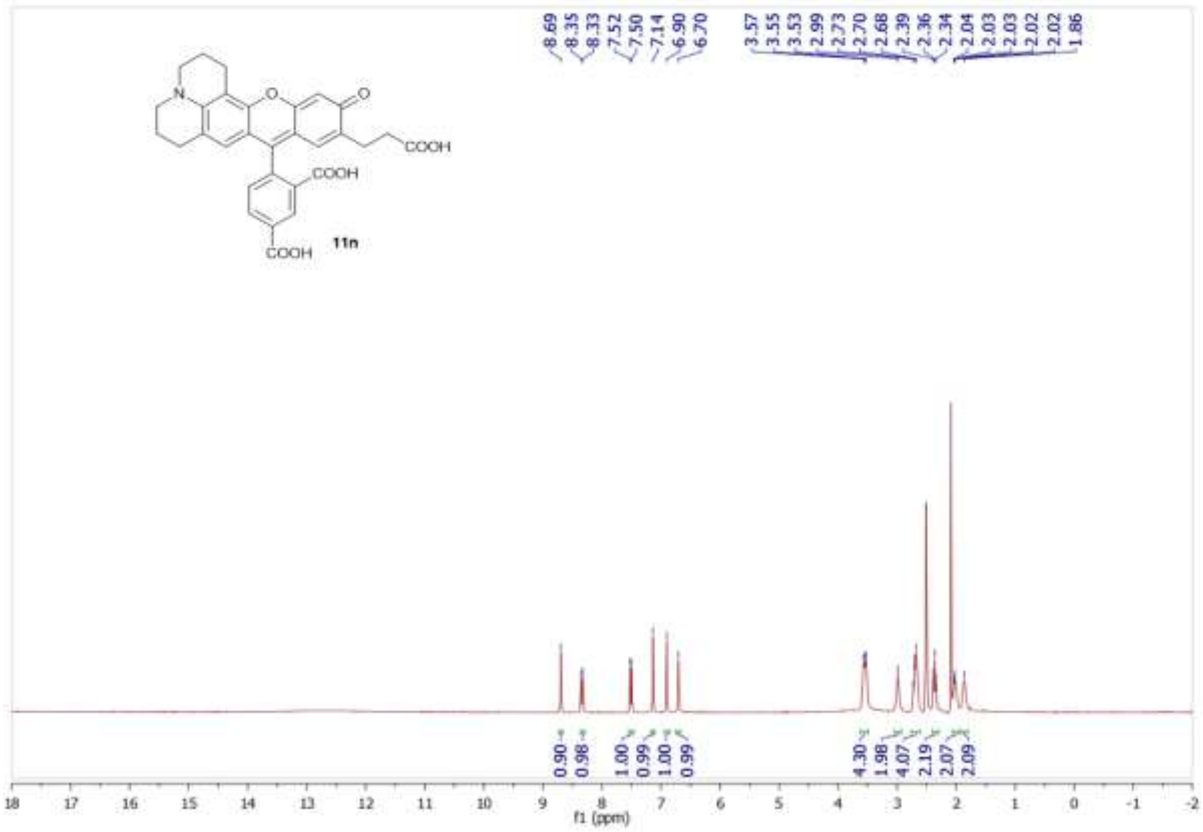

Figure S40. ^{1}H NMR spectrum of **11n** recorded at 300 MHz in DMSO-d_6.

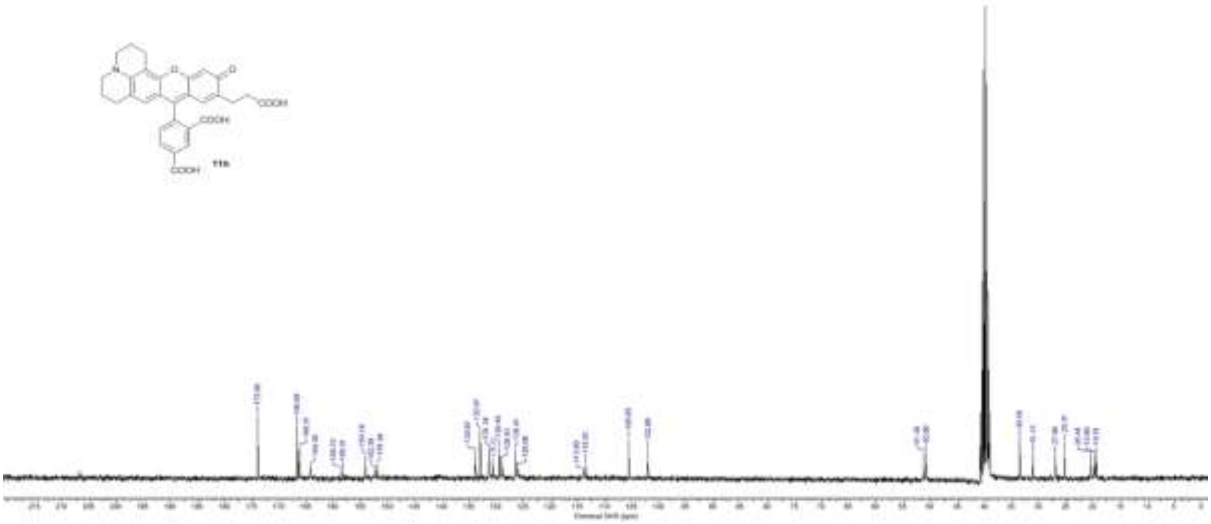

Figure S41. ^{13}C NMR spectrum of **11n** in DMSO-d_6 recorded at 75 MHz.

*4-((3-Azidopropyl)carbamoyl)-2-(11-ethyl-12-oxo-2,3,6,7-tetrahydro-1H,5H,12H-chromeno[2,3-f]pyrido[3,2,1-ij]quinolin-9-yl)benzoic acid (**17**)*

^{1}H NMR (500 MHz, DMSO-*d*$_6$) δ 8.94 (t, *J* = 5.5 Hz, 1H, CONH), 8.67 (s, 1H, ArH), 8.29 (d, *J* = 8.0 Hz, 1H, ArH), 7.53 (d, *J* = 7.9 Hz, 1H, ArH), 7.17 (s, 1H, ArH), 6.83 (s, 1H, ArH), 6.73 (s, 1H, ArH), 3.65 – 3.50 (m, 4H, 2×CH_2), 3.47 (t, *J* = 6.7 Hz, 2H, CH_2), 3.42 (dd, *J* = 12.5, 6.5 Hz, 2H, CH_2), 3.07 – 2.94 (m, 2H, CH_2), 2.71 – 2.65 (m, 2H, CH_2), 2.09 – 1.95 (m, 2H, CH_2), 1.92 – 1.80 (m, 4H, 2×CH_2), 1.00 (t, *J* = 7.4 Hz, 3H, CH_3) ppm; ^{13}C NMR (126 MHz, DMSO-*d*$_6$) *δ* 165.3, 164.4, 155.6, 154.2, 152.2, 136.4, 131.9, 130.5, 129.5, 128.2, 126.5, 113.9, 105.6, 102.0, 51.4, 50.9, 49.0, 37.4, 28.8, 27.1, 22.9, 20.4, 19.8, 19.5, 14.2 ppm. HRMS: calcd. for $C_{32}H_{32}N_5O_5$: 566.2403, $[M+H]^+$ found: 566.2389.

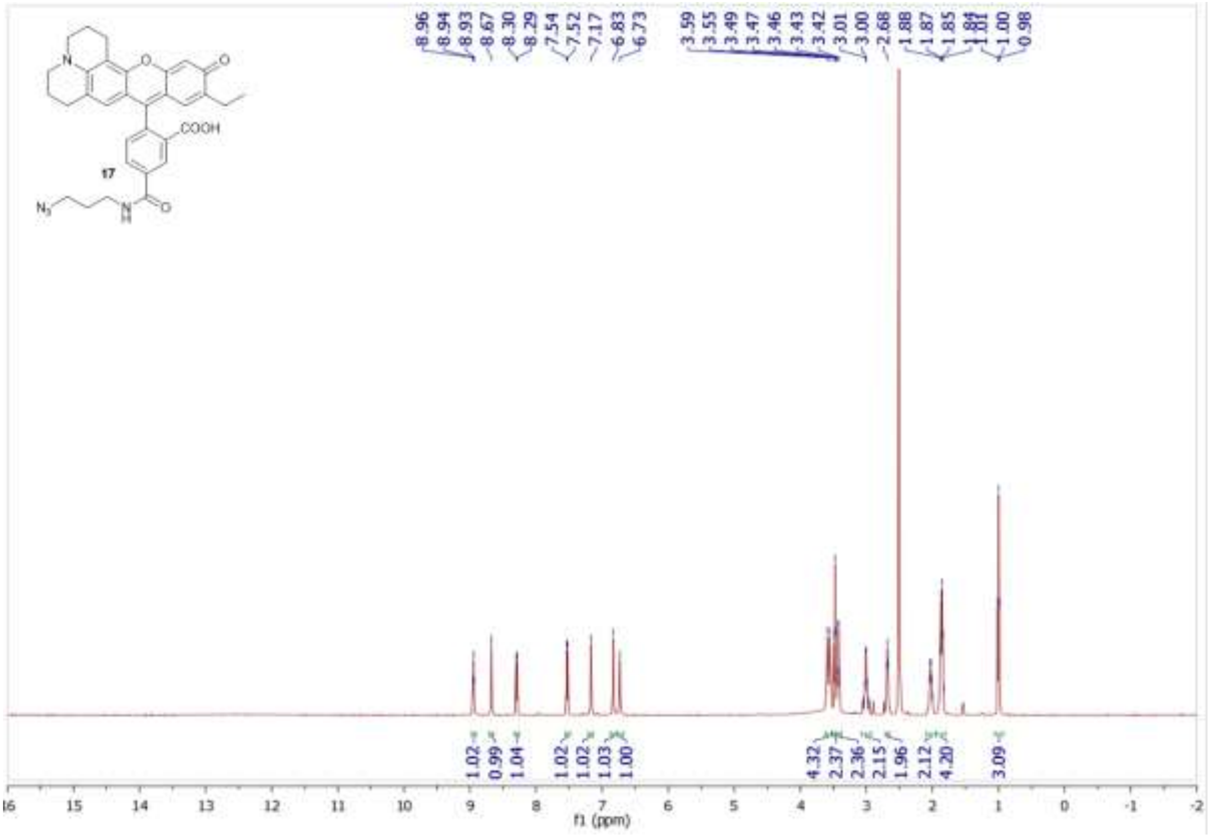

Figure S42. ^{1}H NMR spectrum of **17** in DMSO-d6 recorded at 500 MHz.

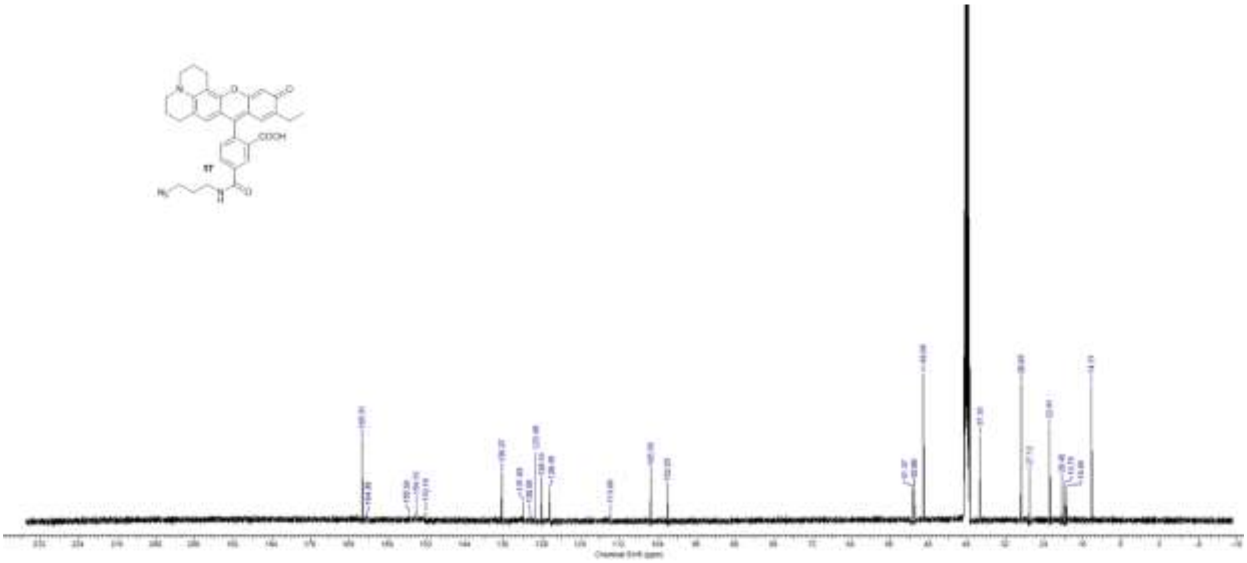

Figure S43. ^{13}C NMR spectrum of **17** in DMSO-d_6 recorded at 126 MHz.

Structure determination of **11f** rhodol was carried out using 2D NMR measurements.

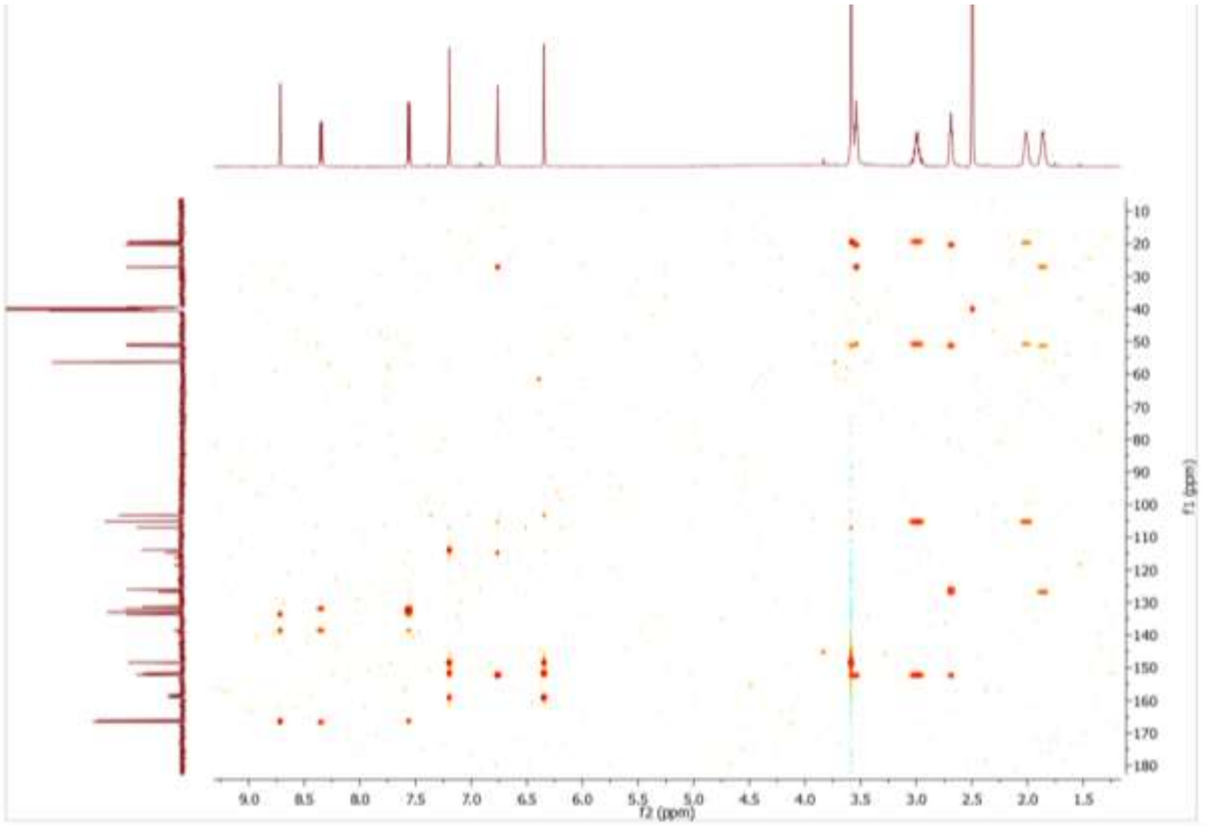

Figure S44. HMBCAD spectrum of **11f** recorded in DMSO-d_6.

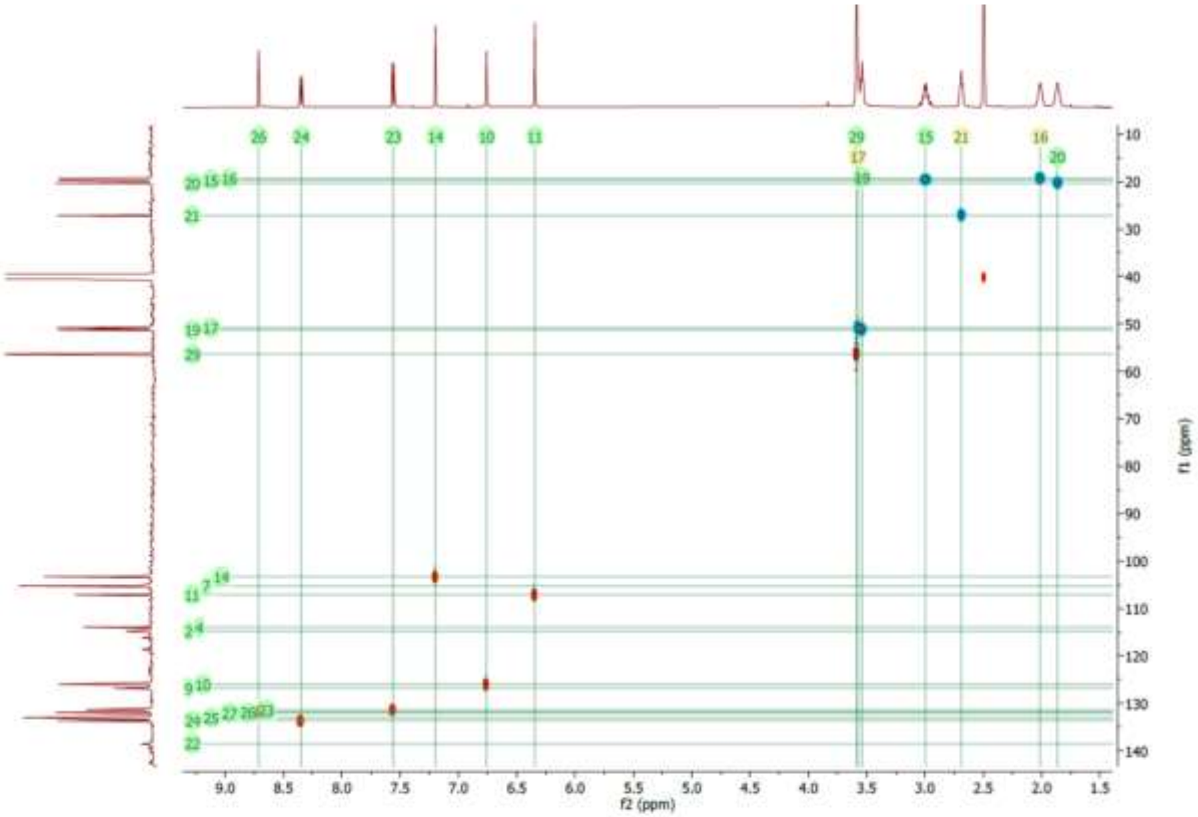

Figure S45. HSQCAD spectrum of **11f** recorded in DMSO-d_6.

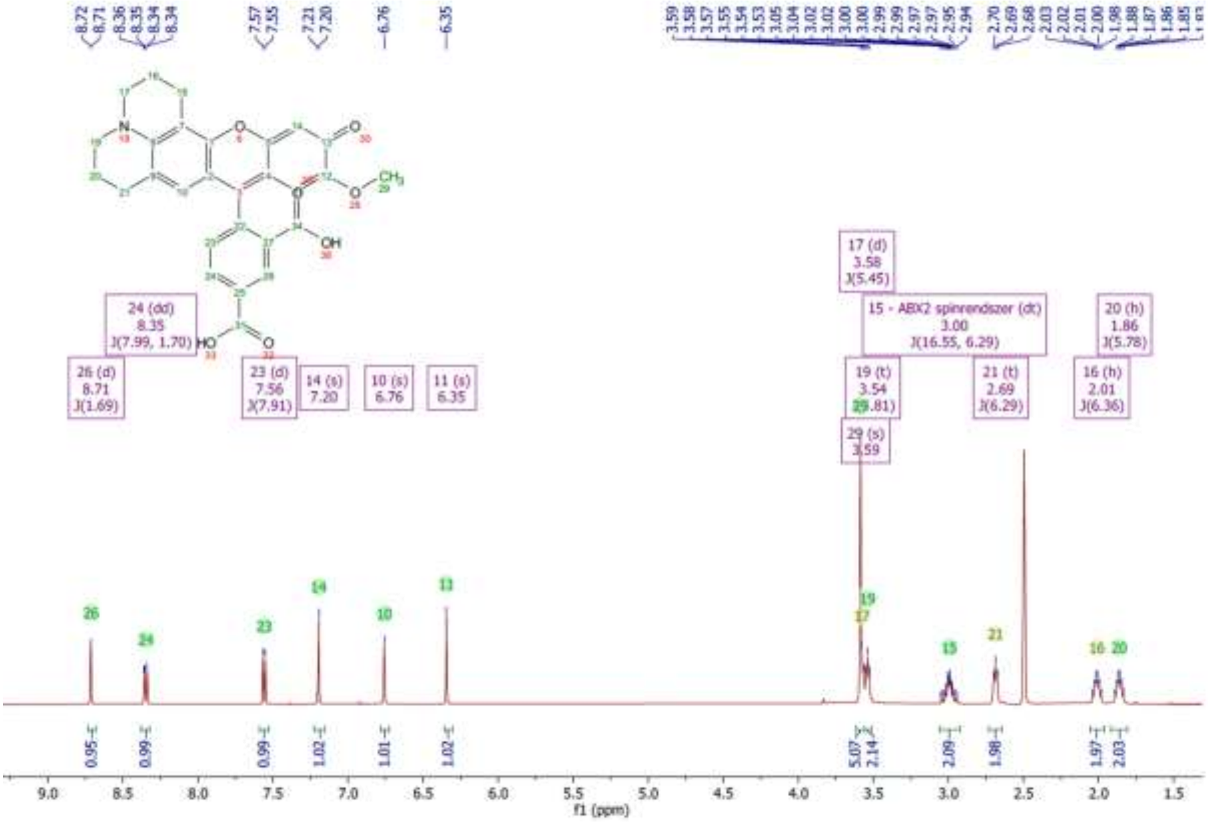

Figure S46. ^{1}H NMR spectrum in DMSO-d_6 recorded at 500 MHz with signal assignment of **11f**.

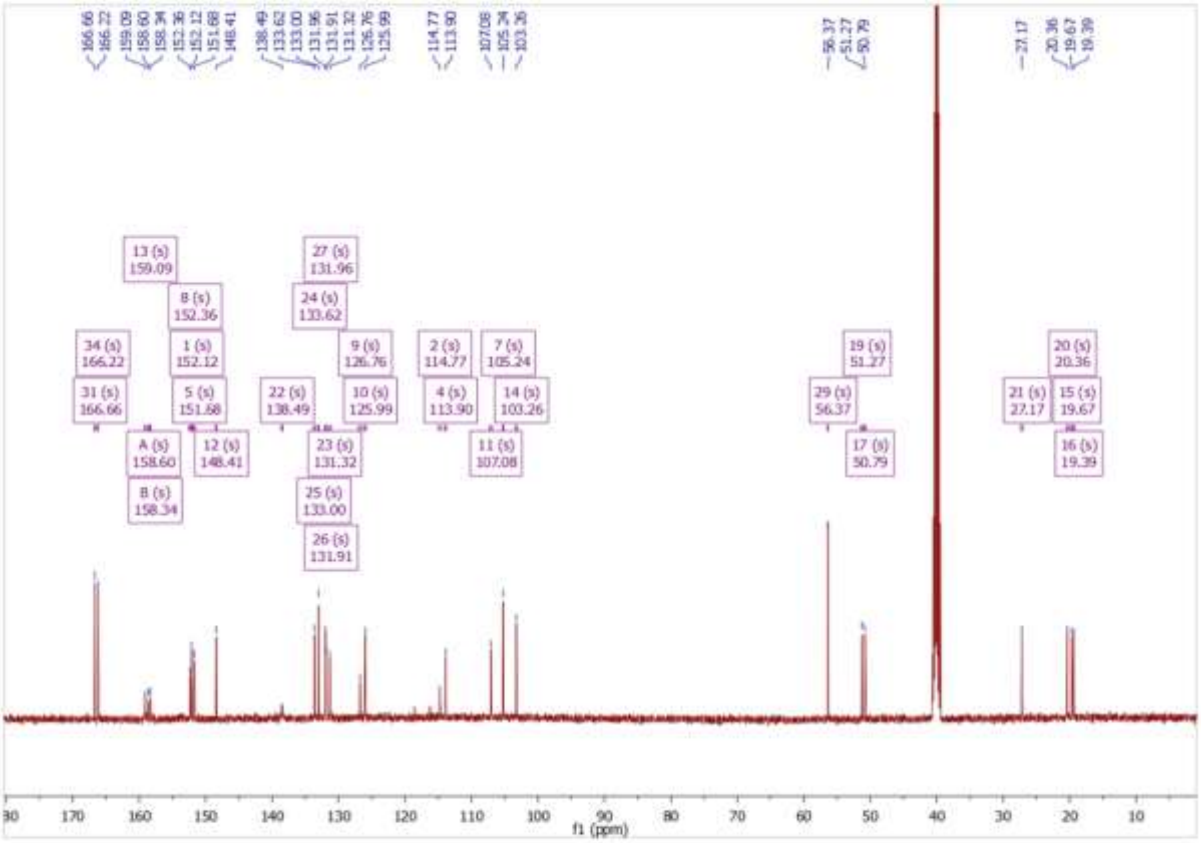

Figure S47. ^{13}C NMR spectrum of **11f** in DMSO-d_6 recorded at 126 MHz with signal assignment.

Two-photon cross section of the relevant rhodol derivatives

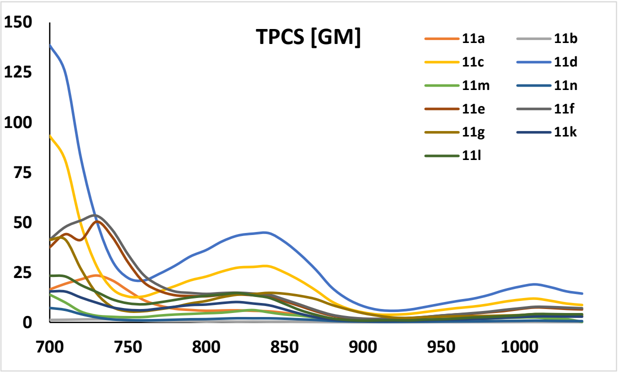

Figure S48. Two-photon cross sections (TPCS) of compounds in HEPES buffer (c= 5 μM), normalized to 1.0 M concentrations. GM: Goeppert-Mayer unit.

The pH dependent of absorption and emission spectra of **11d**

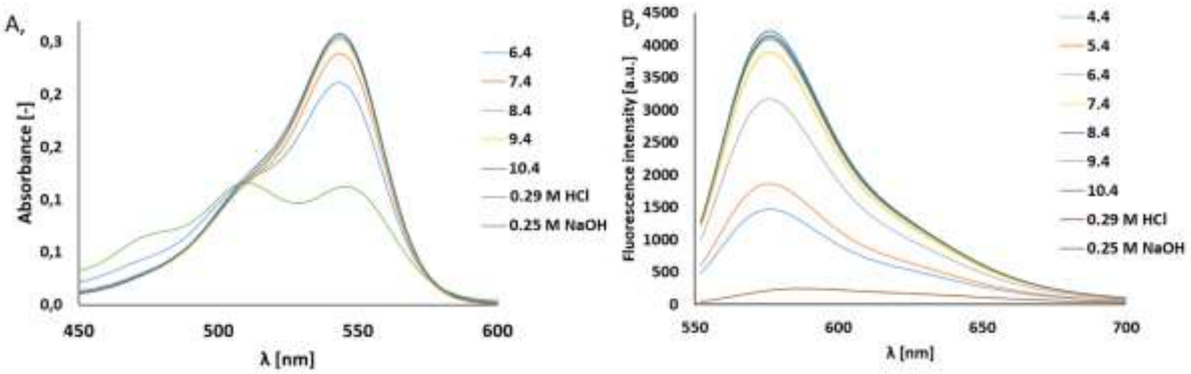

Figure S49. (A) pH-dependent UV-Vis absorption spectra of **11d** in HEPES buffer (c= 5 μM), normalized to 1.0 M concentrations. The numbers placed at the spectra denote the wavelength of absorption maxima. (B) Fluorescence emission spectra of **11d** in HEPES buffer (c= 5 μM) at different pH values. Excitation was performed at or near the wavelength position of absorption maxima in the spectra recorded at the same pH values.

Photostability measurement of **11d** dye

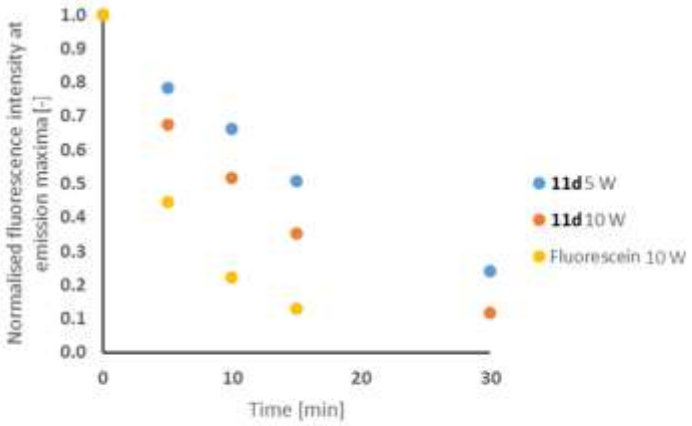

Figure S50. Time dependent of emission intensity at emission maxima of **11d** (exc. 520 nm), 5-TAMRA (exc. 520 nm), fluoresceine isotiocyanate (exc. 450 nm) and Rhodamine B (exc. 520 nm) with continuous excitation using 5 W or 10 W performances (c = 5 μM, pH = 7.4).

Solvent dependence of **11d** rhodol derivative

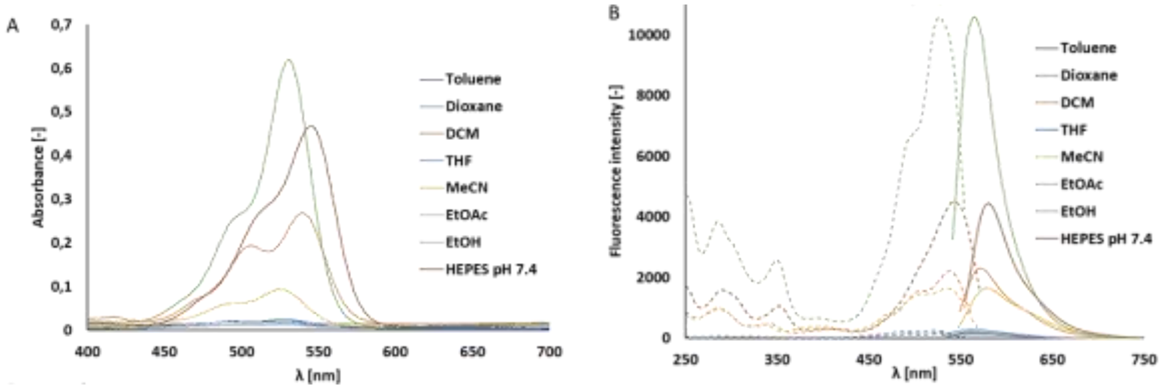

Figure S51. Molar UV-Vis absorption spectra, (B) excitation (dotted line) and fluorescence emission (solid line) spectra, normalised to c = 5.0 μM concentration of **11d** in different solvents. Excitations were executed at or near the wavelength position of absorption maxima.

Aggregation studies of **11d** in aqueous media

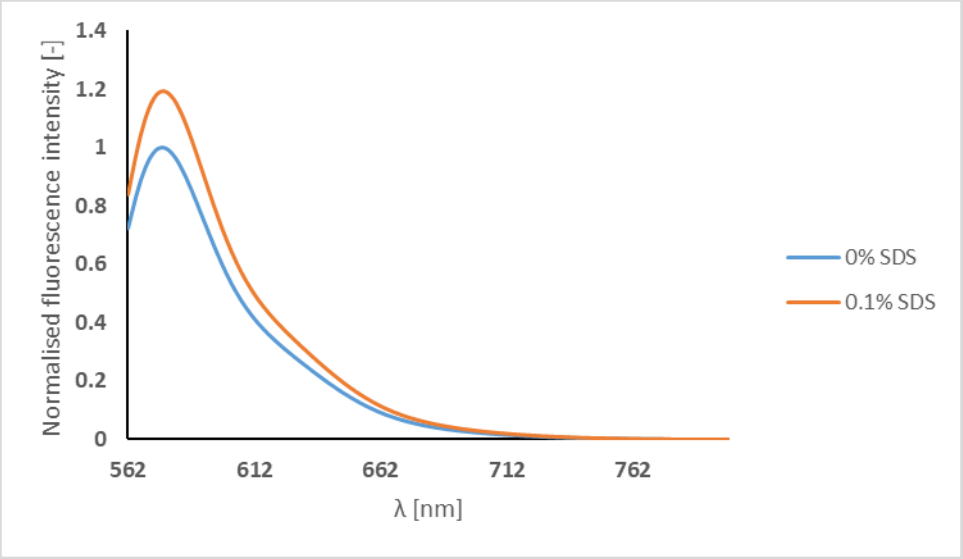

Figure S52. Emission spectra of **11d (**c = 5 μM**)** without (orange) and in the presence of 0.1% SDS (blue line) in HEPES (pH = 7.4) buffer to examine aggregation

pH stability studies of **11d**

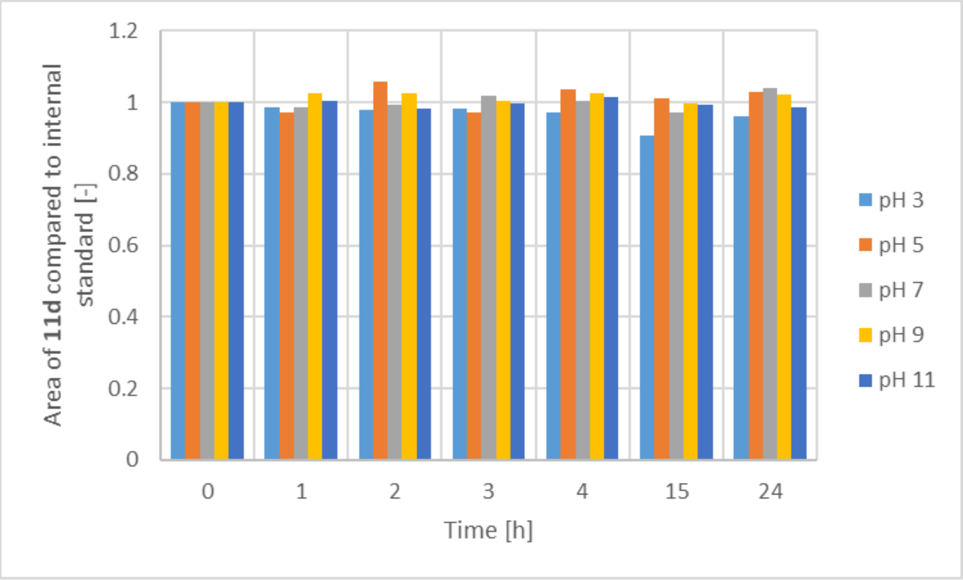

Figure S53. Change of the area of **11d** (c = 5 µM) in different pH HEPES buffers over time followed by HPLC using indoprofen as an internal standard.

SDS-PAGE results of **13** conjugate

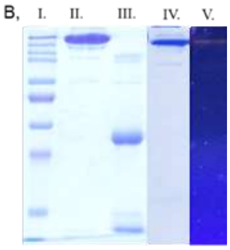

Figure S54. SDS-PAGE in non-reducing gel: I. molecular weight protein marker, II. native trastuzumab, III. Reduced trastuzumab, IV. **13** , V. **13** under 366 nm light.

Flow cytometry measurements of cells without or treated with **13** antibody conjugate.

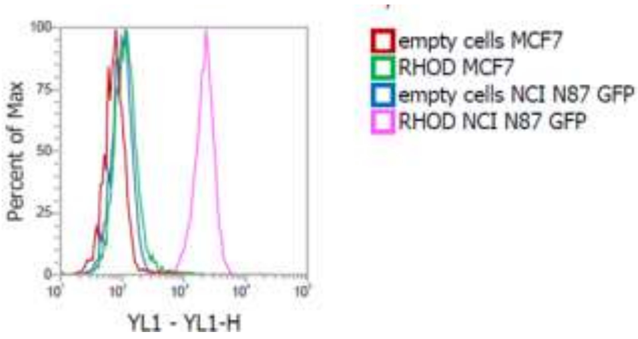

Figure S55. Results of the flow cytometry measurement. Red line: untreated MCF-7 cell line (Her2-); green line: trastuzumab-rhodol conjugate (13) treated MCF-7 cell line (Her2-); blue line: untreated NCI-N87-GFP cell line (Her2+); pink line: trastuzumab-rhodol conjugate (13) treated NCI-N87-GFP cell line (Her2+)

Absorbance measurement of rhodol-trastuzumab conjugate (**13**).

Table S2. Average calculated FAR value from measured absorbance values

Experiment	1	2	3	Average
$A_{280\ nm}$	0.024	0.03	0.166	
$A_{552\ nm}$	0.013	0.019	0.095	
$c_{\mathbf{11d}}$	4.91	7.18	35.9	
c_T	1.38	1.58	9.29	
FAR	3.56	4.54	3.86	3.99

Two-photon measurements of cells without or treated with **13** antibody conjugate.

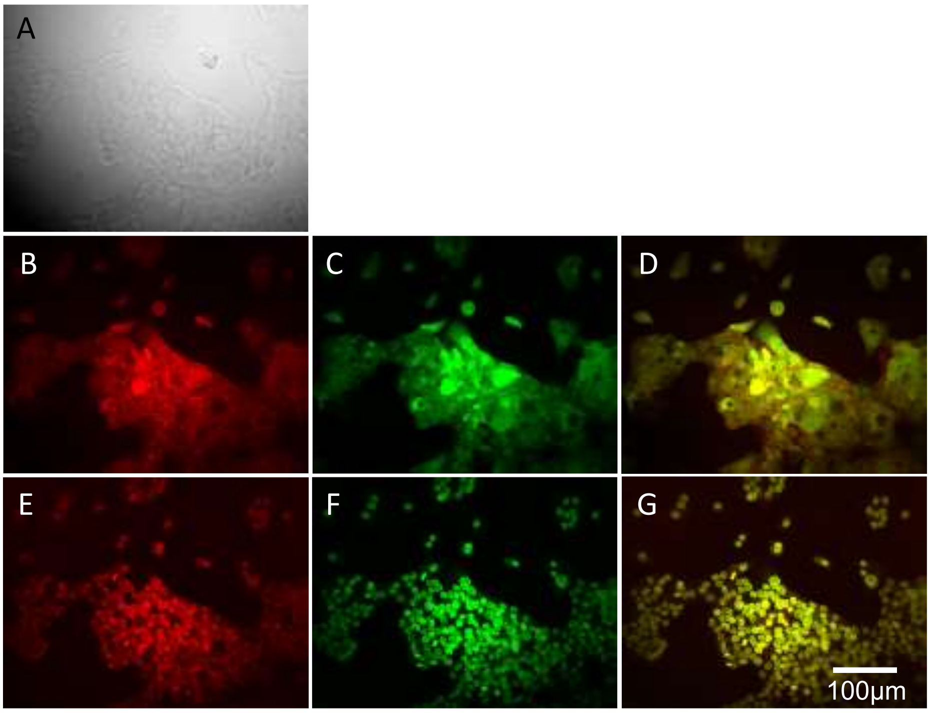

Figure S56. Nci-rhodol positive cell (Her2+) could be visualized in wavelength specific manner by two-photon microscopy. A: CCD camera image of Her2+ cells treated by trastuzumab antibody. B-G: Maximum intensity projection of a Nci-rhodol (Her2+) cells from 9 plane in 840 (B-D) and 750 nm (E-G) wavelength of two-photon laser. Red trastuzumab antibody labelled rhodol conjugated cell surface labelling, green GFP expressed cells. D and G are the merged images of the red and green channels.

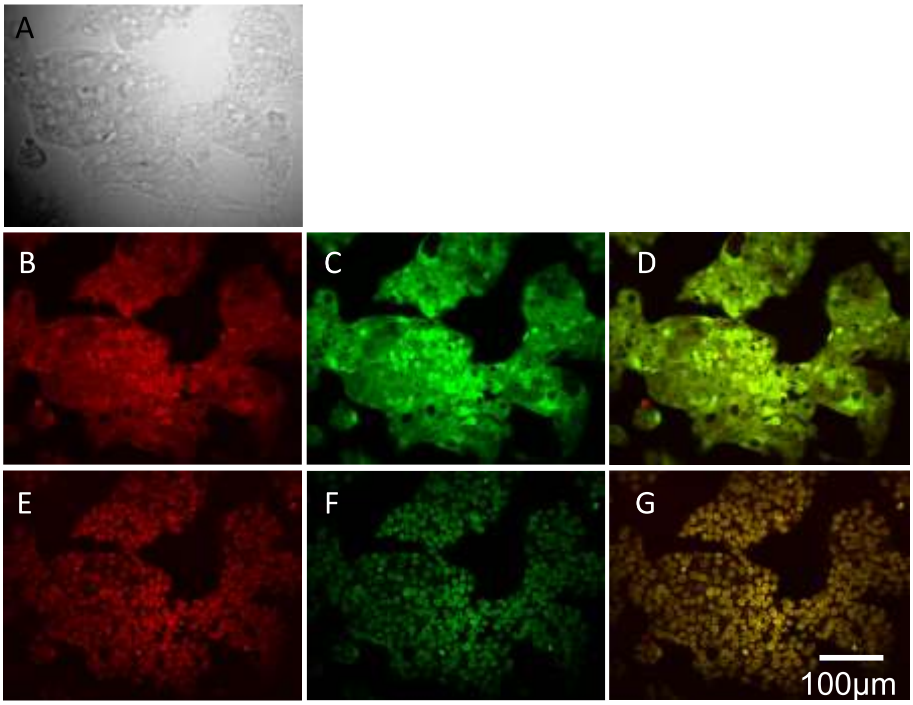

Figure S57. Nci-rhodol negaitve cell (Her2+) not show trastuzumab antibody conjugated cell surface labelling by two-photon imaging. A: CCD camera image of Her2+ cells treated by trastuzumab antibody labelled rhodol. B-G: Maximum intensity projection of a Nci-rhodol (Her2+) cells from 9 plane in 840 (B-D) and 750 nm (E-G) wavelength of two-photon laser. Red antibody conjugated cell surface labelling, green GFP expressed cells. D and G are the merged images of the red and green channels.

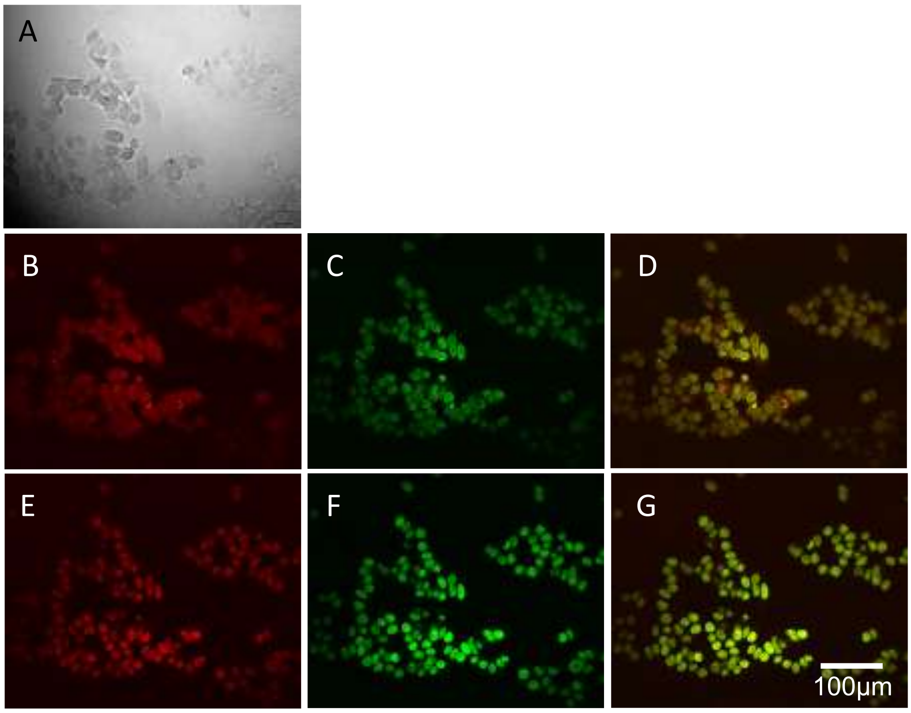

Figure S58. Mcf7-rhodol positive cell (Her2+) not show xx antibody conjugated cell surface labelling by two-photon imaging. A: CCD camera image of Her2+ cells treated by trastuzumab antibody labelled rhodol. B-G: Maximum intensity projection of a Nci-rhodol (Her2+) cells from 9 plane in 840 (B-D) and 750 nm (E-G) wavelength of two-photon laser. Red trastuzumab antibody labelled rhodol conjugated cell surface labelling, green GFP expressed cells. D and G are the merged images of the red and green channels.

Computed enthalpy and Gibbs free energy changes, the coordinates of the optimized structures.

Table S3a. Computed enthalpy (ΔH) and Gibbs free energy changes (ΔG) in the equilibria of Form-II relative to From-IIc and Form-III relative to From-IIIb in kJ mol^{-1} at B3LYP/6-31G(d,p)//PCM(water) level of theory for compound **16a-p**.

Structure		Equilibrium in Form II						Equilibrium in Form II			
		IIa		IIb		IIc		IIIa		IIIb	
		ΔH	ΔG	ΔH	ΔG	ΔH	ΔG	ΔH	ΔG	ΔH	ΔG
**16p**	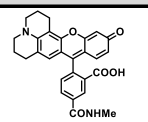	26.9	22.6	13.5	3.9	0.0	0.0	22.7	31.4	0.0	0.0
**16o**	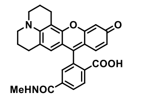	26.6	22.1	13.2	3.2	0.0	0.0	22.4	31.0	0.0	0.0
**16h**	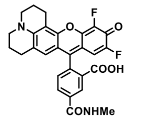	–*	–*	1.7	-7.9	0.0	0.0	13.6	21.5	0.0	0.0
**16g**	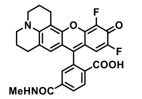	–*	–*	1.5	-7.1	0.0	0.0	13.4	21.2	0.0	0.0
**16d**	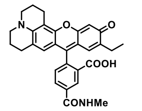	24.2	16.1	7.3	-2.5	0.0	0.0	27.4	35.6	0.0	0.0
**16c**	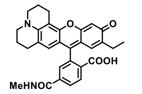	24.0	16.1	6.9	-3.6	0.0	0.0	27.1	35.2	0.0	0.0
**16n**	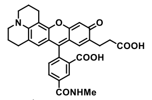	23.2	17.4	8.5	0.1	0.0	0.0	25.3	37.1	0.0	0.0
**16m**	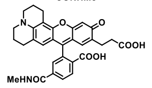	23.2	17.4	8.5	0.1	0.0	0.0	24.8	36.5	0.0	0.0
**16b**	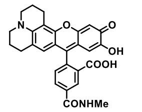	23.1	17.3	-18.0	-27.1	0.0	0.0	23.8	32.0	0.0	0.0
**16a**	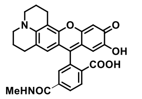	22.7	16.7	-18.4	-23.4	0.0	0.0	23.1	31.4	0.0	0.0
**16f**	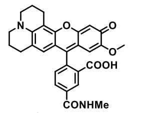	22.0	14.2	10.0	1.3	0.0	0.0	31.6	39.7	0.0	0.0
**16e**	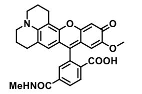	22.2	15.1	9.9	0.3	0.0	0.0	31.1	39.3	0.0	0.0
**16j**	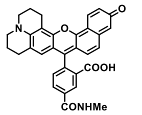	24.5	18.9	-6.1	-14.4	0.0	0.0	24.9	32.0	0.0	0.0
**16i**	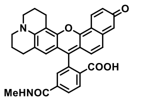	24.0	17.2	-6.3	-13.5	0.0	0.0	24.2	31.2	0.0	0.0
**16l**	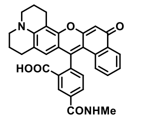	26.0	18.1	38.0	26.8	0.0	0.0	4.6	12.2	0.0	0.0
**16k**	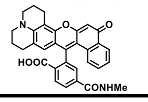	25.5	19.2	37.4	25.7	0.0	0.0	4.2	11.6	0.0	0.0

*not a minimum

Table S3b. Computed enthalpy (Δ*H*) and Gibbs free energy changes (Δ*G*) for the protonation-deprotonation equilibria of Form-I→ Form-IIb as well as Form-I→ Form-IIb in kJ mol^{-1} at B3LYP/6-31G(d,p)//PCM(water) level of theory for compound**16a-p**.

			Protonation eq.			
			**I → IIb**		**I →IIIb**	
	**R¹**	**R²**	Δ*H*	Δ*G*	Δ*H*	Δ*G*
**16p**	H	H	-40.1	-31	-22.2	-19.6
**16o**			-40.1	-31	-22.2	-19.6
**16h**	F	F	-49.7	-42.9	-46.1	-45.1
**16g**			-48.8	-39.3	-44.9	-41.8
**16d**	Et	H	-11.9	-9.3	0.9	-4.8
**16c**			-11.4	-8.7	1.6	-4
**16n**	EtCOO⁻	H	-9.0	-4.9	7.9	7.3
**16m**			-9.0	-4.9	7.9	7.3
**16b**	OH	H	-35.1	-27.3	-47.8	-43.8
**16a**			-11.4	-7.5	-23.9	-23
**16f**	OMe	H	-33.5	-23.5	-17.3	-13.9
**16e**			-11.1	-7.8	5.6	3.5
**16j**	napht1		-13.2	-10.7	-15.8	-19.3
**16i**			-12.8	-10.6	-15.2	-19.9
**16l**	napht2		-13.1	-11	26.8	22.6
**16k**			-12.5	-8.3	27.2	25.1

Consideration of the enthalpy and Gibbs free energy of proton

The correct and accurate consideration of the enthalpy (**Δ*H***) and Gibbs free energy (**Δ*G***) of a proton in a protonation deprotonation process in condensed media is a challenging topic. The surrounding and solvating hydrogen bonds is essential to get reliable values, however, waters form an unlimited HB network. To model the process still accurate, we computed a 4-member water cluster, where the deprotonated (Process A) and protonated (Process B) water molecule is placed in a HB system (**Figure S58**). In order to give a model for the neutral pH, the average values of the processes A and B are considered during the calculations.

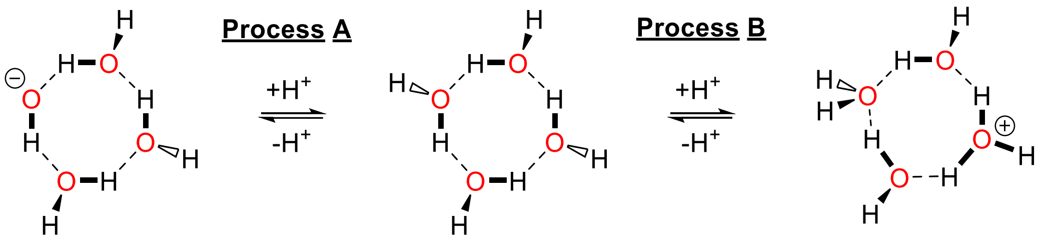

4 water - H$^+$ | **4 water** | **4 waters + H$^+$**

Δ*H*	Δ*G*
-1295.7	-1299.4

Δ*H*	Δ*G*
-1130.6	-1126.5

The average values of the two processes A and B.

Δ*H*	Δ*G*
**-1213.16**	**-1212.97**

Figure S59. The 4-membered waters clusters used for the proton solvation and its computed values in kJ mol^{-1}.

Tables containing the computed row data

Table S4. Computed energies (*E*), given in Hartree at B3LYP/6-31G(d,p) basis set with the consideration of PCM solvent method using the parameter set of water for **small molecules and 16**.

N	original	Type	Filename for the xyz tables	E	ZPE	U	H	G	S
water	–		000b_4_H2O-H+_b3lyp631dp_PCMw.log	-305.24666307	-305.163395	-305.155128	-305.154184	-305.195640	87.252
water	–		000a_4_H2O_b3lyp631dp_PCMw.log	-305.75734393	-305.658185	-305.649107	-305.648163	-305.691027	90.214
water	–		000b_4_H2O_+H+_b3lyp631dp_PCMw.log	-306.19930872	-306.088808	-306.080142	-306.079198	-306.120511	86.951
**1**	–	**I**	001aaa_TAMRA_B3LYP631dp_PCMw.log	-1412.76222108	-1412.351882	-1412.323806	-1412.322862	-1412.412776	189.242
**1**	–	**IIa**	002aaa_TAMRA-H+carb_B3LYP631dp_PCMw.log	-1412.30524021	-1411.907619	-1411.881275	-1411.880331	-1411.965367	178.973
**1**	–	**IIb**	002aba_TAMRA-H+carb_B3LYP631dp_PCMw.log	-1412.30525599	-1411.907360	-1411.880268	-1411.879324	-1411.966300	183.057
**1**	–	**IIc**	003aaa_TAMRA-H+_oH_B3LYP631dp_PCMw.log	-1412.29885072	-1411.901890	-1411.873936	-1411.872992	-1411.963409	190.300
**1**	–	**III**	004aaa_TAMRA-H+_oH_-H+carb_B3LYP631dp_PCMw.log	-1411.82171916	-1411.438015	-1411.410316	-1411.409372	-1411.498435	187.448
**16p**	–	**I**	011aaa_Rh_Julolidin_H_B3LYP631dp_PCMw.log	-1567.63314646	-1567.149332	-1567.119589	-1567.118644	-1567.212413	197.353
**16p**	–	**IIa**	012aaa_Rh_Julolidin_H_-H+_carb_B3LYP631dp_PCMw.log	-1567.16202817	-1566.691588	-1566.662100	-1566.661156	-1566.753174	193.670
**16p**	–	**IIb**	012baa_Rh_Julolidin_H_-H+_carb_B3LYP631dp_PCMw.log	-1567.17220551	-1566.701095	-1566.672356	-1566.671412	-1566.761803	190.244
**16p**	–	**IIc**	013aaa_Rh_Julolidin_H_-H+_oh_B3LYP631dp_PCMw.log	-1567.16706796	-1566.696822	-1566.667199	-1566.666255	-1566.760332	198.002
**16p**	–	**IIIa**	014aaa_Rh_Julolidin_H-H+carb_-H+oh_B3LYP631dp_PCMw.log	-1566.68937203	-1566.232409	-1566.203038	-1566.202093	-1566.295016	195.572
**16p**	–	**IIIb**	014bba_Rh_Julolidin_H-H+carb_B3LYP631dp_PCMw_ring.log	-1566.68044867	-1566.222889	-1566.194382	-1566.193438	-1566.283028	188.558
**16b**	**11b**	**I**	021aaa_Rh_Julolidin_OH_B3LYP631dp_PCMw.log	-1642.85592724	-1642.368171	-1642.337136	-1642.336191	-1642.432103	201.862
**16b**	**11b**	**I**	021aba_Rh_Julolidin_OH_B3LYP631dp_PCMw_f.log	-1642.85494394	-1642.367328	-1642.336256	-1642.335312	-1642.431519	202.485
**16b**	**11b**	**IIa**	022aaa_Rh_Julolidin_OH-H+_carb_B3LYP631dp_PCMw.log	-1642.38430643	-1641.910108	-1641.879182	-1641.878238	-1641.973496	200.486
**16b**	**11b**	**IIb**	022baa_Rh_Julolidin_OH-H+_carb_B3LYP631dp_PCMw_ring.log	-1642.39312620	-1641.918140	-1641.888007	-1641.887063	-1641.980074	195.758
**16b**	**11b**	**IIc**	023aaa_Rh_Julolidin_OH-H+oh_B3LYP631dp_PCMw.log	-1642.40023589	-1641.925434	-1641.894858	-1641.893914	-1641.990393	203.057
**16b**	**11b**	**IIIa**	024aaa_Rh_Julolidin_OH-2H_B3LYP631dp_PCMw.log	-1641.92222129	-1641.460654	-1641.430355	-1641.429411	-1641.523928	198.928
**16b**	**11b**	**IIIb**	024bba_Rh_Julolidin_OH-H+carb_B3LYP631dp_PCMw_ring.log	-1641.91285990	-1641.450689	-1641.421271	-1641.420327	-1641.511746	192.407

Table S4cont.

N	original	Type	Filename	E	ZPE	U	H	G	S
16f	11f	I	031aaa_Rh_Julolidin_OMe_B3LYP631dp_PCMw.log	-1682.16058273	-1681.644278	-1681.611826	-1681.610881	-1681.710534	209.737
16f	11f	IIa	032aaa_Rh_Julolidin_OMe+H+carb_B3LYP631dp_PCMw.log	-1681.68882704	-1681.185973	-1681.153686	-1681.152742	-1681.251645	208.158
16f	11f	IIb	032baa_Rh_Julolidin_OMe+H+carb_B3LYP631dp_PCMw_ring.log	-1681.69729548	-1681.193476	-1681.162079	-1681.161135	-1681.257050	201.869
16f	11f	IIc	033aaa_Rh_Julolidin_OMe+H+oh_B3LYP631dp_PCMw.log	-1681.69348027	-1681.190426	-1681.158249	-1681.157305	-1681.256543	208.864
16f	11f	IIIa	034aaa_Rh_Julolidin_OMe-2H+_B3LYP631dp_PCMw.log	-1681.21511383	-1680.725421	-1680.693413	-1680.692468	-1680.790966	207.306
16f	11f	IIIb	034bba_Rh_Julolidin_OMe-H+carb_B3LYP631dp_PCMw_ring.log	-1681.20267168	-1680.712556	-1680.681381	-1680.680437	-1680.775837	200.787
16h	11h	I	041aaa_Rh_Julolidin_F2_B3LYP631dp_PCMw.log	-1766.07648500	-1765.609232	-1765.577625	-1765.576680	-1765.673743	204.285
16h	11h	IIa	042aaa_Rh_Julolidin_F2-H+carb_B3LYP631dp_PCMw.log	-1765.61927378	-1765.164755	-1765.134048	-1765.133104	-1765.227673	199.037
16h	11h	IIb	042baa_Rh_Julolidin_F2-H+carb_B3LYP631dp_PCMw_ring.log	-1765.61927381	-1765.164755	-1765.134048	-1765.133104	-1765.227667	199.025
16h	11h	IIc	043aaa_Rh_Julolidin_F2-H+oh_B3LYP631dp_PCMw.log	-1765.61861448	-1765.164940	-1765.133386	-1765.132442	-1765.230689	206.779
16h	11h	IIIa	044aaa_Rh_Julolidin_F2-2H+_B3LYP631dp_PCMw.log	-1765.14188573	-1764.701499	-1764.670184	-1764.669240	-1764.766061	203.776
16h	11h	IIIb	044bba_Rh_Julolidin_F2-H+carb_B3LYP631dp_PCMw_ring.log	-1765.13657897	-1764.695492	-1764.665015	-1764.664071	-1764.757863	197.402
16d	11d	I	051aaa_Rh_Julolidin_Et_B3LYP631dp_PCMw.log	-1646.26890783	-1645.728844	-1645.696060	-1645.695116	-1645.795666	211.626
16d	11d	I	051aba_Rh_Julolidin_Et_B3LYP631dp_PCMw.log	-1646.27032432	-1645.730243	-1645.697495	-1645.696551	-1645.797021	211.458
16d	11d	IIa	052aba_Rh_Julolidin_Et-H+carb_B3LYP631dp_PCMw.log	-1645.79866333	-1645.272103	-1645.239509	-1645.238565	-1645.338148	209.590
16d	11d	IIb	052bba_Rh_Julolidin_Et-H+carb_B3LYP631dp_PCMw_ring.log	-1645.80792977	-1645.280424	-1645.248725	-1645.247781	-1645.344278	203.095
16d	11d	IIc	053aaa_Rh_Julolidin_Et-H+oh_B3LYP631dp_PCMw.log	-1645.80511759	-1645.278501	-1645.245940	-1645.244996	-1645.345233	210.966
16d	11d	IIIa	054aaa_Rh_Julolidin_Et-2H+_B3LYP631dp_PCMw.log	-1645.32700969	-1644.813752	-1644.781343	-1644.780399	-1644.880124	209.890
16d	11d	IIIb	054bba_Rh_Julolidin_Et-H+carb_B3LYP631dp_PCMw_ring.log	-1645.31623970	-1644.802443	-1644.770905	-1644.769960	-1644.866568	203.328

Table S4 cont.

N	original	Type	Filename	E	ZPE	U	H	G	S
**16j**	**11j**	**I**	061aaa_Rh_Julolidin_naft1_B3LYP631dp_PCMw.log	-1721.27429075	-1720.743300	-1720.710946	-1720.710002	-1720.808638	207.598
**16j**	**11j**	**IIa**	062aaa_Rh_Julolidin_naft1-H+carb_B3LYP631dp_PCMw.log	-1720.80316159	-1720.285620	-1720.253466	-1720.252522	-1720.350276	205.741
**16j**	**11j**	**IIb**	062baa_Rh_Julolidin_naft1-H+carb_B3LYP631dp_PCMw.log	-1720.81230461	-1720.294211	-1720.262795	-1720.261851	-1720.357478	201.263
**16j**	**11j**	**IIc**	063aaa_Rh_Julolidin_naft1-H+oh_B3LYP631dp_PCMw.log	-1720.81478050	-1720.297340	-1720.265123	-1720.264179	-1720.362967	207.918
**16j**	**11j**	**IIIa**	064aaa_Rh_Julolidin_naft1-2H_B3LYP631dp_PCMw.log	-1720.33745488	-1719.833278	-1719.801280	-1719.800336	-1719.898311	206.205
**16j**	**11j**	**IIIb**	064bba_Rh_Julolidin_naft1-H+carb_B3LYP631dp_PCMw.log	-1720.32751392	-1719.822975	-1719.791796	-1719.790852	-1719.886118	200.504
**16l**	**11l**	**I**	071aaa_Rh_Julolidin_naft2_B3LYP631dp_PCMw.log	-1721.28217261	-1720.751392	-1720.718980	-1720.718035	-1720.817355	209.036
**16l**	**11l**	**IIa**	073aaa_Rh_Julolidin_naft2-H+carb_B3LYP631dp_PCMw.log	-1720.81096637	-1720.293758	-1720.261482	-1720.260538	-1720.359122	207.488
**16l**	**11l**	**IIb**	072baa_Rh_Julolidin_naft2-H+carb_B3LYP631dp_PCMw_ring.log	-1720.82085364	-1720.302780	-1720.271400	-1720.270456	-1720.366030	201.153
**16l**	**11l**	**IIc**	073aaa_Rh_Julolidin_naft2-H+_oh_B3LYP631dp_PCMw.log	-1720.80623260	-1720.289246	-1720.256919	-1720.255974	-1720.355811	210.125
**16l**	**11l**	**IIIa**	074aaa_Rh_Julolidin_naft2-2H+_oh_B3LYP631dp_PCMw.log	-1720.32959193	-1719.825697	-1719.793675	-1719.792731	-1719.890782	206.366
**16l**	**11l**	**IIIb**	074bba_Rh_Julolidin_naft2-H+carb_B3LYP631dp_PCMw_ring.log	-1720.32756466	-1719.823036	-1719.791904	-1719.790960	-1719.886147	200.338
**16n**	**11n**	**I**	081aaa_Rh_Julolidin_EtCOO_B3LYP631dp_PCMw.log	-1834.35688751	-1833.814827	-1833.779521	-1833.778577	-1833.886397	226.926
**16n**	**11n**	**IIa**	081aba_Rh_Julolidin_EtCOO_B3LYP631dp_PCMw.log	-1834.35693467	-1833.814851	-1833.779566	-1833.778622	-1833.886528	227.107
**16n**	**11n**	**IIb**	083aba_Rh_Julolidin_EtCOO-H+carb_B3LYP631dp_PCMw.log	-1833.88427658	-1833.355545	-1833.320473	-1833.319528	-1833.425942	223.966
**16n**	**11n**	**IIc**	082bba_Rh_Julolidin_EtCOO-H+carb_B3LYP631dp_PCMw_ring.log	-1833.89310867	-1833.363596	-1833.329335	-1833.328391	-1833.432581	219.286
**16n**	**11n**	**IIIa**	082aba_Rh_Julolidin_EtCOO-H+oh_B3LYP631dp_PCMw.log	-1833.88977410	-1833.361184	-1833.326080	-1833.325136	-1833.432532	226.035
**16n**	**11n**	**IIIb**	084aba_Rh_Julolidin_EtCOO-2H_B3LYP631dp_PCMw.log	-1833.41071184	-1832.895169	-1832.860387	-1832.859443	-1832.965485	223.185

Table S4 cont.

N	original	Type	Filename	E	ZPE	U	H	G	S
16a	11a	I	121aaa_Rh_Julolidin5COOH_OH_B3LYP631dp_PCMw.log	-1642.85566478	-1642.368176	-1642.336980	-1642.336036	-1642.432941	203.955
16a	11a	IIa	121aba_Rh_Julolidin5COOH_OH_B3LYP631dp_PCMw.log	-1642.85470747	-1642.367057	-1642.335946	-1642.335002	-1642.431609	203.326
16a	11a	IIb	122aaa_Rh_Julolidin5COOH_OH-H+_carb_B3LYP631dp_PCMw.log	-1642.38390989	-1641.909822	-1641.878822	-1641.877878	-1641.973364	200.967
16a	11a	IIc	122baa_Rh_Julolidin5COOH_OH-H+_carb_B3LYP631dp_PCMw_ring.log	-1642.39257683	-1641.917614	-1641.887458	-1641.886514	-1641.979731	196.192
16a	11a	IIIa	123aaa_Rh_Julolidin5COOH_OH-H+oh_B3LYP631dp_PCMw.log	-1642.39985859	-1641.924991	-1641.894488	-1641.893544	-1641.988655	200.179
16a	11a	IIIb	124aaa_Rh_Julolidin5COOH_OH-2H_B3LYP631dp_PCMw.log	-1641.92163133	-1641.460007	-1641.429715	-1641.428771	-1641.523187	198.715
16e	11e	I	131aaa_Rh_Julolidin5COOH_OMe_B3LYP631dp_PCMw.log	-1682.16028024	-1681.643958	-1681.611475	-1681.610531	-1681.710472	210.344
16e	11e	IIa	132aaa_Rh_Julolidin5COOH_OMe+H+carb_B3LYP631dp_PCMw.log	-1681.68841367	-1681.185476	-1681.153209	-1681.152265	-1681.251022	207.852
16e	11e	IIb	132baa_Rh_Julolidin5COOH_OMe+H+carb_B3LYP631dp_PCMw_ring.log	-1681.69674767	-1681.193099	-1681.161655	-1681.160710	-1681.256788	202.213
16e	11e	IIc	133aaa_Rh_Julolidin5COOH_OMe+H+oh_B3LYP631dp_PCMw.log	-1681.69305991	-1681.190127	-1681.157889	-1681.156944	-1681.256682	209.916
16e	11e	IIIa	134aaa_Rh_Julolidin5COOH_OMe-2H+_B3LYP631dp_PCMw.log	-1681.21451023	-1680.724722	-1680.692777	-1680.691832	-1680.790056	206.730
16g	11g	I	141aaa_Rh_Julolidin5COOH_F2_B3LYP631dp_PCMw.log	-1766.07626003	-1765.609177	-1765.577475	-1765.576531	-1765.674082	205.314
16g	11g	IIa	142aaa_Rh_Julolidin5COOH_F2-H+carb_B3LYP631dp_PCMw.log	-1765.61881775	-1765.164202	-1765.133570	-1765.132626	-1765.226630	197.848
16g	11g	IIb	142baa_Rh_Julolidin5COOH_F2-H+carb_B3LYP631dp_PCMw_ring.log	-1765.61881751	-1765.164205	-1765.133572	-1765.132627	-1765.226636	197.858
16g	11g	IIc	143aaa_Rh_Julolidin5COOH_F2-H+oh_B3LYP631dp_PCMw.log	-1765.61838604	-1765.164467	-1765.133012	-1765.132068	-1765.229361	204.770
16g	11g	IIIa	144aaa_Rh_Julolidin5COOH_F2-2H+_B3LYP631dp_PCMw.log	-1765.14144100	-1764.700858	-1764.669585	-1764.668641	-1764.765137	203.092
16c	11c	I	151aba_Rh_Julolidin5COOH_Et_B3LYP631dp_PCMw.log	-1646.27004258	-1645.729945	-1645.697180	-1645.696236	-1645.796721	211.489
16c	11c	IIa	152aba_Rh_Julolidin5COOH_Et-H+carb_B3LYP631dp_PCMw.log	-1645.79827341	-1645.271593	-1645.239027	-1645.238083	-1645.337602	209.457
16c	11c	IIb	152bba_Rh_Julolidin5CCOH_Et-H+carb_B3LYP631dp_PCMw_ring.log	-1645.80739615	-1645.279858	-1645.248173	-1645.247229	-1645.343755	203.156
16c	11c	IIc	153aaa_Rh_Julolidin5COOH_Et-H+oh_B3LYP631dp_PCMw.log	-1645.80468241	-1645.278146	-1645.245550	-1645.244606	-1645.345122	211.555
16c	11c	IIIa	154aaa_Rh_Julolidin5COOH_Et-2H+_B3LYP631dp_PCMw.log	-1645.32638396	-1644.813093	-1644.780718	-1644.779774	-1644.879526	209.946

Table S4 cont.

N	original	Type	Filename	E	ZPE	U	H	G	S
**16i**	**11i**	**I**	161aaa_Rh_Julolidin5COOH_naft1_B3LYP631dp_PCMw.log	-1721.27399453	-1720.743130	-1720.710732	-1720.709787	-1720.808683	208.143
**16i**	**11i**	**IIa**	162aaa_Rh_Julolidin5COOH_naft1-H+carb_B3LYP631dp_PCMw.log	-1720.80265645	-1720.285317	-1720.253100	-1720.252155	-1720.350299	206.562
**16i**	**11i**	**IIb**	162baa_Rh_Julolidin5COOH_naft1-H+carb_B3LYP631dp_PCMw_ring.log	-1720.81187701	-1720.293620	-1720.262242	-1720.261298	-1720.356856	201.118
**16i**	**11i**	**IIc**	163aaa_Rh_Julolidin5COOH_naft1-H+oh_B3LYP631dp_PCMw.log	-1720.81431307	-1720.296822	-1720.264651	-1720.263707	-1720.361997	206.867
**16i**	**11i**	**IIIa**	164aaa_Rh_Julolidin5COOH_naft1-2H_B3LYP631dp_PCMw.log	-1720.33681734	-1719.832653	-1719.800641	-1719.799697	-1719.897961	206.815
**16k**	**11k**	**I**	171aaa_Rh_Julolidin5COOH_naft2_B3LYP631dp_PCMw.log	-1721.28193482	-1720.751323	-1720.718846	-1720.717901	-1720.817639	209.915
**16k**	**11k**	**IIa**	172aaa_Rh_Julolidin5COOH_naft2-H+carb_B3LYP631dp_PCMw.log	-1720.81059644	-1720.293340	-1720.261112	-1720.260168	-1720.358355	206.651
**16k**	**11k**	**IIb**	172baa_Rh_Julolidin5COOH_naft2-H+carb_B3LYP631dp_PCMw_ring.log	-1720.82030874	-1720.302235	-1720.270835	-1720.269891	-1720.365684	201.614
**16k**	**11k**	**IIc**	173aaa_Rh_Julolidin5COOH_naft2-H+_oh_B3LYP631dp_PCMw.log	-1720.80594392	-1720.288872	-1720.256558	-1720.255614	-1720.355891	211.051
**16k**	**11k**	**IIIa**	174aaa_Rh_Julolidin5COOH_naft2-2H+_oh_B3LYP631dp_PCMw.log	-1720.32901648	-1719.825236	-1719.793191	-1719.792247	-1719.890502	206.794

Tables containing XYZ geometries of computed species

001aaa_TAMRA_B3LYP631dp_PCMw.log

Standard orientation:

Center Number	Atomic Number	Atomic Type	Coordinates (Angstroms) X	Y	Z
1	6	0	2.292009	-2.724003	-0.127222
2	6	0	1.237892	-1.860024	-0.146804
3	6	0	1.426673	-0.442247	-0.184078
4	6	0	2.784301	0.012876	-0.199425
5	6	0	3.863751	-0.841107	-0.181458
6	6	0	3.652517	-2.243913	-0.143319
7	6	0	0.389029	0.493791	-0.192698
8	6	0	2.070727	2.269744	-0.258590
9	6	0	0.704691	1.879167	-0.243210
10	6	0	-0.264883	2.916207	-0.271784
11	1	0	-1.316193	2.654529	-0.261218
12	6	0	0.106941	4.238867	-0.313400
13	6	0	1.480620	4.587355	-0.327996
14	6	0	2.465850	3.598590	-0.300816
15	1	0	2.097825	-3.787140	-0.099643
16	1	0	0.227225	-2.250304	-0.135076
17	1	0	4.855090	-0.411512	-0.195120
18	1	0	-0.645512	5.020487	-0.334755
19	1	0	3.516377	3.861195	-0.312849
20	8	0	3.062707	1.342402	-0.233584
21	7	0	4.688570	-3.107496	-0.123412
22	6	0	4.464527	-4.556275	-0.073987
23	1	0	3.912930	-4.901744	-0.953620
24	1	0	5.429049	-5.058863	-0.056705
25	1	0	3.913377	-4.840675	0.827517
26	6	0	6.065539	-2.608123	-0.146281
27	1	0	6.253797	-2.019907	-1.050127
28	1	0	6.272064	-1.985407	0.730271
29	1	0	6.749597	-3.453587	-0.137278
30	8	0	1.894490	5.866191	-0.369422
31	1	0	1.136153	6.469753	-0.385800
32	6	0	-1.039289	0.050190	-0.247740
33	6	0	-1.861732	-0.160503	0.880943
34	6	0	-1.577189	-0.168586	-1.524966
35	6	0	-3.189787	-0.568944	0.697199
36	6	0	-2.895306	-0.580216	-1.688393
37	1	0	-0.950322	-0.013832	-2.396745
38	6	0	-3.722225	-0.770542	-0.575848
39	1	0	-3.777395	-0.745233	1.590931
40	1	0	-3.298177	-0.758297	-2.678911
41	6	0	-1.431132	0.006283	2.304065
42	8	0	-2.146438	-0.242127	3.257054
43	8	0	-0.174817	0.464271	2.441987
44	1	0	0.007484	0.536401	3.395375
45	6	0	-5.137426	-1.226933	-0.822904
46	8	0	-5.437666	-1.817488	-1.865040
47	7	0	-6.038999	-0.959825	0.152546
48	6	0	-7.440701	-1.325936	0.014535
49	1	0	-7.915354	-1.280463	0.995390
50	1	0	-7.972000	-0.654417	-0.669885
51	1	0	-7.515132	-2.342349	-0.375675
52	1	0	-5.786437	-0.327699	0.897105

002aaa_TAMRA-H+carb_B3LYP631dp_PCMw.log

Standard orientation:

Center Number	Atomic Number	Atomic Type	Coordinates (Angstroms) X	Y	Z
1	6	0	2.346047	-2.695932	0.220929
2	6	0	1.284355	-1.827850	0.397104
3	6	0	1.412712	-0.435886	0.246796
4	6	0	2.684307	0.036805	-0.093361
5	6	0	3.775253	-0.808110	-0.282380
6	6	0	3.635194	-2.203205	-0.126912
7	6	0	0.268417	0.504220	0.511443
8	6	0	1.925190	2.277715	-0.219703
9	6	0	0.613114	1.920161	0.117451
10	6	0	-0.348185	2.943135	0.135357
11	1	0	-1.373377	2.699215	0.395173
12	6	0	-0.028669	4.258574	-0.166686
13	6	0	1.296451	4.585620	-0.500952
14	6	0	2.273850	3.593259	-0.530804
15	1	0	2.181670	-3.757145	0.353012
16	1	0	0.316411	-2.240603	0.665469
17	1	0	4.719507	-0.354590	-0.552091
18	1	0	-0.793203	5.029443	-0.144866
19	1	0	3.297610	3.833168	-0.793258
20	8	0	2.950882	1.373462	-0.280023
21	7	0	4.699162	-3.052332	-0.310803
22	6	0	4.536013	-4.486386	-0.114920
23	1	0	3.786094	-4.905926	-0.796864
24	1	0	5.484810	-4.982732	-0.315203
25	1	0	4.235338	-4.729503	0.912620
26	6	0	6.013414	-2.513780	-0.629356
27	1	0	6.003195	-1.954465	-1.573522
28	1	0	6.382504	-1.843001	0.157848
29	1	0	6.721510	-3.335046	-0.733322
30	8	0	1.683022	5.853085	-0.810287
31	1	0	0.921094	6.447512	-0.755598
32	6	0	-1.064620	0.025420	-0.040077
33	6	0	-1.954103	-0.201300	1.002281
34	6	0	-1.453588	-0.197269	-1.359134
35	6	0	-3.258011	-0.640596	0.784927
36	6	0	-2.753706	-0.642783	-1.589827
37	1	0	-0.769460	-0.033005	-2.185153
38	6	0	-3.665598	-0.852969	-0.536210
39	1	0	-3.911818	-0.830851	1.629873
40	1	0	-3.094076	-0.839443	-2.600436
41	6	0	-1.270343	0.094497	2.279551
42	8	0	-1.695703	0.014608	3.414224
43	8	0	-0.005148	0.501169	1.999877
44	6	0	-5.043319	-1.338551	-0.903871
45	8	0	-5.246642	-1.943442	-1.961737
46	7	0	-6.032878	-1.080938	-0.013005
47	6	0	-7.409210	-1.475105	-0.273011
48	1	0	-7.979937	-1.394566	0.652764
49	1	0	-7.877590	-0.842750	-1.036258
50	1	0	-7.435185	-2.508832	-0.622643
51	1	0	-5.860165	-0.433145	0.740869

002aba_TAMRA-H+carb_B3LYP631dp_PCMw.log

Standard orientation:

Center Number	Atomic Number	Atomic Type	Coordinates (Angstroms) X	Y	Z
1	6	0	2.346560	-2.694249	0.254877
2	6	0	1.284133	-1.825048	0.421603
3	6	0	1.414017	-0.433788	0.267129
4	6	0	2.686920	0.036485	-0.071003
5	6	0	3.778009	-0.810144	-0.252091
6	6	0	3.639542	-2.203266	-0.080288
7	6	0	0.268313	0.508129	0.519404
8	6	0	1.928043	2.276132	-0.218735

9	6	0	0.614796	1.921198	0.116560
10	6	0	-0.346425	2.944382	0.123596
11	1	0	-1.372532	2.702512	0.381725
12	6	0	-0.025698	4.257521	-0.187039
13	6	0	1.300621	4.581970	-0.519082
14	6	0	2.277973	3.589295	-0.538287
15	1	0	2.178439	-3.755265	0.383406
16	1	0	0.313655	-2.236853	0.682087
17	1	0	4.720951	-0.358576	-0.529310
18	1	0	-0.790236	5.028577	-0.173570
19	1	0	3.302685	3.827172	-0.798864
20	8	0	2.953915	1.371395	-0.268581
21	7	0	4.710631	-3.052297	-0.231359
22	6	0	4.513180	-4.494788	-0.175922
23	1	0	3.840541	-4.855144	-0.966375
24	1	0	5.476133	-4.989533	-0.298231
25	1	0	4.099301	-4.804111	0.790584
26	6	0	5.994189	-2.527924	-0.675608
27	1	0	5.938775	-2.085668	-1.680271
28	1	0	6.368750	-1.761199	0.012452
29	1	0	6.722390	-3.338010	-0.699614
30	8	0	1.688523	5.847085	-0.836219
31	1	0	0.926585	6.442095	-0.788416
32	6	0	-1.062148	0.025503	-0.034988
33	6	0	-1.956546	-0.193463	1.004818
34	6	0	-1.445073	-0.206337	-1.354231
35	6	0	-3.259647	-0.633700	0.784524
36	6	0	-2.744319	-0.652908	-1.587795
37	1	0	-0.757078	-0.048161	-2.178223
38	6	0	-3.661204	-0.855170	-0.536969
39	1	0	-3.917476	-0.817718	1.627727
40	1	0	-3.080073	-0.856525	-2.598572
41	6	0	-1.278550	0.111071	2.283092
42	8	0	-1.708924	0.039140	3.416372
43	8	0	-0.011992	0.515734	2.006285
44	6	0	-5.037526	-1.342486	-0.907582
45	8	0	-5.236249	-1.955076	-1.961885
46	7	0	-6.031148	-1.077564	-0.023395
47	6	0	-7.406467	-1.472855	-0.287042
48	1	0	-7.981663	-1.384526	0.635247
49	1	0	-7.870660	-0.846295	-1.057601
50	1	0	-7.431467	-2.509320	-0.628553
51	1	0	-5.861747	-0.424000	0.726239

003aaa_TAMRA-H+_oH_B3LYP631dp_PCMw.log

Standard orientation:

Center Number	Atomic Number	Atomic Type	Coordinates (Angstroms) X	Y	Z
1	6	0	2.271453	-2.683822	-0.164828
2	6	0	1.224291	-1.793090	-0.169872
3	6	0	1.429945	-0.390587	-0.186561
4	6	0	2.778270	0.044248	-0.200726
5	6	0	3.851559	-0.829586	-0.196587
6	6	0	3.627615	-2.226981	-0.176765
7	6	0	0.390177	0.587236	-0.187373
8	6	0	2.104686	2.331844	-0.236322
9	6	0	0.715646	1.936637	-0.224734
10	6	0	-0.250206	3.005834	-0.235042
11	1	0	-1.302774	2.742764	-0.222342
12	6	0	0.128175	4.309576	-0.259505
13	6	0	1.539304	4.717086	-0.275996
14	6	0	2.506173	3.637015	-0.261919
15	1	0	2.056476	-3.743730	-0.152712
16	1	0	0.208901	-2.173586	-0.162610
17	1	0	4.847832	-0.409961	-0.209342
18	1	0	-0.610221	5.105839	-0.266863
19	1	0	3.563108	3.880214	-0.271266

20	8	0	3.084734	1.373729	-0.221433
21	7	0	4.667822	-3.108226	-0.170947
22	6	0	4.422245	-4.547967	-0.152182
23	1	0	3.861371	-4.872927	-1.036066
24	1	0	5.377901	-5.069523	-0.147569
25	1	0	3.865611	-4.849968	0.742385
26	6	0	6.042502	-2.618464	-0.187300
27	1	0	6.245824	-2.024721	-1.086419
28	1	0	6.257277	-1.998408	0.691096
29	1	0	6.723785	-3.467436	-0.178983
30	8	0	1.871899	5.921741	-0.300092
31	6	0	-1.039461	0.143850	-0.245177
32	6	0	-1.838987	-0.171366	0.876538
33	6	0	-1.604750	0.016436	-1.523826
34	6	0	-3.165795	-0.584558	0.685661
35	6	0	-2.918544	-0.403220	-1.697311
36	1	0	-0.996205	0.249350	-2.391355
37	6	0	-3.720975	-0.695614	-0.588431
38	1	0	-3.733089	-0.838212	1.573972
39	1	0	-3.338458	-0.509001	-2.691319
40	6	0	-1.393554	-0.110828	2.303856
41	8	0	-2.094021	-0.452857	3.240635
42	8	0	-0.147265	0.361297	2.475672
43	1	0	0.033078	0.351707	3.432110
44	6	0	-5.132169	-1.156287	-0.844089
45	8	0	-5.447112	-1.662137	-1.926142
46	7	0	-6.017295	-0.992376	0.169438
47	6	0	-7.414221	-1.374895	0.029916
48	1	0	-7.864048	-1.439936	1.021463
49	1	0	-7.978379	-0.651134	-0.569955
50	1	0	-7.477001	-2.348237	-0.459415
51	1	0	-5.760820	-0.418030	0.958072

004aaa_TAMRA-H+_oH_-H+carb_B3LYP631dp_PCMw.log

Standard orientation:

Center Number	Atomic Number	Atomic Type	Coordinates (Angstroms) X	Y	Z
1	6	0	2.244791	-2.684019	-0.149551
2	6	0	1.204047	-1.783871	-0.122828
3	6	0	1.417188	-0.384391	-0.129350
4	6	0	2.765182	0.040020	-0.165966
5	6	0	3.834280	-0.842463	-0.193154
6	6	0	3.602355	-2.237070	-0.183121
7	6	0	0.374581	0.596888	-0.093103
8	6	0	2.107650	2.329887	-0.190156
9	6	0	0.720373	1.945504	-0.160908
10	6	0	-0.232873	3.022539	-0.168842
11	1	0	-1.286829	2.766038	-0.138096
12	6	0	0.154469	4.325454	-0.209952
13	6	0	1.565805	4.722554	-0.246626
14	6	0	2.520553	3.634017	-0.233236
15	1	0	2.022069	-3.742624	-0.143825
16	1	0	0.185164	-2.153791	-0.094544
17	1	0	4.832792	-0.428659	-0.223385
18	1	0	-0.579437	5.126390	-0.214887
19	1	0	3.580067	3.865799	-0.260043
20	8	0	3.085149	1.367377	-0.181955
21	7	0	4.639096	-3.127168	-0.207533
22	6	0	4.383238	-4.563907	-0.198769
23	1	0	3.803254	-4.877423	-1.074968
24	1	0	5.334726	-5.093229	-0.216975
25	1	0	3.840405	-4.872555	0.702445
26	6	0	6.014837	-2.645413	-0.244830
27	1	0	6.206453	-2.043109	-1.141334
28	1	0	6.251133	-2.034668	0.635070
29	1	0	6.691868	-3.498022	-0.257822
30	8	0	1.910015	5.926626	-0.287778

31	6	0	-1.053795	0.163523	-0.203761
32	6	0	-1.842318	-0.191724	0.906155
33	6	0	-1.607981	0.091323	-1.493748
34	6	0	-3.163834	-0.597055	0.694610
35	6	0	-2.922278	-0.320388	-1.685157
36	1	0	-0.998064	0.359224	-2.351546
37	6	0	-3.722112	-0.659786	-0.585899
38	1	0	-3.716533	-0.882281	1.584266
39	1	0	-3.344851	-0.382933	-2.681909
40	6	0	-1.298677	-0.145308	2.343799
41	8	0	-2.079250	-0.534313	3.249883
42	8	0	-0.119408	0.281320	2.470285
43	6	0	-5.133671	-1.100907	-0.857436
44	8	0	-5.480298	-1.505665	-1.974082
45	7	0	-6.001732	-1.037300	0.184505
46	6	0	-7.399739	-1.407630	0.037741
47	1	0	-7.830367	-1.569570	1.026977
48	1	0	-7.978995	-0.631854	-0.477580
49	1	0	-7.473770	-2.329327	-0.542220
50	1	0	-5.722704	-0.555556	1.025272

011aaa_Rh_Julolidin_H_B3LYP631dp_PCMw.log

Standard orientation:

Center Number	Atomic Number	Atomic Type	Coordinates (Angstroms) X	Y	Z
1	6	0	-2.021462	-2.239043	0.048884
2	6	0	-0.903908	-1.459572	-0.015735
3	6	0	-0.964064	-0.034676	-0.098271
4	6	0	-2.274185	0.544852	-0.109921
5	6	0	-3.440434	-0.193158	-0.070711
6	6	0	-3.334307	-1.615984	0.007324
7	6	0	0.156715	0.794064	-0.151303
8	6	0	-1.342084	2.724887	-0.233995
9	6	0	-0.021897	2.205810	-0.232493
10	6	0	1.039744	3.143200	-0.314495
11	1	0	2.061706	2.783272	-0.322799
12	6	0	0.795183	4.495560	-0.386531
13	6	0	-0.537591	4.974031	-0.383302
14	6	0	-1.610352	4.084579	-0.307061
15	1	0	0.070648	-1.934062	0.009744
16	1	0	1.618964	5.199656	-0.447576
17	1	0	-2.631618	4.445046	-0.307874
18	8	0	-2.417610	1.898037	-0.166538
19	7	0	-4.446751	-2.383608	0.043931
20	6	0	-4.399191	-3.851596	0.153469
21	1	0	-5.277251	-4.241517	-0.367448
22	1	0	-4.494601	-4.134491	1.211047
23	6	0	-5.786222	-1.782342	0.150124
24	8	0	-0.830745	6.286677	-0.455838
25	1	0	-0.019089	6.812668	-0.515569
26	6	0	1.532806	0.210744	-0.227031
27	6	0	2.411944	0.069325	0.869568
28	6	0	1.973135	-0.190517	-1.497551
29	6	0	3.689729	-0.468936	0.663528
30	6	0	3.246239	-0.716689	-1.685330
31	1	0	1.307346	-0.079023	-2.346823
32	6	0	4.116951	-0.872798	-0.601128
33	1	0	4.338592	-0.535287	1.529391
34	1	0	3.580968	-1.010786	-2.673541
35	6	0	2.096644	0.449531	2.281841
36	8	0	2.909603	0.407990	3.187178
37	8	0	0.822706	0.834383	2.471570
38	1	0	0.724604	1.062837	3.412620
39	6	0	5.487555	-1.434850	-0.880219
40	8	0	5.973919	-1.376431	-2.014002
41	7	0	6.143995	-1.989594	0.166989
42	6	0	7.461776	-2.585894	0.007112

43	1	0	7.914050	-2.711906	0.991422
44	1	0	7.410278	-3.562271	-0.488634
45	1	0	8.088848	-1.927708	-0.596742
46	1	0	5.645310	-2.167916	1.025519
47	6	0	-3.122399	-4.427085	-0.441900
48	1	0	-3.098814	-5.504635	-0.258636
49	1	0	-3.121364	-4.275653	-1.527083
50	6	0	-4.787556	0.493460	-0.111840
51	6	0	-5.867055	-0.465985	-0.610441
52	6	0	-1.913995	-3.739157	0.189552
53	1	0	-6.861221	-0.032224	-0.473252
54	1	0	-5.736298	-0.659121	-1.680869
55	1	0	-5.046242	0.856679	0.891769
56	1	0	-4.732771	1.376936	-0.752888
57	1	0	-0.978433	-4.085679	-0.258152
58	1	0	-1.868756	-3.999036	1.256193
59	1	0	-6.500348	-2.505362	-0.249599
60	1	0	-6.028519	-1.633817	1.211508

012aaa_Rh_Julolidin_H_-H+_carb_B3LYP631dp_PCMw.log

Standard orientation:

Center Number	Atomic Number	Atomic Type	Coordinates (Angstroms) X	Y	Z
1	6	0	-1.983575	-2.238067	0.037506
2	6	0	-0.869438	-1.442311	0.020133
3	6	0	-0.938814	-0.022964	-0.027074
4	6	0	-2.243342	0.542378	-0.059860
5	6	0	-3.408232	-0.210060	-0.067003
6	6	0	-3.294013	-1.628871	-0.014300
7	6	0	0.195316	0.816519	0.006210
8	6	0	-1.334727	2.730970	-0.161918
9	6	0	-0.013161	2.230554	-0.129482
10	6	0	1.035194	3.177865	-0.207745
11	1	0	2.060159	2.826816	-0.182707
12	6	0	0.779340	4.528439	-0.313048
13	6	0	-0.556151	4.990517	-0.340702
14	6	0	-1.617037	4.088825	-0.266424
15	1	0	0.109637	-1.907668	0.059081
16	1	0	1.597323	5.239786	-0.372673
17	1	0	-2.642967	4.435454	-0.293378
18	8	0	-2.408538	1.897669	-0.100991
19	7	0	-4.409660	-2.410039	-0.024025
20	6	0	-4.345357	-3.873334	0.085981
21	1	0	-5.217481	-4.276933	-0.435999
22	1	0	-4.434341	-4.168389	1.141983
23	6	0	-5.747134	-1.816269	0.091404
24	8	0	-0.864456	6.302668	-0.443632
25	1	0	-0.055112	6.832281	-0.495646
26	6	0	1.565679	0.247795	-0.183625
27	6	0	2.405902	0.018526	0.913807
28	6	0	2.020382	-0.043835	-1.478348
29	6	0	3.683446	-0.504060	0.711907
30	6	0	3.299008	-0.559475	-1.669024
31	1	0	1.375661	0.135316	-2.333416
32	6	0	4.141319	-0.806010	-0.575307
33	1	0	4.300718	-0.637371	1.594857
34	1	0	3.662224	-0.778760	-2.666808
35	6	0	1.889706	0.351669	2.307380
36	8	0	2.640846	0.150592	3.286242
37	8	0	0.705652	0.813721	2.319016
38	6	0	5.510992	-1.359082	-0.863047
39	8	0	6.028850	-1.244330	-1.980165
40	7	0	6.144471	-1.980903	0.163180
41	6	0	7.458519	-2.580256	-0.006383
42	1	0	7.883633	-2.784998	0.977007
43	1	0	7.411575	-3.517014	-0.574501
44	1	0	8.110588	-1.888708	-0.543362

45	1	0	5.628151	-2.197178	1.002100
46	6	0	-3.059843	-4.429272	-0.512191
47	1	0	-3.027082	-5.510195	-0.349245
48	1	0	-3.055401	-4.257516	-1.594611
49	6	0	-4.758950	0.469825	-0.132363
50	6	0	-5.827829	-0.490120	-0.653936
51	6	0	-1.859665	-3.742065	0.136881
52	1	0	-6.825879	-0.061895	-0.525180
53	1	0	-5.682651	-0.670936	-1.724918
54	1	0	-5.039704	0.831107	0.866306
55	1	0	-4.696425	1.356159	-0.769397
56	1	0	-0.921063	-4.066703	-0.321974
57	1	0	-1.808862	-4.034683	1.194881
58	1	0	-6.461350	-2.533876	-0.319829
59	1	0	-5.999056	-1.677053	1.153025

012baa_Rh_Julolidin_H_-H+_carb_B3LYP631dp_PCMw.log

Standard orientation:

Center Number	Atomic Number	Atomic Type	Coordinates (Angstroms) X	Y	Z
1	6	0	-1.992926	-2.219499	0.270332
2	6	0	-0.878568	-1.411334	0.422037
3	6	0	-0.924850	-0.014021	0.301903
4	6	0	-2.169156	0.549178	0.007982
5	6	0	-3.329770	-0.212110	-0.177875
6	6	0	-3.249136	-1.619000	-0.040516
7	6	0	0.288725	0.839800	0.555008
8	6	0	-1.253520	2.737629	-0.096349
9	6	0	0.039066	2.282397	0.193222
10	6	0	1.071634	3.233674	0.197034
11	1	0	2.083804	2.912982	0.422179
12	6	0	0.838233	4.573415	-0.075487
13	6	0	-0.469485	4.999125	-0.365197
14	6	0	-1.516086	4.079900	-0.378097
15	1	0	0.070650	-1.886089	0.655876
16	1	0	1.655910	5.287918	-0.064163
17	1	0	-2.527048	4.396611	-0.606562
18	8	0	-2.340799	1.909789	-0.134876
19	7	0	-4.372916	-2.408462	-0.246671
20	6	0	-4.347420	-3.825157	0.110934
21	1	0	-5.166013	-4.315371	-0.425899
22	1	0	-4.540792	-3.964674	1.188463
23	6	0	-5.691152	-1.781590	-0.280294
24	8	0	-0.773039	6.295568	-0.646892
25	1	0	0.031902	6.832068	-0.614188
26	6	0	1.570755	0.273852	-0.033461
27	6	0	2.455954	-0.053426	0.985510
28	6	0	1.924858	0.072178	-1.365692
29	6	0	3.712714	-0.598459	0.732472
30	6	0	3.182565	-0.465020	-1.632768
31	1	0	1.248070	0.326283	-2.174846
32	6	0	4.075905	-0.815833	-0.600791
33	1	0	4.383515	-0.813461	1.557894
34	1	0	3.503575	-0.625280	-2.656057
35	6	0	1.829345	0.282971	2.282031
36	8	0	2.274866	0.155800	3.404867
37	8	0	0.594600	0.791307	2.038304
38	6	0	5.411828	-1.379354	-1.009170
39	8	0	5.888174	-1.147418	-2.125264
40	7	0	6.055458	-2.138986	-0.088202
41	6	0	7.338522	-2.760687	-0.378689
42	1	0	7.790964	-3.090346	0.557306
43	1	0	7.232615	-3.623762	-1.046381
44	1	0	7.995610	-2.034346	-0.860246
45	1	0	5.553559	-2.450864	0.729343
46	6	0	-3.010466	-4.458251	-0.261090
47	1	0	-3.017543	-5.517856	0.012325

48	1	0	-2.874789	-4.398819	-1.347381
49	6	0	-4.636262	0.478153	-0.515615
50	6	0	-5.652759	-0.500665	-1.106655
51	6	0	-1.878819	-3.720053	0.455207
52	1	0	-6.648576	-0.047756	-1.134507
53	1	0	-5.378159	-0.753061	-2.137440
54	1	0	-5.053052	0.941585	0.389588
55	1	0	-4.450911	1.301375	-1.211967
56	1	0	-0.902304	-4.065240	0.098752
57	1	0	-1.917223	-3.963192	1.526623
58	1	0	-6.389655	-2.500757	-0.719133
59	1	0	-6.050693	-1.563036	0.739882

013aaa_Rh_Julolidin_H_-H+_oh_B3LYP631dp_PCMw.log

Standard orientation:

Center Number	Atomic Number	Atomic Type	Coordinates (Angstroms) X	Y	Z
1	6	0	-2.033304	-2.196559	0.013845
2	6	0	-0.909514	-1.405771	-0.045376
3	6	0	-0.971862	0.006869	-0.117648
4	6	0	-2.266845	0.581240	-0.130179
5	6	0	-3.438202	-0.165397	-0.095040
6	6	0	-3.333988	-1.582376	-0.021161
7	6	0	0.165267	0.863118	-0.166639
8	6	0	-1.344712	2.784264	-0.236888
9	6	0	-0.008815	2.241306	-0.238295
10	6	0	1.065104	3.197374	-0.308850
11	1	0	2.083624	2.823326	-0.319612
12	6	0	0.829061	4.534394	-0.362820
13	6	0	-0.528379	5.093650	-0.354949
14	6	0	-1.604277	4.125442	-0.291259
15	1	0	0.064693	-1.883478	-0.025831
16	1	0	1.649898	5.243565	-0.415658
17	1	0	-2.629113	4.480691	-0.288448
18	8	0	-2.421014	1.938730	-0.180017
19	7	0	-4.463525	-2.358266	0.000861
20	6	0	-4.398468	-3.811075	0.183081
21	1	0	-5.292826	-4.238867	-0.279614
22	1	0	-4.439087	-4.060151	1.255001
23	6	0	-5.786659	-1.749071	0.156057
24	8	0	-0.729487	6.327714	-0.403286
25	6	0	1.537241	0.265536	-0.236869
26	6	0	2.392489	0.064954	0.869465
27	6	0	2.004003	-0.089681	-1.512222
28	6	0	3.670931	-0.475896	0.667867
29	6	0	3.272926	-0.625942	-1.696648
30	1	0	1.357936	0.068184	-2.369521
31	6	0	4.119816	-0.836417	-0.602071
32	1	0	4.303608	-0.578439	1.542187
33	1	0	3.625035	-0.884164	-2.688999
34	6	0	2.059348	0.393780	2.290550
35	8	0	2.880640	0.389732	3.190879
36	8	0	0.762508	0.675283	2.502717
37	1	0	0.663811	0.872835	3.450695
38	6	0	5.487822	-1.405643	-0.873795
39	8	0	5.997576	-1.320188	-1.995925
40	7	0	6.119602	-2.000257	0.167592
41	6	0	7.433013	-2.606722	0.012194
42	1	0	7.864882	-2.772217	0.999886
43	1	0	7.380511	-3.564850	-0.518062
44	1	0	8.079676	-1.936062	-0.556375
45	1	0	5.603173	-2.199328	1.010889
46	6	0	-3.140007	-4.398387	-0.444213
47	1	0	-3.107550	-5.474080	-0.248300
48	1	0	-3.176609	-4.261115	-1.531089
49	6	0	-4.784870	0.525061	-0.134422
50	6	0	-5.878292	-0.433204	-0.607230

51	6	0	-1.910304	-3.700362	0.135390
52	1	0	-6.868141	0.007398	-0.456642
53	1	0	-5.766623	-0.632750	-1.679082
54	1	0	-5.034523	0.907771	0.864840
55	1	0	-4.732950	1.400027	-0.788273
56	1	0	-0.995233	-4.038287	-0.361031
57	1	0	-1.809415	-3.976173	1.194513
58	1	0	-6.524297	-2.463393	-0.219876
59	1	0	-6.005926	-1.587847	1.223038

014aaa_Rh_Julolidin_H-H+carb_-H+oh_B3LYP631dp_PCMw.log

Standard orientation:

Center Number	Atomic Number	Atomic Type	Coordinates (Angstroms) X	Y	Z
1	6	0	-1.941120	-2.199798	-0.008370
2	6	0	-0.840586	-1.371981	-0.016403
3	6	0	-0.942971	0.037379	-0.056459
4	6	0	-2.251132	0.570901	-0.098125
5	6	0	-3.401568	-0.213293	-0.115572
6	6	0	-3.256614	-1.625380	-0.066346
7	6	0	0.177890	0.925132	-0.046265
8	6	0	-1.396927	2.800067	-0.154721
9	6	0	-0.049101	2.299340	-0.127782
10	6	0	0.992129	3.289915	-0.156201
11	1	0	2.020753	2.945095	-0.129434
12	6	0	0.716995	4.621188	-0.210635
13	6	0	-0.654788	5.137892	-0.243036
14	6	0	-1.698017	4.135098	-0.211556
15	1	0	0.148658	-1.816391	0.023071
16	1	0	1.517718	5.355161	-0.229959
17	1	0	-2.734176	4.456096	-0.236497
18	8	0	-2.450853	1.923986	-0.130874
19	7	0	-4.366070	-2.437839	-0.097915
20	6	0	-4.256874	-3.885712	0.088763
21	1	0	-5.130372	-4.344143	-0.384879
22	1	0	-4.301601	-4.138799	1.160279
23	6	0	-5.705586	-1.868210	0.051810
24	8	0	-0.895136	6.367473	-0.296129
25	6	0	1.563406	0.371674	-0.172344
26	6	0	2.353428	-0.010632	0.927646
27	6	0	2.079713	0.230653	-1.472560
28	6	0	3.629445	-0.533273	0.693119
29	6	0	3.352801	-0.285790	-1.685734
30	1	0	1.472586	0.530732	-2.321686
31	6	0	4.140987	-0.687296	-0.598998
32	1	0	4.205588	-0.783464	1.578151
33	1	0	3.750366	-0.385550	-2.689724
34	6	0	1.869257	0.144313	2.379595
35	8	0	2.679032	-0.201579	3.278070
36	8	0	0.704827	0.603218	2.525056
37	6	0	5.510990	-1.233083	-0.890831
38	8	0	6.082036	-1.005787	-1.964735
39	7	0	6.088403	-1.981282	0.084130
40	6	0	7.397449	-2.587561	-0.094818
41	1	0	7.781047	-2.897432	0.878253
42	1	0	7.358588	-3.462435	-0.755085
43	1	0	8.080987	-1.859324	-0.535394
44	1	0	5.530748	-2.275590	0.871129
45	6	0	-2.971094	-4.429923	-0.522935
46	1	0	-2.909152	-5.506450	-0.337962
47	1	0	-2.995151	-4.281911	-1.608871
48	6	0	-4.766318	0.439156	-0.185977
49	6	0	-5.821723	-0.543820	-0.693867
50	6	0	-1.771713	-3.702028	0.083516
51	1	0	-6.826968	-0.134054	-0.557774
52	1	0	-5.682955	-0.724905	-1.765958
53	1	0	-5.052881	0.808710	0.808437

54	1	0	-4.721938	1.320450	-0.832243
55	1	0	-0.840986	-4.000658	-0.409518
56	1	0	-1.671881	-3.997026	1.137653
57	1	0	-6.420135	-2.595611	-0.344133
58	1	0	-5.944561	-1.726707	1.118051

014bba_Rh_Julolidin_H-H+carb_B3LYP631dp_PCMw_ring.log

Standard orientation:

Center Number	Atomic Number	Atomic Type	Coordinates (Angstroms) X	Y	Z
1	6	0	-1.967366	-2.206145	0.181800
2	6	0	-0.857342	-1.387690	0.319786
3	6	0	-0.922893	0.011838	0.249332
4	6	0	-2.184857	0.574314	0.025684
5	6	0	-3.342289	-0.201609	-0.138797
6	6	0	-3.240073	-1.610114	-0.055822
7	6	0	0.278494	0.881162	0.489780
8	6	0	-1.281698	2.778288	-0.086377
9	6	0	0.025002	2.299992	0.122232
10	6	0	1.055978	3.268365	0.058899
11	1	0	2.080907	2.942236	0.223053
12	6	0	0.808297	4.598875	-0.197965
13	6	0	-0.532513	5.110162	-0.417476
14	6	0	-1.566020	4.108999	-0.347760
15	1	0	0.105638	-1.857975	0.501452
16	1	0	-2.598124	4.404659	-0.515173
17	8	0	-2.376288	1.928848	-0.058433
18	7	0	-4.365365	-2.408440	-0.249348
19	6	0	-4.308802	-3.829710	0.082532
20	1	0	-5.149476	-4.319708	-0.419621
21	1	0	-4.444031	-3.991256	1.166471
22	6	0	-5.687766	-1.794481	-0.182929
23	8	0	-0.768250	6.334594	-0.657880
24	6	0	1.576436	0.309917	-0.051552
25	6	0	2.443571	-0.018669	0.982653
26	6	0	1.961315	0.110504	-1.376526
27	6	0	3.706274	-0.558874	0.751947
28	6	0	3.223839	-0.425164	-1.622043
29	1	0	1.300114	0.369274	-2.197215
30	6	0	4.098537	-0.774703	-0.573782
31	1	0	4.360447	-0.771191	1.591523
32	1	0	3.564164	-0.583624	-2.639465
33	6	0	1.796479	0.311281	2.274145
34	8	0	2.248924	0.172033	3.398317
35	8	0	0.574400	0.812137	2.027329
36	6	0	5.442810	-1.334601	-0.954966
37	8	0	5.935169	-1.118565	-2.067859
38	7	0	6.080587	-2.075592	-0.013909
39	6	0	7.371942	-2.691029	-0.278334
40	1	0	7.806563	-3.018946	0.666680
41	1	0	7.284158	-3.554543	-0.948191
42	1	0	8.036666	-1.962387	-0.746134
43	1	0	5.571789	-2.376887	0.803179
44	6	0	-2.986392	-4.441313	-0.369529
45	1	0	-2.971270	-5.508020	-0.124903
46	1	0	-2.904781	-4.351611	-1.459265
47	6	0	-4.669434	0.484077	-0.396066
48	6	0	-5.707028	-0.485706	-0.965275
49	6	0	-1.827531	-3.710956	0.310670
50	1	0	-6.707588	-0.043743	-0.925507
51	1	0	-5.485614	-0.700788	-2.017255
52	1	0	-5.046755	0.920322	0.539722
53	1	0	-4.522373	1.328545	-1.076206
54	1	0	-0.867243	-4.032861	-0.107147
55	1	0	-1.807083	-3.990756	1.373812
56	1	0	-6.405787	-2.505072	-0.604498
57	1	0	-5.989456	-1.613112	0.863589

58	1	0	1.629117	5.311227	-0.243474

021aaa_Rh_Julolidin_OH_B3LYP631dp_PCMw.log

Standard orientation:

Center Number	Atomic Number	Atomic Type	Coordinates (Angstroms) X	Y	Z
1	6	0	-2.211739	-2.280375	0.011300
2	6	0	-1.051098	-1.564331	-0.039946
3	6	0	-1.031203	-0.137673	-0.096958
4	6	0	-2.306028	0.512865	-0.100886
5	6	0	-3.513538	-0.159699	-0.073967
6	6	0	-3.487468	-1.585117	-0.018335
7	6	0	0.135182	0.629109	-0.133634
8	6	0	-1.250161	2.635618	-0.187549
9	6	0	0.038973	2.048503	-0.191709
10	6	0	1.161864	2.918238	-0.251931
11	1	0	2.162572	2.501641	-0.262412
12	6	0	0.985802	4.279890	-0.298238
13	6	0	-0.327919	4.839574	-0.289812
14	6	0	-1.441569	4.014041	-0.236029
15	1	0	-0.105618	-2.094903	-0.024327
16	1	0	-2.439881	4.433712	-0.232637
17	8	0	-2.370604	1.871247	-0.136671
18	7	0	-4.642443	-2.291309	0.005814
19	6	0	-4.674385	-3.760134	0.098893
20	1	0	-5.575085	-4.096575	-0.420876
21	1	0	-4.778414	-4.051806	1.153468
22	6	0	-5.944594	-1.617089	0.125614
23	8	0	-0.483026	6.173085	-0.337148
24	1	0	0.397562	6.584871	-0.372596
25	6	0	1.476421	-0.030114	-0.212637
26	6	0	2.323965	-0.273594	0.891009
27	6	0	1.915859	-0.402918	-1.492196
28	6	0	3.569293	-0.882626	0.682584
29	6	0	3.157265	-0.999340	-1.682060
30	1	0	1.274183	-0.214410	-2.346455
31	6	0	3.995243	-1.257032	-0.591417
32	1	0	4.195814	-1.026358	1.555438
33	1	0	3.492520	-1.270908	-2.676563
34	6	0	2.009439	0.069612	2.313083
35	8	0	2.789369	-0.096918	3.233141
36	8	0	0.777789	0.576691	2.493563
37	1	0	0.678014	0.767335	3.442840
38	6	0	5.334692	-1.888994	-0.872037
39	8	0	5.845232	-1.812544	-1.994098
40	7	0	5.937171	-2.525463	0.161021
41	6	0	7.220717	-3.191370	-0.002764
42	1	0	7.644605	-3.388549	0.982494
43	1	0	7.122197	-4.139401	-0.544234
44	1	0	7.897838	-2.545126	-0.563997
45	1	0	5.412840	-2.708624	1.003151
46	6	0	-3.433927	-4.396815	-0.511543
47	1	0	-3.469066	-5.476773	-0.344765
48	1	0	-3.429121	-4.228933	-1.594331
49	6	0	-4.819319	0.603239	-0.105869
50	6	0	-5.951912	-0.286955	-0.615598
51	6	0	-2.186569	-3.786815	0.124336
52	1	0	-6.919891	0.200378	-0.470559
53	1	0	-5.833430	-0.471697	-1.688965
54	1	0	-5.057286	0.969330	0.901845
55	1	0	-4.714351	1.489632	-0.736782
56	1	0	-1.273442	-4.175868	-0.334794
57	1	0	-2.150236	-4.068335	1.185884
58	1	0	-6.700128	-2.291929	-0.282595
59	1	0	-6.177169	-1.469838	1.189576
60	8	0	1.975299	5.212747	-0.355904
61	1	0	2.849687	4.797891	-0.358948

021aba_Rh_Julolidin_OH_B3LYP631dp_PCMw_f.log

Standard orientation:

Center Number	Atomic Number	Atomic Type	Coordinates (Angstroms) X	Y	Z
1	6	0	-2.198792	-2.291847	0.015465
2	6	0	-1.041308	-1.571502	-0.035689
3	6	0	-1.026815	-0.144148	-0.093123
4	6	0	-2.305128	0.501192	-0.096044
5	6	0	-3.509786	-0.176237	-0.069032
6	6	0	-3.477849	-1.601539	-0.014532
7	6	0	0.135981	0.626110	-0.131109
8	6	0	-1.256610	2.625866	-0.185167
9	6	0	0.035022	2.047327	-0.190651
10	6	0	1.152133	2.920473	-0.255980
11	1	0	2.158210	2.520159	-0.269288
12	6	0	0.977794	4.284623	-0.307216
13	6	0	-0.340529	4.830841	-0.295887
14	6	0	-1.452674	4.005035	-0.236375
15	1	0	-0.093715	-2.098180	-0.019753
16	1	0	-2.458930	4.408476	-0.229626
17	8	0	-2.374864	1.858763	-0.131267
18	7	0	-4.629411	-2.312474	0.008678
19	6	0	-4.656008	-3.781832	0.097645
20	1	0	-5.554014	-4.120085	-0.425563
21	1	0	-4.762086	-4.076239	1.151197
22	6	0	-5.934666	-1.643857	0.128225
23	8	0	-0.384433	6.179073	-0.350761
24	1	0	-1.295548	6.507976	-0.346797
25	6	0	1.479432	-0.027989	-0.211126
26	6	0	2.333301	-0.257853	0.890468
27	6	0	1.914574	-0.409713	-1.489477
28	6	0	3.580434	-0.862901	0.681535
29	6	0	3.157849	-1.002021	-1.680005
30	1	0	1.268162	-0.231347	-2.342337
31	6	0	4.002078	-1.246557	-0.591163
32	1	0	4.211587	-0.996333	1.552699
33	1	0	3.489689	-1.280751	-2.673673
34	6	0	2.023068	0.095156	2.310943
35	8	0	2.809777	-0.054140	3.228191
36	8	0	0.786510	0.589498	2.493828
37	1	0	0.690306	0.788687	3.441708
38	6	0	5.342421	-1.876151	-0.872617
39	8	0	5.847185	-1.808314	-1.997843
40	7	0	5.952242	-2.501304	0.163065
41	6	0	7.237205	-3.164315	-0.001300
42	1	0	7.665423	-3.353614	0.983626
43	1	0	7.139520	-4.116251	-0.536039
44	1	0	7.910297	-2.519844	-0.569416
45	1	0	5.432842	-2.678692	1.009461
46	6	0	-3.411549	-4.412443	-0.510748
47	1	0	-3.442645	-5.492723	-0.345364
48	1	0	-3.404828	-4.243213	-1.593309
49	6	0	-4.818640	0.581369	-0.100528
50	6	0	-5.947305	-0.313026	-0.611598
51	6	0	-2.168277	-3.798034	0.128859
52	1	0	-6.917384	0.170019	-0.466315
53	1	0	-5.827709	-0.496107	-1.685115
54	1	0	-5.058405	0.945404	0.907497
55	1	0	-4.717137	1.468795	-0.730560
56	1	0	-1.252300	-4.183737	-0.327334
57	1	0	-2.134315	-4.079026	1.190626
58	1	0	-6.686973	-2.321594	-0.281040
59	1	0	-6.168124	-1.498876	1.192251
60	8	0	2.049055	5.113570	-0.370554
61	1	0	1.730301	6.029778	-0.405107

022aaa_Rh_Julolidin_OH-H+_carb_B3LYP631dp_PCMw.log

Standard orientation:

Center Number	Atomic Number	Atomic Type	Coordinates (Angstroms) X	Y	Z
1	6	0	-2.192062	-2.277050	0.021222
2	6	0	-1.030677	-1.552546	0.008248
3	6	0	-1.012166	-0.130584	-0.027926
4	6	0	-2.280138	0.513809	-0.055938
5	6	0	-3.489969	-0.165753	-0.066427
6	6	0	-3.463223	-1.588046	-0.024174
7	6	0	0.170222	0.636581	0.006388
8	6	0	-1.232772	2.638510	-0.136197
9	6	0	0.054077	2.060474	-0.106024
10	6	0	1.171176	2.933701	-0.165362
11	1	0	2.172326	2.518111	-0.139173
12	6	0	0.992547	4.294436	-0.251270
13	6	0	-0.319757	4.847744	-0.279306
14	6	0	-1.428459	4.017059	-0.223699
15	1	0	-0.082759	-2.078754	0.041906
16	1	0	-2.429124	4.430837	-0.249637
17	8	0	-2.357803	1.874650	-0.088949
18	7	0	-4.624717	-2.299840	-0.038255
19	6	0	-4.649178	-3.764861	0.063745
20	1	0	-5.545753	-4.112068	-0.457403
21	1	0	-4.752478	-4.060351	1.118383
22	6	0	-5.922692	-1.625770	0.084652
23	8	0	-0.480937	6.183603	-0.365045
24	1	0	0.399698	6.594577	-0.392943
25	6	0	1.503239	-0.017098	-0.172441
26	6	0	2.326764	-0.305287	0.925129
27	6	0	1.940330	-0.331550	-1.468557
28	6	0	3.568223	-0.907754	0.717213
29	6	0	3.183155	-0.926282	-1.663352
30	1	0	1.307924	-0.107906	-2.322403
31	6	0	4.007748	-1.230921	-0.571018
32	1	0	4.173372	-1.085214	1.600793
33	1	0	3.531358	-1.163425	-2.662397
34	6	0	1.846392	0.048442	2.329426
35	8	0	2.599833	-0.214732	3.293395
36	8	0	0.698611	0.587858	2.364921
37	6	0	5.341120	-1.865450	-0.860995
38	8	0	5.863132	-1.782102	-1.979098
39	7	0	5.937878	-2.523327	0.164789
40	6	0	7.213815	-3.199874	-0.004637
41	1	0	7.635218	-3.413358	0.978552
42	1	0	7.108799	-4.141144	-0.557361
43	1	0	7.899169	-2.554866	-0.557596
44	1	0	5.411168	-2.704632	1.005576
45	6	0	-3.401642	-4.394634	-0.541799
46	1	0	-3.434672	-5.476608	-0.386007
47	1	0	-3.389302	-4.216234	-1.623093
48	6	0	-4.795814	0.597210	-0.125499
49	6	0	-5.922816	-0.291636	-0.651020
50	6	0	-2.160187	-3.786722	0.108675
51	1	0	-6.892402	0.196297	-0.516970
52	1	0	-5.791169	-0.473316	-1.723601
53	1	0	-5.052365	0.969811	0.875527
54	1	0	-4.679307	1.481434	-0.757978
55	1	0	-1.244052	-4.164139	-0.355185
56	1	0	-2.124691	-4.089954	1.164355
57	1	0	-6.680661	-2.294937	-0.330019
58	1	0	-6.163901	-1.478902	1.147792
59	8	0	1.985947	5.228284	-0.314667
60	1	0	2.857675	4.809550	-0.285661

022baa_Rh_Julolidin_OH-H+_carb_B3LYP631dp_PCMw_ring.log

Standard orientation:

Center Number	Atomic Number	Atomic Type	Coordinates (Angstroms) X	Y	Z
1	6	0	-2.231219	-2.234659	0.276381
2	6	0	-1.060189	-1.511155	0.427989
3	6	0	-1.001593	-0.114432	0.302443
4	6	0	-2.199852	0.539716	0.003113
5	6	0	-3.412996	-0.135299	-0.185009
6	6	0	-3.438098	-1.543110	-0.042322
7	6	0	0.272590	0.645277	0.555545
8	6	0	-1.119019	2.649373	-0.093921
9	6	0	0.131457	2.102906	0.196720
10	6	0	1.244498	2.968182	0.209799
11	1	0	2.230295	2.572220	0.435252
12	6	0	1.103836	4.317008	-0.058299
13	6	0	-0.172756	4.849327	-0.350635
14	6	0	-1.277708	4.013369	-0.371024
15	1	0	-0.149390	-2.053978	0.666805
16	1	0	-2.260002	4.409196	-0.601143
17	8	0	-2.269658	1.906274	-0.141932
18	7	0	-4.616819	-2.248192	-0.250660
19	6	0	-4.698878	-3.659081	0.119838
20	1	0	-5.551463	-4.091155	-0.413996
21	1	0	-4.903261	-3.774127	1.198303
22	6	0	-5.884241	-1.524545	-0.294873
23	8	0	-0.314648	6.174021	-0.618605
24	1	0	0.563975	6.583333	-0.561138
25	6	0	1.508895	-0.014524	-0.033371
26	6	0	2.369628	-0.404209	0.984667
27	6	0	1.845182	-0.243452	-1.365847
28	6	0	3.583137	-1.039235	0.730371
29	6	0	3.059856	-0.871442	-1.634164
30	1	0	1.187223	0.058269	-2.174229
31	6	0	3.927338	-1.284444	-0.603109
32	1	0	4.238043	-1.301218	1.555020
33	1	0	3.366212	-1.056312	-2.657762
34	6	0	1.770904	-0.022627	2.282010
35	8	0	2.208251	-0.181620	3.404121
36	8	0	0.576021	0.573174	2.039924
37	6	0	5.218727	-1.943150	-1.012347
38	8	0	5.709443	-1.746720	-2.129064
39	7	0	5.807276	-2.746057	-0.091221
40	6	0	7.044571	-3.454713	-0.380481
41	1	0	7.478566	-3.804805	0.556859
42	1	0	6.878688	-4.315306	-1.039183
43	1	0	7.745975	-2.778160	-0.871822
44	1	0	5.286000	-3.020463	0.727692
45	6	0	-3.412967	-4.394421	-0.244307
46	1	0	-3.500652	-5.448460	0.036603
47	1	0	-3.271334	-4.353000	-1.330670
48	6	0	-4.662666	0.649282	-0.531132
49	6	0	-5.745316	-0.253328	-1.125546
50	6	0	-2.230114	-3.738629	0.469597
51	1	0	-6.704621	0.272051	-1.161877
52	1	0	-5.483486	-0.529609	-2.153553
53	1	0	-5.049142	1.145123	0.370371
54	1	0	-4.412910	1.454340	-1.228657
55	1	0	-1.281599	-4.157897	0.116907
56	1	0	-2.287962	-3.971985	1.542292
57	1	0	-6.632126	-2.191064	-0.735585
58	1	0	-6.232741	-1.275095	0.722115
59	8	0	2.121657	5.235852	-0.071096
60	1	0	2.965955	4.812090	0.135275

023aaa_Rh_Julolidin_OH-H+oh_B3LYP631dp_PCMw.log

Standard orientation:

Center Number	Atomic Number	Atomic Type	Coordinates (Angstroms) X	Y	Z
1	6	0	-2.232030	-2.247131	-0.006191
2	6	0	-1.060996	-1.527473	-0.054006
3	6	0	-1.036671	-0.111971	-0.107664
4	6	0	-2.294649	0.536670	-0.112572
5	6	0	-3.511668	-0.135862	-0.086551
6	6	0	-3.493881	-1.555393	-0.033054
7	6	0	0.148061	0.675360	-0.145077
8	6	0	-1.240680	2.677027	-0.194626
9	6	0	0.064001	2.065993	-0.200251
10	6	0	1.202039	2.943788	-0.259386
11	1	0	2.201745	2.525687	-0.272142
12	6	0	1.023763	4.291969	-0.301450
13	6	0	-0.312835	4.917855	-0.291559
14	6	0	-1.434927	4.038729	-0.237562
15	1	0	-0.118635	-2.065370	-0.040507
16	1	0	-2.439103	4.447084	-0.232606
17	8	0	-2.360408	1.900792	-0.146275
18	7	0	-4.669271	-2.262790	-0.025099
19	6	0	-4.691423	-3.718544	0.140812
20	1	0	-5.609407	-4.087265	-0.326652
21	1	0	-4.747032	-3.978258	1.209744
22	6	0	-5.950780	-1.575511	0.147469
23	8	0	-0.353614	6.176509	-0.335088
24	6	0	1.481540	-0.002075	-0.219584
25	6	0	2.326399	-0.252759	0.884589
26	6	0	1.926140	-0.379691	-1.496511
27	6	0	3.572631	-0.863054	0.679836
28	6	0	3.163034	-0.985101	-1.684382
29	1	0	1.288505	-0.184023	-2.352362
30	6	0	3.999031	-1.244241	-0.591856
31	1	0	4.200216	-1.002714	1.552690
32	1	0	3.498633	-1.260736	-2.677806
33	6	0	2.014575	0.092003	2.306705
34	8	0	2.838498	0.050522	3.203796
35	8	0	0.732928	0.432844	2.524525
36	1	0	0.648821	0.636708	3.472575
37	6	0	5.334333	-1.884763	-0.867007
38	8	0	5.849049	-1.819169	-1.988252
39	7	0	5.931888	-2.519405	0.171067
40	6	0	7.210942	-3.194792	0.013694
41	1	0	7.637127	-3.379835	1.000438
42	1	0	7.106485	-4.150399	-0.513445
43	1	0	7.889634	-2.560482	-0.559054
44	1	0	5.403727	-2.695565	1.012249
45	6	0	-3.469893	-4.372568	-0.493831
46	1	0	-3.501953	-5.450694	-0.311585
47	1	0	-3.497294	-4.219720	-1.578912
48	6	0	-4.812649	0.637770	-0.116640
49	6	0	-5.964509	-0.245620	-0.597145
50	6	0	-2.200789	-3.757382	0.094765
51	1	0	-6.924816	0.252934	-0.436039
52	1	0	-5.869542	-0.436608	-1.672156
53	1	0	-5.036533	1.025593	0.886723
54	1	0	-4.707618	1.514177	-0.762300
55	1	0	-1.307121	-4.143284	-0.405620
56	1	0	-2.117892	-4.052500	1.150274
57	1	0	-6.733654	-2.237065	-0.233610
58	1	0	-6.154605	-1.415580	1.217916
59	8	0	2.037065	5.184532	-0.358462
60	1	0	1.555203	6.045638	-0.377337

024aaa_Rh_Julolidin_OH-2H_B3LYP631dp_PCMw.log

Standard orientation:

Center Atomic Atomic Coordinates (Angstroms)

Number	Number	Type	X	Y	Z
1	6	0	-2.122333	-2.262196	-0.022685
2	6	0	-0.976472	-1.499364	-0.025370
3	6	0	-0.999162	-0.084994	-0.055445
4	6	0	-2.276015	0.518022	-0.092165
5	6	0	-3.470693	-0.198893	-0.113426
6	6	0	-3.404871	-1.615496	-0.075685
7	6	0	0.166920	0.738672	-0.036483
8	6	0	-1.296619	2.691443	-0.132139
9	6	0	0.023343	2.128039	-0.104222
10	6	0	1.128905	3.047888	-0.122153
11	1	0	2.141195	2.661685	-0.094920
12	6	0	0.904670	4.389417	-0.170689
13	6	0	-0.449745	4.967060	-0.203330
14	6	0	-1.538419	4.048429	-0.182606
15	1	0	-0.014607	-2.000439	0.011097
16	1	0	-2.557086	4.419022	-0.208512
17	8	0	-2.393842	1.879297	-0.117036
18	7	0	-4.557990	-2.365882	-0.114948
19	6	0	-4.529474	-3.818870	0.060445
20	1	0	-5.426296	-4.224574	-0.417907
21	1	0	-4.589662	-4.078451	1.129802
22	6	0	-5.861688	-1.722976	0.049996
23	8	0	-0.533358	6.227147	-0.248864
24	6	0	1.519251	0.109050	-0.162501
25	6	0	2.284882	-0.324912	0.935896
26	6	0	2.029530	-0.051972	-1.462878
27	6	0	3.529819	-0.916904	0.698731
28	6	0	3.271370	-0.638520	-1.678491
29	1	0	1.441513	0.287535	-2.310638
30	6	0	4.034123	-1.091195	-0.593633
31	1	0	4.088444	-1.205739	1.583209
32	1	0	3.663847	-0.754455	-2.682760
33	6	0	1.808114	-0.157683	2.389399
34	8	0	2.593263	-0.564703	3.284421
35	8	0	0.674182	0.370127	2.539908
36	6	0	5.370604	-1.713584	-0.887852
37	8	0	5.949134	-1.524072	-1.965166
38	7	0	5.910422	-2.487958	0.088112
39	6	0	7.184407	-3.163988	-0.093743
40	1	0	7.549683	-3.499507	0.877828
41	1	0	7.097736	-4.031839	-0.758639
42	1	0	7.907570	-2.472340	-0.530304
43	1	0	5.342978	-2.744840	0.881145
44	6	0	-3.274776	-4.428320	-0.554211
45	1	0	-3.272156	-5.508005	-0.377498
46	1	0	-3.288807	-4.270886	-1.638999
47	6	0	-4.796125	0.530941	-0.176271
48	6	0	-5.907977	-0.387924	-0.684645
49	6	0	-2.038183	-3.772310	0.059495
50	1	0	-6.887766	0.076685	-0.539142
51	1	0	-5.785971	-0.567983	-1.758924
52	1	0	-5.057492	0.911882	0.820768
53	1	0	-4.704180	1.411104	-0.819213
54	1	0	-1.123976	-4.119339	-0.432673
55	1	0	-1.958414	-4.078563	1.112149
56	1	0	-6.619230	-2.405479	-0.345895
57	1	0	-6.084964	-1.576595	1.119116
58	8	0	1.888246	5.321449	-0.191540
59	1	0	1.369306	6.160894	-0.225073

031aaa_Rh_Julolidin_OMe_B3LYP631dp_PCMw.log

Standard orientation:

Center Number	Atomic Number	Atomic Type	Coordinates (Angstroms) X	Y	Z
1	6	0	-2.536634	-2.183769	-0.013869

2	6	0	-1.312085	-1.582236	-0.060146
3	6	0	-1.154846	-0.164070	-0.102868
4	6	0	-2.360268	0.606100	-0.101385
5	6	0	-3.627418	0.053289	-0.077315
6	6	0	-3.739076	-1.368066	-0.032491
7	6	0	0.080688	0.487695	-0.127832
8	6	0	-1.103805	2.617114	-0.167593
9	6	0	0.122527	1.909408	-0.172243
10	6	0	1.328447	2.664604	-0.217804
11	1	0	2.275070	2.141591	-0.227293
12	6	0	1.285220	4.038496	-0.251647
13	6	0	0.026729	4.721465	-0.244803
14	6	0	-1.161213	4.008565	-0.204298
15	1	0	-0.421459	-2.200622	-0.051572
16	1	0	-2.114218	4.522884	-0.201139
17	8	0	-2.292680	1.964959	-0.126785
18	7	0	-4.957636	-1.959653	-0.008601
19	6	0	-5.130159	-3.418712	0.076957
20	1	0	-6.062950	-3.664180	-0.437218
21	1	0	-5.253297	-3.706208	1.130738
22	6	0	-6.187456	-1.162716	0.122507
23	8	0	0.004510	6.063009	-0.280591
24	1	0	0.925196	6.378979	-0.305587
25	6	0	1.351205	-0.299426	-0.206218
26	6	0	2.162988	-0.635296	0.900177
27	6	0	1.759412	-0.704099	-1.486512
28	6	0	3.340462	-1.367264	0.693699
29	6	0	2.933914	-1.424110	-1.674289
30	1	0	1.145956	-0.444198	-2.342743
31	6	0	3.732299	-1.776526	-0.580406
32	1	0	3.945137	-1.581131	1.567568
33	1	0	3.245491	-1.721600	-2.669142
34	6	0	1.876685	-0.270788	2.322970
35	8	0	2.638556	-0.504959	3.243403
36	8	0	0.691108	0.336227	2.503540
37	1	0	0.606561	0.532759	3.453120
38	6	0	4.997469	-2.548569	-0.854043
39	8	0	5.533043	-2.509251	-1.966143
40	7	0	5.504454	-3.267738	0.176649
41	6	0	6.705319	-4.075274	0.022963
42	1	0	7.104193	-4.308494	1.010968
43	1	0	6.502133	-5.011948	-0.509163
44	1	0	7.449520	-3.513390	-0.543612
45	1	0	4.942713	-3.408777	1.002824
46	6	0	-3.961241	-4.167365	-0.547384
47	1	0	-4.099685	-5.240262	-0.388840
48	1	0	-3.947325	-3.991708	-1.628880
49	6	0	-4.852946	0.939536	-0.098777
50	6	0	-6.068486	0.167392	-0.609344
51	6	0	-2.656359	-3.686702	0.083959
52	1	0	-6.984008	0.745044	-0.455798
53	1	0	-5.973320	-0.019577	-1.684656
54	1	0	-5.050369	1.320056	0.912353
55	1	0	-4.664863	1.816023	-0.724291
56	1	0	-1.788620	-4.157499	-0.386588
57	1	0	-2.639020	-3.981384	1.142384
58	1	0	-7.007104	-1.757297	-0.286851
59	1	0	-6.400097	-1.001021	1.188555
60	8	0	2.342943	4.888438	-0.294732
61	6	0	3.664319	4.336151	-0.301793
62	1	0	3.843071	3.749751	0.605038
63	1	0	3.817353	3.709891	-1.186411
64	1	0	4.342240	5.187703	-0.330657

032aaa_Rh_Julolidin_OMe+H+carb_B3LYP631dp_PCMw.log

Standard orientation:

Center Number	Atomic Number	Atomic Type	Coordinates (Angstroms) X	Y	Z

1	6	0	-2.525900	-2.175492	0.007906
2	6	0	-1.298069	-1.570006	-0.001606
3	6	0	-1.138353	-0.156858	-0.029939
4	6	0	-2.335667	0.610512	-0.056537
5	6	0	-3.607312	0.055152	-0.070778
6	6	0	-3.722177	-1.362751	-0.033816
7	6	0	0.114182	0.489265	0.009326
8	6	0	-1.080879	2.619997	-0.121626
9	6	0	0.141530	1.917335	-0.090765
10	6	0	1.344635	2.672657	-0.136760
11	1	0	2.289382	2.146745	-0.106832
12	6	0	1.303325	4.046067	-0.212939
13	6	0	0.047838	4.726806	-0.244292
14	6	0	-1.137998	4.012396	-0.200186
15	1	0	-0.406820	-2.187459	0.028602
16	1	0	-2.092078	4.524401	-0.228217
17	8	0	-2.276252	1.972585	-0.083736
18	7	0	-4.949126	-1.955537	-0.049307
19	6	0	-5.118360	-3.410754	0.052490
20	1	0	-6.047122	-3.667200	-0.464835
21	1	0	-5.245572	-3.695415	1.107550
22	6	0	-6.173604	-1.155569	0.074914
23	8	0	0.024589	6.071365	-0.321963
24	6	0	1.375056	-0.295386	-0.165936
25	6	0	2.159864	-0.675781	0.932444
26	6	0	1.782741	-0.644553	-1.463023
27	6	0	3.330641	-1.405656	0.722604
28	6	0	2.955711	-1.367103	-1.658923
29	1	0	1.180940	-0.348973	-2.317137
30	6	0	3.737843	-1.766631	-0.566071
31	1	0	3.909883	-1.652737	1.606808
32	1	0	3.280330	-1.632513	-2.658828
33	6	0	1.720428	-0.283659	2.340784
34	8	0	2.448657	-0.628067	3.299199
35	8	0	0.632984	0.366854	2.385361
36	6	0	4.994029	-2.543533	-0.854119
37	8	0	5.526814	-2.514471	-1.969800
38	7	0	5.508364	-3.267280	0.172300
39	6	0	6.699637	-4.084865	0.008818
40	1	0	7.108810	-4.317793	0.992940
41	1	0	6.484847	-5.023131	-0.516740
42	1	0	7.442722	-3.532612	-0.568948
43	1	0	4.958078	-3.391661	1.008315
44	6	0	-3.941834	-4.160446	-0.558496
45	1	0	-4.082088	-5.234095	-0.404671
46	1	0	-3.915140	-3.981931	-1.639512
47	6	0	-4.830515	0.944548	-0.126285
48	6	0	-6.039937	0.174593	-0.655797
49	6	0	-2.643935	-3.681081	0.089166
50	1	0	-6.956230	0.755932	-0.519883
51	1	0	-5.926521	-0.014901	-1.729115
52	1	0	-5.049168	1.336079	0.876499
53	1	0	-4.626515	1.815831	-0.754618
54	1	0	-1.771487	-4.145895	-0.379580
55	1	0	-2.634622	-3.990280	1.143662
56	1	0	-6.994232	-1.743906	-0.343079
57	1	0	-6.400152	-0.988980	1.138349
58	8	0	2.366749	4.894925	-0.263815
59	6	0	3.683679	4.337187	-0.234835
60	1	0	3.848813	3.777979	0.691889
61	1	0	3.847538	3.681678	-1.096403
62	1	0	4.367586	5.183548	-0.280993
63	1	0	0.946222	6.383144	-0.342456

032baa_Rh_Julolidin_OMe+H+carb_B3LYP631dp_PCMw_ring.log

Standard orientation:

Center Atomic Atomic Coordinates (Angstroms)

Number	Number	Type	X	Y	Z
1	6	0	-2.603838	-2.085724	0.281114
2	6	0	-1.355534	-1.504454	0.426896
3	6	0	-1.133587	-0.124951	0.295201
4	6	0	-2.247092	0.664065	-0.005837
5	6	0	-3.532033	0.135742	-0.187737
6	6	0	-3.722086	-1.258544	-0.036822
7	6	0	0.221532	0.480643	0.543191
8	6	0	-0.923488	2.629718	-0.116518
9	6	0	0.253169	1.942670	0.177216
10	6	0	1.464509	2.668032	0.188504
11	1	0	2.386613	2.148447	0.418333
12	6	0	1.483831	4.023709	-0.085910
13	6	0	0.274120	4.700626	-0.382495
14	6	0	-0.920798	4.002636	-0.400496
15	1	0	-0.514833	-2.150268	0.666497
16	1	0	-1.849645	4.510656	-0.632213
17	8	0	-2.154313	2.028522	-0.159720
18	7	0	-4.977061	-1.821085	-0.235689
19	6	0	-5.222310	-3.209688	0.146742
20	1	0	-6.124184	-3.541741	-0.377524
21	1	0	-5.429782	-3.291903	1.227614
22	6	0	-6.149781	-0.952206	-0.272259
23	8	0	0.293414	6.030817	-0.654842
24	6	0	1.369758	-0.324764	-0.042785
25	6	0	2.179015	-0.809800	0.976521
26	6	0	1.675507	-0.597238	-1.374439
27	6	0	3.307903	-1.586193	0.724316
28	6	0	2.806332	-1.366620	-1.640647
29	1	0	1.057667	-0.221857	-2.183623
30	6	0	3.619303	-1.876112	-0.608361
31	1	0	3.927079	-1.922012	1.549865
32	1	0	3.087698	-1.590669	-2.663582
33	6	0	1.631563	-0.353394	2.272502
34	8	0	2.048290	-0.558290	3.395096
35	8	0	0.516292	0.379609	2.028272
36	6	0	4.821521	-2.686803	-1.017165
37	8	0	5.329986	-2.553310	-2.135264
38	7	0	5.311019	-3.552982	-0.095307
39	6	0	6.451010	-4.408622	-0.387427
40	1	0	6.853171	-4.794892	0.549975
41	1	0	6.175014	-5.252687	-1.030471
42	1	0	7.220583	-3.827094	-0.897745
43	1	0	4.761773	-3.761809	0.724804
44	6	0	-4.035140	-4.094138	-0.220696
45	1	0	-4.244587	-5.128787	0.067766
46	1	0	-3.897345	-4.076310	-1.308224
47	6	0	-4.681621	1.060393	-0.535879
48	6	0	-5.869318	0.287947	-1.113816
49	6	0	-2.778312	-3.578644	0.481687
50	1	0	-6.759813	0.923207	-1.146567
51	1	0	-5.651235	-0.024678	-2.141613
52	1	0	-4.999138	1.608269	0.362418
53	1	0	-4.342023	1.822672	-1.243564
54	1	0	-1.888104	-4.107856	0.124775
55	1	0	-2.855343	-3.798946	1.555899
56	1	0	-6.976353	-1.527486	-0.700696
57	1	0	-6.454949	-0.656749	0.746335
58	8	0	2.584022	4.838000	-0.108353
59	6	0	3.857801	4.261356	0.177707
60	1	0	3.879767	3.836864	1.187496
61	1	0	4.110372	3.483632	-0.551485
62	1	0	4.579253	5.074982	0.108055
63	1	0	1.217491	6.324842	-0.590887

033aaa_Rh_Julolidin_OMe+H+oh_B3LYP631dp_PCMw.log

Standard orientation:

Center Number	Atomic Number	Atomic Type	Coordinates (Angstroms) X	Y	Z
1	6	0	-2.449187	-2.164968	-0.101298
2	6	0	-1.234536	-1.516186	-0.118792
3	6	0	-1.121855	-0.105755	-0.119493
4	6	0	-2.335610	0.619747	-0.118828
5	6	0	-3.592045	0.022484	-0.121078
6	6	0	-3.663342	-1.396268	-0.107936
7	6	0	0.114185	0.609525	-0.120941
8	6	0	-1.150141	2.692766	-0.137071
9	6	0	0.112025	1.999074	-0.142240
10	6	0	1.301080	2.810680	-0.150061
11	1	0	2.256194	2.302104	-0.148440
12	6	0	1.232433	4.173476	-0.160362
13	6	0	-0.068388	4.895406	-0.161412
14	6	0	-1.241524	4.058432	-0.147828
15	1	0	-0.327886	-2.112656	-0.123981
16	1	0	-2.213426	4.539352	-0.145900
17	8	0	-2.319839	1.987592	-0.118608
18	7	0	-4.883245	-2.028814	-0.124634
19	6	0	-4.991711	-3.482114	0.019687
20	1	0	-5.936328	-3.787744	-0.440199
21	1	0	-5.047548	-3.757465	1.084974
22	6	0	-6.117224	-1.266189	0.071491
23	8	0	-0.112996	6.141223	-0.172941
24	6	0	1.402335	-0.150609	-0.194800
25	6	0	2.084713	-0.705760	0.911726
26	6	0	1.951970	-0.333686	-1.474060
27	6	0	3.265834	-1.433101	0.702521
28	6	0	3.131066	-1.044663	-1.664360
29	1	0	1.437230	0.091115	-2.329554
30	6	0	3.794400	-1.619581	-0.574064
31	1	0	3.765912	-1.821776	1.582237
32	1	0	3.549056	-1.166399	-2.657394
33	6	0	1.666909	-0.579118	2.343262
34	8	0	2.246621	-1.131999	3.262161
35	8	0	0.596766	0.208130	2.543756
36	1	0	0.420766	0.210435	3.501093
37	6	0	5.068333	-2.376617	-0.843176
38	8	0	5.736699	-2.154291	-1.858053
39	7	0	5.433198	-3.296925	0.083248
40	6	0	6.627099	-4.111956	-0.081224
41	1	0	6.893214	-4.548452	0.882311
42	1	0	6.474614	-4.919217	-0.807282
43	1	0	7.448631	-3.485714	-0.432832
44	1	0	4.771145	-3.566013	0.795277
45	6	0	-3.819736	-4.196295	-0.643602
46	1	0	-3.917661	-5.274680	-0.487720
47	1	0	-3.848272	-4.014703	-1.724241
48	6	0	-4.841739	0.877009	-0.137520
49	6	0	-6.046273	0.080282	-0.639548
50	6	0	-2.508356	-3.677520	-0.054352
51	1	0	-6.973433	0.634340	-0.465758
52	1	0	-5.962457	-0.089582	-1.719064
53	1	0	-5.044040	1.256950	0.873467
54	1	0	-4.678767	1.758985	-0.763378
55	1	0	-1.646666	-4.098464	-0.582224
56	1	0	-2.427728	-4.014935	0.988590
57	1	0	-6.942317	-1.865837	-0.323139
58	1	0	-6.308598	-1.119344	1.146411
59	8	0	2.297084	5.014230	-0.168747
60	6	0	3.600244	4.437034	-0.169040
61	1	0	3.766819	3.829431	0.728280
62	1	0	3.760473	3.816004	-1.058255
63	1	0	4.300962	5.272195	-0.177749

034aaa_Rh_Julolidin_OMe-2H+_B3LYP631dp_PCMw.log

Standard orientation:

Center Number	Atomic Number	Atomic Type	Coordinates (Angstroms) X	Y	Z
1	6	0	-2.475982	-2.156285	-0.053028
2	6	0	-1.254870	-1.517150	-0.052656
3	6	0	-1.128048	-0.109546	-0.064650
4	6	0	-2.332812	0.625841	-0.088457
5	6	0	-3.596467	0.038718	-0.107810
6	6	0	-3.681260	-1.377403	-0.086607
7	6	0	0.122227	0.587895	-0.044908
8	6	0	-1.128092	2.684354	-0.120018
9	6	0	0.122652	1.980195	-0.106099
10	6	0	1.316897	2.783026	-0.125258
11	1	0	2.266200	2.263770	-0.106682
12	6	0	1.261473	4.146450	-0.162254
13	6	0	-0.029215	4.880221	-0.183286
14	6	0	-1.207177	4.053124	-0.159154
15	1	0	-0.351093	-2.117553	-0.030906
16	1	0	-2.175742	4.541076	-0.174853
17	8	0	-2.308341	1.993922	-0.098921
18	7	0	-4.910126	-2.000494	-0.125515
19	6	0	-5.030317	-3.448028	0.051517
20	1	0	-5.974314	-3.757789	-0.407460
21	1	0	-5.093937	-3.702276	1.122191
22	6	0	-6.134975	-1.223896	0.064383
23	8	0	-0.063431	6.128805	-0.221280
24	6	0	1.400084	-0.183017	-0.163458
25	6	0	2.109253	-0.695409	0.939914
26	6	0	1.898127	-0.399303	-1.460815
27	6	0	3.284552	-1.417975	0.708677
28	6	0	3.071092	-1.115731	-1.670014
29	1	0	1.354096	0.000559	-2.311675
30	6	0	3.774120	-1.647215	-0.580799
31	1	0	3.804006	-1.764794	1.596333
32	1	0	3.453957	-1.273772	-2.672280
33	6	0	1.651257	-0.478064	2.393982
34	8	0	2.390511	-0.965753	3.288264
35	8	0	0.580013	0.166363	2.545644
36	6	0	5.035996	-2.412893	-0.866143
37	8	0	5.643479	-2.284582	-1.936517
38	7	0	5.474290	-3.247524	0.111103
39	6	0	6.662949	-4.066319	-0.061241
40	1	0	6.969064	-4.455041	0.910943
41	1	0	6.486117	-4.909368	-0.740085
42	1	0	7.470709	-3.459995	-0.475930
43	1	0	4.870680	-3.442264	0.895044
44	6	0	-3.858633	-4.183657	-0.589081
45	1	0	-3.968076	-5.258952	-0.419094
46	1	0	-3.875181	-4.017196	-1.672524
47	6	0	-4.837229	0.905889	-0.149896
48	6	0	-6.045900	0.117190	-0.655248
49	6	0	-2.548107	-3.668507	0.005786
50	1	0	-6.968930	0.681858	-0.493143
51	1	0	-5.955445	-0.061830	-1.732845
52	1	0	-5.046996	1.301743	0.853574
53	1	0	-4.657837	1.778454	-0.784709
54	1	0	-1.685746	-4.100874	-0.512023
55	1	0	-2.477711	-3.998997	1.051785
56	1	0	-6.966587	-1.816043	-0.328687
57	1	0	-6.328955	-1.066324	1.137894
58	8	0	2.339362	4.977062	-0.181682
59	6	0	3.632622	4.382304	-0.162432
60	1	0	3.787194	3.789844	0.747514
61	1	0	3.790842	3.740061	-1.037307
62	1	0	4.346495	5.206442	-0.183916

034bba_Rh_Julolidin_OMe-H+carb_B3LYP631dp_PCMw_ring.log

Standard orientation:

Center Number	Atomic Number	Atomic Type	Coordinates (Angstroms) X	Y	Z
1	6	0	-2.561330	-2.098558	0.226075
2	6	0	-1.322259	-1.494700	0.368033
3	6	0	-1.127533	-0.108818	0.265766
4	6	0	-2.261214	0.669905	0.004575
5	6	0	-3.538494	0.114454	-0.170290
6	6	0	-3.698482	-1.285641	-0.054061
7	6	0	0.212680	0.524908	0.503098
8	6	0	-0.964570	2.663778	-0.097419
9	6	0	0.222052	1.966289	0.144700
10	6	0	1.424786	2.729128	0.125437
11	1	0	2.357333	2.210660	0.318638
12	6	0	1.427392	4.083204	-0.123931
13	6	0	0.193734	4.833033	-0.382343
14	6	0	-0.989803	4.030322	-0.357407
15	1	0	-0.465485	-2.129212	0.580294
16	1	0	-1.942749	4.512679	-0.554975
17	8	0	-2.198590	2.032276	-0.111853
18	7	0	-4.948003	-1.869982	-0.254414
19	6	0	-5.159312	-3.265936	0.118772
20	1	0	-6.068234	-3.608866	-0.386376
21	1	0	-5.337858	-3.365728	1.203927
22	6	0	-6.135181	-1.021472	-0.231834
23	8	0	0.202401	6.079671	-0.613963
24	6	0	1.381943	-0.273533	-0.045353
25	6	0	2.182646	-0.757440	0.981425
26	6	0	1.714982	-0.534541	-1.373784
27	6	0	3.324394	-1.517555	0.739289
28	6	0	2.856856	-1.290152	-1.630583
29	1	0	1.105722	-0.156684	-2.188568
30	6	0	3.660982	-1.796797	-0.590074
31	1	0	3.935034	-1.849765	1.572891
32	1	0	3.154930	-1.504104	-2.651068
33	6	0	1.615299	-0.320116	2.279136
34	8	0	2.045255	-0.543922	3.398915
35	8	0	0.502607	0.393457	2.044116
36	6	0	4.877092	-2.591473	-0.984549
37	8	0	5.395256	-2.458468	-2.098787
38	7	0	5.372017	-3.446939	-0.054437
39	6	0	6.525715	-4.287724	-0.334133
40	1	0	6.920077	-4.672433	0.607262
41	1	0	6.269136	-5.133102	-0.983661
42	1	0	7.295590	-3.695902	-0.832272
43	1	0	4.818901	-3.660022	0.761861
44	6	0	-3.965919	-4.124369	-0.288818
45	1	0	-4.151684	-5.167486	-0.014311
46	1	0	-3.852828	-4.085348	-1.378732
47	6	0	-4.711232	1.025033	-0.475761
48	6	0	-5.898392	0.246049	-1.045993
49	6	0	-2.702473	-3.599313	0.394691
50	1	0	-6.801266	0.864797	-1.042004
51	1	0	-5.699066	-0.036461	-2.086385
52	1	0	-5.019735	1.550044	0.439476
53	1	0	-4.397887	1.808217	-1.172878
54	1	0	-1.811617	-4.103148	0.003592
55	1	0	-2.748354	-3.848029	1.464709
56	1	0	-6.964896	-1.598533	-0.652384
57	1	0	-6.416382	-0.758157	0.802977
58	6	0	3.805356	4.227461	0.078060
59	1	0	3.849715	3.774844	1.078206
60	1	0	4.012898	3.446129	-0.665985
61	1	0	4.570880	5.002512	0.003889
62	8	0	2.563251	4.865611	-0.158742

041aaa_Rh_Julolidin_F2_B3LYP631dp_PCMw.log

Standard orientation:

Center Number	Atomic Number	Atomic Type	Coordinates (Angstroms) X	Y	Z
1	6	0	-2.080529	-2.516624	0.058985
2	6	0	-0.940214	-1.772856	-0.000703
3	6	0	-0.955466	-0.344408	-0.074851
4	6	0	-2.250888	0.273760	-0.078783
5	6	0	-3.438126	-0.425200	-0.041096
6	6	0	-3.375539	-1.852727	0.023005
7	6	0	0.190425	0.444022	-0.123760
8	6	0	-1.246738	2.409176	-0.192317
9	6	0	0.059132	1.867051	-0.199195
10	6	0	1.155570	2.760455	-0.283663
11	1	0	2.174261	2.395314	-0.300799
12	6	0	0.917109	4.107201	-0.348957
13	6	0	-0.381974	4.663683	-0.338851
14	6	0	-1.458963	3.785773	-0.260788
15	1	0	0.018630	-2.277979	0.021186
16	8	0	-2.351616	1.632078	-0.123435
17	7	0	-4.508352	-2.584395	0.054130
18	6	0	-4.507535	-4.055865	0.142973
19	1	0	-5.394671	-4.409542	-0.387873
20	1	0	-4.618817	-4.346922	1.196409
21	6	0	-5.831188	-1.944442	0.160808
22	8	0	-0.603306	5.984949	-0.406074
23	1	0	0.249469	6.446766	-0.460073
24	6	0	1.547719	-0.179534	-0.197440
25	6	0	2.430469	-0.317478	0.897211
26	6	0	1.961330	-0.637619	-1.457750
27	6	0	3.694201	-0.888073	0.694209
28	6	0	3.214904	-1.211642	-1.638630
29	1	0	1.288898	-0.538225	-2.303197
30	6	0	4.103395	-1.328515	-0.564315
31	1	0	4.327857	-0.999574	1.566565
32	1	0	3.519050	-1.575424	-2.613495
33	6	0	2.122936	0.074081	2.308408
34	8	0	2.922798	-0.018125	3.221286
35	8	0	0.872290	0.533745	2.486246
36	1	0	0.776561	0.763751	3.427182
37	6	0	5.440866	-1.972824	-0.826899
38	8	0	5.598080	-2.730780	-1.789073
39	7	0	6.436850	-1.681003	0.043744
40	6	0	7.777109	-2.222516	-0.124983
41	1	0	8.319166	-1.723408	-0.936564
42	1	0	8.330675	-2.089829	0.805221
43	1	0	7.714017	-3.286683	-0.358585
44	1	0	6.310251	-0.929684	0.704785
45	6	0	-3.246561	-4.664343	-0.452667
46	1	0	-3.257011	-5.743503	-0.278545
47	1	0	-3.236199	-4.503811	-1.536405
48	6	0	-4.763199	0.302990	-0.069229
49	6	0	-5.870332	-0.616418	-0.582257
50	6	0	-2.020363	-4.019726	0.189859
51	1	0	-6.851083	-0.155008	-0.439513
52	1	0	-5.744314	-0.798524	-1.655081
53	1	0	-5.009275	0.655609	0.941221
54	1	0	-4.681927	1.195525	-0.694641
55	1	0	-1.093490	-4.391830	-0.255004
56	1	0	-1.989355	-4.286745	1.255238
57	1	0	-6.563442	-2.641614	-0.251251
58	1	0	-6.072049	-1.804297	1.223319
59	9	0	-2.708847	4.268823	-0.254122
60	9	0	1.930183	4.996088	-0.432187

042aaa_Rh_Julolidin_F2-H+carb_B3LYP631dp_PCMw.log

Standard orientation:

Center Atomic Atomic Coordinates (Angstroms)

Number	Number	Type	X	Y	Z
1	6	0	-1.930367	-2.558024	0.222565
2	6	0	-0.823435	-1.740455	0.377211
3	6	0	-0.891893	-0.340244	0.309378
4	6	0	-2.151894	0.211897	0.066440
5	6	0	-3.306899	-0.555735	-0.116838
6	6	0	-3.202965	-1.965717	-0.034564
7	6	0	0.313346	0.521912	0.574304
8	6	0	-1.260563	2.398262	-0.010542
9	6	0	0.047834	1.968173	0.228542
10	6	0	1.082271	2.919743	0.209114
11	1	0	2.110694	2.632411	0.393390
12	6	0	0.792176	4.240624	-0.047572
13	6	0	-0.511297	4.697076	-0.293492
14	6	0	-1.526725	3.748624	-0.274225
15	1	0	0.137314	-2.209747	0.570834
16	8	0	-2.345035	1.576625	-0.023725
17	7	0	-4.319413	-2.762428	-0.242522
18	6	0	-4.266092	-4.192794	0.052913
19	1	0	-5.088413	-4.671818	-0.488157
20	1	0	-4.435446	-4.380296	1.126989
21	6	0	-5.647619	-2.155348	-0.230094
22	8	0	-0.798684	5.993752	-0.553454
23	6	0	1.603315	-0.018349	-0.020481
24	6	0	2.493897	-0.335527	0.996977
25	6	0	1.960102	-0.205226	-1.353958
26	6	0	3.760655	-0.855209	0.740727
27	6	0	3.228037	-0.716724	-1.623793
28	1	0	1.278568	0.040285	-2.161701
29	6	0	4.127897	-1.056343	-0.593922
30	1	0	4.434880	-1.062985	1.565095
31	1	0	3.552257	-0.864409	-2.647900
32	6	0	1.861037	-0.020723	2.295564
33	8	0	2.304891	-0.148675	3.417950
34	8	0	0.615989	0.468740	2.053383
35	6	0	5.475397	-1.589436	-1.006858
36	8	0	5.944254	-1.340669	-2.122365
37	7	0	6.136533	-2.339850	-0.091172
38	6	0	7.432592	-2.931747	-0.386640
39	1	0	7.895262	-3.252696	0.547376
40	1	0	7.344065	-3.795783	-1.055562
41	1	0	8.071303	-2.189675	-0.868882
42	1	0	5.643763	-2.665659	0.726475
43	6	0	-2.928138	-4.789017	-0.372639
44	1	0	-2.913413	-5.858642	-0.142189
45	1	0	-2.816798	-4.683735	-1.458203
46	6	0	-4.630865	0.127823	-0.393815
47	6	0	-5.644247	-0.841848	-1.004778
48	6	0	-1.792322	-4.062466	0.348998
49	1	0	-6.646973	-0.403841	-0.994662
50	1	0	-5.386521	-1.047557	-2.050155
51	1	0	-5.033520	0.541889	0.541186
52	1	0	-4.474559	0.985260	-1.055132
53	1	0	-0.818494	-4.380287	-0.038607
54	1	0	-1.806929	-4.345019	1.411226
55	1	0	-6.342094	-2.867734	-0.685886
56	1	0	-5.992608	-1.983971	0.803807
57	9	0	-2.793339	4.136110	-0.513840
58	9	0	1.769690	5.182183	-0.074883
59	1	0	0.029926	6.497931	-0.530401

042baa_Rh_Julolidin_F2-H+carb_B3LYP631dp_PCMw_ring.log

Standard orientation:

Center Number	Atomic Number	Atomic Type	Coordinates (Angstroms) X	Y	Z
1	6	0	-1.930592	-2.557968	0.222228

2	6	0	-0.823576	-1.740505	0.376782
3	6	0	-0.891912	-0.340278	0.309055
4	6	0	-2.151889	0.211967	0.066296
5	6	0	-3.306996	-0.555536	-0.116874
6	6	0	-3.203201	-1.965531	-0.034604
7	6	0	0.313391	0.521768	0.574225
8	6	0	-1.260378	2.398275	-0.010649
9	6	0	0.047976	1.968098	0.228451
10	6	0	1.082480	2.919602	0.209161
11	1	0	2.110877	2.632184	0.393446
12	6	0	0.792487	4.240511	-0.047467
13	6	0	-0.510955	4.697078	-0.293386
14	6	0	-1.526426	3.748684	-0.274285
15	1	0	0.137166	-2.209889	0.570232
16	8	0	-2.344915	1.576731	-0.023987
17	7	0	-4.319792	-2.762105	-0.242108
18	6	0	-4.266506	-4.192509	0.053077
19	1	0	-5.089032	-4.671389	-0.487805
20	1	0	-4.435528	-4.380198	1.127176
21	6	0	-5.647905	-2.154856	-0.229590
22	8	0	-0.798224	5.993803	-0.553254
23	6	0	1.603367	-0.018518	-0.020542
24	6	0	2.493828	-0.335836	0.996992
25	6	0	1.960258	-0.205291	-1.353995
26	6	0	3.760595	-0.855525	0.740803
27	6	0	3.228202	-0.716816	-1.623757
28	1	0	1.278806	0.040325	-2.161775
29	6	0	4.127966	-1.056518	-0.593830
30	1	0	4.434730	-1.063417	1.565218
31	1	0	3.552530	-0.864435	-2.647838
32	6	0	1.860854	-0.021109	2.295529
33	8	0	2.304506	-0.149152	3.417967
34	8	0	0.615833	0.468485	2.053198
35	6	0	5.475477	-1.589622	-1.006715
36	8	0	5.944186	-1.341212	-2.122368
37	7	0	6.136829	-2.339616	-0.090845
38	6	0	7.432841	-2.931588	-0.386431
39	1	0	7.895324	-3.252993	0.547516
40	1	0	7.344237	-3.795327	-1.055724
41	1	0	8.071759	-2.189441	-0.868293
42	1	0	5.644215	-2.665295	0.726949
43	6	0	-2.928732	-4.788786	-0.372977
44	1	0	-2.914032	-5.858447	-0.142692
45	1	0	-2.817710	-4.683342	-1.458558
46	6	0	-4.630894	0.128168	-0.393819
47	6	0	-5.644491	-0.841471	-1.004472
48	6	0	-1.792646	-4.062442	0.348435
49	1	0	-6.647165	-0.403343	-0.994251
50	1	0	-5.386966	-1.047366	-2.049862
51	1	0	-5.033374	0.542539	0.541119
52	1	0	-4.474524	0.985417	-1.055371
53	1	0	-0.818967	-4.380291	-0.039525
54	1	0	-1.806949	-4.345158	1.410623
55	1	0	-6.342551	-2.867211	-0.685168
56	1	0	-5.992745	-1.983255	0.804326
57	9	0	-2.793013	4.136262	-0.513935
58	9	0	1.770056	5.182022	-0.074654
59	1	0	0.030421	6.497923	-0.530087

043aaa_Rh_Julolidin_F2-H+oh_B3LYP631dp_PCMw.log

Standard orientation:

Center Number	Atomic Number	Atomic Type	Coordinates (Angstroms) X	Y	Z
1	6	0	-1.855654	-2.554011	-0.073778
2	6	0	-0.773366	-1.707448	-0.101299
3	6	0	-0.905577	-0.296407	-0.110497
4	6	0	-2.230038	0.210524	-0.104221

5	6	0	-3.362349	-0.591556	-0.096726
6	6	0	-3.187140	-2.003936	-0.079013
7	6	0	0.187768	0.611914	-0.121344
8	6	0	-1.414671	2.442328	-0.137354
9	6	0	-0.052030	1.985175	-0.148234
10	6	0	0.974309	2.986950	-0.171517
11	1	0	2.017697	2.695479	-0.176368
12	6	0	0.643929	4.301875	-0.189921
13	6	0	-0.726466	4.822820	-0.185777
14	6	0	-1.721841	3.778842	-0.157044
15	1	0	0.223551	-2.135732	-0.107167
16	8	0	-2.452372	1.560832	-0.106725
17	7	0	-4.274656	-2.833773	-0.082855
18	6	0	-4.137300	-4.288909	0.035927
19	1	0	-5.012510	-4.739385	-0.441044
20	1	0	-4.159489	-4.583045	1.096515
21	6	0	-5.628700	-2.298104	0.085900
22	8	0	-0.992000	6.040310	-0.205955
23	6	0	1.589315	0.090708	-0.202475
24	6	0	2.384868	-0.277999	0.905741
25	6	0	2.137603	-0.034507	-1.488598
26	6	0	3.682202	-0.766721	0.692688
27	6	0	3.429010	-0.510836	-1.682752
28	1	0	1.535616	0.249842	-2.345273
29	6	0	4.213391	-0.897173	-0.589833
30	1	0	4.264780	-1.011322	1.573442
31	1	0	3.844631	-0.589135	-2.681061
32	6	0	1.971727	-0.180475	2.340848
33	8	0	2.682339	-0.525755	3.268641
34	8	0	0.742055	0.327159	2.529895
35	1	0	0.582463	0.341453	3.489957
36	6	0	5.608840	-1.392797	-0.866792
37	8	0	6.191758	-1.090803	-1.913197
38	7	0	6.178851	-2.169531	0.086721
39	6	0	7.511650	-2.727898	-0.083643
40	1	0	7.884060	-3.057429	0.887063
41	1	0	7.512585	-3.580220	-0.773189
42	1	0	8.177910	-1.962332	-0.484681
43	1	0	5.607315	-2.530389	0.835578
44	6	0	-2.855612	-4.786221	-0.621160
45	1	0	-2.768909	-5.866087	-0.470283
46	1	0	-2.906317	-4.606174	-1.701135
47	6	0	-4.741341	0.032326	-0.107155
48	6	0	-5.783722	-0.958837	-0.624477
49	6	0	-1.657771	-4.053409	-0.018441
50	1	0	-6.794829	-0.575434	-0.460301
51	1	0	-5.659337	-1.108268	-1.702995
52	1	0	-5.008739	0.354968	0.908482
53	1	0	-4.733736	0.938150	-0.719660
54	1	0	-0.730669	-4.323632	-0.533385
55	1	0	-1.535785	-4.367698	1.027588
56	1	0	-6.326709	-3.033300	-0.323472
57	1	0	-5.859519	-2.191996	1.156925
58	9	0	-3.020889	4.158773	-0.149034
59	9	0	1.613793	5.240513	-0.212988

044aaa_Rh_Julolidin_F2-2H+_B3LYP631dp_PCMw.log

Standard orientation:

Center Number	Atomic Number	Atomic Type	Coordinates (Angstroms) X	Y	Z
1	6	0	-1.848968	-2.554682	-0.024395
2	6	0	-0.766330	-1.705390	-0.028381
3	6	0	-0.897655	-0.296585	-0.045797
4	6	0	-2.219040	0.208194	-0.067250
5	6	0	-3.352959	-0.596644	-0.084527
6	6	0	-3.178167	-2.006643	-0.061038
7	6	0	0.205451	0.608562	-0.028292

8	6	0	-1.406320	2.437852	-0.108399
9	6	0	-0.047381	1.982961	-0.099761
10	6	0	0.973842	2.986501	-0.134423
11	1	0	2.017031	2.694174	-0.122089
12	6	0	0.642296	4.301906	-0.181926
13	6	0	-0.725312	4.821824	-0.197618
14	6	0	-1.715306	3.775896	-0.157258
15	1	0	0.232146	-2.129444	-0.005065
16	8	0	-2.447256	1.558388	-0.077437
17	7	0	-4.268744	-2.839775	-0.094629
18	6	0	-4.129964	-4.290445	0.053137
19	1	0	-4.996001	-4.753087	-0.429664
20	1	0	-4.166686	-4.570023	1.117952
21	6	0	-5.621573	-2.302054	0.062067
22	8	0	-0.994753	6.040970	-0.245315
23	6	0	1.601653	0.087736	-0.165008
24	6	0	2.406819	-0.257055	0.935528
25	6	0	2.113752	-0.054945	-1.466281
26	6	0	3.695369	-0.747739	0.703250
27	6	0	3.400070	-0.538897	-1.677866
28	1	0	1.494910	0.217047	-2.316348
29	6	0	4.203884	-0.904464	-0.589833
30	1	0	4.283073	-0.971081	1.587898
31	1	0	3.795808	-0.640813	-2.682336
32	6	0	1.916229	-0.094183	2.382996
33	8	0	2.731730	-0.399022	3.289871
34	8	0	0.736629	0.331445	2.514829
35	6	0	5.586993	-1.417121	-0.881524
36	8	0	6.151172	-1.178024	-1.956305
37	7	0	6.182185	-2.148832	0.095097
38	6	0	7.504989	-2.725115	-0.082447
39	1	0	7.897933	-3.018613	0.891976
40	1	0	7.485488	-3.605488	-0.736223
41	1	0	8.169766	-1.983899	-0.529881
42	1	0	5.631105	-2.455798	0.881954
43	6	0	-2.836166	-4.794014	-0.575886
44	1	0	-2.751109	-5.872534	-0.413567
45	1	0	-2.868420	-4.624179	-1.658356
46	6	0	-4.731289	0.028315	-0.128588
47	6	0	-5.766611	-0.965359	-0.656034
48	6	0	-1.649215	-4.054216	0.040344
49	1	0	-6.779993	-0.580426	-0.509149
50	1	0	-5.626306	-1.120330	-1.731942
51	1	0	-5.018722	0.362950	0.877802
52	1	0	-4.710511	0.928144	-0.749951
53	1	0	-0.714290	-4.325208	-0.460327
54	1	0	-1.540737	-4.364980	1.089012
55	1	0	-6.318582	-3.036993	-0.350438
56	1	0	-5.864359	-2.188846	1.130415
57	9	0	-3.018676	4.150760	-0.172049
58	9	0	1.616414	5.240844	-0.216709

044bba_Rh_Julolidin_F2-H+carb_B3LYP631dp_PCMw_ring.log

Standard orientation:

Center Number	Atomic Number	Atomic Type	Coordinates (Angstroms) X	Y	Z
1	6	0	-1.907236	-2.543683	0.168645
2	6	0	-0.806165	-1.715738	0.319337
3	6	0	-0.888748	-0.315640	0.279731
4	6	0	-2.159411	0.233460	0.075629
5	6	0	-3.308699	-0.549844	-0.099515
6	6	0	-3.188763	-1.958669	-0.050778
7	6	0	0.306204	0.560853	0.534889
8	6	0	-1.282184	2.433404	-0.001400
9	6	0	0.034865	1.984987	0.184937
10	6	0	1.055651	2.960617	0.124051
11	1	0	2.093992	2.679191	0.268459

12	6	0	0.757824	4.278344	-0.118074
13	6	0	-0.570980	4.809228	-0.320280
14	6	0	-1.558097	3.773288	-0.247585
15	1	0	0.163102	-2.178445	0.485850
16	8	0	-2.369926	1.589276	0.023441
17	7	0	-4.303701	-2.765099	-0.259535
18	6	0	-4.229758	-4.194764	0.030585
19	1	0	-5.063095	-4.680261	-0.487782
20	1	0	-4.365487	-4.389071	1.108882
21	6	0	-5.634763	-2.170157	-0.187626
22	8	0	-0.827531	6.028399	-0.546321
23	6	0	1.608008	0.017975	-0.026919
24	6	0	2.486740	-0.305246	0.998938
25	6	0	1.987503	-0.158802	-1.356351
26	6	0	3.758314	-0.818608	0.755427
27	6	0	3.259007	-0.666732	-1.614400
28	1	0	1.316638	0.095493	-2.170476
29	6	0	4.146392	-1.010770	-0.575020
30	1	0	4.421328	-1.028287	1.588623
31	1	0	3.596792	-0.806689	-2.635326
32	6	0	1.839374	-0.001762	2.295871
33	8	0	2.292309	-0.144057	3.417869
34	8	0	0.604854	0.481488	2.056043
35	6	0	5.499874	-1.538098	-0.971384
36	8	0	5.981179	-1.294251	-2.083191
37	7	0	6.156992	-2.280641	-0.045327
38	6	0	7.458709	-2.866667	-0.325933
39	1	0	7.908178	-3.193625	0.612460
40	1	0	7.383138	-3.725889	-1.002733
41	1	0	8.103566	-2.119894	-0.792813
42	1	0	5.658479	-2.604846	0.769357
43	6	0	-2.898962	-4.776795	-0.435911
44	1	0	-2.870020	-5.849028	-0.218433
45	1	0	-2.817722	-4.658292	-1.522889
46	6	0	-4.645087	0.125828	-0.333242
47	6	0	-5.667228	-0.840861	-0.934404
48	6	0	-1.749940	-4.049103	0.263639
49	1	0	-6.673689	-0.413437	-0.887028
50	1	0	-5.438100	-1.023410	-1.990846
51	1	0	-5.028339	0.523102	0.617387
52	1	0	-4.510314	0.995876	-0.982984
53	1	0	-0.785073	-4.350166	-0.158916
54	1	0	-1.728875	-4.352065	1.320324
55	1	0	-6.339802	-2.878700	-0.633750
56	1	0	-5.946469	-2.021439	0.860914
57	9	0	-2.862085	4.133361	-0.439969
58	9	0	1.782862	5.181857	-0.177156

051aaa_Rh_Julolidin_Et_B3LYP631dp_PCMw.log

Standard orientation:

Center Number	Atomic Number	Atomic Type	Coordinates (Angstroms) X	Y	Z
1	6	0	-2.557154	-2.164486	-0.018161
2	6	0	-1.330579	-1.567898	-0.061731
3	6	0	-1.166418	-0.150011	-0.100837
4	6	0	-2.368745	0.627135	-0.098317
5	6	0	-3.637142	0.079775	-0.076817
6	6	0	-3.755589	-1.342601	-0.035995
7	6	0	0.072084	0.494562	-0.124103
8	6	0	-1.103573	2.632464	-0.161112
9	6	0	0.118087	1.916770	-0.167544
10	6	0	1.317146	2.675657	-0.214553
11	1	0	2.257650	2.139694	-0.224449
12	6	0	1.322708	4.054781	-0.250136
13	6	0	0.059503	4.723207	-0.238565
14	6	0	-1.141756	4.018298	-0.196237
15	1	0	-0.442478	-2.189942	-0.054144

16	1	0	-2.087787	4.545622	-0.191233
17	8	0	-2.297492	1.986834	-0.120060
18	7	0	-4.975969	-1.927523	-0.014486
19	6	0	-5.156783	-3.386631	0.061971
20	1	0	-6.088843	-3.623637	-0.457310
21	1	0	-5.286075	-3.677983	1.113804
22	6	0	-6.203334	-1.125601	0.115047
23	8	0	-0.038947	6.067893	-0.270659
24	1	0	0.836286	6.480442	-0.298406
25	6	0	1.338835	-0.298750	-0.203031
26	6	0	2.142499	-0.649718	0.904489
27	6	0	1.749419	-0.697027	-1.484512
28	6	0	3.314002	-1.391147	0.697986
29	6	0	2.918320	-1.426109	-1.672264
30	1	0	1.142225	-0.425468	-2.341585
31	6	0	3.707869	-1.794697	-0.577298
32	1	0	3.912694	-1.617144	1.572939
33	1	0	3.231924	-1.718639	-2.667955
34	6	0	1.853465	-0.291795	2.328314
35	8	0	2.605794	-0.545186	3.251478
36	8	0	0.676946	0.333461	2.506311
37	1	0	0.589254	0.523655	3.456894
38	6	0	4.966183	-2.578011	-0.850340
39	8	0	5.509206	-2.534814	-1.958657
40	7	0	5.458274	-3.312378	0.176885
41	6	0	6.650019	-4.133268	0.023043
42	1	0	7.037088	-4.384186	1.011397
43	1	0	6.439440	-5.060364	-0.522803
44	1	0	7.406529	-3.574087	-0.529791
45	1	0	4.889382	-3.454489	0.997938
46	6	0	-3.989716	-4.138744	-0.561420
47	1	0	-4.134428	-5.211410	-0.407136
48	1	0	-3.971473	-3.959342	-1.642222
49	6	0	-4.859163	0.970907	-0.096744
50	6	0	-6.076881	0.205916	-0.612541
51	6	0	-2.684528	-3.666918	0.075775
52	1	0	-6.990178	0.787039	-0.459028
53	1	0	-5.980322	0.021876	-1.688223
54	1	0	-5.056701	1.347627	0.915769
55	1	0	-4.666192	1.849240	-0.718011
56	1	0	-1.817687	-4.140879	-0.393182
57	1	0	-2.672386	-3.964361	1.133473
58	1	0	-7.023976	-1.715963	-0.298148
59	1	0	-6.417511	-0.966668	1.181049
60	6	0	2.593018	4.881771	-0.301957
61	1	0	2.561546	5.522906	-1.194973
62	1	0	2.606926	5.563602	0.560835
63	6	0	3.903701	4.092071	-0.317846
64	1	0	4.015735	3.480035	0.582215
65	1	0	3.966407	3.434600	-1.190300
66	1	0	4.749799	4.782604	-0.358875

051aba_Rh_Julolidin_Et_B3LYP631dp_PCMw.log

Standard orientation:

Center Number	Atomic Number	Atomic Type	Coordinates (Angstroms) X	Y	Z
1	6	0	-2.550593	-2.171888	-0.016885
2	6	0	-1.324998	-1.572860	-0.061078
3	6	0	-1.163873	-0.154858	-0.101089
4	6	0	-2.367698	0.619084	-0.098536
5	6	0	-3.635302	0.069469	-0.076432
6	6	0	-3.750741	-1.352838	-0.034956
7	6	0	0.073298	0.493227	-0.125100
8	6	0	-1.106420	2.626083	-0.162093
9	6	0	0.116597	1.915125	-0.168931
10	6	0	1.314889	2.678695	-0.216280
11	1	0	2.257022	2.145440	-0.226545

12	6	0	1.315695	4.056426	-0.251521
13	6	0	0.050511	4.720400	-0.239483
14	6	0	-1.149391	4.014313	-0.197046
15	1	0	-0.435705	-2.193196	-0.053320
16	1	0	-2.107211	4.522937	-0.190058
17	8	0	-2.298533	1.979158	-0.120784
18	7	0	-4.970201	-1.940512	-0.013271
19	6	0	-5.147543	-3.399781	0.065373
20	1	0	-6.079463	-3.639802	-0.452826
21	1	0	-5.275199	-3.690399	1.117647
22	6	0	-6.198972	-1.141036	0.116371
23	8	0	0.087811	6.067544	-0.274174
24	1	0	-0.809003	6.434704	-0.264277
25	6	0	1.341666	-0.297493	-0.203819
26	6	0	2.148051	-0.643103	0.903381
27	6	0	1.751227	-0.698553	-1.484751
28	6	0	3.321393	-1.381695	0.697162
29	6	0	2.921813	-1.424987	-1.672261
30	1	0	1.141948	-0.431136	-2.341648
31	6	0	3.714312	-1.787970	-0.577548
32	1	0	3.922123	-1.603300	1.571835
33	1	0	3.234542	-1.719743	-2.667573
34	6	0	1.860040	-0.282361	2.326678
35	8	0	2.615858	-0.528367	3.249006
36	8	0	0.680028	0.336087	2.505454
37	1	0	0.593560	0.528872	3.455626
38	6	0	4.974351	-2.568545	-0.850417
39	8	0	5.514561	-2.528197	-1.960223
40	7	0	5.471239	-3.297212	0.178585
41	6	0	6.665341	-4.114765	0.025300
42	1	0	7.056932	-4.358468	1.013688
43	1	0	6.456154	-5.045825	-0.514308
44	1	0	7.417745	-3.556072	-0.533571
45	1	0	4.904900	-3.437695	1.001684
46	6	0	-3.979168	-4.149850	-0.558123
47	1	0	-4.121299	-5.222732	-0.402889
48	1	0	-3.962023	-3.971273	-1.639084
49	6	0	-4.859064	0.958217	-0.096491
50	6	0	-6.075328	0.190503	-0.611763
51	6	0	-2.674575	-3.674608	0.077816
52	1	0	-6.989771	0.769881	-0.458411
53	1	0	-5.978483	0.006230	-1.687382
54	1	0	-5.057227	1.335001	0.915881
55	1	0	-4.668091	1.836671	-0.718242
56	1	0	-1.807014	-4.146863	-0.391550
57	1	0	-2.660923	-3.971529	1.135646
58	1	0	-7.018622	-1.733053	-0.296502
59	1	0	-6.413501	-0.981965	1.182325
60	6	0	2.573952	4.896915	-0.302950
61	1	0	2.515952	5.546112	-1.185949
62	1	0	2.561731	5.584333	0.552575
63	6	0	3.891532	4.121342	-0.320710
64	1	0	4.013796	3.507338	0.577415
65	1	0	3.964208	3.464465	-1.193588
66	1	0	4.731848	4.819698	-0.360920

052aaa_Rh_Julolidin_Et-H+carb_B3LYP631dp_PCMw.log

Standard orientation:

Center Number	Atomic Number	Atomic Type	Coordinates (Angstroms) X	Y	Z
1	6	0	-2.545965	-2.156404	0.000656
2	6	0	-1.315351	-1.556149	-0.004530
3	6	0	-1.148380	-0.144119	-0.026264
4	6	0	-2.341486	0.629941	-0.050691
5	6	0	-3.615169	0.080746	-0.068416
6	6	0	-3.737501	-1.337575	-0.037820
7	6	0	0.108681	0.495068	0.019672

8	6	0	-1.078647	2.634252	-0.112755
9	6	0	0.140089	1.924084	-0.083932
10	6	0	1.335201	2.683219	-0.134402
11	1	0	2.274689	2.145894	-0.105953
12	6	0	1.341794	4.062563	-0.214262
13	6	0	0.080541	4.727940	-0.241052
14	6	0	-1.117828	4.021124	-0.192379
15	1	0	-0.427070	-2.178041	0.023620
16	1	0	-2.065606	4.545097	-0.218967
17	8	0	-2.278132	1.993497	-0.071923
18	7	0	-4.967346	-1.923383	-0.056287
19	6	0	-5.144480	-3.378437	0.036068
20	1	0	-6.073868	-3.626591	-0.484142
21	1	0	-5.274801	-3.668665	1.089179
22	6	0	-6.188084	-1.117900	0.070274
23	8	0	-0.019307	6.075463	-0.319387
24	6	0	1.364871	-0.295697	-0.162710
25	6	0	2.140239	-0.690883	0.936362
26	6	0	1.775956	-0.638253	-1.460106
27	6	0	3.305140	-1.430396	0.729643
28	6	0	2.943432	-1.370497	-1.653997
29	1	0	1.181873	-0.330933	-2.315444
30	6	0	3.715150	-1.786269	-0.559655
31	1	0	3.878268	-1.689447	1.614452
32	1	0	3.271475	-1.631609	-2.653936
33	6	0	1.690751	-0.298794	2.339825
34	8	0	2.403452	-0.648055	3.307203
35	8	0	0.606610	0.360344	2.371258
36	6	0	4.964277	-2.574985	-0.845787
37	8	0	5.509938	-2.536927	-1.954891
38	7	0	5.457177	-3.320875	0.175290
39	6	0	6.638214	-4.153165	0.011213
40	1	0	7.020927	-4.424193	0.996148
41	1	0	6.419380	-5.070245	-0.548855
42	1	0	7.403996	-3.596555	-0.531954
43	1	0	4.894970	-3.451648	1.002358
44	6	0	-3.971171	-4.130777	-0.577842
45	1	0	-4.117399	-5.204505	-0.430273
46	1	0	-3.942342	-3.946310	-1.657806
47	6	0	-4.834320	0.976046	-0.120638
48	6	0	-6.047164	0.214619	-0.654535
49	6	0	-2.671501	-3.661812	0.073856
50	1	0	-6.960576	0.800027	-0.516714
51	1	0	-5.933939	0.029256	-1.728600
52	1	0	-5.051830	1.363588	0.883948
53	1	0	-4.625619	1.849431	-0.744390
54	1	0	-1.801075	-4.128516	-0.396797
55	1	0	-2.664791	-3.977062	1.126567
56	1	0	-7.011161	-1.700443	-0.350929
57	1	0	-6.414592	-0.954901	1.134226
58	6	0	2.613112	4.888928	-0.270693
59	1	0	2.596089	5.505371	-1.181592
60	1	0	2.615329	5.595707	0.572119
61	6	0	3.924187	4.099966	-0.245120
62	1	0	4.022997	3.513871	0.673596
63	1	0	3.998550	3.416561	-1.096535
64	1	0	4.771682	4.788512	-0.294348
65	1	0	0.857613	6.483283	-0.349946

052aba_Rh_Julolidin_Et-H+carb_B3LYP631dp_PCMw.log

Standard orientation:

Center Number	Atomic Number	Atomic Type	Coordinates (Angstroms) X	Y	Z
1	6	0	-2.536764	-2.164799	0.000293
2	6	0	-1.307798	-1.560945	-0.005594
3	6	0	-1.145158	-0.148485	-0.027649
4	6	0	-2.340648	0.621429	-0.051297

5	6	0	-3.612910	0.068711	-0.068470
6	6	0	-3.730864	-1.349779	-0.037901
7	6	0	0.109820	0.495286	0.016545
8	6	0	-1.083274	2.628370	-0.112822
9	6	0	0.137251	1.923894	-0.086355
10	6	0	1.331060	2.688756	-0.137572
11	1	0	2.272525	2.154626	-0.111521
12	6	0	1.332157	4.066797	-0.215149
13	6	0	0.068401	4.726762	-0.238066
14	6	0	-1.128074	4.017684	-0.189646
15	1	0	-0.417691	-2.180232	0.022061
16	1	0	-2.087703	4.523039	-0.211758
17	8	0	-2.280651	1.985295	-0.072036
18	7	0	-4.959091	-1.939479	-0.056327
19	6	0	-5.131447	-3.394906	0.037850
20	1	0	-6.060539	-3.646688	-0.481162
21	1	0	-5.259723	-3.684617	1.091380
22	6	0	-6.182131	-1.137678	0.070578
23	8	0	0.100173	6.077207	-0.313677
24	6	0	1.368439	-0.291996	-0.164062
25	6	0	2.144424	-0.684951	0.935519
26	6	0	1.781063	-0.633903	-1.461171
27	6	0	3.311361	-1.421377	0.729081
28	6	0	2.950484	-1.363149	-1.654590
29	1	0	1.186492	-0.328356	-2.316816
30	6	0	3.722944	-1.776542	-0.559901
31	1	0	3.884609	-1.678508	1.614366
32	1	0	3.279506	-1.623686	-2.654357
33	6	0	1.694663	-0.294603	2.339650
34	8	0	2.409246	-0.643324	3.306025
35	8	0	0.609278	0.361966	2.372725
36	6	0	4.974239	-2.562045	-0.845394
37	8	0	5.518584	-2.525061	-1.955202
38	7	0	5.470721	-3.303802	0.176968
39	6	0	6.654414	-4.132471	0.013853
40	1	0	7.040408	-4.397795	0.999071
41	1	0	6.437728	-5.052823	-0.541683
42	1	0	7.416801	-3.575363	-0.533534
43	1	0	4.910212	-3.433847	1.005289
44	6	0	-3.956280	-4.144004	-0.576528
45	1	0	-4.098969	-5.218104	-0.428173
46	1	0	-3.928937	-3.960113	-1.656630
47	6	0	-4.834690	0.960427	-0.120215
48	6	0	-6.045286	0.195380	-0.654080
49	6	0	-2.657506	-3.670620	0.073790
50	1	0	-6.960451	0.778027	-0.516156
51	1	0	-5.931534	0.010495	-1.728171
52	1	0	-5.053205	1.347132	0.884476
53	1	0	-4.628820	1.834541	-0.743918
54	1	0	-1.786027	-4.134634	-0.397579
55	1	0	-2.648727	-3.985611	1.126564
56	1	0	-7.003567	-1.722551	-0.350649
57	1	0	-6.409138	-0.975412	1.134565
58	6	0	2.591375	4.906813	-0.273054
59	1	0	2.547076	5.531388	-1.174626
60	1	0	2.569415	5.618349	0.562498
61	6	0	3.909442	4.131778	-0.251198
62	1	0	4.019495	3.543544	0.665598
63	1	0	3.992911	3.449118	-1.103144
64	1	0	4.751083	4.828208	-0.300735
65	1	0	-0.799934	6.435176	-0.321099

052bba_Rh_Julolidin_Et-H+carb_B3LYP631dp_PCMw_ring.log

Standard orientation:

Center Number	Atomic Number	Atomic Type	Coordinates (Angstroms) X	Y	Z
1	6	0	-2.623661	-2.071692	0.284014

2	6	0	-1.372324	-1.496362	0.429137
3	6	0	-1.143440	-0.118291	0.296645
4	6	0	-2.253349	0.675541	-0.004573
5	6	0	-3.540776	0.154107	-0.185835
6	6	0	-3.737832	-1.239426	-0.033965
7	6	0	0.215192	0.481868	0.542605
8	6	0	-0.922547	2.635469	-0.117200
9	6	0	0.252346	1.943484	0.176139
10	6	0	1.455501	2.674699	0.181857
11	1	0	2.373185	2.144881	0.411653
12	6	0	1.520015	4.034883	-0.089135
13	6	0	0.301121	4.694006	-0.382125
14	6	0	-0.906006	4.004948	-0.400077
15	1	0	-0.534670	-2.146187	0.668572
16	1	0	-1.839887	4.508270	-0.630071
17	8	0	-2.154498	2.041153	-0.159322
18	7	0	-4.995624	-1.795705	-0.231887
19	6	0	-5.247547	-3.182799	0.151795
20	1	0	-6.151480	-3.510705	-0.371507
21	1	0	-5.454545	-3.263067	1.232883
22	6	0	-6.164091	-0.921147	-0.269339
23	8	0	0.376691	6.028608	-0.649527
24	6	0	1.358647	-0.328779	-0.044924
25	6	0	2.165786	-0.819313	0.973412
26	6	0	1.662603	-0.601469	-1.376949
27	6	0	3.290512	-1.601242	0.720027
28	6	0	2.789187	-1.376641	-1.644421
29	1	0	1.046739	-0.221719	-2.185604
30	6	0	3.599927	-1.891532	-0.613059
31	1	0	3.908350	-1.940801	1.545068
32	1	0	3.068912	-1.601129	-2.667719
33	6	0	1.621774	-0.360629	2.269914
34	8	0	2.038329	-0.568229	3.392178
35	8	0	0.510243	0.378284	2.027048
36	6	0	4.797431	-2.708415	-1.023039
37	8	0	5.305190	-2.578493	-2.141916
38	7	0	5.283855	-3.576681	-0.101499
39	6	0	6.418969	-4.438180	-0.395381
40	1	0	6.814948	-4.833365	0.540899
41	1	0	6.139335	-5.276010	-1.044953
42	1	0	7.194751	-3.859057	-0.899125
43	1	0	4.735105	-3.782075	0.719788
44	6	0	-4.065083	-4.073412	-0.215944
45	1	0	-4.279441	-5.106802	0.073408
46	1	0	-3.928097	-4.057046	-1.303592
47	6	0	-4.686163	1.084037	-0.534150
48	6	0	-5.877430	0.316896	-1.111804
49	6	0	-2.805101	-3.563744	0.485048
50	1	0	-6.764795	0.956475	-1.145193
51	1	0	-5.660633	0.002464	-2.139317
52	1	0	-5.001148	1.633385	0.364126
53	1	0	-4.343233	1.844623	-1.242009
54	1	0	-1.917811	-4.097228	0.127231
55	1	0	-2.881917	-3.783427	1.559390
56	1	0	-6.993350	-1.492737	-0.697504
57	1	0	-6.468005	-0.623443	0.748961
58	6	0	2.807144	4.836037	-0.087046
59	1	0	2.913428	5.324495	-1.064596
60	1	0	2.701061	5.662420	0.628173
61	6	0	4.081908	4.051772	0.229025
62	1	0	4.045865	3.601716	1.226655
63	1	0	4.254856	3.249661	-0.496104
64	1	0	4.948344	4.718625	0.198256
65	1	0	-0.507388	6.375865	-0.835662

053aaa_Rh_Julolidin_Et-H+oh_B3LYP631dp_PCMw.log

Standard orientation:

Center Atomic Atomic Coordinates (Angstroms)

Number	Number	Type	X	Y	Z
1	6	0	-2.491728	-2.131821	-0.100583
2	6	0	-1.269353	-1.499248	-0.118892
3	6	0	-1.137545	-0.090155	-0.119637
4	6	0	-2.341582	0.653948	-0.119240
5	6	0	-3.604844	0.072985	-0.120981
6	6	0	-3.695464	-1.345720	-0.106444
7	6	0	0.107924	0.606589	-0.120822
8	6	0	-1.126774	2.710585	-0.138422
9	6	0	0.123667	1.996074	-0.142803
10	6	0	1.321501	2.796100	-0.149350
11	1	0	2.268289	2.267671	-0.145916
12	6	0	1.301365	4.158032	-0.159757
13	6	0	0.008214	4.881705	-0.162881
14	6	0	-1.191580	4.075179	-0.149943
15	1	0	-0.370519	-2.107389	-0.124585
16	1	0	-2.153402	4.576544	-0.148666
17	8	0	-2.309120	2.021063	-0.119559
18	7	0	-4.922365	-1.960809	-0.119008
19	6	0	-5.052363	-3.413647	0.017489
20	1	0	-6.000209	-3.702780	-0.446179
21	1	0	-5.115272	-3.691763	1.081402
22	6	0	-6.148084	-1.181895	0.069282
23	8	0	-0.028615	6.132705	-0.175206
24	6	0	1.385047	-0.172622	-0.194690
25	6	0	2.058257	-0.738010	0.912014
26	6	0	1.930778	-0.365150	-1.474048
27	6	0	3.228128	-1.483318	0.703225
28	6	0	3.098977	-1.094070	-1.663817
29	1	0	1.422539	0.066844	-2.329794
30	6	0	3.753876	-1.678120	-0.573303
31	1	0	3.722098	-1.878974	1.583275
32	1	0	3.515145	-1.222451	-2.656768
33	6	0	1.641791	-0.604012	2.343151
34	8	0	2.209340	-1.168620	3.262401
35	8	0	0.587285	0.204517	2.542252
36	1	0	0.409109	0.209981	3.499156
37	6	0	5.016711	-2.453615	-0.842500
38	8	0	5.686632	-2.242670	-1.858768
39	7	0	5.369995	-3.377027	0.085272
40	6	0	6.551944	-4.209287	-0.079102
41	1	0	6.815541	-4.644433	0.885741
42	1	0	6.385796	-5.018152	-0.800388
43	1	0	7.380587	-3.596124	-0.436940
44	1	0	4.705545	-3.635049	0.799131
45	6	0	-3.889605	-4.142434	-0.645776
46	1	0	-4.003140	-5.219581	-0.492198
47	1	0	-3.913888	-3.958294	-1.726066
48	6	0	-4.843444	0.943348	-0.137701
49	6	0	-6.056928	0.162608	-0.642778
50	6	0	-2.572208	-3.643158	-0.053404
51	1	0	-6.977210	0.728825	-0.471898
52	1	0	-5.972321	-0.009109	-1.721924
53	1	0	-5.042476	1.324022	0.873647
54	1	0	-4.668098	1.824065	-0.761855
55	1	0	-1.715493	-4.076263	-0.579436
56	1	0	-2.498936	-3.981630	0.989719
57	1	0	-6.978418	-1.771876	-0.328650
58	1	0	-6.342065	-1.032100	1.143065
59	6	0	2.541215	5.019002	-0.167219
60	1	0	2.483736	5.686186	-1.037672
61	1	0	2.486828	5.697642	0.694559
62	6	0	3.879902	4.281018	-0.164657
63	1	0	3.994397	3.651934	0.724642
64	1	0	3.990474	3.638668	-1.044916
65	1	0	4.707014	4.997135	-0.171764

054aaa_Rh_Julolidin_Et-2H+_B3LYP631dp_PCMw.log

Standard orientation:

Center Number	Atomic Number	Atomic Type	Coordinates (Angstroms) X	Y	Z
1	6	0	-2.510213	-2.125432	-0.058096
2	6	0	-1.282713	-1.499552	-0.056307
3	6	0	-1.140133	-0.093441	-0.064621
4	6	0	-2.336459	0.657214	-0.087565
5	6	0	-3.605764	0.083602	-0.108107
6	6	0	-3.706595	-1.332339	-0.088976
7	6	0	0.118330	0.588722	-0.041785
8	6	0	-1.108311	2.702783	-0.117141
9	6	0	0.133364	1.981314	-0.103804
10	6	0	1.333049	2.775498	-0.122376
11	1	0	2.275816	2.240100	-0.103715
12	6	0	1.322066	4.138545	-0.158851
13	6	0	0.036396	4.870157	-0.179257
14	6	0	-1.165385	4.069982	-0.155735
15	1	0	-0.385484	-2.109724	-0.036303
16	1	0	-2.125528	4.574943	-0.170834
17	8	0	-2.298654	2.024914	-0.095249
18	7	0	-4.941372	-1.940875	-0.125760
19	6	0	-5.078956	-3.388335	0.041843
20	1	0	-6.025962	-3.683761	-0.420217
21	1	0	-5.147237	-3.647335	1.110896
22	6	0	-6.159045	-1.151378	0.059798
23	8	0	0.006902	6.123780	-0.216518
24	6	0	1.386532	-0.197976	-0.161961
25	6	0	2.080755	-0.730167	0.941088
26	6	0	1.887985	-0.411874	-1.458180
27	6	0	3.245471	-1.469881	0.711732
28	6	0	3.050882	-1.145160	-1.665801
29	1	0	1.355446	0.003052	-2.309056
30	6	0	3.738663	-1.696738	-0.576830
31	1	0	3.754334	-1.832065	1.599414
32	1	0	3.437391	-1.301261	-2.666981
33	6	0	1.616017	-0.512986	2.392302
34	8	0	2.336581	-1.021295	3.289967
35	8	0	0.557105	0.153462	2.537930
36	6	0	4.989423	-2.480864	-0.860931
37	8	0	5.609607	-2.348243	-1.923408
38	7	0	5.402073	-3.337035	0.108919
39	6	0	6.576770	-4.175996	-0.061852
40	1	0	6.876937	-4.567993	0.910934
41	1	0	6.386224	-5.017428	-0.739101
42	1	0	7.393511	-3.583150	-0.477998
43	1	0	4.785797	-3.532022	0.882927
44	6	0	-3.915445	-4.134416	-0.601334
45	1	0	-4.037544	-5.209038	-0.435933
46	1	0	-3.929310	-3.963210	-1.684060
47	6	0	-4.837297	0.963844	-0.148579
48	6	0	-6.053433	0.189434	-0.657813
49	6	0	-2.599669	-3.636742	-0.003410
50	1	0	-6.970588	0.763837	-0.496744
51	1	0	-5.962641	0.011057	-1.735482
52	1	0	-5.044142	1.358446	0.855988
53	1	0	-4.647870	1.836491	-0.780253
54	1	0	-1.741864	-4.077515	-0.521651
55	1	0	-2.534377	-3.971081	1.041683
56	1	0	-6.995109	-1.734538	-0.337078
57	1	0	-6.354945	-0.993498	1.132726
58	6	0	2.568958	4.990594	-0.177959
59	1	0	2.519547	5.644575	-1.059051
60	1	0	2.519801	5.683942	0.672605
61	6	0	3.902339	4.242906	-0.160739
62	1	0	4.010501	3.627710	0.739045
63	1	0	4.008890	3.584135	-1.029385
64	1	0	4.735671	4.951855	-0.178789

054bba_Rh_Julolidin_Et-H+carb_B3LYP631dp_PCMw_ring.log

Standard orientation:

Center Number	Atomic Number	Atomic Type	Coordinates (Angstroms) X	Y	Z
1	6	0	-2.593647	-2.071989	0.216241
2	6	0	-1.347952	-1.481749	0.359340
3	6	0	-1.139060	-0.097602	0.265614
4	6	0	-2.264948	0.694588	0.012079
5	6	0	-3.548200	0.153766	-0.163625
6	6	0	-3.722712	-1.245573	-0.055830
7	6	0	0.208398	0.522534	0.503961
8	6	0	-0.949950	2.678683	-0.089031
9	6	0	0.234502	1.964626	0.143901
10	6	0	1.437342	2.717125	0.112424
11	1	0	2.366366	2.184109	0.297195
12	6	0	1.480774	4.073441	-0.133964
13	6	0	0.245570	4.818392	-0.376685
14	6	0	-0.960509	4.041464	-0.345300
15	1	0	-0.497379	-2.126574	0.565279
16	1	0	-1.908196	4.538018	-0.534408
17	8	0	-2.189465	2.057937	-0.094143
18	7	0	-4.978748	-1.815291	-0.256525
19	6	0	-5.203760	-3.211515	0.107524
20	1	0	-6.117416	-3.541445	-0.397739
21	1	0	-5.380761	-3.316857	1.192390
22	6	0	-6.156886	-0.954596	-0.226429
23	8	0	0.255985	6.069238	-0.608011
24	6	0	1.367814	-0.289702	-0.044492
25	6	0	2.159428	-0.785940	0.983427
26	6	0	1.699870	-0.554564	-1.372404
27	6	0	3.290749	-1.562046	0.743234
28	6	0	2.831396	-1.326203	-1.627363
29	1	0	1.097937	-0.167389	-2.188237
30	6	0	3.626080	-1.845192	-0.585608
31	1	0	3.894926	-1.903402	1.577862
32	1	0	3.128308	-1.543753	-2.647441
33	6	0	1.595650	-0.341257	2.279871
34	8	0	2.019268	-0.570813	3.400796
35	8	0	0.493246	0.388104	2.042368
36	6	0	4.831211	-2.657468	-0.977785
37	8	0	5.354476	-2.531191	-2.090427
38	7	0	5.310517	-3.521368	-0.047225
39	6	0	6.451944	-4.379485	-0.324628
40	1	0	6.836760	-4.772412	0.617322
41	1	0	6.184530	-5.219304	-0.976987
42	1	0	7.232942	-3.798799	-0.818558
43	1	0	4.751765	-3.726775	0.767198
44	6	0	-4.020411	-4.079547	-0.308886
45	1	0	-4.216405	-5.122511	-0.040978
46	1	0	-3.909611	-4.034352	-1.398799
47	6	0	-4.712162	1.078253	-0.461236
48	6	0	-5.908468	0.314991	-1.033733
49	6	0	-2.749843	-3.572370	0.374907
50	1	0	-6.804797	0.943129	-1.024459
51	1	0	-5.714007	0.036403	-2.076100
52	1	0	-5.013536	1.600841	0.457763
53	1	0	-4.392035	1.862431	-1.154129
54	1	0	-1.865319	-4.082656	-0.022186
55	1	0	-2.795386	-3.828122	1.443284
56	1	0	-6.993473	-1.520460	-0.648555
57	1	0	-6.433191	-0.694374	0.810469
58	6	0	2.764017	4.874409	-0.174569
59	1	0	2.825548	5.381916	-1.148261
60	1	0	2.681498	5.699884	0.547019
61	6	0	4.061161	4.104458	0.079960
62	1	0	4.070291	3.640607	1.072771
63	1	0	4.211182	3.307482	-0.657071
64	1	0	4.924604	4.775184	0.021456

061aaa_Rh_Julolidin_naft1_B3LYP631dp_PCMw.log

Standard orientation:

Center Number	Atomic Number	Atomic Type	Coordinates (Angstroms) X	Y	Z
1	6	0	-2.453605	-2.210961	-0.048344
2	6	0	-1.248855	-1.564230	-0.072414
3	6	0	-1.139875	-0.141335	-0.081152
4	6	0	-2.371600	0.569370	-0.108762
5	6	0	-3.621586	-0.021350	-0.106336
6	6	0	-3.684143	-1.444308	-0.065804
7	6	0	0.078432	0.565904	-0.062574
8	6	0	-1.185765	2.623518	-0.171869
9	6	0	0.080087	1.992013	-0.074700
10	6	0	1.242356	2.885424	-0.015769
11	6	0	1.055534	4.296758	-0.170634
12	6	0	-0.272057	4.829609	-0.326056
13	6	0	-1.372546	4.004177	-0.300155
14	1	0	-0.344247	-2.160112	-0.068688
15	1	0	-2.382028	4.389346	-0.388251
16	8	0	-2.342345	1.926918	-0.167351
17	7	0	-4.882260	-2.079162	-0.056734
18	6	0	-4.999031	-3.543262	0.021602
19	1	0	-5.923285	-3.822305	-0.490962
20	1	0	-5.106475	-3.843495	1.073690
21	6	0	-6.137653	-1.323875	0.067802
22	8	0	-0.368588	6.160759	-0.473880
23	1	0	-1.296199	6.427134	-0.564116
24	6	0	1.322526	-0.264172	-0.169118
25	6	0	1.932155	-0.941831	0.910051
26	6	0	1.846463	-0.444087	-1.457835
27	6	0	3.033549	-1.774536	0.669526
28	6	0	2.950541	-1.260062	-1.677025
29	1	0	1.378277	0.063879	-2.294307
30	6	0	3.549475	-1.947720	-0.614951
31	1	0	3.483539	-2.255015	1.530729
32	1	0	3.358474	-1.379029	-2.674404
33	6	0	1.516433	-0.817717	2.341165
34	8	0	1.930089	-1.540516	3.228781
35	8	0	0.655579	0.190262	2.567146
36	1	0	0.445603	0.188686	3.517602
37	6	0	4.748098	-2.808074	-0.923806
38	8	0	5.423923	-2.607561	-1.937827
39	7	0	5.037724	-3.789419	-0.035602
40	6	0	6.155719	-4.697440	-0.243585
41	1	0	6.380424	-5.202146	0.696719
42	1	0	5.932661	-5.451043	-1.007819
43	1	0	7.030123	-4.129990	-0.566574
44	1	0	4.363711	-4.025808	0.676688
45	6	0	-3.803409	-4.240895	-0.612605
46	1	0	-3.900688	-5.320081	-0.466337
47	1	0	-3.798828	-4.052388	-1.692052
48	6	0	-4.875237	0.823844	-0.154293
49	6	0	-6.058585	0.006218	-0.670742
50	6	0	-2.515160	-3.719597	0.021355
51	1	0	-6.994761	0.553062	-0.529147
52	1	0	-5.947078	-0.183944	-1.743917
53	1	0	-5.098152	1.208914	0.849760
54	1	0	-4.708570	1.699141	-0.787713
55	1	0	-1.632367	-4.147858	-0.461943
56	1	0	-2.480012	-4.033015	1.073917
57	1	0	-6.936055	-1.946533	-0.341926
58	1	0	-6.360781	-1.163014	1.132012
59	6	0	2.156054	5.181995	-0.140039
60	6	0	2.568109	2.449621	0.218214
61	6	0	3.436927	4.709448	0.062409
62	1	0	4.277720	5.394645	0.086864

63	6	0	3.633657	3.333383	0.254737
64	1	0	4.631279	2.948414	0.440950
65	1	0	1.972352	6.241712	-0.268032
66	1	0	2.775800	1.407046	0.388893

062aaa_Rh_Julolidin_naft1-H+carb_B3LYP631dp_PCMw.log

Standard orientation:

Center Number	Atomic Number	Atomic Type	Coordinates (Angstroms) X	Y	Z
1	6	0	-2.480394	-2.190146	0.039715
2	6	0	-1.265643	-1.554181	0.039773
3	6	0	-1.138036	-0.138232	0.002073
4	6	0	-2.352507	0.583925	-0.070548
5	6	0	-3.613115	0.004338	-0.096840
6	6	0	-3.695688	-1.413092	-0.030923
7	6	0	0.105260	0.548341	0.063884
8	6	0	-1.143168	2.619478	-0.160574
9	6	0	0.106543	1.981751	-0.017853
10	6	0	1.274600	2.861129	0.032847
11	6	0	1.115727	4.268164	-0.181470
12	6	0	-0.199771	4.811433	-0.385226
13	6	0	-1.308408	4.000878	-0.348033
14	1	0	-0.366543	-2.156837	0.091367
15	1	0	-2.311235	4.393274	-0.474806
16	8	0	-2.314748	1.942735	-0.157779
17	7	0	-4.908789	-2.036348	-0.054304
18	6	0	-5.040167	-3.491751	0.082593
19	1	0	-5.959135	-3.786005	-0.432196
20	1	0	-5.163922	-3.756300	1.143467
21	6	0	-6.151373	-1.261744	0.036341
22	8	0	-0.275273	6.140980	-0.590622
23	6	0	1.326986	-0.294436	-0.146935
24	6	0	1.963316	-0.938636	0.924563
25	6	0	1.786408	-0.506318	-1.454645
26	6	0	3.043240	-1.786052	0.677599
27	6	0	2.875674	-1.341589	-1.686694
28	1	0	1.293106	-0.016418	-2.288658
29	6	0	3.508252	-2.002902	-0.624843
30	1	0	3.513474	-2.236947	1.545885
31	1	0	3.246472	-1.497612	-2.693649
32	6	0	1.497715	-0.642639	2.347271
33	8	0	2.038266	-1.267933	3.286699
34	8	0	0.591093	0.242235	2.421305
35	6	0	4.678121	-2.890308	-0.953350
36	8	0	5.306559	-2.761555	-2.010649
37	7	0	5.006149	-3.829614	-0.030545
38	6	0	6.097159	-4.765302	-0.250862
39	1	0	6.356941	-5.236087	0.698180
40	1	0	5.828438	-5.546149	-0.972367
41	1	0	6.966529	-4.229677	-0.636716
42	1	0	4.373872	-4.008771	0.734360
43	6	0	-3.840120	-4.222237	-0.506739
44	1	0	-3.952866	-5.296239	-0.333663
45	1	0	-3.811736	-4.063191	-1.590775
46	6	0	-4.853905	0.864752	-0.200241
47	6	0	-6.036891	0.052460	-0.727072
48	6	0	-2.558329	-3.697826	0.138844
49	1	0	-6.968196	0.615858	-0.620732
50	1	0	-5.902015	-0.162665	-1.793045
51	1	0	-5.098925	1.280104	0.786720
52	1	0	-4.658752	1.721905	-0.850603
53	1	0	-1.671966	-4.144692	-0.321396
54	1	0	-2.543715	-3.993878	1.197067
55	1	0	-6.954114	-1.877938	-0.376512
56	1	0	-6.395854	-1.072314	1.092180
57	6	0	2.232273	5.134977	-0.158478
58	6	0	2.585682	2.411174	0.322272

59	6	0	3.498154	4.648313	0.095237
60	1	0	4.350934	5.319101	0.113673
61	6	0	3.665178	3.276789	0.349522
62	1	0	4.651231	2.884569	0.578936
63	1	0	2.071422	6.192007	-0.332823
64	1	0	2.759245	1.371172	0.542380
65	1	0	-1.198010	6.410684	-0.711213

062baa_Rh_Julolidin_naft1-H+carb_B3LYP631dp_PCMw.log

Standard orientation:

Center Number	Atomic Number	Atomic Type	Coordinates (Angstroms) X	Y	Z
1	6	0	-2.706369	-1.950108	0.467243
2	6	0	-1.437096	-1.409829	0.594965
3	6	0	-1.147772	-0.061583	0.330142
4	6	0	-2.214888	0.726899	-0.090546
5	6	0	-3.517766	0.242367	-0.262837
6	6	0	-3.776754	-1.117825	0.024299
7	6	0	0.243051	0.506274	0.536091
8	6	0	-0.817584	2.635951	-0.259750
9	6	0	0.324634	1.974442	0.166781
10	6	0	1.539510	2.758979	0.256410
11	6	0	1.534476	4.145200	-0.117187
12	6	0	0.312961	4.741623	-0.569947
13	6	0	-0.836691	4.000115	-0.632946
14	1	0	-0.639262	-2.068609	0.925151
15	1	0	-1.776975	4.427583	-0.964259
16	8	0	-2.047925	2.058817	-0.381327
17	7	0	-5.049443	-1.644736	-0.159017
18	6	0	-5.367969	-2.972118	0.361543
19	1	0	-6.268823	-3.321041	-0.153513
20	1	0	-5.609005	-2.928301	1.437699
21	6	0	-6.178827	-0.732930	-0.317813
22	8	0	0.368100	6.053121	-0.917725
23	6	0	1.283176	-0.411503	-0.095099
24	6	0	1.953349	-1.113894	0.900423
25	6	0	1.572229	-0.642744	-1.436612
26	6	0	2.923515	-2.069609	0.610938
27	6	0	2.550175	-1.589383	-1.739277
28	1	0	1.060535	-0.102196	-2.226247
29	6	0	3.217854	-2.317439	-0.734797
30	1	0	3.445417	-2.571203	1.419391
31	1	0	2.820374	-1.786123	-2.770886
32	6	0	1.492665	-0.619351	2.216570
33	8	0	1.845234	-0.954524	3.329059
34	8	0	0.564619	0.351339	2.005005
35	6	0	4.261367	-3.308338	-1.179718
36	8	0	4.821270	-3.197011	-2.275410
37	7	0	4.552684	-4.310998	-0.313816
38	6	0	5.525221	-5.341297	-0.644730
39	1	0	5.813920	-5.862022	0.269089
40	1	0	5.122437	-6.070493	-1.357553
41	1	0	6.407468	-4.878674	-1.090283
42	1	0	3.948723	-4.468167	0.478803
43	6	0	-4.211589	-3.939408	0.129346
44	1	0	-4.475401	-4.927768	0.518018
45	1	0	-4.041917	-4.043414	-0.948762
46	6	0	-4.611616	1.176897	-0.739350
47	6	0	-5.819705	0.402224	-1.270676
48	6	0	-2.952877	-3.405577	0.814959
49	1	0	-6.678516	1.069464	-1.392059
50	1	0	-5.592162	-0.021781	-2.255571
51	1	0	-4.924894	1.829068	0.087901
52	1	0	-4.218990	1.845137	-1.511744
53	1	0	-2.077855	-4.005881	0.543077
54	1	0	-3.068933	-3.508993	1.903172
55	1	0	-7.020496	-1.311443	-0.711005

56	1	0	-6.495267	-0.322680	0.656651
57	6	0	2.715065	4.922873	-0.033818
58	6	0	2.780097	2.239477	0.718389
59	6	0	3.896190	4.372156	0.413709
60	1	0	4.796375	4.975240	0.476253
61	6	0	3.919078	3.016248	0.793345
62	1	0	4.841644	2.570381	1.153133
63	1	0	2.667460	5.964927	-0.327674
64	1	0	2.854600	1.207583	1.020016
65	1	0	-0.506186	6.352866	-1.206084

063aaa_Rh_Julolidin_naft1-H+oh_B3LYP631dp_PCMw.log

Standard orientation:

Center Number	Atomic Number	Atomic Type	Coordinates (Angstroms) X	Y	Z
1	6	0	-2.501224	-2.124897	-0.092099
2	6	0	-1.278733	-1.488851	-0.102233
3	6	0	-1.147484	-0.079134	-0.088387
4	6	0	-2.353579	0.650060	-0.110139
5	6	0	-3.617818	0.072342	-0.115471
6	6	0	-3.706857	-1.345542	-0.096445
7	6	0	0.102868	0.629059	-0.044377
8	6	0	-1.141599	2.709642	-0.179083
9	6	0	0.126516	2.022679	-0.039846
10	6	0	1.311005	2.899926	0.062279
11	6	0	1.173996	4.297028	-0.171682
12	6	0	-0.146951	4.929077	-0.438863
13	6	0	-1.282187	4.057424	-0.371520
14	1	0	-0.385269	-2.102819	-0.108551
15	1	0	-2.273597	4.476080	-0.502787
16	8	0	-2.315538	2.013489	-0.150533
17	7	0	-4.932924	-1.962884	-0.111747
18	6	0	-5.057717	-3.415670	0.025413
19	1	0	-6.005991	-3.708563	-0.435060
20	1	0	-5.115407	-3.695047	1.089366
21	6	0	-6.158207	-1.183701	0.075137
22	8	0	-0.247289	6.150152	-0.675523
23	6	0	1.331601	-0.223886	-0.150602
24	6	0	1.870337	-1.003650	0.898050
25	6	0	1.915539	-0.327503	-1.422642
26	6	0	2.955967	-1.854232	0.645445
27	6	0	3.003753	-1.160313	-1.654515
28	1	0	1.502263	0.257218	-2.237610
29	6	0	3.529655	-1.947132	-0.622581
30	1	0	3.348922	-2.414770	1.485948
31	1	0	3.455652	-1.217410	-2.638482
32	6	0	1.390959	-0.981476	2.314279
33	8	0	1.694027	-1.823019	3.141400
34	8	0	0.613708	0.074804	2.610542
35	1	0	0.357537	-0.008763	3.545867
36	6	0	4.713800	-2.822333	-0.939450
37	8	0	5.446344	-2.572723	-1.902340
38	7	0	4.929313	-3.877022	-0.115233
39	6	0	6.025851	-4.806360	-0.341379
40	1	0	6.179815	-5.397359	0.562190
41	1	0	5.821549	-5.483299	-1.179159
42	1	0	6.936415	-4.248317	-0.566367
43	1	0	4.211931	-4.143955	0.541733
44	6	0	-3.893964	-4.138990	-0.642847
45	1	0	-4.003044	-5.216944	-0.491600
46	1	0	-3.922581	-3.952318	-1.722602
47	6	0	-4.856103	0.942669	-0.149130
48	6	0	-6.068493	0.155570	-0.647825
49	6	0	-2.575661	-3.637125	-0.054004
50	1	0	-6.989323	0.722398	-0.482012
51	1	0	-5.983433	-0.025314	-1.725423
52	1	0	-5.058570	1.336624	0.856393

53	1	0	-4.678614	1.815095	-0.784335
54	1	0	-1.720151	-4.063919	-0.587262
55	1	0	-2.495275	-3.981806	0.986544
56	1	0	-6.989605	-1.776513	-0.316304
57	1	0	-6.349735	-1.025283	1.148208
58	6	0	2.289876	5.145661	-0.104002
59	6	0	2.596368	2.439139	0.428105
60	6	0	3.546760	4.658207	0.217248
61	1	0	4.402690	5.324204	0.268655
62	6	0	3.688261	3.294402	0.500102
63	1	0	4.655498	2.893670	0.788707
64	1	0	2.123136	6.199394	-0.299715
65	1	0	2.751238	1.402904	0.682522

064aaa_Rh_Julolidin_naft1-2H_B3LYP631dp_PCMw.log

Standard orientation:

Center Number	Atomic Number	Atomic Type	Coordinates (Angstroms) X	Y	Z
1	6	0	-2.532455	-2.108746	-0.005206
2	6	0	-1.302981	-1.484057	0.005890
3	6	0	-1.156058	-0.078014	-0.018987
4	6	0	-2.350906	0.661559	-0.094076
5	6	0	-3.622696	0.095028	-0.125021
6	6	0	-3.727459	-1.318862	-0.071527
7	6	0	0.108401	0.610847	0.045539
8	6	0	-1.116804	2.703782	-0.179752
9	6	0	0.133898	2.008269	0.008747
10	6	0	1.322024	2.874032	0.119084
11	6	0	1.213290	4.265538	-0.159548
12	6	0	-0.090731	4.906372	-0.479840
13	6	0	-1.235770	4.050612	-0.414818
14	1	0	-0.413071	-2.101248	0.054881
15	1	0	-2.218878	4.474793	-0.586539
16	8	0	-2.301412	2.024076	-0.171441
17	7	0	-4.962682	-1.926484	-0.116304
18	6	0	-5.103347	-3.368786	0.086375
19	1	0	-6.044652	-3.675892	-0.379713
20	1	0	-5.184512	-3.601402	1.160638
21	6	0	-6.180101	-1.128949	0.029483
22	8	0	-0.167617	6.123606	-0.755537
23	6	0	1.327642	-0.247678	-0.116441
24	6	0	1.889196	-1.019847	0.918897
25	6	0	1.866364	-0.340768	-1.410911
26	6	0	2.967071	-1.860699	0.627205
27	6	0	2.948865	-1.171451	-1.678958
28	1	0	1.428437	0.246947	-2.212263
29	6	0	3.507184	-1.954148	-0.659960
30	1	0	3.373587	-2.413324	1.468087
31	1	0	3.369311	-1.229690	-2.676936
32	6	0	1.396578	-0.919738	2.372922
33	8	0	1.886671	-1.743211	3.187517
34	8	0	0.557401	-0.008675	2.605020
35	6	0	4.677014	-2.831084	-1.009976
36	8	0	5.355136	-2.629305	-2.025131
37	7	0	4.952783	-3.848732	-0.154364
38	6	0	6.039387	-4.781649	-0.403283
39	1	0	6.253039	-5.330072	0.515200
40	1	0	5.792021	-5.499298	-1.194876
41	1	0	6.931457	-4.231826	-0.709348
42	1	0	4.287041	-4.075182	0.568230
43	6	0	-3.931661	-4.129847	-0.524256
44	1	0	-4.055924	-5.200371	-0.335446
45	1	0	-3.932271	-3.983939	-1.610773
46	6	0	-4.849650	0.977487	-0.220031
47	6	0	-6.058945	0.189961	-0.726198
48	6	0	-2.622571	-3.618996	0.077857
49	1	0	-6.977099	0.771041	-0.598378

50	1	0	-5.950201	-0.021468	-1.796185
51	1	0	-5.074524	1.406233	0.766470
52	1	0	-4.645210	1.827817	-0.877057
53	1	0	-1.759342	-4.069798	-0.422549
54	1	0	-2.568104	-3.932969	1.129887
55	1	0	-7.012546	-1.720712	-0.362280
56	1	0	-6.391080	-0.938485	1.094305
57	6	0	2.341422	5.098421	-0.081529
58	6	0	2.584928	2.404576	0.548520
59	6	0	3.578406	4.601284	0.296422
60	1	0	4.443592	5.254947	0.355458
61	6	0	3.687232	3.244720	0.630062
62	1	0	4.637451	2.840783	0.967762
63	1	0	2.198496	6.148380	-0.313909
64	1	0	2.700226	1.374294	0.846462

064bba_Rh_Julolidin_naft1-H+carb_B3LYP631dp_PCMw.log

Standard orientation:

Center Number	Atomic Number	Atomic Type	Coordinates (Angstroms) X	Y	Z
1	6	0	-2.667735	-1.971904	0.382684
2	6	0	-1.406106	-1.411535	0.507455
3	6	0	-1.145358	-0.048341	0.295011
4	6	0	-2.234606	0.742197	-0.067738
5	6	0	-3.532559	0.234134	-0.231520
6	6	0	-3.760656	-1.140920	0.000911
7	6	0	0.230624	0.545688	0.505884
8	6	0	-0.862997	2.687289	-0.232176
9	6	0	0.297649	1.998575	0.148835
10	6	0	1.509105	2.794597	0.210645
11	6	0	1.483806	4.180531	-0.158926
12	6	0	0.240919	4.856920	-0.595925
13	6	0	-0.911975	4.033454	-0.593770
14	1	0	-0.589540	-2.068351	0.793192
15	1	0	-1.867959	4.454870	-0.888304
16	8	0	-2.101786	2.080922	-0.304614
17	7	0	-5.030284	-1.685383	-0.181672
18	6	0	-5.311964	-3.031969	0.308389
19	1	0	-6.225221	-3.379204	-0.185903
20	1	0	-5.515966	-3.026624	1.393575
21	6	0	-6.176707	-0.785429	-0.260712
22	8	0	0.236366	6.077508	-0.932465
23	6	0	1.293157	-0.369453	-0.088673
24	6	0	1.953350	-1.070108	0.914721
25	6	0	1.610535	-0.595544	-1.425388
26	6	0	2.937340	-2.014962	0.638130
27	6	0	2.600757	-1.532689	-1.716206
28	1	0	1.108124	-0.054083	-2.220578
29	6	0	3.258307	-2.257809	-0.702949
30	1	0	3.449588	-2.513441	1.454914
31	1	0	2.889144	-1.724139	-2.744012
32	6	0	1.467686	-0.587303	2.229004
33	8	0	1.822822	-0.939447	3.340397
34	8	0	0.536989	0.364242	2.020254
35	6	0	4.315730	-3.239544	-1.131163
36	8	0	4.887387	-3.131459	-2.221591
37	7	0	4.608538	-4.234919	-0.256269
38	6	0	5.593754	-5.257650	-0.571406
39	1	0	5.874989	-5.773494	0.347575
40	1	0	5.207134	-5.992587	-1.287436
41	1	0	6.478128	-4.789167	-1.006679
42	1	0	3.997627	-4.393071	0.530679
43	6	0	-4.150004	-3.973142	0.006200
44	1	0	-4.388020	-4.979491	0.364670
45	1	0	-4.012434	-4.033903	-1.079870
46	6	0	-4.653449	1.167319	-0.643951
47	6	0	-5.864391	0.395802	-1.173315

48	6	0	-2.878639	-3.445848	0.673459
49	1	0	-6.737262	1.052531	-1.242148
50	1	0	-5.658922	0.017227	-2.181455
51	1	0	-4.954816	1.783922	0.214737
52	1	0	-4.290259	1.871198	-1.399067
53	1	0	-2.003309	-4.018456	0.347332
54	1	0	-2.957272	-3.600448	1.759148
55	1	0	-7.023637	-1.358651	-0.650986
56	1	0	-6.466363	-0.422072	0.740905
57	6	0	2.658594	4.955127	-0.099703
58	6	0	2.764892	2.287946	0.651733
59	6	0	3.864714	4.424678	0.320205
60	1	0	4.760936	5.037389	0.361717
61	6	0	3.904333	3.072196	0.702542
62	1	0	4.835739	2.630616	1.048143
63	1	0	2.566249	5.995241	-0.396877
64	1	0	2.852697	1.258353	0.962726

071aaa_Rh_Julolidin_naft2_B3LYP631dp_PCMw.log

Standard orientation:

Center Number	Atomic Number	Atomic Type	Coordinates (Angstroms) X	Y	Z
1	6	0	0.587138	3.356794	0.033010
2	6	0	-0.171592	2.223520	-0.025997
3	6	0	0.404106	0.918611	-0.082270
4	6	0	1.832587	0.857807	-0.077906
5	6	0	2.653544	1.968447	-0.047267
6	6	0	2.036830	3.254634	0.010980
7	6	0	-0.331866	-0.267011	-0.122687
8	6	0	1.756292	-1.515198	-0.156348
9	6	0	0.350037	-1.517434	-0.172840
10	6	0	-0.315534	-2.785749	-0.237293
11	1	0	-1.398514	-2.803969	-0.259317
12	6	0	0.391508	-3.950995	-0.273298
13	6	0	1.824062	-3.953416	-0.247587
14	6	0	2.524800	-2.707194	-0.189273
15	1	0	-1.252035	2.312171	-0.016073
16	1	0	-0.128589	-4.902217	-0.322769
17	8	0	2.451001	-0.355780	-0.107169
18	7	0	2.793906	4.376166	0.046841
19	6	0	2.211831	5.724288	0.151626
20	1	0	2.896027	6.410996	-0.353019
21	1	0	2.175030	6.018664	1.209892
22	6	0	4.258059	4.302576	0.172160
23	6	0	-1.825419	-0.228483	-0.208160
24	6	0	-2.700681	-0.401889	0.887307
25	6	0	-2.375801	-0.018106	-1.481756
26	6	0	-4.085570	-0.379761	0.672377
27	6	0	-3.752357	0.012621	-1.675373
28	1	0	-1.710899	0.119899	-2.327799
29	6	0	-4.624394	-0.185841	-0.599249
30	1	0	-4.720397	-0.491088	1.543921
31	1	0	-4.166836	0.188971	-2.661502
32	6	0	-2.275948	-0.591455	2.309441
33	8	0	-3.055840	-0.783936	3.224376
34	8	0	-0.946461	-0.522718	2.496466
35	1	0	-0.778196	-0.662297	3.444956
36	6	0	-6.104774	-0.132292	-0.876150
37	8	0	-6.542481	0.477543	-1.856791
38	7	0	-6.911065	-0.775948	0.002531
39	6	0	-8.354099	-0.816216	-0.182234
40	1	0	-8.818695	-1.153715	0.744802
41	1	0	-8.719994	0.182895	-0.425641
42	1	0	-8.642394	-1.495039	-0.993223
43	1	0	-6.503568	-1.405197	0.677749
44	6	0	0.824519	5.794329	-0.470352
45	1	0	0.406502	6.789722	-0.297967

46	1	0	0.899136	5.649453	-1.553881
47	6	0	4.158132	1.817119	-0.075568
48	6	0	4.819730	3.100837	-0.575183
49	6	0	-0.062173	4.716078	0.148864
50	1	0	5.902097	3.058846	-0.426924
51	1	0	4.638408	3.226214	-1.648310
52	1	0	4.524195	1.575592	0.931229
53	1	0	4.433289	0.973385	-0.713948
54	1	0	-1.050200	4.694077	-0.319373
55	1	0	-0.221844	4.948112	1.210929
56	1	0	4.666919	5.232708	-0.228359
57	1	0	4.526061	4.257509	1.236973
58	6	0	3.944888	-2.708915	-0.163479
59	6	0	4.642785	-3.891133	-0.194296
60	1	0	5.726941	-3.904983	-0.173966
61	6	0	2.561097	-5.153941	-0.279905
62	6	0	3.948242	-5.126789	-0.252910
63	1	0	2.031691	-6.101459	-0.325273
64	1	0	4.478038	-1.766969	-0.115553
65	8	0	4.715350	-6.242208	-0.279153
66	1	0	4.155509	-7.032078	-0.315278

072aaa_Rh_Julolidin_naft2-H+carb_B3LYP631dp_PCMw.log

Standard orientation:

Center Number	Atomic Number	Atomic Type	Coordinates (Angstroms) X	Y	Z
1	6	0	-0.535742	-3.350080	0.040302
2	6	0	0.209012	-2.200958	0.032037
3	6	0	-0.378765	-0.906539	0.007563
4	6	0	-1.798704	-0.862335	-0.015772
5	6	0	-2.607624	-1.988752	-0.037973
6	6	0	-1.979239	-3.265863	-0.003666
7	6	0	0.360934	0.293530	0.050829
8	6	0	-1.754709	1.509593	-0.067087
9	6	0	-0.355647	1.533953	-0.042629
10	6	0	0.290914	2.810520	-0.087731
11	1	0	1.373821	2.840310	-0.065584
12	6	0	-0.428648	3.968202	-0.155062
13	6	0	-1.859629	3.949361	-0.178794
14	6	0	-2.540974	2.692814	-0.134964
15	1	0	1.290726	-2.274760	0.062503
16	1	0	0.079994	4.926580	-0.188628
17	8	0	-2.442825	0.341554	-0.034642
18	7	0	-2.728790	-4.403203	-0.023566
19	6	0	-2.128675	-5.739585	0.081336
20	1	0	-2.794118	-6.436330	-0.435874
21	1	0	-2.093623	-6.047144	1.136982
22	6	0	-4.190632	-4.344478	0.094102
23	6	0	1.842106	0.274885	-0.157225
24	6	0	2.725851	0.366949	0.926551
25	6	0	2.351489	0.166864	-1.460179
26	6	0	4.102289	0.366837	0.698937
27	6	0	3.726616	0.162217	-1.674962
28	1	0	1.671823	0.088207	-2.303254
29	6	0	4.617899	0.276496	-0.598564
30	1	0	4.741630	0.411722	1.575062
31	1	0	4.127914	0.071276	-2.678121
32	6	0	2.151990	0.448370	2.335777
33	8	0	2.947868	0.495372	3.299292
34	8	0	0.882253	0.459576	2.375487
35	6	0	6.090618	0.261173	-0.907375
36	8	0	6.522505	-0.222275	-1.960459
37	7	0	6.912517	0.799287	0.028875
38	6	0	8.351191	0.870512	-0.170993
39	1	0	8.829421	1.098413	0.782416
40	1	0	8.719698	-0.089677	-0.537101
41	1	0	8.625881	1.642615	-0.899512

42	1	0	6.512187	1.326578	0.789775
43	6	0	-0.733085	-5.780751	-0.527146
44	1	0	-0.303266	-6.774316	-0.371968
45	1	0	-0.799788	-5.614143	-1.608294
46	6	0	-4.113536	-1.852318	-0.101474
47	6	0	-4.753855	-3.132722	-0.637353
48	6	0	0.134203	-4.703603	0.122001
49	1	0	-5.839377	-3.103358	-0.508069
50	1	0	-4.552389	-3.234910	-1.709584
51	1	0	-4.508669	-1.629437	0.898871
52	1	0	-4.381669	-1.000507	-0.732623
53	1	0	1.122575	-4.655298	-0.344596
54	1	0	0.298430	-4.964048	1.176885
55	1	0	-4.591284	-5.269521	-0.327765
56	1	0	-4.475712	-4.319598	1.156214
57	6	0	-3.960364	2.673162	-0.158570
58	6	0	-4.676711	3.844036	-0.221610
59	1	0	-5.761188	3.839469	-0.238533
60	6	0	-2.616303	5.139018	-0.245469
61	6	0	-4.001192	5.090238	-0.264946
62	1	0	-2.100531	6.094825	-0.279643
63	1	0	-4.479671	1.722869	-0.122546
64	8	0	-4.786125	6.195909	-0.324673
65	1	0	-4.234779	6.992049	-0.343570

073aaa_Rh_Julolidin_naft2-H+_oh_B3LYP631dp_PCMw.log

Standard orientation:

Center Number	Atomic Number	Atomic Type	Coordinates (Angstroms) X	Y	Z
1	6	0	-0.416463	-3.285169	-0.074323
2	6	0	0.273323	-2.097363	-0.107353
3	6	0	-0.376253	-0.837238	-0.108870
4	6	0	-1.793772	-0.861228	-0.093934
5	6	0	-2.547304	-2.026631	-0.084664
6	6	0	-1.857641	-3.271769	-0.068525
7	6	0	0.290112	0.414929	-0.126071
8	6	0	-1.878891	1.525091	-0.122367
9	6	0	-0.452577	1.600892	-0.145609
10	6	0	0.131501	2.918865	-0.168651
11	1	0	1.212012	3.003735	-0.180335
12	6	0	-0.644033	4.033811	-0.175903
13	6	0	-2.086689	3.961592	-0.157171
14	6	0	-2.702375	2.651553	-0.127293
15	1	0	1.357823	-2.125306	-0.123837
16	1	0	-0.182257	5.016832	-0.193782
17	8	0	-2.495224	0.312580	-0.091712
18	7	0	-2.555558	-4.447637	-0.063769
19	6	0	-1.884085	-5.746763	0.048978
20	1	0	-2.532193	-6.491366	-0.421978
21	1	0	-1.785296	-6.027564	1.108740
22	6	0	-4.010170	-4.455622	0.117313
23	6	0	1.783987	0.457913	-0.215953
24	6	0	2.664071	0.350590	0.884247
25	6	0	2.335251	0.580050	-1.501203
26	6	0	4.049354	0.373008	0.666943
27	6	0	3.710725	0.599414	-1.701010
28	1	0	1.667566	0.653998	-2.353239
29	6	0	4.586319	0.507985	-0.612773
30	1	0	4.685314	0.257482	1.537150
31	1	0	4.122804	0.682936	-2.700341
32	6	0	2.247384	0.207231	2.314147
33	8	0	3.023237	-0.054254	3.216574
34	8	0	0.936639	0.411525	2.530213
35	1	0	0.781019	0.291183	3.483523
36	6	0	6.065295	0.520764	-0.898306
37	8	0	6.497800	0.209207	-2.012720
38	7	0	6.879772	0.880952	0.123593

39	6	0	8.322848	0.957321	-0.046675
40	1	0	8.792535	0.999159	0.936704
41	1	0	8.675600	0.071176	-0.577307
42	1	0	8.619847	1.842675	-0.620959
43	1	0	6.479065	1.291957	0.953092
44	6	0	-0.515341	-5.729852	-0.620034
45	1	0	-0.030025	-6.698929	-0.472556
46	1	0	-0.638474	-5.582388	-1.699089
47	6	0	-4.059594	-1.962888	-0.093131
48	6	0	-4.659379	-3.276141	-0.596297
49	6	0	0.326593	-4.602497	-0.024340
50	1	0	-5.739287	-3.296557	-0.424210
51	1	0	-4.496093	-3.375946	-1.675325
52	1	0	-4.427693	-1.750936	0.920014
53	1	0	-4.391439	-1.128826	-0.717796
54	1	0	1.284091	-4.506841	-0.545586
55	1	0	0.564083	-4.847390	1.020401
56	1	0	-4.386526	-5.400873	-0.282552
57	1	0	-4.254984	-4.435999	1.190309
58	6	0	-4.134300	2.575256	-0.103029
59	6	0	-4.900113	3.701763	-0.110437
60	1	0	-5.984744	3.635137	-0.091816
61	6	0	-2.878385	5.097402	-0.165211
62	6	0	-4.321528	5.046962	-0.142968
63	1	0	-2.412436	6.079475	-0.187988
64	1	0	-4.601934	1.596259	-0.076735
65	8	0	-5.044339	6.075733	-0.149909

074aaa_Rh_Julolidin_naft2-2H+_oh_B3LYP631dp_PCMw.log

Standard orientation:

Center Number	Atomic Number	Atomic Type	Coordinates (Angstroms) X	Y	Z
1	6	0	-0.513948	-3.298234	-0.006928
2	6	0	0.212956	-2.130971	-0.016740
3	6	0	-0.394770	-0.852074	-0.032071
4	6	0	-1.809078	-0.831146	-0.046889
5	6	0	-2.600023	-1.974779	-0.061663
6	6	0	-1.951865	-3.239435	-0.036549
7	6	0	0.321932	0.376864	-0.016353
8	6	0	-1.817340	1.552943	-0.084728
9	6	0	-0.395382	1.584326	-0.080830
10	6	0	0.228152	2.881913	-0.112918
11	1	0	1.311126	2.928997	-0.103165
12	6	0	-0.510327	4.022578	-0.151186
13	6	0	-1.953625	3.996152	-0.159086
14	6	0	-2.608431	2.707167	-0.124034
15	1	0	1.296322	-2.188188	0.000441
16	1	0	-0.016329	4.990014	-0.174400
17	8	0	-2.477285	0.362255	-0.055543
18	7	0	-2.689128	-4.395881	-0.060904
19	6	0	-2.060551	-5.711443	0.084229
20	1	0	-2.718706	-6.443228	-0.393554
21	1	0	-1.992718	-5.984658	1.148990
22	6	0	-4.143870	-4.355096	0.101369
23	6	0	1.810784	0.371306	-0.167792
24	6	0	2.695327	0.350142	0.925287
25	6	0	2.328770	0.375217	-1.474287
26	6	0	4.072051	0.343776	0.682221
27	6	0	3.701260	0.363834	-1.697483
28	1	0	1.646434	0.386625	-2.319220
29	6	0	4.592164	0.359482	-0.615817
30	1	0	4.708231	0.300433	1.560467
31	1	0	4.098560	0.359917	-2.706430
32	6	0	2.184709	0.331730	2.374043
33	8	0	3.055203	0.287285	3.279517
34	8	0	0.930675	0.365055	2.507759
35	6	0	6.064309	0.343793	-0.921976

36	8	0	6.491066	-0.026310	-2.022716
37	7	0	6.896350	0.750834	0.070650
38	6	0	8.336612	0.810597	-0.118058
39	1	0	8.818391	0.895874	0.856939
40	1	0	8.680187	-0.101211	-0.610313
41	1	0	8.634832	1.665844	-0.736457
42	1	0	6.504949	1.194068	0.887532
43	6	0	-0.676282	-5.741745	-0.552541
44	1	0	-0.225159	-6.725095	-0.390640
45	1	0	-0.770297	-5.595230	-1.634778
46	6	0	-4.109142	-1.861504	-0.106071
47	6	0	-4.741680	-3.154393	-0.622277
48	6	0	0.187269	-4.638542	0.057982
49	1	0	-5.825028	-3.139656	-0.471537
50	1	0	-4.560585	-3.258867	-1.698130
51	1	0	-4.494666	-1.635462	0.897650
52	1	0	-4.398650	-1.017465	-0.738666
53	1	0	1.156991	-4.573321	-0.445483
54	1	0	0.398556	-4.890125	1.106817
55	1	0	-4.547193	-5.287338	-0.303636
56	1	0	-4.405991	-4.325530	1.170627
57	6	0	-4.040351	2.676279	-0.130826
58	6	0	-4.770935	3.827290	-0.170684
59	1	0	-5.857589	3.792931	-0.174778
60	6	0	-2.711941	5.157649	-0.200615
61	6	0	-4.153006	5.153855	-0.209558
62	1	0	-2.212387	6.123277	-0.227897
63	1	0	-4.539969	1.713046	-0.100911
64	8	0	-4.847454	6.204779	-0.247856

074bba_Rh_Julolidin_naft2-H+carb_B3LYP631dp_PCMw_ring.log

Standard orientation:

Center Number	Atomic Number	Atomic Type	Coordinates (Angstroms) X	Y	Z
1	6	0	0.554927	3.327980	0.200926
2	6	0	-0.185437	2.170764	0.380183
3	6	0	0.381515	0.887776	0.349504
4	6	0	1.757643	0.812842	0.119121
5	6	0	2.558268	1.944207	-0.092877
6	6	0	1.955691	3.223395	-0.046189
7	6	0	-0.428021	-0.347252	0.627679
8	6	0	1.717652	-1.567191	0.131699
9	6	0	0.346979	-1.601153	0.353201
10	6	0	-0.281840	-2.880882	0.389693
11	1	0	-1.352351	-2.928377	0.567596
12	6	0	0.424847	-4.039815	0.209206
13	6	0	1.840331	-4.023178	-0.023423
14	6	0	2.492822	-2.739885	-0.063463
15	1	0	-1.252291	2.267972	0.564121
16	1	0	-0.086432	-4.998623	0.242257
17	8	0	2.422020	-0.387776	0.072267
18	7	0	2.714873	4.365910	-0.280480
19	6	0	2.152705	5.682335	0.011880
20	1	0	2.755407	6.424447	-0.521715
21	1	0	2.231829	5.917343	1.087591
22	6	0	4.170961	4.272237	-0.230487
23	6	0	-1.814490	-0.326646	0.006038
24	6	0	-2.789003	-0.314849	0.995714
25	6	0	-2.180044	-0.313264	-1.338760
26	6	0	-4.150075	-0.300364	0.699906
27	6	0	-3.538363	-0.291582	-1.648784
28	1	0	-1.432515	-0.316776	-2.125336
29	6	0	-4.529308	-0.298942	-0.646770
30	1	0	-4.876363	-0.263736	1.505539
31	1	0	-3.864141	-0.268043	-2.682916
32	6	0	-2.123097	-0.314377	2.317344
33	8	0	-2.629991	-0.299771	3.423694

34	8	0	-0.786363	-0.335735	2.122308
35	6	0	-5.965734	-0.265507	-1.097053
36	8	0	-6.279231	0.190725	-2.201829
37	7	0	-6.884725	-0.751719	-0.225143
38	6	0	-8.300117	-0.796234	-0.558143
39	1	0	-8.875794	-0.929878	0.358699
40	1	0	-8.590839	0.142271	-1.033002
41	1	0	-8.533449	-1.616620	-1.247199
42	1	0	-6.571375	-1.279830	0.575272
43	6	0	0.694620	5.761637	-0.428907
44	1	0	0.298273	6.758177	-0.210924
45	1	0	0.639801	5.615023	-1.514068
46	6	0	4.040213	1.773736	-0.361260
47	6	0	4.651529	3.033449	-0.978526
48	6	0	-0.116360	4.684713	0.293175
49	1	0	5.744383	2.982629	-0.949122
50	1	0	4.355779	3.119709	-2.030585
51	1	0	4.559789	1.532552	0.576771
52	1	0	4.199860	0.913195	-1.018210
53	1	0	-1.131993	4.625434	-0.113102
54	1	0	-0.225665	4.970207	1.349170
55	1	0	4.578132	5.179235	-0.688258
56	1	0	4.530858	4.246779	0.812771
57	6	0	3.899244	-2.714490	-0.297154
58	6	0	4.608910	-3.872420	-0.477030
59	1	0	5.682356	-3.833302	-0.653665
60	6	0	2.587193	-5.195659	-0.213650
61	6	0	3.997669	-5.199764	-0.447090
62	1	0	2.075190	-6.156348	-0.182362
63	1	0	4.406864	-1.754978	-0.328397
64	8	0	4.690150	-6.252130	-0.618414

081aaa_Rh_Julolidin_EtCOO_B3LYP631dp_PCMw.log

Standard orientation:

Center Number	Atomic Number	Atomic Type	Coordinates (Angstroms) X	Y	Z
1	6	0	3.664011	1.369300	-0.095919
2	6	0	2.305686	1.234314	-0.103844
3	6	0	1.660073	-0.038911	-0.097592
4	6	0	2.518309	-1.183725	-0.091252
5	6	0	3.898452	-1.109750	-0.106714
6	6	0	4.502701	0.183292	-0.107651
7	6	0	0.274004	-0.215382	-0.079982
8	6	0	0.636161	-2.627490	-0.067166
9	6	0	-0.262832	-1.532644	-0.075500
10	6	0	-1.652351	-1.829503	-0.071587
11	1	0	-2.351190	-1.002748	-0.077191
12	6	0	-2.137116	-3.120935	-0.057999
13	6	0	-1.180557	-4.184009	-0.047225
14	6	0	0.191757	-3.941060	-0.054266
15	1	0	1.688883	2.126240	-0.100520
16	1	0	0.896710	-4.763406	-0.049615
17	8	0	1.980605	-2.434410	-0.070648
18	7	0	5.851132	0.309179	-0.119891
19	6	0	6.525782	1.616541	-0.076854
20	1	0	7.475473	1.507544	-0.606830
21	1	0	6.761949	1.865872	0.967294
22	6	0	6.726853	-0.864920	0.016692
23	8	0	-1.561850	-5.477196	-0.029352
24	1	0	-2.528300	-5.544916	-0.041027
25	6	0	-0.642450	0.965455	-0.162981
26	6	0	-1.217307	1.622233	0.947651
27	6	0	-0.954175	1.425993	-1.451375
28	6	0	-2.068478	2.715952	0.736348
29	6	0	-1.806548	2.507554	-1.643374
30	1	0	-0.522166	0.923428	-2.310357
31	6	0	-2.363479	3.175058	-0.546818

32	1	0	-2.507011	3.173391	1.615841
33	1	0	-2.049835	2.846907	-2.643829
34	6	0	-1.001007	1.247753	2.380042
35	8	0	-1.561045	1.797930	3.310818
36	8	0	-0.122049	0.246350	2.557073
37	1	0	-0.059289	0.080621	3.514139
38	6	0	-3.287485	4.333030	-0.824381
39	8	0	-3.870409	4.430026	-1.908833
40	7	0	-3.440931	5.238225	0.172496
41	6	0	-4.286496	6.411723	0.013380
42	1	0	-4.521161	6.815486	0.999081
43	1	0	-3.796731	7.190559	-0.582824
44	1	0	-5.211706	6.125890	-0.489363
45	1	0	-2.814177	5.211119	0.962572
46	6	0	5.680890	2.713313	-0.709303
47	1	0	6.190723	3.672541	-0.584925
48	1	0	5.582154	2.527725	-1.784673
49	6	0	4.735328	-2.369703	-0.118744
50	6	0	6.131401	-2.085637	-0.671553
51	6	0	4.305002	2.736866	-0.047204
52	1	0	6.790044	-2.944042	-0.514250
53	1	0	6.080633	-1.902465	-1.750533
54	1	0	4.812436	-2.770325	0.900928
55	1	0	4.236273	-3.139469	-0.713059
56	1	0	3.646622	3.469957	-0.521637
57	1	0	4.416606	3.045440	1.001435
58	1	0	7.691922	-0.605004	-0.423716
59	1	0	6.896552	-1.065485	1.083823
60	6	0	-3.612169	-3.463547	-0.042321
61	1	0	-3.850215	-4.163554	-0.855103
62	1	0	-3.835695	-4.009448	0.887070
63	6	0	-4.592869	-2.298687	-0.171778
64	1	0	-4.522046	-1.618678	0.683770
65	1	0	-4.343611	-1.703359	-1.061898
66	6	0	-6.077736	-2.754828	-0.322588
67	8	0	-6.943298	-1.906388	0.017652
68	8	0	-6.272629	-3.907250	-0.796922

081aba_Rh_Julolidin_EtCOO_B3LYP631dp_PCMw.log

Standard orientation:

Center Number	Atomic Number	Atomic Type	Coordinates (Angstroms) X	Y	Z
1	6	0	3.668511	1.396778	-0.069831
2	6	0	2.310763	1.255508	-0.084377
3	6	0	1.671263	-0.020733	-0.091974
4	6	0	2.535266	-1.160936	-0.088688
5	6	0	3.915225	-1.080547	-0.097637
6	6	0	4.513090	0.215230	-0.087792
7	6	0	0.285721	-0.204684	-0.083610
8	6	0	0.660108	-2.612686	-0.082001
9	6	0	-0.244549	-1.524518	-0.089426
10	6	0	-1.633576	-1.829929	-0.097701
11	1	0	-2.336074	-1.006451	-0.106235
12	6	0	-2.112604	-3.122692	-0.093588
13	6	0	-1.149549	-4.179725	-0.080707
14	6	0	0.221512	-3.930673	-0.078102
15	1	0	1.689626	2.144341	-0.075431
16	1	0	0.945440	-4.738805	-0.073087
17	8	0	2.002901	-2.414348	-0.077760
18	7	0	5.861145	0.347891	-0.094987
19	6	0	6.529288	1.658121	-0.041125
20	1	0	7.480349	1.557763	-0.570444
21	1	0	6.762687	1.900924	1.005197
22	6	0	6.741989	-0.822693	0.037376
23	8	0	-1.644466	-5.433831	-0.072807
24	1	0	-0.924483	-6.082204	-0.065344
25	6	0	-0.635634	0.972031	-0.165653

26	6	0	-1.271975	1.578496	0.940106
27	6	0	-0.893665	1.477032	-1.449211
28	6	0	-2.131939	2.665387	0.729733
29	6	0	-1.753244	2.552929	-1.640756
30	1	0	-0.415805	1.012171	-2.305103
31	6	0	-2.373203	3.169166	-0.548028
32	1	0	-2.619135	3.081678	1.604095
33	1	0	-1.954485	2.927163	-2.638110
34	6	0	-1.109882	1.157595	2.366647
35	8	0	-1.753947	1.631107	3.285100
36	8	0	-0.174056	0.210968	2.554011
37	1	0	-0.153227	0.007405	3.505582
38	6	0	-3.301258	4.323499	-0.827343
39	8	0	-3.827292	4.456404	-1.936773
40	7	0	-3.523835	5.184984	0.194791
41	6	0	-4.379920	6.350853	0.035070
42	1	0	-4.661927	6.720318	1.021756
43	1	0	-3.877119	7.154484	-0.515613
44	1	0	-5.279009	6.068640	-0.515156
45	1	0	-2.940647	5.136288	1.016517
46	6	0	5.680128	2.755598	-0.666654
47	1	0	6.184855	3.716380	-0.533689
48	1	0	5.584434	2.578030	-1.743662
49	6	0	4.758258	-2.336347	-0.114515
50	6	0	6.154868	-2.041931	-0.660659
51	6	0	4.302715	2.766899	-0.007305
52	1	0	6.817126	-2.898144	-0.506590
53	1	0	6.106840	-1.852097	-1.738615
54	1	0	4.833835	-2.742947	0.902899
55	1	0	4.265215	-3.104867	-0.715451
56	1	0	3.641938	3.501095	-0.476660
57	1	0	4.410686	3.066622	1.044274
58	1	0	7.707365	-0.555439	-0.397998
59	1	0	6.909056	-1.029311	1.103793
60	6	0	-3.582919	-3.477836	-0.099331
61	1	0	-3.789795	-4.157431	-0.932978
62	1	0	-3.792983	-4.077585	0.796923
63	6	0	-4.568245	-2.314186	-0.178549
64	1	0	-4.485647	-1.656391	0.694069
65	1	0	-4.337881	-1.689179	-1.054079
66	6	0	-6.056836	-2.761579	-0.316394
67	8	0	-6.914727	-1.918188	0.058009
68	8	0	-6.267245	-3.901255	-0.814445

082aba_Rh_Julolidin_EtCOO-H+carb_B3LYP631dp_PCMw.log

Standard orientation:

Center Number	Atomic Number	Atomic Type	Coordinates (Angstroms) X	Y	Z
1	6	0	3.660609	1.370531	-0.069830
2	6	0	2.298032	1.235024	-0.045493
3	6	0	1.651608	-0.031316	-0.025356
4	6	0	2.501871	-1.170762	-0.040458
5	6	0	3.886800	-1.098561	-0.087730
6	6	0	4.493429	0.188920	-0.097628
7	6	0	0.251939	-0.195309	0.054911
8	6	0	0.621660	-2.612265	-0.025244
9	6	0	-0.275603	-1.524784	-0.004147
10	6	0	-1.663030	-1.825186	-0.018511
11	1	0	-2.359467	-0.996667	0.003501
12	6	0	-2.150624	-3.116338	-0.056032
13	6	0	-1.194521	-4.175619	-0.073292
14	6	0	0.175870	-3.930302	-0.060117
15	1	0	1.681654	2.127463	-0.025235
16	1	0	0.897354	-4.740730	-0.079663
17	8	0	1.969126	-2.427355	-0.019620
18	7	0	5.850280	0.311024	-0.145114
19	6	0	6.521012	1.615807	-0.084987

20	1	0	7.473226	1.517145	-0.613816
21	1	0	6.755521	1.863960	0.960935
22	6	0	6.717406	-0.864400	-0.004403
23	8	0	-1.693809	-5.432728	-0.107198
24	1	0	-0.972193	-6.078646	-0.118542
25	6	0	-0.658407	0.977187	-0.126617
26	6	0	-1.217463	1.643260	0.973208
27	6	0	-0.966608	1.406583	-1.426787
28	6	0	-2.066395	2.730599	0.762499
29	6	0	-1.819217	2.488659	-1.623846
30	1	0	-0.539768	0.891832	-2.282282
31	6	0	-2.370581	3.170842	-0.530084
32	1	0	-2.490220	3.191858	1.649085
33	1	0	-2.068139	2.819830	-2.625813
34	6	0	-0.887061	1.163391	2.383239
35	8	0	-1.409788	1.766884	3.347402
36	8	0	-0.092462	0.175362	2.423779
37	6	0	-3.286861	4.328802	-0.819925
38	8	0	-3.846377	4.447980	-1.916389
39	7	0	-3.467280	5.224725	0.183454
40	6	0	-4.304549	6.401643	0.016185
41	1	0	-4.554992	6.801857	0.999666
42	1	0	-3.802881	7.183247	-0.567072
43	1	0	-5.222898	6.124338	-0.504251
44	1	0	-2.869256	5.178803	0.994188
45	6	0	5.672987	2.715361	-0.710869
46	1	0	6.184381	3.674732	-0.591444
47	1	0	5.566519	2.528694	-1.785562
48	6	0	4.718280	-2.362605	-0.126107
49	6	0	6.111094	-2.082152	-0.690269
50	6	0	4.300928	2.740759	-0.039676
51	1	0	6.766985	-2.944885	-0.543369
52	1	0	6.050855	-1.894149	-1.768155
53	1	0	4.804676	-2.779240	0.886648
54	1	0	4.208716	-3.122710	-0.724557
55	1	0	3.638824	3.468418	-0.518311
56	1	0	4.419428	3.064272	1.003846
57	1	0	7.684273	-0.614025	-0.448058
58	1	0	6.891588	-1.070835	1.062013
59	6	0	-3.623855	-3.463321	-0.074175
60	1	0	-3.832536	-4.122406	-0.923656
61	1	0	-3.842292	-4.081708	0.807481
62	6	0	-4.603258	-2.293047	-0.130565
63	1	0	-4.519581	-1.654909	0.756492
64	1	0	-4.365421	-1.649105	-0.990325
65	6	0	-6.094303	-2.726327	-0.282928
66	8	0	-6.948063	-1.882365	0.100725
67	8	0	-6.312689	-3.856170	-0.800191

082bba_Rh_Julolidin_EtCOO-H+carb_B3LYP631dp_PCMw_ring.log

Standard orientation:

Center Number	Atomic Number	Atomic Type	Coordinates (Angstroms) X	Y	Z
1	6	0	3.720860	1.227386	0.255026
2	6	0	2.348072	1.129217	0.409646
3	6	0	1.650223	-0.083269	0.300767
4	6	0	2.409782	-1.221130	0.014598
5	6	0	3.797598	-1.186505	-0.174092
6	6	0	4.470701	0.052158	-0.047537
7	6	0	0.169041	-0.165889	0.557649
8	6	0	0.474363	-2.589222	-0.071653
9	6	0	-0.381335	-1.524514	0.208344
10	6	0	-1.765471	-1.784556	0.215939
11	1	0	-2.439078	-0.964499	0.438705
12	6	0	-2.309088	-3.036270	-0.043993
13	6	0	-1.397778	-4.085660	-0.320408
14	6	0	-0.024489	-3.867990	-0.338511

15	1	0	1.792132	2.035017	0.636985
16	1	0	0.671573	-4.671771	-0.558751
17	8	0	1.837868	-2.467230	-0.116211
18	7	0	5.842303	0.129798	-0.256692
19	6	0	6.566551	1.346622	0.102994
20	1	0	7.524818	1.328520	-0.426050
21	1	0	6.795397	1.366797	1.182506
22	6	0	6.631367	-1.098337	-0.282716
23	8	0	-1.935047	-5.313760	-0.572576
24	1	0	-1.226651	-5.947723	-0.753754
25	6	0	-0.621561	0.988625	-0.035447
26	6	0	-1.192355	1.745198	0.979873
27	6	0	-0.825410	1.334249	-1.369675
28	6	0	-1.971814	2.870350	0.721275
29	6	0	-1.610225	2.452922	-1.642374
30	1	0	-0.392404	0.751505	-2.176112
31	6	0	-2.175555	3.233537	-0.614220
32	1	0	-2.420637	3.415938	1.544991
33	1	0	-1.805663	2.748418	-2.667277
34	6	0	-0.832943	1.139032	2.280185
35	8	0	-1.137471	1.495090	3.401364
36	8	0	-0.058839	0.051068	2.042747
37	6	0	-3.014753	4.413610	-1.028791
38	8	0	-3.545074	4.460001	-2.143744
39	7	0	-3.159231	5.405620	-0.115188
40	6	0	-3.923028	6.607369	-0.414083
41	1	0	-4.127065	7.136660	0.517401
42	1	0	-3.382042	7.277175	-1.092833
43	1	0	-4.867845	6.332446	-0.886400
44	1	0	-2.567296	5.412706	0.701524
45	6	0	5.768847	2.589045	-0.280195
46	1	0	6.333610	3.486419	-0.009192
47	1	0	5.628175	2.603532	-1.367398
48	6	0	4.543388	-2.464245	-0.503944
49	6	0	5.921711	-2.172125	-1.100248
50	6	0	4.414156	2.564736	0.429151
51	1	0	6.529490	-3.081905	-1.124129
52	1	0	5.818605	-1.819381	-2.133042
53	1	0	4.655707	-3.071262	0.405417
54	1	0	3.950131	-3.071309	-1.194345
55	1	0	3.767625	3.368779	0.061132
56	1	0	4.568936	2.762465	1.499400
57	1	0	7.603435	-0.859333	-0.725201
58	1	0	6.823545	-1.467046	0.739656
59	6	0	-3.796118	-3.327919	-0.046414
60	1	0	-4.074589	-3.801453	-0.994246
61	1	0	-4.001132	-4.097611	0.711397
62	6	0	-4.730687	-2.139745	0.174519
63	1	0	-4.591886	-1.695675	1.166980
64	1	0	-4.496043	-1.344605	-0.549216
65	6	0	-6.241549	-2.483605	-0.002856
66	8	0	-7.047821	-1.746817	0.626769
67	8	0	-6.524536	-3.435751	-0.781080

083aba_Rh_Julolidin_EtCOO-H+oh_B3LYP631dp_PCMw.log

Standard orientation:

Center Number	Atomic Number	Atomic Type	Coordinates (Angstroms) X	Y	Z
1	6	0	3.653069	1.332882	-0.125027
2	6	0	2.283838	1.187430	-0.124719
3	6	0	1.649848	-0.077243	-0.107563
4	6	0	2.501010	-1.207267	-0.101244
5	6	0	3.889245	-1.125000	-0.121085
6	6	0	4.488655	0.163763	-0.130048
7	6	0	0.235658	-0.274054	-0.088641
8	6	0	0.621565	-2.681667	-0.072085
9	6	0	-0.284638	-1.561693	-0.084794

10	6	0	-1.691889	-1.872396	-0.078890
11	1	0	-2.383199	-1.037326	-0.086017
12	6	0	-2.173075	-3.147258	-0.064154
13	6	0	-1.227388	-4.290576	-0.046504
14	6	0	0.184768	-3.975393	-0.054567
15	1	0	1.666395	2.080067	-0.128207
16	1	0	0.897692	-4.792917	-0.045842
17	8	0	1.974240	-2.469088	-0.075039
18	7	0	5.855943	0.290844	-0.163239
19	6	0	6.505741	1.598310	-0.046863
20	1	0	7.488484	1.519421	-0.521567
21	1	0	6.678305	1.845605	1.012676
22	6	0	6.715203	-0.876487	0.041133
23	8	0	-1.641426	-5.471211	-0.026341
24	6	0	-0.671619	0.915478	-0.169063
25	6	0	-1.184786	1.624563	0.940412
26	6	0	-1.036500	1.341088	-1.456314
27	6	0	-2.028412	2.725316	0.728558
28	6	0	-1.876777	2.431044	-1.650630
29	1	0	-0.652504	0.799613	-2.314606
30	6	0	-2.372936	3.147015	-0.554782
31	1	0	-2.422367	3.218292	1.609933
32	1	0	-2.158966	2.739864	-2.650898
33	6	0	-0.920431	1.298813	2.376763
34	8	0	-1.458229	1.876632	3.305751
35	8	0	-0.025399	0.315103	2.568621
36	6	0	-3.286909	4.312091	-0.830317
37	8	0	-3.907920	4.393156	-1.895238
38	7	0	-3.390146	5.246707	0.146651
39	6	0	-4.219872	6.430690	-0.015621
40	1	0	-4.408387	6.868627	0.965438
41	1	0	-3.740475	7.182009	-0.654248
42	1	0	-5.169301	6.147955	-0.473064
43	1	0	-2.736391	5.228916	0.914677
44	6	0	5.678272	2.692704	-0.710736
45	1	0	6.177340	3.656670	-0.574566
46	1	0	5.618944	2.500430	-1.788253
47	6	0	4.726527	-2.386440	-0.128931
48	6	0	6.137942	-2.107644	-0.647483
49	6	0	4.277677	2.712177	-0.099254
50	1	0	6.790667	-2.966847	-0.467269
51	1	0	6.114519	-1.933820	-1.729360
52	1	0	4.780420	-2.800956	0.887249
53	1	0	4.238243	-3.150778	-0.740120
54	1	0	3.629790	3.421723	-0.623704
55	1	0	4.347012	3.064883	0.939481
56	1	0	7.701238	-0.635628	-0.365912
57	1	0	6.846959	-1.068729	1.117737
58	6	0	-3.641249	-3.493969	-0.062706
59	1	0	-3.847971	-4.176753	-0.894520
60	1	0	-3.844887	-4.103806	0.829168
61	6	0	-4.632331	-2.334824	-0.131839
62	1	0	-4.542463	-1.676768	0.740518
63	1	0	-4.406605	-1.705487	-1.006257
64	6	0	-6.122617	-2.773706	-0.260771
65	8	0	-6.976247	-1.935544	0.138726
66	8	0	-6.346371	-3.902458	-0.779000
67	1	0	0.062207	0.193689	3.530387

084aba_Rh_Julolidin_EtCOO-2H_B3LYP631dp_PCMw.log

Standard orientation:

Center Number	Atomic Number	Atomic Type	Coordinates (Angstroms) X	Y	Z
1	6	0	3.641383	1.286390	-0.102198
2	6	0	2.269948	1.152518	-0.071780
3	6	0	1.623700	-0.103982	-0.049452
4	6	0	2.462810	-1.239424	-0.071097

5	6	0	3.853607	-1.169733	-0.120747
6	6	0	4.464494	0.111291	-0.133000
7	6	0	0.202595	-0.279731	0.000780
8	6	0	0.572439	-2.695406	-0.042034
9	6	0	-0.320893	-1.570277	-0.031444
10	6	0	-1.728623	-1.872908	-0.021509
11	1	0	-2.411455	-1.031024	-0.002334
12	6	0	-2.220996	-3.145023	-0.030658
13	6	0	-1.287109	-4.294921	-0.043929
14	6	0	0.125110	-3.988438	-0.050106
15	1	0	1.658395	2.048908	-0.052699
16	1	0	0.833617	-4.810097	-0.063783
17	8	0	1.928629	-2.498889	-0.048507
18	7	0	5.836124	0.225697	-0.201026
19	6	0	6.495523	1.524864	-0.064346
20	1	0	7.475964	1.447829	-0.544696
21	1	0	6.674901	1.756762	0.998190
22	6	0	6.682532	-0.950281	-0.001936
23	8	0	-1.710877	-5.475288	-0.050737
24	6	0	-0.691323	0.914685	-0.124504
25	6	0	-1.116043	1.691104	0.970607
26	6	0	-1.107540	1.268403	-1.420265
27	6	0	-1.931832	2.802256	0.732702
28	6	0	-1.921915	2.374268	-1.636163
29	1	0	-0.784454	0.666330	-2.264586
30	6	0	-2.334537	3.165726	-0.556098
31	1	0	-2.253015	3.346144	1.615289
32	1	0	-2.245031	2.638646	-2.636989
33	6	0	-0.732264	1.352989	2.422829
34	8	0	-1.196087	2.112480	3.312744
35	8	0	0.010541	0.347579	2.577510
36	6	0	-3.213433	4.349452	-0.850060
37	8	0	-3.846661	4.438130	-1.909526
38	7	0	-3.277913	5.311583	0.106120
39	6	0	-4.065581	6.519216	-0.078253
40	1	0	-4.203074	7.004424	0.889095
41	1	0	-3.580897	7.225757	-0.763007
42	1	0	-5.041712	6.259940	-0.492497
43	1	0	-2.629432	5.278511	0.877544
44	6	0	5.671593	2.634762	-0.707363
45	1	0	6.180999	3.592932	-0.567336
46	1	0	5.599188	2.453561	-1.786116
47	6	0	4.676894	-2.440343	-0.157575
48	6	0	6.085556	-2.172671	-0.690044
49	6	0	4.277654	2.661222	-0.080600
50	1	0	6.731357	-3.039826	-0.522455
51	1	0	6.052045	-1.992001	-1.770634
52	1	0	4.738478	-2.870834	0.851633
53	1	0	4.171916	-3.190730	-0.772826
54	1	0	3.629446	3.377288	-0.596146
55	1	0	4.361051	3.012061	0.957825
56	1	0	7.669385	-0.721054	-0.414436
57	1	0	6.819334	-1.148851	1.073525
58	6	0	-3.693043	-3.479661	-0.024880
59	1	0	-3.910333	-4.151920	-0.862419
60	1	0	-3.899674	-4.096558	0.861818
61	6	0	-4.675764	-2.312285	-0.077570
62	1	0	-4.579468	-1.665545	0.802624
63	1	0	-4.445568	-1.673026	-0.943695
64	6	0	-6.169887	-2.736205	-0.210129
65	8	0	-7.016526	-1.894611	0.197889
66	8	0	-6.405242	-3.857617	-0.739576

084bba_Rh_Julolidin_EtCOO-2H+carb_B3LYP631dp_PCMw_ring.log

Standard orientation:

Center Number	Atomic Number	Atomic Type	Coordinates (Angstroms) X	Y	Z

1	6	0	3.693928	1.239785	0.168286
2	6	0	2.320991	1.128696	0.322499
3	6	0	1.638077	-0.095324	0.260432
4	6	0	2.411959	-1.238608	0.027455
5	6	0	3.802858	-1.187913	-0.154539
6	6	0	4.458560	0.062740	-0.079244
7	6	0	0.161586	-0.199090	0.515908
8	6	0	0.481061	-2.632315	-0.039408
9	6	0	-0.373919	-1.542903	0.180168
10	6	0	-1.766143	-1.823356	0.159376
11	1	0	-2.447361	-0.995801	0.339580
12	6	0	-2.290414	-3.078413	-0.070643
13	6	0	-1.395395	-4.214140	-0.300810
14	6	0	0.007934	-3.913792	-0.277366
15	1	0	1.752790	2.035784	0.511909
16	1	0	0.717746	-4.716429	-0.458147
17	8	0	1.860804	-2.489436	-0.049647
18	7	0	5.833971	0.149268	-0.289260
19	6	0	6.536978	1.385008	0.044523
20	1	0	7.507168	1.360287	-0.462533
21	1	0	6.742331	1.446697	1.127823
22	6	0	6.634419	-1.069752	-0.230745
23	8	0	-1.843556	-5.385185	-0.515760
24	6	0	-0.645294	0.963222	-0.033721
25	6	0	-1.189082	1.724794	0.992888
26	6	0	-0.887086	1.308373	-1.362793
27	6	0	-1.973780	2.849411	0.749918
28	6	0	-1.675064	2.428045	-1.620471
29	1	0	-0.478304	0.719165	-2.177366
30	6	0	-2.212522	3.212354	-0.580350
31	1	0	-2.400690	3.396352	1.584614
32	1	0	-1.895429	2.720905	-2.641199
33	6	0	-0.799370	1.127903	2.292447
34	8	0	-1.094876	1.513120	3.412265
35	8	0	-0.035907	0.049298	2.059661
36	6	0	-3.056836	4.394535	-0.973768
37	8	0	-3.606769	4.451035	-2.079249
38	7	0	-3.185004	5.382255	-0.051893
39	6	0	-3.950554	6.587154	-0.331483
40	1	0	-4.135946	7.113266	0.605752
41	1	0	-3.420834	7.258848	-1.017405
42	1	0	-4.904912	6.317338	-0.787398
43	1	0	-2.579600	5.382912	0.754813
44	6	0	5.733605	2.603660	-0.398943
45	1	0	6.285338	3.517171	-0.155740
46	1	0	5.610008	2.573064	-1.487999
47	6	0	4.565945	-2.469889	-0.422554
48	6	0	5.952655	-2.191779	-1.006411
49	6	0	4.368146	2.593169	0.290151
50	1	0	6.570920	-3.094452	-0.974355
51	1	0	5.866306	-1.891182	-2.057208
52	1	0	4.666833	-3.041867	0.510868
53	1	0	3.988038	-3.108498	-1.097645
54	1	0	3.718023	3.371443	-0.124650
55	1	0	4.504512	2.843852	1.351988
56	1	0	7.614878	-0.845040	-0.662635
57	1	0	6.806212	-1.383450	0.814011
58	6	0	-3.774320	-3.373567	-0.100821
59	1	0	-4.026045	-3.866843	-1.047402
60	1	0	-3.989669	-4.145544	0.654148
61	6	0	-4.730112	-2.196953	0.092345
62	1	0	-4.588597	-1.721996	1.070782
63	1	0	-4.506262	-1.416374	-0.651914
64	6	0	-6.238694	-2.552255	-0.062124
65	8	0	-7.044681	-1.839085	0.598337
66	8	0	-6.532629	-3.490130	-0.855087

121aaa_Rh_Julolidin5COOH_OH_B3LYP631dp_PCMw.log

Standard orientation:

Center Number	Atomic Number	Atomic Type	Coordinates (Angstroms) X	Y	Z
1	6	0	-2.455626	-1.834643	0.373655
2	6	0	-1.178299	-1.359164	0.447027
3	6	0	-0.844682	-0.004799	0.141746
4	6	0	-1.929589	0.839451	-0.256003
5	6	0	-3.237850	0.408367	-0.373293
6	6	0	-3.527287	-0.952390	-0.057443
7	6	0	0.445760	0.522556	0.224133
8	6	0	-0.447689	2.677315	-0.488805
9	6	0	0.666045	1.889684	-0.107028
10	6	0	1.941192	2.518085	-0.075143
11	1	0	2.814915	1.941650	0.206752
12	6	0	2.070445	3.846227	-0.401632
13	6	0	0.926255	4.612634	-0.779476
14	6	0	-0.329213	4.023978	-0.822638
15	1	0	-0.384762	-2.027532	0.762130
16	1	0	-1.199275	4.600493	-1.111394
17	8	0	-1.695761	2.147648	-0.547227
18	7	0	-4.792447	-1.424954	-0.157631
19	6	0	-5.154570	-2.807855	0.193085
20	1	0	-6.001049	-3.089752	-0.438327
21	1	0	-5.501737	-2.835412	1.235588
22	6	0	-5.919904	-0.524762	-0.444563
23	8	0	1.070142	5.909953	-1.097009
24	1	0	2.010024	6.146058	-1.012381
25	6	0	1.610423	-0.356323	0.561168
26	6	0	2.146626	-0.527970	1.858614
27	6	0	2.208902	-1.028525	-0.509173
28	6	0	3.261117	-1.360395	2.031069
29	6	0	3.309838	-1.871190	-0.326353
30	1	0	1.824002	-0.899045	-1.514430
31	6	0	3.841917	-2.026120	0.958844
32	1	0	3.665943	-1.468422	3.030276
33	6	0	1.622637	0.117444	3.102835
34	8	0	2.135099	-0.019507	4.198580
35	8	0	0.523302	0.866534	2.908194
36	1	0	0.268880	1.239569	3.770504
37	6	0	3.879587	-2.533689	-1.556208
38	8	0	3.682394	-2.059436	-2.679284
39	7	0	4.613533	-3.653914	-1.354755
40	6	0	5.192741	-4.392713	-2.466790
41	1	0	5.954110	-5.071461	-2.081063
42	1	0	4.436976	-4.974738	-3.006800
43	1	0	5.653313	-3.694434	-3.167737
44	1	0	4.605679	-4.094433	-0.447242
45	6	0	-3.992052	-3.769350	-0.008257
46	1	0	-4.286846	-4.762730	0.340711
47	1	0	-3.760775	-3.848900	-1.076359
48	6	0	-4.323086	1.360156	-0.825636
49	6	0	-5.512432	0.589964	-1.398256
50	6	0	-2.774689	-3.259876	0.760520
51	1	0	-6.363788	1.257611	-1.555836
52	1	0	-5.249729	0.156096	-2.369387
53	1	0	-4.650901	1.975384	0.023046
54	1	0	-3.920922	2.053444	-1.568958
55	1	0	-1.900388	-3.893950	0.588687
56	1	0	-2.987016	-3.301267	1.837801
57	1	0	-6.719480	-1.130331	-0.876742
58	1	0	-6.297261	-0.108144	0.499756
59	8	0	3.229940	4.559022	-0.408915
60	1	0	3.981486	4.009915	-0.144092
61	1	0	4.720943	-2.637877	1.131495

121aba_Rh_Julolidin5COOH_OH_B3LYP631dp_PCMw.log

Standard orientation:

Center Number	Atomic Number	Atomic Type	Coordinates (Angstroms) X	Y	Z
1	6	0	-2.442785	-1.846206	0.380351
2	6	0	-1.167736	-1.365401	0.450753
3	6	0	-0.840113	-0.009518	0.142924
4	6	0	-1.930158	0.829937	-0.253623
5	6	0	-3.236593	0.392876	-0.368116
6	6	0	-3.519391	-0.968828	-0.050336
7	6	0	0.447408	0.522384	0.222759
8	6	0	-0.455889	2.671957	-0.489146
9	6	0	0.662645	1.892056	-0.110169
10	6	0	1.933212	2.524465	-0.081922
11	1	0	2.816262	1.962903	0.196500
12	6	0	2.064702	3.854714	-0.408498
13	6	0	0.912297	4.609867	-0.780559
14	6	0	-0.342360	4.020801	-0.822877
15	1	0	-0.370646	-2.029642	0.765484
16	1	0	-1.224718	4.582810	-1.108254
17	8	0	-1.702357	2.138500	-0.545780
18	7	0	-4.782179	-1.447183	-0.147525
19	6	0	-5.137736	-2.831668	0.204449
20	1	0	-5.983358	-3.117725	-0.426194
21	1	0	-5.484073	-2.859588	1.247176
22	6	0	-5.914383	-0.552706	-0.434553
23	8	0	1.168462	5.902097	-1.075738
24	1	0	0.363320	6.380842	-1.322000
25	6	0	1.616340	-0.350771	0.558936
26	6	0	2.162611	-0.508650	1.853856
27	6	0	2.209285	-1.030622	-0.509555
28	6	0	3.281543	-1.335151	2.025719
29	6	0	3.314953	-1.867189	-0.327232
30	1	0	1.816694	-0.911672	-1.513129
31	6	0	3.857265	-2.008187	0.955320
32	1	0	3.693855	-1.432718	3.022941
33	6	0	1.644246	0.145104	3.095952
34	8	0	2.165888	0.021802	4.188993
35	8	0	0.538298	0.884956	2.902921
36	1	0	0.288653	1.265120	3.763511
37	6	0	3.878081	-2.538489	-1.555354
38	8	0	3.669946	-2.075619	-2.681207
39	7	0	4.618712	-3.653587	-1.349756
40	6	0	5.192698	-4.399235	-2.459914
41	1	0	5.956233	-5.075119	-2.073482
42	1	0	4.434504	-4.985066	-2.992343
43	1	0	5.649632	-3.705563	-3.167845
44	1	0	4.620252	-4.086184	-0.438449
45	6	0	-3.971096	-3.788064	0.003219
46	1	0	-4.261037	-4.782239	0.353937
47	1	0	-3.740465	-3.868140	-1.064972
48	6	0	-4.326917	1.339323	-0.819275
49	6	0	-5.513353	0.563094	-1.389688
50	6	0	-2.755454	-3.271958	0.770193
51	1	0	-6.368124	1.226507	-1.546579
52	1	0	-5.249931	0.129722	-2.360835
53	1	0	-4.656317	1.953438	0.029585
54	1	0	-3.929197	2.033990	-1.563700
55	1	0	-1.878499	-3.902533	0.599194
56	1	0	-2.967020	-3.311829	1.847675
57	1	0	-6.711301	-1.162724	-0.865307
58	1	0	-6.292677	-0.137260	0.509852
59	8	0	3.280760	4.454024	-0.379305
60	1	0	3.174208	5.383043	-0.639692
61	1	0	4.740107	-2.614683	1.127131

122aaa_Rh_Julolidin5COOH_OH-H+_carb_B3LYP631dp_PCMw.log

Standard orientation:

Center Atomic Atomic Coordinates (Angstroms)

Number	Number	Type	X	Y	Z
1	6	0	-2.471218	-1.814565	0.381931
2	6	0	-1.191905	-1.339967	0.494514
3	6	0	-0.845909	0.011350	0.215707
4	6	0	-1.909974	0.859365	-0.199048
5	6	0	-3.221082	0.431931	-0.353908
6	6	0	-3.525021	-0.926475	-0.058255
7	6	0	0.454624	0.534127	0.371326
8	6	0	-0.411939	2.682196	-0.424044
9	6	0	0.685331	1.894342	-0.016936
10	6	0	1.964882	2.507930	0.007842
11	1	0	2.826007	1.926826	0.318407
12	6	0	2.116968	3.825197	-0.356491
13	6	0	0.988644	4.595179	-0.760310
14	6	0	-0.272432	4.020009	-0.794397
15	1	0	-0.407916	-2.013503	0.823553
16	1	0	-1.133636	4.598000	-1.106613
17	8	0	-1.671442	2.171299	-0.484163
18	7	0	-4.797089	-1.394252	-0.198901
19	6	0	-5.170749	-2.769975	0.153767
20	1	0	-6.018985	-3.048805	-0.477706
21	1	0	-5.518586	-2.802656	1.196962
22	6	0	-5.909049	-0.478020	-0.477208
23	8	0	1.151647	5.885551	-1.115878
24	1	0	2.094584	6.107053	-1.032103
25	6	0	1.617457	-0.376805	0.608676
26	6	0	2.137750	-0.572581	1.897732
27	6	0	2.200945	-1.027338	-0.482737
28	6	0	3.237373	-1.416041	2.069988
29	6	0	3.295574	-1.882282	-0.304965
30	1	0	1.819459	-0.879008	-1.487319
31	6	0	3.817590	-2.066373	0.984449
32	1	0	3.627194	-1.540193	3.074629
33	6	0	1.490618	0.148636	3.075985
34	8	0	1.957819	-0.050955	4.219368
35	8	0	0.517448	0.896983	2.753544
36	6	0	3.868118	-2.532122	-1.535468
37	8	0	3.672967	-2.060264	-2.661633
38	7	0	4.611449	-3.651325	-1.342796
39	6	0	5.190469	-4.379824	-2.460481
40	1	0	5.944648	-5.069923	-2.080101
41	1	0	4.434841	-4.949580	-3.014367
42	1	0	5.661292	-3.677365	-3.150770
43	1	0	4.598193	-4.101328	-0.440278
44	6	0	-4.013240	-3.739061	-0.047878
45	1	0	-4.317669	-4.735363	0.285065
46	1	0	-3.773192	-3.805977	-1.115169
47	6	0	-4.288580	1.394251	-0.828666
48	6	0	-5.479723	0.639481	-1.419240
49	6	0	-2.798301	-3.247853	0.737508
50	1	0	-6.321485	1.317769	-1.584817
51	1	0	-5.209540	0.207314	-2.389318
52	1	0	-4.623642	2.018557	0.010835
53	1	0	-3.865752	2.080623	-1.567399
54	1	0	-1.925664	-3.882200	0.555556
55	1	0	-3.015920	-3.315750	1.812517
56	1	0	-6.716518	-1.066220	-0.920195
57	1	0	-6.289250	-0.060126	0.466615
58	8	0	3.292270	4.519007	-0.375245
59	1	0	4.030116	3.959649	-0.094769
60	1	0	4.691899	-2.689971	1.144367

122baa_Rh_Julolidin5COOH_OH-H+_carb_B3LYP631dp_PCMw_ring.log

Standard orientation:

Center	Atomic	Atomic	Coordinates (Angstroms)		
Number	Number	Type	X	Y	Z

1	6	0	-2.636719	-1.559084	0.563100
2	6	0	-1.337185	-1.154885	0.819495
3	6	0	-0.858934	0.126954	0.506489
4	6	0	-1.763968	1.003938	-0.098226
5	6	0	-3.085211	0.650864	-0.402308
6	6	0	-3.537131	-0.646748	-0.063224
7	6	0	0.529187	0.566155	0.885383
8	6	0	-0.101611	2.678850	-0.339514
9	6	0	0.883925	1.887584	0.252350
10	6	0	2.197868	2.395973	0.303288
11	1	0	2.984975	1.804358	0.761146
12	6	0	2.503359	3.638382	-0.220680
13	6	0	1.489083	4.423045	-0.815341
14	6	0	0.191932	3.939039	-0.875670
15	1	0	-0.664697	-1.863465	1.295544
16	1	0	-0.591547	4.527799	-1.338330
17	8	0	-1.410089	2.285959	-0.452340
18	7	0	-4.832420	-1.041413	-0.374977
19	6	0	-5.368472	-2.284885	0.173948
20	1	0	-6.232816	-2.568538	-0.435183
21	1	0	-5.735826	-2.136887	1.204128
22	6	0	-5.817705	-0.027481	-0.740132
23	8	0	1.786945	5.643029	-1.334818
24	1	0	2.737672	5.794006	-1.207713
25	6	0	1.589325	-0.509169	0.708460
26	6	0	2.124320	-0.857971	1.943018
27	6	0	2.037440	-1.122498	-0.454245
28	6	0	3.121441	-1.823568	2.067603
29	6	0	3.027476	-2.109774	-0.349993
30	1	0	1.647716	-0.861883	-1.431771
31	6	0	3.569046	-2.448622	0.906235
32	1	0	3.538302	-2.071099	3.038085
33	6	0	1.467209	-0.045040	2.990051
34	8	0	1.652483	-0.047860	4.190768
35	8	0	0.560918	0.767120	2.391421
36	6	0	3.494660	-2.746039	-1.635339
37	8	0	3.392546	-2.150394	-2.712606
38	7	0	4.029678	-3.988094	-1.537369
39	6	0	4.483838	-4.715449	-2.712938
40	1	0	5.139411	-5.527352	-2.395560
41	1	0	3.646523	-5.135868	-3.282273
42	1	0	5.036983	-4.038886	-3.366301
43	1	0	3.927688	-4.501172	-0.674626
44	6	0	-4.315423	-3.388255	0.153915
45	1	0	-4.741534	-4.309933	0.562048
46	1	0	-4.026527	-3.589684	-0.884346
47	6	0	-3.995072	1.657647	-1.077436
48	6	0	-5.212169	0.980598	-1.710856
49	6	0	-3.097635	-2.946231	0.966472
50	1	0	-5.963914	1.726227	-1.987407
51	1	0	-4.917898	0.456168	-2.627355
52	1	0	-4.328230	2.403775	-0.342402
53	1	0	-3.433680	2.216010	-1.832667
54	1	0	-2.274026	-3.659269	0.852174
55	1	0	-3.361045	-2.950933	2.033753
56	1	0	-6.667754	-0.540842	-1.200209
57	1	0	-6.199718	0.492644	0.155102
58	8	0	3.746687	4.217237	-0.222172
59	1	0	4.399973	3.622116	0.170117
60	1	0	4.365996	-3.181441	0.975035

123aaa_Rh_Julolidin5COOH_OH-H+oh_B3LYP631dp_PCMw.log

Standard orientation:

Center Number	Atomic Number	Atomic Type	Coordinates (Angstroms) X	Y	Z
1	6	0	-2.445208	-1.830910	0.328863
2	6	0	-1.163552	-1.338679	0.414240

3	6	0	-0.842691	0.009961	0.122652
4	6	0	-1.918881	0.837886	-0.276372
5	6	0	-3.230982	0.394200	-0.401932
6	6	0	-3.513269	-0.963526	-0.094111
7	6	0	0.463150	0.568625	0.214007
8	6	0	-0.455395	2.710651	-0.497900
9	6	0	0.674800	1.909586	-0.102697
10	6	0	1.957217	2.559567	-0.051700
11	1	0	2.833881	1.992597	0.238920
12	6	0	2.067917	3.878293	-0.367459
13	6	0	0.909649	4.698797	-0.771533
14	6	0	-0.357082	4.044727	-0.821053
15	1	0	-0.367928	-2.006835	0.727472
16	1	0	-1.239534	4.602106	-1.113937
17	8	0	-1.698313	2.154772	-0.565516
18	7	0	-4.790759	-1.450026	-0.215832
19	6	0	-5.138691	-2.809477	0.205068
20	1	0	-6.018709	-3.115000	-0.368783
21	1	0	-5.427871	-2.816170	1.267853
22	6	0	-5.913484	-0.538427	-0.444423
23	8	0	1.134235	5.909089	-1.040875
24	6	0	1.623002	-0.315035	0.559957
25	6	0	2.123632	-0.529797	1.865316
26	6	0	2.255840	-0.956290	-0.510662
27	6	0	3.237499	-1.364087	2.043660
28	6	0	3.351831	-1.803949	-0.324598
29	1	0	1.897599	-0.795758	-1.521279
30	6	0	3.849429	-1.997100	0.969842
31	1	0	3.616491	-1.499298	3.049682
32	6	0	1.570483	0.073729	3.118137
33	8	0	2.080207	-0.072693	4.215393
34	8	0	0.448200	0.791651	2.940134
35	1	0	0.185169	1.131056	3.813662
36	6	0	3.955639	-2.432985	-1.554653
37	8	0	3.791658	-1.931991	-2.671701
38	7	0	4.683975	-3.559353	-1.361815
39	6	0	5.292541	-4.269707	-2.476355
40	1	0	6.030102	-4.972068	-2.086648
41	1	0	4.549071	-4.822504	-3.062336
42	1	0	5.788087	-3.556531	-3.137586
43	1	0	4.649301	-4.023116	-0.466607
44	6	0	-3.985762	-3.778065	-0.029366
45	1	0	-4.269021	-4.771426	0.330713
46	1	0	-3.791481	-3.859257	-1.105083
47	6	0	-4.318110	1.345444	-0.855180
48	6	0	-5.523806	0.580701	-1.402859
49	6	0	-2.740334	-3.269830	0.696178
50	1	0	-6.374636	1.253714	-1.543339
51	1	0	-5.282882	0.146939	-2.380091
52	1	0	-4.631394	1.977840	-0.013016
53	1	0	-3.921513	2.027449	-1.612682
54	1	0	-1.870740	-3.895207	0.470819
55	1	0	-2.903911	-3.344623	1.780457
56	1	0	-6.737965	-1.126774	-0.856480
57	1	0	-6.261222	-0.117247	0.511949
58	8	0	3.232835	4.563518	-0.344568
59	1	0	2.948357	5.467124	-0.621257
60	1	0	4.725375	-2.611927	1.147762

124aaa_Rh_Julolidin5COOH_OH-2H_B3LYP631dp_PCMw.log

Standard orientation:

Center Number	Atomic Number	Atomic Type	Coordinates (Angstroms) X	Y	Z
1	6	0	-2.482596	-1.806081	0.272613
2	6	0	-1.198321	-1.330125	0.413353
3	6	0	-0.851375	0.019570	0.168607
4	6	0	-1.901736	0.868361	-0.245491

5	6	0	-3.216087	0.442815	-0.426643
6	6	0	-3.524647	-0.916056	-0.160918
7	6	0	0.462972	0.554663	0.328293
8	6	0	-0.407520	2.720329	-0.391355
9	6	0	0.695425	1.898600	0.018892
10	6	0	1.983779	2.531422	0.112067
11	1	0	2.840974	1.946725	0.424919
12	6	0	2.123525	3.851653	-0.187597
13	6	0	0.992077	4.693299	-0.612035
14	6	0	-0.280373	4.058838	-0.699260
15	1	0	-0.419761	-2.012638	0.738200
16	1	0	-1.147649	4.630739	-1.009920
17	8	0	-1.660100	2.188927	-0.500683
18	7	0	-4.806555	-1.384813	-0.340114
19	6	0	-5.184021	-2.740397	0.062813
20	1	0	-6.060508	-3.026197	-0.526827
21	1	0	-5.488884	-2.755967	1.121748
22	6	0	-5.907900	-0.447523	-0.559493
23	8	0	1.243838	5.905674	-0.864629
24	6	0	1.610110	-0.364331	0.616812
25	6	0	2.014256	-0.723206	1.918204
26	6	0	2.298367	-0.884407	-0.486640
27	6	0	3.102460	-1.592060	2.065147
28	6	0	3.375531	-1.763483	-0.330080
29	1	0	2.002773	-0.608912	-1.493510
30	6	0	3.780786	-2.110364	0.966975
31	1	0	3.402799	-1.838993	3.077619
32	6	0	1.313228	-0.179498	3.175110
33	8	0	1.750343	-0.591063	4.280340
34	8	0	0.366764	0.627302	2.971195
35	6	0	4.053569	-2.260551	-1.576155
36	8	0	3.960703	-1.653343	-2.649894
37	7	0	4.777647	-3.403969	-1.458324
38	6	0	5.453209	-3.998474	-2.600329
39	1	0	6.188563	-4.719954	-2.241326
40	1	0	4.753433	-4.510425	-3.271993
41	1	0	5.962201	-3.218446	-3.169340
42	1	0	4.683951	-3.959412	-0.621750
43	6	0	-4.042680	-3.724743	-0.165503
44	1	0	-4.349301	-4.720096	0.169611
45	1	0	-3.828348	-3.788844	-1.238684
46	6	0	-4.274611	1.419555	-0.894934
47	6	0	-5.481128	0.686769	-1.484029
48	6	0	-2.803705	-3.251151	0.594264
49	1	0	-6.317643	1.377100	-1.627734
50	1	0	-5.226910	0.270043	-2.465408
51	1	0	-4.597651	2.047535	-0.052992
52	1	0	-3.846072	2.104478	-1.632477
53	1	0	-1.938470	-3.882005	0.366335
54	1	0	-2.986442	-3.355005	1.673155
55	1	0	-6.737679	-1.008739	-0.998616
56	1	0	-6.263413	-0.041279	0.401185
57	8	0	3.299907	4.521848	-0.125779
58	1	0	3.028513	5.430367	-0.401469
59	1	0	4.640674	-2.753912	1.127805

124bba_Rh_Julolidin5COOH_OH-2H_B3LYP631dp_PCMw_ring.log

Standard orientation:

Center Number	Atomic Number	Atomic Type	Coordinates (Angstroms) X	Y	Z
1	6	0	-2.592558	-1.596558	0.534007
2	6	0	-1.300456	-1.165122	0.787724
3	6	0	-0.849558	0.128051	0.480915
4	6	0	-1.774894	0.993946	-0.112992
5	6	0	-3.091776	0.609904	-0.410928
6	6	0	-3.513822	-0.698257	-0.080024
7	6	0	0.527475	0.599713	0.849033

8	6	0	-0.136714	2.704806	-0.351403
9	6	0	0.857897	1.911920	0.225863
10	6	0	2.176665	2.453721	0.277507
11	1	0	2.980814	1.875578	0.723530
12	6	0	2.437746	3.699883	-0.230789
13	6	0	1.412100	4.532153	-0.837639
14	6	0	0.112916	3.976670	-0.880332
15	1	0	-0.610978	-1.862265	1.256830
16	1	0	-0.703286	4.533360	-1.330707
17	8	0	-1.450956	2.278085	-0.457345
18	7	0	-4.805051	-1.119880	-0.391944
19	6	0	-5.312694	-2.367547	0.172745
20	1	0	-6.185148	-2.666173	-0.417662
21	1	0	-5.661353	-2.222517	1.210442
22	6	0	-5.812888	-0.117207	-0.722513
23	8	0	1.787121	5.679107	-1.281062
24	6	0	1.607202	-0.459531	0.705698
25	6	0	2.132316	-0.804554	1.945429
26	6	0	2.085126	-1.062456	-0.451500
27	6	0	3.145460	-1.752153	2.079345
28	6	0	3.090358	-2.033146	-0.338883
29	1	0	1.704506	-0.802848	-1.432974
30	6	0	3.621232	-2.366539	0.923528
31	1	0	3.553503	-1.994219	3.055178
32	6	0	1.448641	-0.009243	2.992619
33	8	0	1.638594	-0.029655	4.197061
34	8	0	0.537442	0.783944	2.403238
35	6	0	3.584524	-2.658549	-1.618478
36	8	0	3.478352	-2.069488	-2.699315
37	7	0	4.149948	-3.887453	-1.514037
38	6	0	4.631804	-4.605121	-2.684149
39	1	0	5.305538	-5.399474	-2.360128
40	1	0	3.810505	-5.048187	-3.259831
41	1	0	5.172659	-3.915987	-3.334728
42	1	0	4.052485	-4.401530	-0.651470
43	6	0	-4.243706	-3.455236	0.135362
44	1	0	-4.649597	-4.384790	0.546696
45	1	0	-3.967124	-3.649104	-0.907773
46	6	0	-4.025999	1.603377	-1.072521
47	6	0	-5.241856	0.911403	-1.692643
48	6	0	-3.021062	-2.996261	0.931501
49	1	0	-6.011133	1.646145	-1.950238
50	1	0	-4.953975	0.400365	-2.618831
51	1	0	-4.360518	2.344177	-0.332410
52	1	0	-3.481550	2.172215	-1.832622
53	1	0	-2.186441	-3.693177	0.797158
54	1	0	-3.266927	-3.017183	2.002953
55	1	0	-6.665052	-0.637504	-1.171226
56	1	0	-6.184463	0.387542	0.186515
57	8	0	3.671891	4.289806	-0.226033
58	1	0	3.442044	5.148801	-0.669549
59	1	0	4.430783	-3.084675	1.001485

131aaa_Rh_Julolidin5COOH_OMe_B3LYP631dp_PCMw.log

Standard orientation:

Center Number	Atomic Number	Atomic Type	Coordinates (Angstroms) X	Y	Z
1	6	0	-2.855695	-1.489431	0.445963
2	6	0	-1.517366	-1.224576	0.493760
3	6	0	-0.974710	0.052293	0.156576
4	6	0	-1.916203	1.052744	-0.242474
5	6	0	-3.278834	0.836677	-0.333002
6	6	0	-3.778431	-0.453663	0.013801
7	6	0	0.385626	0.366670	0.212123
8	6	0	-0.162412	2.624408	-0.526254
9	6	0	0.816618	1.676692	-0.141397
10	6	0	2.178735	2.091151	-0.135766

11	1	0	2.942624	1.380144	0.148022
12	6	0	2.514265	3.376852	-0.489720
13	6	0	1.497074	4.309248	-0.871823
14	6	0	0.164472	3.928996	-0.889579
15	1	0	-0.837732	-2.007014	0.812121
16	1	0	-0.606653	4.631007	-1.181580
17	8	0	-1.479357	2.300969	-0.562651
18	7	0	-5.105238	-0.717257	-0.058590
19	6	0	-5.678171	-2.016829	0.326945
20	1	0	-6.572797	-2.168982	-0.282397
21	1	0	-6.002300	-1.971163	1.376301
22	6	0	-6.077183	0.347701	-0.350679
23	8	0	1.844266	5.558905	-1.217391
24	1	0	2.812499	5.633188	-1.145595
25	6	0	1.396170	-0.687525	0.541979
26	6	0	2.006107	-0.855356	1.806931
27	6	0	1.773763	-1.534850	-0.504816
28	6	0	2.975541	-1.854368	1.970341
29	6	0	2.724468	-2.544936	-0.327785
30	1	0	1.331378	-1.413383	-1.487060
31	6	0	3.335891	-2.692653	0.922486
32	1	0	3.442618	-1.957294	2.942641
33	6	0	1.699579	-0.044738	3.026558
34	8	0	2.335057	-0.120546	4.062015
35	8	0	0.641515	0.772898	2.885671
36	1	0	0.532260	1.254381	3.724525
37	6	0	3.056818	-3.395183	-1.528725
38	8	0	2.861618	-2.978067	-2.674569
39	7	0	3.578627	-4.618987	-1.275503
40	6	0	3.910637	-5.540812	-2.351748
41	1	0	4.529953	-6.342907	-1.949190
42	1	0	3.013005	-5.976595	-2.805337
43	1	0	4.465530	-5.012565	-3.129214
44	1	0	3.560965	-4.978704	-0.333172
45	6	0	-4.690359	-3.156691	0.122604
46	1	0	-5.134440	-4.084121	0.494222
47	1	0	-4.496610	-3.288257	-0.947808
48	6	0	-4.203708	1.942647	-0.790890
49	6	0	-5.509470	1.363232	-1.333026
50	6	0	-3.391705	-2.840032	0.861149
51	1	0	-6.244621	2.155993	-1.495883
52	1	0	-5.333900	0.873276	-2.297089
53	1	0	-4.415743	2.617924	0.048878
54	1	0	-3.706847	2.548510	-1.553052
55	1	0	-2.633773	-3.607994	0.682800
56	1	0	-3.585770	-2.834179	1.942662
57	1	0	-6.970358	-0.129262	-0.760061
58	1	0	-6.368585	0.837785	0.588909
59	8	0	3.760346	3.914539	-0.531210
60	6	0	4.874653	3.087789	-0.176681
61	1	0	4.784096	2.739081	0.856877
62	1	0	4.951674	2.230903	-0.853414
63	1	0	5.756564	3.718352	-0.277708
64	1	0	4.108689	-3.436747	1.083224

132aaa_Rh_Julolidin5COOH_OMe+H+carb_B3LYP631dp_PCMw.log

Standard orientation:

Center Number	Atomic Number	Atomic Type	Coordinates (Angstroms) X	Y	Z
1	6	0	-2.854732	-1.510149	0.405834
2	6	0	-1.518263	-1.229618	0.505384
3	6	0	-0.977863	0.053001	0.211678
4	6	0	-1.907461	1.044860	-0.206069
5	6	0	-3.268905	0.815713	-0.347283
6	6	0	-3.768969	-0.479186	-0.034260
7	6	0	0.386752	0.379139	0.357128
8	6	0	-0.157557	2.624010	-0.450752

9	6	0	0.813250	1.688761	-0.036698
10	6	0	2.171026	2.108092	-0.009539
11	1	0	2.926103	1.403489	0.312089
12	6	0	2.513736	3.387964	-0.381834
13	6	0	1.505300	4.309924	-0.798049
14	6	0	0.175368	3.924762	-0.832310
15	1	0	-0.840701	-2.009807	0.834943
16	1	0	-0.592311	4.619098	-1.151334
17	8	0	-1.478050	2.303366	-0.507993
18	7	0	-5.098095	-0.753544	-0.159222
19	6	0	-5.668003	-2.054420	0.213719
20	1	0	-6.557332	-2.209234	-0.403691
21	1	0	-6.001861	-2.026030	1.261674
22	6	0	-6.063494	0.314766	-0.443437
23	8	0	1.857304	5.558336	-1.162045
24	6	0	1.400062	-0.695829	0.595624
25	6	0	1.905552	-0.952182	1.880134
26	6	0	1.851265	-1.450129	-0.491945
27	6	0	2.859908	-1.957485	2.050718
28	6	0	2.794847	-2.469235	-0.314413
29	1	0	1.478309	-1.260097	-1.492685
30	6	0	3.305749	-2.712722	0.969526
31	1	0	3.242781	-2.126163	3.051587
32	6	0	1.396918	-0.126166	3.058044
33	8	0	1.857290	-0.374823	4.194798
34	8	0	0.534547	0.749595	2.742765
35	6	0	3.223811	-3.232481	-1.538130
36	8	0	3.107083	-2.751772	-2.671314
37	7	0	3.746321	-4.467747	-1.329471
38	6	0	4.160065	-5.315042	-2.436985
39	1	0	4.758852	-6.138751	-2.046503
40	1	0	3.301465	-5.726419	-2.980919
41	1	0	4.761836	-4.734720	-3.138973
42	1	0	3.658102	-4.889912	-0.417748
43	6	0	-4.670723	-3.187016	0.007800
44	1	0	-5.115907	-4.124246	0.353446
45	1	0	-4.456878	-3.296922	-1.061473
46	6	0	-4.185349	1.921149	-0.825691
47	6	0	-5.478701	1.345188	-1.401244
48	6	0	-3.386777	-2.877279	0.775394
49	1	0	-6.211721	2.138790	-1.571418
50	1	0	-5.281663	0.866376	-2.367009
51	1	0	-4.418399	2.596295	0.008948
52	1	0	-3.671110	2.530088	-1.574209
53	1	0	-2.619915	-3.634788	0.588043
54	1	0	-3.598801	-2.906317	1.853261
55	1	0	-6.952225	-0.152242	-0.875093
56	1	0	-6.371566	0.795272	0.496959
57	8	0	3.764708	3.925818	-0.405980
58	6	0	4.865059	3.105699	-0.002874
59	1	0	4.748142	2.781789	1.036434
60	1	0	4.957555	2.230809	-0.654650
61	1	0	5.752779	3.729747	-0.096384
62	1	0	2.823282	5.632625	-1.070489
63	1	0	4.069312	-3.468374	1.127383

132baa_Rh_Julolidin5COOH_OMe+H+carb_B3LYP631dp_PCMw_ring.log

Standard orientation:

Center Number	Atomic Number	Atomic Type	Coordinates (Angstroms) X	Y	Z
1	6	0	3.011534	1.218318	0.616561
2	6	0	1.664175	1.009422	0.859583
3	6	0	0.996634	-0.172976	0.504595
4	6	0	1.759200	-1.158021	-0.129167
5	6	0	3.120533	-1.001672	-0.420706
6	6	0	3.764648	0.199306	-0.039512
7	6	0	-0.445296	-0.403024	0.867270

8	6	0	-0.133882	-2.561880	-0.398983
9	6	0	-0.988334	-1.652604	0.222306
10	6	0	-2.362872	-1.968306	0.289877
11	1	0	-3.038623	-1.272766	0.772208
12	6	0	-2.849260	-3.146378	-0.248216
13	6	0	-1.960589	-4.054677	-0.876430
14	6	0	-0.610832	-3.758273	-0.952530
15	1	0	1.105736	1.796347	1.359695
16	1	0	0.074930	-4.443273	-1.437357
17	8	0	1.215396	-2.358471	-0.526162
18	7	0	5.106979	0.400071	-0.337397
19	6	0	5.824756	1.523115	0.260994
20	1	0	6.729895	1.689863	-0.331786
21	1	0	6.151991	1.282383	1.287210
22	6	0	5.926691	-0.738032	-0.742967
23	8	0	-2.438045	-5.209235	-1.408577
24	6	0	-1.332409	0.818588	0.685298
25	6	0	-1.824140	1.237652	1.915999
26	6	0	-1.675335	1.493459	-0.479147
27	6	0	-2.672546	2.336902	2.034847
28	6	0	-2.513130	2.613114	-0.380485
29	1	0	-1.317708	1.182183	-1.454158
30	6	0	-3.012911	3.023441	0.871780
31	1	0	-3.059759	2.639304	3.002068
32	6	0	-1.304472	0.333230	2.965320
33	8	0	-1.499347	0.359076	4.164242
34	8	0	-0.521818	-0.601681	2.371203
35	6	0	-2.869479	3.314502	-1.667430
36	8	0	-2.841739	2.714964	-2.747131
37	7	0	-3.220606	4.620378	-1.569421
38	6	0	-3.551591	5.407457	-2.747719
39	1	0	-4.051569	6.323621	-2.431401
40	1	0	-2.658753	5.670775	-3.326805
41	1	0	-4.221233	4.835489	-3.392315
42	1	0	-3.057705	5.110965	-0.702980
43	6	0	4.957945	2.777996	0.275374
44	1	0	5.518298	3.606918	0.718606
45	1	0	4.715324	3.057979	-0.756456
46	6	0	3.868882	-2.113208	-1.128932
47	6	0	5.175237	-1.608375	-1.744479
48	6	0	3.677387	2.504885	1.065805
49	1	0	5.804946	-2.449637	-2.050115
50	1	0	4.964551	-1.014011	-2.641186
51	1	0	4.084163	-2.923592	-0.418434
52	1	0	3.230746	-2.556099	-1.899442
53	1	0	2.975054	3.339998	0.969310
54	1	0	3.926316	2.436474	2.134322
55	1	0	6.844332	-0.343601	-1.190419
56	1	0	6.226992	-1.340964	0.131390
57	8	0	-4.151709	-3.568003	-0.250330
58	6	0	-5.134888	-2.727967	0.353129
59	1	0	-4.924690	-2.578397	1.417929
60	1	0	-5.184175	-1.755892	-0.149917
61	1	0	-6.086083	-3.247019	0.238363
62	1	0	-3.397491	-5.223154	-1.254556
63	1	0	-3.695926	3.863807	0.935790

133aaa_Rh_Julolidin5COOH_OMe+H+oh_B3LYP631dp_PCMw.log

Standard orientation:

Center Number	Atomic Number	Atomic Type	Coordinates (Angstroms) X	Y	Z
1	6	0	-2.827141	-1.589388	0.249781
2	6	0	-1.492111	-1.270690	0.362219
3	6	0	-0.993134	0.029204	0.110037
4	6	0	-1.942997	0.998159	-0.288579
5	6	0	-3.299804	0.731959	-0.442581
6	6	0	-3.763941	-0.581795	-0.165241

7	6	0	0.377985	0.409140	0.231027
8	6	0	-0.241083	2.665148	-0.452257
9	6	0	0.763543	1.713683	-0.052986
10	6	0	2.116413	2.196907	0.040614
11	1	0	2.880442	1.495190	0.348403
12	6	0	2.430830	3.493994	-0.244033
13	6	0	1.398741	4.478788	-0.666695
14	6	0	0.052796	3.969553	-0.745277
15	1	0	-0.797171	-2.047221	0.665353
16	1	0	-0.735520	4.650664	-1.046065
17	8	0	-1.548223	2.281021	-0.552289
18	7	0	-5.094242	-0.892647	-0.314886
19	6	0	-5.623188	-2.197727	0.086635
20	1	0	-6.534382	-2.374918	-0.492770
21	1	0	-5.914728	-2.181990	1.148986
22	6	0	-6.081505	0.164666	-0.537607
23	8	0	1.703040	5.659312	-0.928117
24	6	0	1.401862	-0.631521	0.567916
25	6	0	1.736422	-1.054703	1.875655
26	6	0	2.047880	-1.240157	-0.514228
27	6	0	2.696229	-2.063802	2.046217
28	6	0	2.993030	-2.255177	-0.339122
29	1	0	1.813236	-0.925359	-1.524833
30	6	0	3.321797	-2.661426	0.959799
31	1	0	2.946332	-2.363932	3.056915
32	6	0	1.153321	-0.506958	3.140339
33	8	0	1.374172	-0.980589	4.241323
34	8	0	0.366663	0.569300	2.968435
35	1	0	0.045887	0.831005	3.849387
36	6	0	3.626771	-2.831303	-1.579717
37	8	0	3.674377	-2.183058	-2.630022
38	7	0	4.137935	-4.081838	-1.472279
39	6	0	4.742578	-4.753715	-2.612843
40	1	0	5.296433	-5.623019	-2.257080
41	1	0	3.989167	-5.082959	-3.337804
42	1	0	5.428615	-4.071582	-3.118271
43	1	0	3.925314	-4.633781	-0.654899
44	6	0	-4.609157	-3.308550	-0.160725
45	1	0	-5.025428	-4.261342	0.179414
46	1	0	-4.421053	-3.395544	-1.237113
47	6	0	-4.243760	1.825361	-0.896621
48	6	0	-5.531837	1.236010	-1.472356
49	6	0	-3.311343	-2.986186	0.579105
50	1	0	-6.283220	2.018403	-1.614122
51	1	0	-5.335430	0.787356	-2.452850
52	1	0	-4.483101	2.483866	-0.050162
53	1	0	-3.747534	2.457768	-1.638320
54	1	0	-2.530512	-3.714858	0.338978
55	1	0	-3.488221	-3.066833	1.660886
56	1	0	-6.972343	-0.300904	-0.968519
57	1	0	-6.382685	0.614541	0.421869
58	8	0	3.674452	4.033187	-0.178670
59	6	0	4.747358	3.182952	0.217874
60	1	0	4.589249	2.787684	1.228306
61	1	0	4.871043	2.346256	-0.479809
62	1	0	5.644512	3.802265	0.205840
63	1	0	4.080067	-3.417304	1.135033

134aaa_Rh_Julolidin5COOH_OMe-2H+_B3LYP631dp_PCMw.log

Standard orientation:

Center Number	Atomic Number	Atomic Type	Coordinates (Angstroms) X	Y	Z
1	6	0	-2.838035	-1.556320	0.319001
2	6	0	-1.502297	-1.240534	0.443635
3	6	0	-0.992523	0.049840	0.173825
4	6	0	-1.931037	1.015809	-0.249497
5	6	0	-3.289310	0.752751	-0.416592

6	6	0	-3.763119	-0.552519	-0.124974
7	6	0	0.384108	0.417468	0.313146
8	6	0	-0.216700	2.663380	-0.436310
9	6	0	0.775019	1.713378	-0.018820
10	6	0	2.132018	2.186630	0.055368
11	1	0	2.887782	1.483029	0.379242
12	6	0	2.459756	3.473182	-0.262452
13	6	0	1.439610	4.457976	-0.703639
14	6	0	0.090422	3.959466	-0.763894
15	1	0	-0.812378	-2.010152	0.774513
16	1	0	-0.692860	4.638886	-1.082129
17	8	0	-1.530035	2.293484	-0.530202
18	7	0	-5.095747	-0.861922	-0.293201
19	6	0	-5.635134	-2.147587	0.150622
20	1	0	-6.550137	-2.334950	-0.419893
21	1	0	-5.922814	-2.099702	1.213659
22	6	0	-6.072728	0.200050	-0.531237
23	8	0	1.755471	5.630933	-0.997640
24	6	0	1.406018	-0.638544	0.604482
25	6	0	1.788916	-1.026500	1.904654
26	6	0	1.991467	-1.267562	-0.502140
27	6	0	2.752762	-2.033255	2.044042
28	6	0	2.939507	-2.285197	-0.351675
29	1	0	1.711143	-0.971969	-1.507786
30	6	0	3.325392	-2.661221	0.942983
31	1	0	3.042593	-2.300627	3.054196
32	6	0	1.198432	-0.377675	3.170230
33	8	0	1.619141	-0.819799	4.270585
34	8	0	0.349852	0.533639	2.979482
35	6	0	3.506166	-2.897335	-1.601852
36	8	0	3.477247	-2.300730	-2.685045
37	7	0	4.055184	-4.133980	-1.476925
38	6	0	4.598486	-4.845378	-2.622758
39	1	0	5.248265	-5.646995	-2.268457
40	1	0	3.809980	-5.279361	-3.249592
41	1	0	5.179806	-4.154959	-3.236119
42	1	0	3.897795	-4.655748	-0.628096
43	6	0	-4.630671	-3.273702	-0.066667
44	1	0	-5.056129	-4.214879	0.294333
45	1	0	-4.440777	-3.387430	-1.140348
46	6	0	-4.221493	1.844240	-0.899749
47	6	0	-5.509284	1.254997	-1.476662
48	6	0	-3.331912	-2.945390	0.669253
49	1	0	-6.254428	2.040195	-1.635875
50	1	0	-5.308466	0.790760	-2.449093
51	1	0	-4.463922	2.520665	-0.068267
52	1	0	-3.714005	2.459738	-1.648257
53	1	0	-2.555047	-3.682655	0.441986
54	1	0	-3.510660	-3.008998	1.751953
55	1	0	-6.966080	-0.262584	-0.960901
56	1	0	-6.375437	0.666112	0.420636
57	8	0	3.714573	3.998838	-0.214964
58	6	0	4.774240	3.143057	0.198946
59	1	0	4.614478	2.773351	1.219115
60	1	0	4.884530	2.286797	-0.477516
61	1	0	5.681715	3.747161	0.170066
62	1	0	4.090543	-3.416319	1.098170

134bba_Rh_Julolidin5COOH_OMe+2H+carb_B3LYP631dp_PCMw_ring.log

Standard orientation:

Center Number	Atomic Number	Atomic Type	Coordinates (Angstroms) X	Y	Z
1	6	0	2.936359	1.324793	0.548012
2	6	0	1.596366	1.062021	0.785570
3	6	0	0.985240	-0.159912	0.465483
4	6	0	1.798044	-1.136320	-0.123061
5	6	0	3.156547	-0.923686	-0.403494

6	6	0	3.740554	0.317941	-0.061967
7	6	0	-0.444900	-0.452452	0.814112
8	6	0	-0.043201	-2.621369	-0.387538
9	6	0	-0.931668	-1.701398	0.176728
10	6	0	-2.305407	-2.076862	0.202239
11	1	0	-3.012340	-1.383258	0.643593
12	6	0	-2.745169	-3.277461	-0.308821
13	6	0	-1.829101	-4.255499	-0.905397
14	6	0	-0.460544	-3.839499	-0.913733
15	1	0	0.997252	1.839765	1.252195
16	1	0	0.276030	-4.504430	-1.355426
17	8	0	1.316869	-2.368356	-0.476096
18	7	0	5.078772	0.570909	-0.358779
19	6	0	5.737133	1.735289	0.227015
20	1	0	6.651230	1.921690	-0.346241
21	1	0	6.045843	1.536595	1.268494
22	6	0	5.952606	-0.549887	-0.690852
23	8	0	-2.237842	-5.360312	-1.374801
24	6	0	-1.377912	0.738634	0.682988
25	6	0	-1.858151	1.139402	1.924387
26	6	0	-1.771974	1.406826	-0.470279
27	6	0	-2.741438	2.208495	2.063034
28	6	0	-2.642010	2.499513	-0.352094
29	1	0	-1.425883	1.107133	-1.453101
30	6	0	-3.129358	2.889415	0.911526
31	1	0	-3.118564	2.493124	3.039840
32	6	0	-1.287716	0.255008	2.969263
33	8	0	-1.482157	0.296850	4.173426
34	8	0	-0.486443	-0.645743	2.380922
35	6	0	-3.043554	3.196788	-1.626585
36	8	0	-3.026958	2.606011	-2.711647
37	7	0	-3.422270	4.494659	-1.513168
38	6	0	-3.795072	5.281094	-2.678961
39	1	0	-4.304436	6.186353	-2.346415
40	1	0	-2.921435	5.564141	-3.277917
41	1	0	-4.469039	4.700216	-3.311053
42	1	0	-3.247377	4.983606	-0.648170
43	6	0	4.821995	2.954862	0.183724
44	1	0	5.340073	3.819965	0.609332
45	1	0	4.590114	3.190624	-0.861648
46	6	0	3.963207	-2.027043	-1.058690
47	6	0	5.260823	-1.493720	-1.668811
48	6	0	3.537242	2.656336	0.958005
49	1	0	5.932109	-2.319181	-1.925852
50	1	0	5.045708	-0.946584	-2.594218
51	1	0	4.196377	-2.803890	-0.316669
52	1	0	3.356917	-2.522736	-1.822990
53	1	0	2.802496	3.456223	0.813630
54	1	0	3.765907	2.642946	2.033387
55	1	0	6.865707	-0.139807	-1.134057
56	1	0	6.254263	-1.101853	0.216760
57	8	0	-4.064297	-3.682921	-0.314708
58	6	0	-5.022191	-2.802520	0.245025
59	1	0	-4.827032	-2.607232	1.308462
60	1	0	-5.053150	-1.840257	-0.284377
61	1	0	-5.991378	-3.295449	0.145383
62	1	0	-3.838782	3.706343	0.992958

141aaa_Rh_Julolidin5COOH_F2_B3LYP631dp_PCMw.log

Standard orientation:

Center Number	Atomic Number	Atomic Type	Coordinates (Angstroms) X	Y	Z
1	6	0	-2.305230	-2.141543	0.356610
2	6	0	-1.057778	-1.598539	0.438535
3	6	0	-0.800500	-0.216549	0.175117
4	6	0	-1.937905	0.579617	-0.187929
5	6	0	-3.218580	0.084430	-0.306682

6	6	0	-3.429694	-1.304141	-0.036300
7	6	0	0.457650	0.371047	0.268269
8	6	0	-0.559361	2.489047	-0.372954
9	6	0	0.603521	1.763369	-0.026075
10	6	0	1.839793	2.454549	0.011315
11	1	0	2.758499	1.941979	0.265639
12	6	0	1.867062	3.791333	-0.283529
13	6	0	0.712774	4.530661	-0.628218
14	6	0	-0.500781	3.850556	-0.668668
15	1	0	-0.225320	-2.230777	0.725553
16	8	0	-1.779831	1.910140	-0.437750
17	7	0	-4.663594	-1.839736	-0.141715
18	6	0	-4.946289	-3.255225	0.157255
19	1	0	-5.780953	-3.557311	-0.479758
20	1	0	-5.283356	-3.336731	1.199697
21	6	0	-5.844064	-0.999393	-0.408568
22	8	0	0.752927	5.840341	-0.915362
23	1	0	1.669543	6.152813	-0.840724
24	6	0	1.668207	-0.451935	0.580310
25	6	0	2.235116	-0.593601	1.868638
26	6	0	2.268146	-1.110397	-0.497593
27	6	0	3.375766	-1.391873	2.026646
28	6	0	3.417647	-1.890172	-0.333916
29	1	0	1.842926	-1.025059	-1.491212
30	6	0	3.965833	-2.034815	0.945403
31	1	0	3.784973	-1.503351	3.023674
32	6	0	1.704088	0.032644	3.119762
33	8	0	2.226661	-0.097271	4.211185
34	8	0	0.586926	0.757522	2.934150
35	1	0	0.327467	1.121686	3.798762
36	6	0	3.972364	-2.565984	-1.563296
37	8	0	3.250994	-2.797372	-2.538466
38	7	0	5.283270	-2.904069	-1.527064
39	6	0	5.933616	-3.540243	-2.663277
40	1	0	6.097792	-2.834365	-3.485506
41	1	0	6.895634	-3.938892	-2.340032
42	1	0	5.310474	-4.357099	-3.031870
43	1	0	5.867629	-2.544006	-0.787617
44	6	0	-3.734366	-4.142234	-0.086603
45	1	0	-3.971374	-5.161997	0.228016
46	1	0	-3.508021	-4.170532	-1.158280
47	6	0	-4.360928	0.988959	-0.711135
48	6	0	-5.501954	0.173163	-1.316900
49	6	0	-2.541322	-3.594708	0.693399
50	1	0	-6.391767	0.794335	-1.448955
51	1	0	-5.214378	-0.205104	-2.303997
52	1	0	-4.721578	1.539308	0.168009
53	1	0	-4.005115	1.740937	-1.419898
54	1	0	-1.634237	-4.171438	0.492997
55	1	0	-2.742115	-3.685730	1.769752
56	1	0	-6.599966	-1.638455	-0.869333
57	1	0	-6.250923	-0.645662	0.548429
58	9	0	-1.620627	4.509849	-0.995649
59	9	0	3.021473	4.491761	-0.262660
60	1	0	4.830399	-2.667437	1.115132

142aaa_Rh_Julolidin5COOH_F2-H+carb_B3LYP631dp_PCMw.log

Standard orientation:

Center Number	Atomic Number	Atomic Type	Coordinates (Angstroms) X	Y	Z
1	6	0	-2.505855	-1.873474	0.539707
2	6	0	-1.228656	-1.410033	0.807820
3	6	0	-0.826426	-0.089195	0.555793
4	6	0	-1.787310	0.760524	0.002211
5	6	0	-3.088685	0.353226	-0.309551
6	6	0	-3.463285	-0.984492	-0.033947
7	6	0	0.537678	0.410577	0.949755

8	6	0	-0.228092	2.525852	-0.178708
9	6	0	0.818126	1.774630	0.364782
10	6	0	2.100224	2.348505	0.417611
11	1	0	2.940648	1.804878	0.832672
12	6	0	2.300381	3.622155	-0.064880
13	6	0	1.267881	4.395922	-0.615325
14	6	0	0.004808	3.818654	-0.666515
15	1	0	-0.513234	-2.102109	1.243615
16	8	0	-1.509200	2.081360	-0.291609
17	7	0	-4.735007	-1.435062	-0.356821
18	6	0	-5.194423	-2.738355	0.118714
19	1	0	-6.039584	-3.039052	-0.508761
20	1	0	-5.570664	-2.670678	1.153814
21	6	0	-5.778961	-0.466422	-0.680538
22	8	0	1.461924	5.646022	-1.096279
23	6	0	1.657588	-0.592310	0.723745
24	6	0	2.222257	-0.951336	1.942011
25	6	0	2.132261	-1.133970	-0.463462
26	6	0	3.276910	-1.858313	2.025700
27	6	0	3.181843	-2.061373	-0.400610
28	1	0	1.719720	-0.863068	-1.428691
29	6	0	3.752552	-2.411776	0.839351
30	1	0	3.715672	-2.115059	2.983990
31	6	0	1.525706	-0.217631	3.020808
32	8	0	1.715728	-0.249740	4.219178
33	8	0	0.566778	0.561189	2.453813
34	6	0	3.678865	-2.618962	-1.711382
35	8	0	3.536294	-1.988618	-2.763978
36	7	0	4.286125	-3.830092	-1.663249
37	6	0	4.777607	-4.482574	-2.867503
38	1	0	5.469006	-5.276372	-2.582257
39	1	0	3.962551	-4.915617	-3.459108
40	1	0	5.301353	-3.753410	-3.487839
41	1	0	4.218595	-4.381360	-0.820980
42	6	0	-4.076882	-3.773633	0.040500
43	1	0	-4.445824	-4.739055	0.400123
44	1	0	-3.779780	-3.903212	-1.006798
45	6	0	-4.060343	1.339079	-0.926705
46	6	0	-5.233765	0.622740	-1.598113
47	6	0	-2.884553	-3.302816	0.874614
48	1	0	-6.028591	1.334827	-1.840128
49	1	0	-4.907175	0.163419	-2.538236
50	1	0	-4.438164	2.018416	-0.149832
51	1	0	-3.536698	1.974351	-1.647379
52	1	0	-2.021359	-3.960362	0.725712
53	1	0	-3.144528	-3.374178	1.940250
54	1	0	-6.593868	-1.008340	-1.170218
55	1	0	-6.195609	-0.015024	0.235982
56	9	0	-1.016469	4.517461	-1.196251
57	9	0	3.527832	4.201154	-0.029921
58	1	0	2.397829	5.873719	-0.980575
59	1	0	4.592716	-3.097055	0.876403

142baa_Rh_Julolidin5COOH_F2-H+carb_B3LYP631dp_PCMw_ring.log

Standard orientation:

Center Number	Atomic Number	Atomic Type	Coordinates (Angstroms) X	Y	Z
1	6	0	-2.505580	-1.873971	0.539247
2	6	0	-1.228437	-1.410365	0.807393
3	6	0	-0.826454	-0.089415	0.555587
4	6	0	-1.787493	0.760241	0.002171
5	6	0	-3.088817	0.352787	-0.309588
6	6	0	-3.463189	-0.985030	-0.034158
7	6	0	0.537566	0.410561	0.949576
8	6	0	-0.228533	2.525795	-0.178679
9	6	0	0.817836	1.774644	0.364632
10	6	0	2.099879	2.348656	0.417332

11	1	0	2.940424	1.805097	0.832240
12	6	0	2.299828	3.622370	-0.065083
13	6	0	1.267173	4.396072	-0.615327
14	6	0	0.004157	3.818665	-0.666411
15	1	0	-0.512873	-2.102393	1.243033
16	8	0	-1.509595	2.081155	-0.291489
17	7	0	-4.734891	-1.435745	-0.357014
18	6	0	-5.194124	-2.738996	0.118841
19	1	0	-6.039556	-3.039730	-0.508247
20	1	0	-5.569898	-2.671229	1.154105
21	6	0	-5.779025	-0.467101	-0.680186
22	8	0	1.461015	5.646239	-1.096195
23	6	0	1.657650	-0.592141	0.723746
24	6	0	2.222360	-0.950922	1.942077
25	6	0	2.132488	-1.133817	-0.463381
26	6	0	3.277215	-1.857637	2.025881
27	6	0	3.182299	-2.060969	-0.400413
28	1	0	1.719924	-0.863112	-1.428657
29	6	0	3.753044	-2.411108	0.839594
30	1	0	3.716011	-2.114201	2.984206
31	6	0	1.525614	-0.217259	3.020786
32	8	0	1.715600	-0.249281	4.219169
33	8	0	0.566520	0.561232	2.453720
34	6	0	3.679475	-2.618533	-1.711148
35	8	0	3.536469	-1.988439	-2.763835
36	7	0	4.287297	-3.829366	-1.662935
37	6	0	4.779144	-4.481634	-2.867157
38	1	0	5.470262	-5.275634	-2.581807
39	1	0	3.964229	-4.914376	-3.459172
40	1	0	5.303290	-3.752459	-3.487158
41	1	0	4.220552	-4.380422	-0.820470
42	6	0	-4.076593	-3.774253	0.040200
43	1	0	-4.445371	-4.739706	0.399913
44	1	0	-3.779875	-3.903772	-1.007213
45	6	0	-4.060639	1.338606	-0.926547
46	6	0	-5.234188	0.622255	-1.597735
47	6	0	-2.883972	-3.303459	0.873915
48	1	0	-6.029142	1.334314	-1.839416
49	1	0	-4.907823	0.163109	-2.538021
50	1	0	-4.438300	2.017939	-0.149595
51	1	0	-3.537147	1.973889	-1.647329
52	1	0	-2.020754	-3.960851	0.724449
53	1	0	-3.143444	-3.375139	1.939651
54	1	0	-6.594056	-1.008982	-1.169699
55	1	0	-6.195379	-0.015901	0.236567
56	9	0	-1.017264	4.517391	-1.195971
57	9	0	3.527221	4.201500	-0.030233
58	1	0	2.396917	5.874019	-0.980622
59	1	0	4.593365	-3.096190	0.876777

143aaa_Rh_Julolidin5COOH_F2-H+oh_B3LYP631dp_PCMw.log

Standard orientation:

Center Number	Atomic Number	Atomic Type	Coordinates (Angstroms) X	Y	Z
1	6	0	-2.246881	-2.157502	0.231235
2	6	0	-1.006353	-1.577155	0.345590
3	6	0	-0.787132	-0.192160	0.139707
4	6	0	-1.925111	0.577835	-0.211068
5	6	0	-3.199332	0.048035	-0.358141
6	6	0	-3.379598	-1.345702	-0.132655
7	6	0	0.476035	0.447649	0.266334
8	6	0	-0.599340	2.546113	-0.331900
9	6	0	0.587519	1.814992	0.018819
10	6	0	1.811681	2.557214	0.106145
11	1	0	2.734003	2.053638	0.369437
12	6	0	1.821000	3.889982	-0.143561
13	6	0	0.643401	4.683256	-0.508869

14	6	0	-0.562164	3.894171	-0.581075
15	1	0	-0.161473	-2.203583	0.612268
16	8	0	-1.807468	1.924131	-0.426347
17	7	0	-4.616029	-1.913616	-0.275598
18	6	0	-4.859953	-3.323481	0.045984
19	1	0	-5.716843	-3.651042	-0.549719
20	1	0	-5.144312	-3.423730	1.104752
21	6	0	-5.806254	-1.080951	-0.472007
22	8	0	0.689442	5.908652	-0.731934
23	6	0	1.694009	-0.366863	0.578685
24	6	0	2.135946	-0.696153	1.881574
25	6	0	2.426068	-0.840072	-0.515632
26	6	0	3.287471	-1.481707	2.035585
27	6	0	3.565084	-1.635100	-0.355384
28	1	0	2.110390	-0.591940	-1.522698
29	6	0	3.998790	-1.949024	0.938049
30	1	0	3.616403	-1.711213	3.042118
31	6	0	1.478848	-0.266860	3.156132
32	8	0	1.854518	-0.625648	4.258196
33	8	0	0.433018	0.560645	2.988969
34	1	0	0.087469	0.767817	3.875057
35	6	0	4.280499	-2.079652	-1.606253
36	8	0	4.163420	-1.451680	-2.663324
37	7	0	5.052616	-3.187726	-1.496879
38	6	0	5.771509	-3.730332	-2.639745
39	1	0	6.523251	-4.434479	-2.281489
40	1	0	5.100843	-4.248597	-3.334966
41	1	0	6.264369	-2.919638	-3.179167
42	1	0	4.982086	-3.757818	-0.667672
43	6	0	-3.639342	-4.184870	-0.253860
44	1	0	-3.846316	-5.218146	0.039189
45	1	0	-3.445630	-4.179641	-1.332679
46	6	0	-4.358092	0.940700	-0.747225
47	6	0	-5.497426	0.122128	-1.354897
48	6	0	-2.431591	-3.634930	0.503714
49	1	0	-6.397228	0.734790	-1.461501
50	1	0	-5.217971	-0.228702	-2.354835
51	1	0	-4.721197	1.481165	0.137701
52	1	0	-4.016781	1.705990	-1.449797
53	1	0	-1.518847	-4.176736	0.237058
54	1	0	-2.583620	-3.792391	1.580713
55	1	0	-6.574029	-1.708207	-0.932754
56	1	0	-6.198078	-0.750238	0.502033
57	9	0	-1.706698	4.537102	-0.910438
58	9	0	2.976378	4.583430	-0.060957
59	1	0	4.901095	-2.528862	1.100683

144aaa_Rh_Julolidin5COOH_F2-2H+_B3LYP631dp_PCMw.log

Standard orientation:

Center Number	Atomic Number	Atomic Type	Coordinates (Angstroms) X	Y	Z
1	6	0	-2.246450	-2.143543	0.293183
2	6	0	-1.007482	-1.558799	0.421573
3	6	0	-0.785053	-0.178311	0.204433
4	6	0	-1.915990	0.585845	-0.167321
5	6	0	-3.190054	0.051992	-0.328332
6	6	0	-3.372520	-1.337665	-0.094022
7	6	0	0.482742	0.459652	0.354723
8	6	0	-0.587747	2.547700	-0.310392
9	6	0	0.592633	1.821763	0.054638
10	6	0	1.816276	2.562655	0.121599
11	1	0	2.735177	2.060559	0.399628
12	6	0	1.831395	3.890269	-0.161274
13	6	0	0.659884	4.679554	-0.542673
14	6	0	-0.544927	3.891520	-0.594009
15	1	0	-0.164817	-2.177733	0.711754
16	8	0	-1.800699	1.931067	-0.395660

17	7	0	-4.609525	-1.910828	-0.256580
18	6	0	-4.856850	-3.310200	0.099035
19	1	0	-5.712965	-3.651808	-0.490480
20	1	0	-5.143179	-3.388768	1.159797
21	6	0	-5.795734	-1.076341	-0.456950
22	8	0	0.709151	5.901833	-0.798050
23	6	0	1.707015	-0.362941	0.612281
24	6	0	2.180633	-0.651402	1.906996
25	6	0	2.391865	-0.863065	-0.502019
26	6	0	3.334561	-1.431821	2.040414
27	6	0	3.534202	-1.658414	-0.358194
28	1	0	2.041944	-0.641930	-1.504906
29	6	0	4.008993	-1.934193	0.932340
30	1	0	3.687181	-1.624295	3.047791
31	6	0	1.471809	-0.126443	3.165586
32	8	0	1.980049	-0.444241	4.270225
33	8	0	0.443551	0.574273	2.959836
34	6	0	4.201324	-2.150813	-1.612297
35	8	0	4.035073	-1.585172	-2.699594
36	7	0	4.998573	-3.243038	-1.485301
37	6	0	5.671570	-3.830955	-2.632396
38	1	0	6.429582	-4.530138	-2.276932
39	1	0	4.974168	-4.366658	-3.287540
40	1	0	6.153661	-3.045762	-3.218020
41	1	0	4.966301	-3.776478	-0.630068
42	6	0	-3.635812	-4.179212	-0.178389
43	1	0	-3.846099	-5.207800	0.129111
44	1	0	-3.435407	-4.190917	-1.256061
45	6	0	-4.343263	0.941744	-0.741517
46	6	0	-5.480708	0.119358	-1.348278
47	6	0	-2.431686	-3.619339	0.578194
48	1	0	-6.378829	0.732726	-1.466167
49	1	0	-5.195971	-0.241175	-2.343388
50	1	0	-4.713304	1.496723	0.131661
51	1	0	-3.993472	1.696539	-1.451542
52	1	0	-1.517503	-4.163197	0.319876
53	1	0	-2.587182	-3.768673	1.655959
54	1	0	-6.566449	-1.703092	-0.914319
55	1	0	-6.189076	-0.735023	0.513574
56	9	0	-1.690910	4.527634	-0.942628
57	9	0	2.994754	4.579175	-0.096313
58	1	0	4.917545	-2.510953	1.078255

144bba_Rh_Julolidin5COOH_F2-2H+carb_B3LYP631dp_PCMw_ring.log

Standard orientation:

Center Number	Atomic Number	Atomic Type	Coordinates (Angstroms) X	Y	Z
1	6	0	-2.441461	-1.920653	0.515727
2	6	0	-1.176982	-1.419038	0.781059
3	6	0	-0.812212	-0.087287	0.531045
4	6	0	-1.798047	0.741039	-0.016293
5	6	0	-3.089442	0.289951	-0.322791
6	6	0	-3.424504	-1.057067	-0.050282
7	6	0	0.537163	0.455694	0.914849
8	6	0	-0.285079	2.555925	-0.195111
9	6	0	0.777968	1.808089	0.334244
10	6	0	2.041221	2.440892	0.378468
11	1	0	2.903202	1.920227	0.783708
12	6	0	2.205190	3.721853	-0.086611
13	6	0	1.158322	4.547718	-0.644703
14	6	0	-0.092248	3.849117	-0.666890
15	1	0	-0.440317	-2.091283	1.213214
16	8	0	-1.566153	2.063285	-0.302419
17	7	0	-4.687841	-1.544231	-0.372497
18	6	0	-5.110130	-2.849374	0.129114
19	1	0	-5.962900	-3.175299	-0.475378
20	1	0	-5.464320	-2.778967	1.172477

21	6	0	-5.760038	-0.596523	-0.660648
22	8	0	1.328443	5.727966	-1.071209
23	6	0	1.681973	-0.523377	0.718577
24	6	0	2.235952	-0.882477	1.941545
25	6	0	2.191202	-1.044751	-0.464436
26	6	0	3.309364	-1.766657	2.033478
27	6	0	3.257833	-1.951576	-0.394420
28	1	0	1.788982	-0.770785	-1.433258
29	6	0	3.816369	-2.301316	0.851581
30	1	0	3.738629	-2.020807	2.996999
31	6	0	1.507965	-0.175214	3.020710
32	8	0	1.700004	-0.228309	4.222572
33	8	0	0.544275	0.582721	2.462264
34	6	0	3.785060	-2.490254	-1.700248
35	8	0	3.646296	-1.859355	-2.753359
36	7	0	4.415846	-3.690118	-1.649785
37	6	0	4.934738	-4.327462	-2.850309
38	1	0	5.633715	-5.112786	-2.559845
39	1	0	4.135411	-4.768955	-3.457129
40	1	0	5.456723	-3.586967	-3.458704
41	1	0	4.346645	-4.246276	-0.810972
42	6	0	-3.972336	-3.861477	0.037142
43	1	0	-4.314126	-4.833685	0.405764
44	1	0	-3.690316	-3.988182	-1.014776
45	6	0	-4.090972	1.251401	-0.931247
46	6	0	-5.260322	0.510189	-1.583106
47	6	0	-2.776635	-3.361727	0.849779
48	1	0	-6.076810	1.203792	-1.807074
49	1	0	-4.941638	0.062062	-2.531475
50	1	0	-4.471161	1.928355	-0.153077
51	1	0	-3.589342	1.894519	-1.660910
52	1	0	-1.899846	-3.996442	0.680470
53	1	0	-3.013479	-3.447141	1.919975
54	1	0	-6.576056	-1.151794	-1.133729
55	1	0	-6.163392	-0.158908	0.269187
56	9	0	-1.170874	4.503432	-1.191695
57	9	0	3.449906	4.285915	-0.027219
58	1	0	4.670683	-2.968629	0.896603

151aba_Rh_Julolidin5COOH_Et_B3LYP631dp_PCMw.log

Standard orientation:

Center Number	Atomic Number	Atomic Type	Coordinates (Angstroms) X	Y	Z
1	6	0	-2.863467	-1.487295	0.450378
2	6	0	-1.525686	-1.220441	0.495895
3	6	0	-0.984503	0.056749	0.156830
4	6	0	-1.927663	1.056773	-0.241845
5	6	0	-3.289264	0.839024	-0.330149
6	6	0	-3.787526	-0.452609	0.018534
7	6	0	0.375408	0.371872	0.210154
8	6	0	-0.176237	2.629035	-0.529819
9	6	0	0.802763	1.682615	-0.146064
10	6	0	2.158649	2.110567	-0.148349
11	1	0	2.918444	1.393845	0.136066
12	6	0	2.532664	3.389794	-0.499234
13	6	0	1.499840	4.302709	-0.875181
14	6	0	0.158585	3.927817	-0.892509
15	1	0	-0.844468	-2.001681	0.813881
16	1	0	-0.621796	4.622724	-1.183238
17	8	0	-1.493935	2.306911	-0.564189
18	7	0	-5.113396	-0.716805	-0.051627
19	6	0	-5.685113	-2.017395	0.333795
20	1	0	-6.579462	-2.170159	-0.275691
21	1	0	-6.009325	-1.971121	1.382983
22	6	0	-6.087467	0.346070	-0.346031
23	8	0	1.900968	5.544266	-1.214449
24	1	0	1.140759	6.094489	-1.456068

25	6	0	1.387626	-0.681311	0.538520
26	6	0	1.999329	-0.849147	1.802472
27	6	0	1.763717	-1.528376	-0.508940
28	6	0	2.969564	-1.847603	1.964268
29	6	0	2.715446	-2.537787	-0.333595
30	1	0	1.319760	-1.407133	-1.490497
31	6	0	3.328976	-2.685344	0.915668
32	1	0	3.438177	-1.950380	2.935846
33	6	0	1.693769	-0.038951	3.022458
34	8	0	2.329154	-0.115691	4.057900
35	8	0	0.636325	0.779667	2.881944
36	1	0	0.527390	1.260844	3.721002
37	6	0	3.046234	-3.387643	-1.535246
38	8	0	2.847635	-2.971019	-2.680693
39	7	0	3.570438	-4.610664	-1.283170
40	6	0	3.901532	-5.532042	-2.360054
41	1	0	4.524152	-6.332395	-1.959115
42	1	0	3.003662	-5.970334	-2.810761
43	1	0	4.452669	-5.002610	-3.139376
44	1	0	3.555990	-4.970173	-0.340716
45	6	0	-4.696360	-3.156425	0.129779
46	1	0	-5.139625	-4.083854	0.502328
47	1	0	-4.503143	-3.288660	-0.940632
48	6	0	-4.216618	1.943274	-0.787200
49	6	0	-5.521265	1.361452	-1.329166
50	6	0	-3.397614	-2.838044	0.867326
51	1	0	-6.257687	2.152889	-1.492609
52	1	0	-5.344877	0.871048	-2.292840
53	1	0	-4.429843	2.617382	0.053189
54	1	0	-3.721124	2.550571	-1.549009
55	1	0	-2.639044	-3.605316	0.688846
56	1	0	-3.590881	-2.831599	1.948953
57	1	0	-6.979282	-0.133433	-0.755249
58	1	0	-6.380146	0.836200	0.593018
59	6	0	3.966796	3.875033	-0.511892
60	1	0	4.195038	4.242440	-1.520687
61	1	0	4.033565	4.761572	0.131586
62	6	0	5.019779	2.851053	-0.087300
63	1	0	4.857187	2.502710	0.937860
64	1	0	5.025033	1.976494	-0.745920
65	1	0	6.014402	3.303132	-0.129757
66	1	0	4.102633	-3.428825	1.075067

152aba_Rh_Julolidin5COOH_Et-H+carb_B3LYP631dp_PCMw.log

Standard orientation:

Center Number	Atomic Number	Atomic Type	Coordinates (Angstroms) X	Y	Z
1	6	0	-2.862490	-1.512198	0.405503
2	6	0	-1.526578	-1.228269	0.506632
3	6	0	-0.988731	0.055619	0.216026
4	6	0	-1.919924	1.046551	-0.199708
5	6	0	-3.280293	0.814724	-0.342364
6	6	0	-3.778197	-0.482558	-0.032668
7	6	0	0.376255	0.384366	0.363661
8	6	0	-0.173902	2.628565	-0.445599
9	6	0	0.798645	1.694656	-0.035251
10	6	0	2.149598	2.127749	-0.019724
11	1	0	2.902395	1.417781	0.298910
12	6	0	2.529733	3.402301	-0.388732
13	6	0	1.503395	4.304551	-0.795530
14	6	0	0.165211	3.924183	-0.826167
15	1	0	-0.847393	-2.007696	0.834804
16	1	0	-0.612785	4.611195	-1.142063
17	8	0	-1.494575	2.308907	-0.499003
18	7	0	-5.106550	-0.758951	-0.159566
19	6	0	-5.674073	-2.062435	0.208162
20	1	0	-6.561802	-2.217396	-0.411512

21	1	0	-6.010097	-2.037943	1.255490
22	6	0	-6.074062	0.307778	-0.442752
23	8	0	1.905761	5.546055	-1.152738
24	6	0	1.391824	-0.690249	0.594658
25	6	0	1.895419	-0.949658	1.878656
26	6	0	1.845829	-1.440150	-0.494393
27	6	0	2.851381	-1.952996	2.049539
28	6	0	2.791339	-2.457770	-0.317519
29	1	0	1.474559	-1.248091	-1.495335
30	6	0	3.300578	-2.703984	0.966673
31	1	0	3.233175	-2.123673	3.050506
32	6	0	1.379447	-0.124700	3.052452
33	8	0	1.834072	-0.366823	4.192348
34	8	0	0.514097	0.746737	2.729476
35	6	0	3.224276	-3.216218	-1.542977
36	8	0	3.104007	-2.733424	-2.674909
37	7	0	3.754610	-4.448656	-1.336916
38	6	0	4.174770	-5.291555	-2.445267
39	1	0	4.807753	-6.092341	-2.060763
40	1	0	3.320267	-5.735277	-2.970075
41	1	0	4.743213	-4.696078	-3.161833
42	1	0	3.669392	-4.872950	-0.425874
43	6	0	-4.673786	-3.192136	0.000716
44	1	0	-5.117445	-4.131302	0.343051
45	1	0	-4.458077	-3.298596	-1.068534
46	6	0	-4.199119	1.919007	-0.819091
47	6	0	-5.490357	1.341434	-1.397655
48	6	0	-3.391629	-2.881568	0.770987
49	1	0	-6.224866	2.133811	-1.567145
50	1	0	-5.290994	0.865073	-2.364159
51	1	0	-4.434921	2.591339	0.017045
52	1	0	-3.685479	2.531189	-1.565312
53	1	0	-2.622828	-3.636814	0.582410
54	1	0	-3.604983	-2.914248	1.848479
55	1	0	-6.961188	-0.160372	-0.876439
56	1	0	-6.384358	0.785619	0.498251
57	6	0	3.964019	3.889759	-0.383387
58	1	0	4.211295	4.242992	-1.392921
59	1	0	4.020661	4.785391	0.248637
60	6	0	5.010956	2.873487	0.074155
61	1	0	4.831341	2.540447	1.101575
62	1	0	5.026815	1.988420	-0.570205
63	1	0	6.006443	3.324908	0.041336
64	1	0	1.143772	6.084830	-1.412261
65	1	0	4.065606	-3.458295	1.123702

152bba_Rh_Julolidin5CCOH_Et-H+carb_B3LYP631dp_PCMw_ring.log

Standard orientation:

Center Number	Atomic Number	Atomic Type	Coordinates (Angstroms) X	Y	Z
1	6	0	3.018290	1.217044	0.626259
2	6	0	1.670485	1.006475	0.866422
3	6	0	1.004499	-0.175484	0.507971
4	6	0	1.769374	-1.158220	-0.126317
5	6	0	3.130831	-1.000813	-0.415021
6	6	0	3.773456	0.200161	-0.030280
7	6	0	-0.439213	-0.407425	0.865516
8	6	0	-0.120057	-2.564587	-0.403265
9	6	0	-0.978034	-1.657916	0.218968
10	6	0	-2.345429	-1.988785	0.275643
11	1	0	-3.018923	-1.290704	0.760290
12	6	0	-2.867072	-3.160335	-0.256717
13	6	0	-1.957072	-4.048175	-0.880751
14	6	0	-0.600269	-3.755646	-0.956811
15	1	0	1.110358	1.792091	1.366727
16	1	0	0.097376	-4.432639	-1.440177
17	8	0	1.227200	-2.359768	-0.527828

18	7	0	5.116121	0.402418	-0.325271
19	6	0	5.831790	1.525288	0.275989
20	1	0	6.737700	1.693891	-0.315082
21	1	0	6.157602	1.283266	1.302337
22	6	0	5.937589	-0.734026	-0.731911
23	8	0	-2.481227	-5.192620	-1.404258
24	6	0	-1.326532	0.813057	0.678350
25	6	0	-1.821391	1.234740	1.906848
26	6	0	-1.667276	1.485019	-0.488409
27	6	0	-2.671009	2.333518	2.021189
28	6	0	-2.506126	2.604282	-0.394344
29	1	0	-1.307418	1.171584	-1.461910
30	6	0	-3.009173	3.017185	0.855797
31	1	0	-3.060987	2.637627	2.986775
32	6	0	-1.304366	0.332376	2.959100
33	8	0	-1.502568	0.360035	4.157500
34	8	0	-0.519689	-0.603282	2.368817
35	6	0	-2.859931	3.302405	-1.683717
36	8	0	-2.828026	2.700866	-2.762206
37	7	0	-3.213673	4.607906	-1.589450
38	6	0	-3.542580	5.391892	-2.770355
39	1	0	-4.045624	6.307583	-2.457535
40	1	0	-2.648494	5.656060	-3.347146
41	1	0	-4.208860	4.817129	-3.415946
42	1	0	-3.054046	5.100575	-0.723580
43	6	0	4.963639	2.779237	0.290892
44	1	0	5.522385	3.608040	0.736357
45	1	0	4.722470	3.060600	-0.740901
46	6	0	3.881692	-2.110368	-1.123829
47	6	0	5.188518	-1.603065	-1.736310
48	6	0	3.682019	2.503590	1.078696
49	1	0	5.819448	-2.443180	-2.042567
50	1	0	4.978692	-1.007170	-2.632198
51	1	0	4.096779	-2.921522	-0.414168
52	1	0	3.245506	-2.552814	-1.896162
53	1	0	2.978977	3.338103	0.982200
54	1	0	3.929050	2.433786	2.147553
55	1	0	6.855467	-0.337753	-1.177228
56	1	0	6.237235	-1.338408	0.141658
57	6	0	-4.336375	-3.530356	-0.205965
58	1	0	-4.687346	-3.700250	-1.232417
59	1	0	-4.431707	-4.506820	0.286987
60	6	0	-5.256108	-2.521508	0.483773
61	1	0	-4.979239	-2.370095	1.532339
62	1	0	-5.235050	-1.546496	-0.014265
63	1	0	-6.288702	-2.881622	0.462867
64	1	0	-1.777207	-5.719402	-1.808706
65	1	0	-3.693123	3.857055	0.916260

153aaa_Rh_Julolidin5COOH_Et-H+oh_B3LYP631dp_PCMw.log

Standard orientation:

Center Number	Atomic Number	Atomic Type	Coordinates (Angstroms) X	Y	Z
1	6	0	-2.854464	-1.557428	0.256547
2	6	0	-1.516892	-1.251237	0.365531
3	6	0	-1.005489	0.043617	0.110395
4	6	0	-1.946264	1.023301	-0.288122
5	6	0	-3.305014	0.769296	-0.438645
6	6	0	-3.782027	-0.540305	-0.158114
7	6	0	0.369059	0.408808	0.227647
8	6	0	-0.227202	2.672719	-0.459067
9	6	0	0.766261	1.708767	-0.061526
10	6	0	2.123765	2.183114	0.024714
11	1	0	2.878680	1.467684	0.331253
12	6	0	2.477770	3.467947	-0.257096
13	6	0	1.447250	4.448194	-0.673339
14	6	0	0.085339	3.969951	-0.752507

15	1	0	-0.828558	-2.033861	0.667974
16	1	0	-0.691963	4.664750	-1.051645
17	8	0	-1.541513	2.302047	-0.554323
18	7	0	-5.114252	-0.837048	-0.302503
19	6	0	-5.656273	-2.139623	0.091688
20	1	0	-6.566400	-2.306368	-0.492317
21	1	0	-5.952262	-2.123678	1.152550
22	6	0	-6.092729	0.227264	-0.534464
23	8	0	1.755671	5.631783	-0.939177
24	6	0	1.382063	-0.642732	0.565193
25	6	0	1.713872	-1.065753	1.873563
26	6	0	2.017172	-1.262882	-0.516632
27	6	0	2.661513	-2.085970	2.045283
28	6	0	2.949696	-2.289308	-0.340109
29	1	0	1.784134	-0.948636	-1.527755
30	6	0	3.276652	-2.695213	0.959333
31	1	0	2.910543	-2.385504	3.056418
32	6	0	1.140442	-0.506036	3.137340
33	8	0	1.360709	-0.975600	4.240108
34	8	0	0.362629	0.576106	2.961752
35	1	0	0.046675	0.845950	3.841973
36	6	0	3.573126	-2.877815	-1.580089
37	8	0	3.630264	-2.232337	-2.631625
38	7	0	4.063953	-4.136265	-1.470340
39	6	0	4.656621	-4.820654	-2.609761
40	1	0	5.200447	-5.695432	-2.251911
41	1	0	3.897013	-5.143358	-3.331204
42	1	0	5.349698	-4.149238	-3.119803
43	1	0	3.842154	-4.683094	-0.651932
44	6	0	-4.651648	-3.258980	-0.154758
45	1	0	-5.077380	-4.208068	0.183889
46	1	0	-4.462092	-3.346976	-1.230788
47	6	0	-4.239851	1.870435	-0.892458
48	6	0	-5.531306	1.291208	-1.470390
49	6	0	-3.352602	-2.948657	0.587886
50	1	0	-6.275737	2.079532	-1.615634
51	1	0	-5.336296	0.838737	-2.449396
52	1	0	-4.475530	2.529616	-0.045514
53	1	0	-3.737677	2.499655	-1.632713
54	1	0	-2.578347	-3.685227	0.350793
55	1	0	-3.533039	-3.025491	1.669343
56	1	0	-6.984900	-0.233588	-0.967436
57	1	0	-6.394316	0.681912	0.422346
58	6	0	3.891495	3.990749	-0.174187
59	1	0	4.153222	4.413937	-1.153348
60	1	0	3.892479	4.857022	0.500899
61	6	0	4.961524	2.990391	0.262581
62	1	0	4.761503	2.594440	1.264082
63	1	0	5.028003	2.140716	-0.425464
64	1	0	5.943131	3.472915	0.287723
65	1	0	4.025672	-3.460153	1.135008

154aaa_Rh_Julolidin5COOH_Et-2H+_B3LYP631dp_PCMw.log

Standard orientation:

Center Number	Atomic Number	Atomic Type	Coordinates (Angstroms) X	Y	Z
1	6	0	-2.865019	-1.527691	0.313618
2	6	0	-1.527023	-1.223862	0.439801
3	6	0	-1.005285	0.062746	0.174473
4	6	0	-1.934282	1.039979	-0.246800
5	6	0	-3.294142	0.788972	-0.415040
6	6	0	-3.780513	-0.513483	-0.127533
7	6	0	0.374909	0.416274	0.314510
8	6	0	-0.203962	2.671960	-0.432687
9	6	0	0.777679	1.708414	-0.019764
10	6	0	2.137867	2.172887	0.048764
11	1	0	2.885408	1.455472	0.368702

12	6	0	2.504609	3.448959	-0.262256
13	6	0	1.484571	4.430470	-0.692327
14	6	0	0.120227	3.962133	-0.755690
15	1	0	-0.844003	-2.000739	0.767972
16	1	0	-0.652840	4.655487	-1.069817
17	8	0	-1.523690	2.315286	-0.523451
18	7	0	-5.114806	-0.809164	-0.295841
19	6	0	-5.666855	-2.094249	0.135041
20	1	0	-6.580062	-2.269776	-0.441978
21	1	0	-5.960138	-2.051026	1.196553
22	6	0	-6.082852	0.260243	-0.539461
23	8	0	1.803912	5.607782	-0.985424
24	6	0	1.385018	-0.651756	0.603840
25	6	0	1.750668	-1.053875	1.904336
26	6	0	1.971891	-1.280146	-0.502107
27	6	0	2.699460	-2.074184	2.046228
28	6	0	2.905221	-2.311065	-0.349565
29	1	0	1.704631	-0.974093	-1.508160
30	6	0	3.273941	-2.701674	0.945785
31	1	0	2.976326	-2.352599	3.057076
32	6	0	1.157257	-0.401783	3.166039
33	8	0	1.549742	-0.861632	4.269240
34	8	0	0.334331	0.531766	2.968457
35	6	0	3.476646	-2.921732	-1.598211
36	8	0	3.472735	-2.315205	-2.676241
37	7	0	4.001029	-4.169372	-1.478686
38	6	0	4.546152	-4.877280	-2.625955
39	1	0	5.137157	-5.723119	-2.272175
40	1	0	3.758089	-5.248864	-3.292002
41	1	0	5.187513	-4.205539	-3.199458
42	1	0	3.823580	-4.697022	-0.637523
43	6	0	-4.670765	-3.227173	-0.084753
44	1	0	-5.105459	-4.166598	0.269649
45	1	0	-4.477699	-3.336793	-1.158282
46	6	0	-4.216746	1.889652	-0.895448
47	6	0	-5.506395	1.312450	-1.479900
48	6	0	-3.372190	-2.913396	0.657706
49	1	0	-6.244584	2.103865	-1.640540
50	1	0	-5.304796	0.848948	-2.452517
51	1	0	-4.457557	2.563284	-0.061227
52	1	0	-3.702142	2.505321	-1.638810
53	1	0	-2.601003	-3.656682	0.430829
54	1	0	-3.555999	-2.979121	1.739424
55	1	0	-6.976602	-0.195839	-0.975103
56	1	0	-6.387930	0.726670	0.411257
57	6	0	3.925411	3.957443	-0.197635
58	1	0	4.186400	4.361983	-1.185062
59	1	0	3.942944	4.834487	0.463510
60	6	0	4.987447	2.952179	0.247922
61	1	0	4.790366	2.575086	1.257290
62	1	0	5.037913	2.089857	-0.425712
63	1	0	5.975370	3.422621	0.257613
64	1	0	4.027638	-3.467978	1.101944

154bba_Rh_Julolidin5CCOH_Et-2H+carb_B3LYP631dp_PCMw_ring.log

Standard orientation:

Center Number	Atomic Number	Atomic Type	Coordinates (Angstroms) X	Y	Z
1	6	0	2.962563	1.295842	0.548179
2	6	0	1.620043	1.045919	0.786395
3	6	0	0.997125	-0.169905	0.467135
4	6	0	1.799655	-1.154239	-0.121839
5	6	0	3.160063	-0.955032	-0.402571
6	6	0	3.756502	0.281042	-0.061463
7	6	0	-0.436544	-0.449277	0.817010
8	6	0	-0.055310	-2.624727	-0.390006
9	6	0	-0.937446	-1.694099	0.178612

10	6	0	-2.308423	-2.060510	0.198042
11	1	0	-3.008262	-1.357533	0.642265
12	6	0	-2.785843	-3.250310	-0.310661
13	6	0	-1.866172	-4.219439	-0.906147
14	6	0	-0.484350	-3.832785	-0.918537
15	1	0	1.028673	1.829644	1.252932
16	1	0	0.243718	-4.506216	-1.362209
17	8	0	1.307154	-2.382723	-0.474408
18	7	0	5.097164	0.520423	-0.358342
19	6	0	5.767022	1.678554	0.226861
20	1	0	6.682864	1.855649	-0.346558
21	1	0	6.073865	1.477269	1.268386
22	6	0	5.959883	-0.608998	-0.690079
23	8	0	-2.279317	-5.323095	-1.385390
24	6	0	-1.356707	0.751264	0.682176
25	6	0	-1.830411	1.160491	1.923227
26	6	0	-1.745332	1.420667	-0.472194
27	6	0	-2.702321	2.239091	2.060670
28	6	0	-2.603811	2.522548	-0.355470
29	1	0	-1.404393	1.114586	-1.454824
30	6	0	-3.085072	2.920874	0.907978
31	1	0	-3.075194	2.530037	3.037252
32	6	0	-1.267894	0.271767	2.968294
33	8	0	-1.458127	0.316706	4.172815
34	8	0	-0.477967	-0.639508	2.379057
35	6	0	-3.000498	3.220222	-1.631292
36	8	0	-2.989656	2.626842	-2.715032
37	7	0	-3.368350	4.521468	-1.520774
38	6	0	-3.735830	5.308060	-2.688127
39	1	0	-4.239044	6.217449	-2.357461
40	1	0	-2.860328	5.583979	-3.287702
41	1	0	-4.413710	4.730430	-3.319004
42	1	0	-3.189328	5.010969	-0.656948
43	6	0	4.864071	2.907162	0.183037
44	1	0	5.390707	3.767216	0.608368
45	1	0	4.634692	3.144815	-0.862456
46	6	0	3.955943	-2.066404	-1.057468
47	6	0	5.258751	-1.546127	-1.667809
48	6	0	3.576211	2.621814	0.957231
49	1	0	5.921752	-2.378287	-1.924769
50	1	0	5.048966	-0.997020	-2.593271
51	1	0	4.181580	-2.845216	-0.315167
52	1	0	3.344810	-2.556485	-1.821491
53	1	0	2.849351	3.428707	0.811864
54	1	0	3.804362	2.607198	2.032703
55	1	0	6.877011	-0.208063	-1.133348
56	1	0	6.256108	-1.163700	0.217659
57	6	0	-4.248655	-3.637586	-0.296428
58	1	0	-4.560571	-3.854461	-1.328364
59	1	0	-4.347139	-4.605248	0.216357
60	6	0	-5.218453	-2.631470	0.325823
61	1	0	-4.983271	-2.438413	1.378552
62	1	0	-5.196688	-1.669300	-0.198249
63	1	0	-6.245408	-3.008581	0.281082
64	1	0	-3.786088	3.745121	0.988386

161aaa_Rh_Julolidin5COOH_naft1_B3LYP631dp_PCMw.log

Standard orientation:

Center Number	Atomic Number	Atomic Type	Coordinates (Angstroms) X	Y	Z
1	6	0	-3.018115	-1.386240	0.272816
2	6	0	-1.668113	-1.191800	0.373644
3	6	0	-1.053170	0.073668	0.134404
4	6	0	-1.932897	1.119243	-0.260466
5	6	0	-3.301663	0.985458	-0.402097
6	6	0	-3.877208	-0.288356	-0.125769
7	6	0	0.326287	0.328622	0.265553

8	6	0	-0.089749	2.601953	-0.444190
9	6	0	0.841930	1.633124	0.007235
10	6	0	2.230994	2.087907	0.136766
11	6	0	2.583259	3.408978	-0.289324
12	6	0	1.565594	4.294769	-0.789849
13	6	0	0.248592	3.900252	-0.841288
14	1	0	-1.046959	-2.030789	0.662995
15	1	0	-0.536401	4.559840	-1.193355
16	8	0	-1.410067	2.340124	-0.548809
17	7	0	-5.214425	-0.478792	-0.245761
18	6	0	-5.860034	-1.762442	0.068423
19	1	0	-6.751581	-1.838254	-0.559292
20	1	0	-6.197716	-1.752990	1.114620
21	6	0	-6.119107	0.648722	-0.513672
22	8	0	1.972804	5.514475	-1.176821
23	1	0	1.222261	6.041622	-1.490343
24	6	0	1.193418	-0.862791	0.547682
25	6	0	1.387313	-1.432680	1.827144
26	6	0	1.759732	-1.493993	-0.563736
27	6	0	2.149996	-2.602131	1.947222
28	6	0	2.512404	-2.665739	-0.437994
29	1	0	1.620798	-1.076972	-1.554721
30	6	0	2.713726	-3.214442	0.834183
31	1	0	2.299569	-3.015850	2.937550
32	6	0	0.861509	-0.861025	3.104873
33	8	0	0.845681	-1.467710	4.159609
34	8	0	0.427190	0.406648	2.988035
35	1	0	0.105034	0.688027	3.862403
36	6	0	3.089601	-3.249945	-1.703915
37	8	0	3.271592	-2.543883	-2.700530
38	7	0	3.396170	-4.568846	-1.676356
39	6	0	3.932092	-5.248641	-2.846394
40	1	0	4.363024	-6.200691	-2.534848
41	1	0	3.157187	-5.437170	-3.598437
42	1	0	4.709092	-4.632155	-3.301413
43	1	0	3.074601	-5.136009	-0.906528
44	6	0	-4.927278	-2.939984	-0.179702
45	1	0	-5.424320	-3.861842	0.134273
46	1	0	-4.719917	-3.024421	-1.252358
47	6	0	-4.151271	2.156259	-0.844192
48	6	0	-5.472004	1.670216	-1.440152
49	6	0	-3.628209	-2.730905	0.596339
50	1	0	-6.158683	2.508049	-1.588089
51	1	0	-5.297869	1.209625	-2.418843
52	1	0	-4.349079	2.812327	0.014054
53	1	0	-3.602030	2.760148	-1.571284
54	1	0	-2.906287	-3.524846	0.384678
55	1	0	-3.842128	-2.778992	1.672991
56	1	0	-7.026655	0.239620	-0.963312
57	1	0	-6.407199	1.117502	0.437907
58	6	0	3.915267	3.870473	-0.196719
59	6	0	3.273174	1.309688	0.694083
60	6	0	4.906522	3.065851	0.327425
61	1	0	5.927315	3.426619	0.397200
62	6	0	4.571705	1.782319	0.785222
63	1	0	5.334240	1.146241	1.223552
64	1	0	4.140823	4.873825	-0.536668
65	1	0	3.070427	0.323838	1.076212
66	1	0	3.326842	-4.098387	0.972684

162aaa_Rh_Julolidin5COOH_naft1-H+carb_B3LYP631dp_PCMw.log

Standard orientation:

Center Number	Atomic Number	Atomic Type	Coordinates (Angstroms) X	Y	Z
1	6	0	-3.043274	-1.313006	0.375090
2	6	0	-1.688973	-1.134761	0.494352
3	6	0	-1.048823	0.105581	0.221780

4	6	0	-1.897983	1.149053	-0.215997
5	6	0	-3.270913	1.031057	-0.380273
6	6	0	-3.872887	-0.219535	-0.074213
7	6	0	0.343324	0.335047	0.396006
8	6	0	-0.027485	2.586378	-0.436906
9	6	0	0.876338	1.625699	0.062438
10	6	0	2.269537	2.056214	0.182538
11	6	0	2.661994	3.341597	-0.312117
12	6	0	1.670284	4.222729	-0.866611
13	6	0	0.346555	3.856983	-0.901982
14	1	0	-1.084902	-1.968620	0.831975
15	1	0	-0.420900	4.514861	-1.294471
16	8	0	-1.356288	2.355531	-0.542313
17	7	0	-5.217825	-0.393300	-0.221182
18	6	0	-5.888246	-1.644965	0.150449
19	1	0	-6.781311	-1.734170	-0.474469
20	1	0	-6.227982	-1.589304	1.195567
21	6	0	-6.093075	0.745445	-0.519008
22	8	0	2.110492	5.412614	-1.320442
23	6	0	1.192781	-0.888430	0.574664
24	6	0	1.420418	-1.443899	1.844550
25	6	0	1.690310	-1.528629	-0.562913
26	6	0	2.155525	-2.625735	1.952045
27	6	0	2.420550	-2.718779	-0.452371
28	1	0	1.522113	-1.115798	-1.551765
29	6	0	2.657498	-3.261984	0.819049
30	1	0	2.332637	-3.023641	2.945630
31	6	0	0.911839	-0.701812	3.077264
32	8	0	1.046222	-1.250241	4.193531
33	8	0	0.400827	0.431864	2.823046
34	6	0	2.930271	-3.334709	-1.727304
35	8	0	3.082521	-2.659748	-2.752002
36	7	0	3.215969	-4.661057	-1.688702
37	6	0	3.681859	-5.372526	-2.868520
38	1	0	4.089867	-6.336457	-2.561901
39	1	0	2.874230	-5.542261	-3.590547
40	1	0	4.463551	-4.791693	-3.361572
41	1	0	2.911308	-5.206360	-0.896867
42	6	0	-4.976947	-2.850126	-0.044335
43	1	0	-5.494350	-3.750716	0.298210
44	1	0	-4.762258	-2.977703	-1.111496
45	6	0	-4.090495	2.202057	-0.877919
46	6	0	-5.416926	1.725700	-1.470246
47	6	0	-3.679663	-2.637635	0.734820
48	1	0	-6.083657	2.573392	-1.651693
49	1	0	-5.244021	1.230759	-2.432502
50	1	0	-4.282692	2.897129	-0.049281
51	1	0	-3.520348	2.765733	-1.621489
52	1	0	-2.970577	-3.450557	0.551853
53	1	0	-3.902871	-2.653217	1.810715
54	1	0	-7.009407	0.348412	-0.963186
55	1	0	-6.376899	1.251612	0.415740
56	6	0	4.006328	3.770816	-0.227657
57	6	0	3.280394	1.284607	0.805435
58	6	0	4.966845	2.970955	0.356509
59	1	0	5.996802	3.306729	0.419805
60	6	0	4.589584	1.726089	0.887098
61	1	0	5.328860	1.099419	1.376557
62	1	0	4.264547	4.746730	-0.620740
63	1	0	3.033639	0.332525	1.244473
64	1	0	1.371092	5.936264	-1.663751
65	1	0	3.257063	-4.159582	0.935532

162baa_Rh_Julolidin5COOH_naft1-H+carb_B3LYP631dp_PCMw_ring.log

Standard orientation:

Center Number	Atomic Number	Atomic Type	Coordinates (Angstroms) X	Y	Z

1	6	0	3.214843	0.797271	0.806171
2	6	0	1.848740	0.745455	1.030133
3	6	0	1.030631	-0.285726	0.541651
4	6	0	1.664120	-1.273281	-0.207143
5	6	0	3.035368	-1.270462	-0.493803
6	6	0	3.831663	-0.222290	0.022166
7	6	0	-0.452612	-0.334131	0.851915
8	6	0	-0.381867	-2.430069	-0.525538
9	6	0	-1.134431	-1.527951	0.212118
10	6	0	-2.550335	-1.808557	0.340110
11	6	0	-3.120649	-2.958137	-0.303700
12	6	0	-2.271021	-3.826374	-1.063051
13	6	0	-0.930458	-3.566037	-1.165685
14	1	0	1.403747	1.541289	1.620398
15	1	0	-0.263298	-4.209186	-1.729804
16	8	0	0.962749	-2.330143	-0.734260
17	7	0	5.190454	-0.166031	-0.263501
18	6	0	6.047305	0.770710	0.459482
19	1	0	6.968707	0.889674	-0.119639
20	1	0	6.336446	0.364412	1.444153
21	6	0	5.852625	-1.348983	-0.805498
22	8	0	-2.865150	-4.895038	-1.653857
23	6	0	-1.090238	1.027952	0.598733
24	6	0	-1.358134	1.653246	1.812528
25	6	0	-1.353880	1.671711	-0.601518
26	6	0	-1.900615	2.934789	1.876273
27	6	0	-1.889026	2.967715	-0.560159
28	1	0	-1.165214	1.205023	-1.561754
29	6	0	-2.163093	3.589077	0.674278
30	1	0	-2.122545	3.399539	2.831083
31	6	0	-1.030618	0.713075	2.907754
32	8	0	-1.137163	0.864368	4.107743
33	8	0	-0.580049	-0.443917	2.354183
34	6	0	-2.181645	3.626610	-1.885393
35	8	0	-2.387253	2.950908	-2.898777
36	7	0	-2.210491	4.981998	-1.896318
37	6	0	-2.453310	5.726630	-3.122586
38	1	0	-2.714074	6.754213	-2.866534
39	1	0	-1.573409	5.735123	-3.776539
40	1	0	-3.280759	5.269505	-3.667772
41	1	0	-1.865362	5.488785	-1.095254
42	6	0	5.351768	2.116347	0.639169
43	1	0	6.014003	2.803963	1.174122
44	1	0	5.151431	2.551110	-0.347120
45	6	0	3.631159	-2.380773	-1.336006
46	6	0	4.999353	-1.986661	-1.896959
47	6	0	4.043273	1.917008	1.405671
48	1	0	5.512855	-2.863090	-2.304070
49	1	0	4.877982	-1.267979	-2.715742
50	1	0	3.728658	-3.291938	-0.729280
51	1	0	2.946313	-2.637608	-2.149795
52	1	0	3.458459	2.843022	1.424622
53	1	0	4.276686	1.680699	2.453551
54	1	0	6.818958	-1.032847	-1.210561
55	1	0	6.060652	-2.085375	-0.010207
56	6	0	-4.503274	-3.242334	-0.188988
57	6	0	-3.448085	-1.004868	1.095458
58	6	0	-5.335453	-2.427919	0.547619
59	1	0	-6.393935	-2.652882	0.631117
60	6	0	-4.793003	-1.301066	1.194964
61	1	0	-5.435761	-0.653382	1.783796
62	1	0	-4.892542	-4.118950	-0.693425
63	1	0	-3.087546	-0.128951	1.609183
64	1	0	-2.203670	-5.411581	-2.136309
65	1	0	-2.616201	4.574369	0.699269

163aaa_Rh_Julolidin5COOH_naft1-H+oh_B3LYP631dp_PCMw.log

Standard orientation:

Center Number	Atomic Number	Atomic Type	Coordinates (Angstroms) X	Y	Z
1	6	0	-3.025110	-1.402962	0.181098
2	6	0	-1.669308	-1.188691	0.304472
3	6	0	-1.072331	0.080501	0.110586
4	6	0	-1.947963	1.119504	-0.264487
5	6	0	-3.320733	0.968507	-0.421391
6	6	0	-3.885230	-0.314368	-0.187785
7	6	0	0.326992	0.364406	0.277799
8	6	0	-0.114297	2.644363	-0.431738
9	6	0	0.818157	1.650743	0.060750
10	6	0	2.205502	2.124958	0.244849
11	6	0	2.578726	3.411280	-0.236239
12	6	0	1.594761	4.342453	-0.853100
13	6	0	0.233655	3.895356	-0.864683
14	1	0	-1.042815	-2.031622	0.573436
15	1	0	-0.532936	4.556921	-1.252126
16	8	0	-1.447163	2.362838	-0.519886
17	7	0	-5.234144	-0.516395	-0.342275
18	6	0	-5.859414	-1.794992	0.002900
19	1	0	-6.780508	-1.877847	-0.581661
20	1	0	-6.149624	-1.803527	1.065491
21	6	0	-6.137901	0.619100	-0.534331
22	8	0	1.944062	5.453540	-1.301263
23	6	0	1.201201	-0.824848	0.547238
24	6	0	1.310223	-1.486071	1.792918
25	6	0	1.866407	-1.369530	-0.556274
26	6	0	2.084028	-2.651421	1.889920
27	6	0	2.630653	-2.535732	-0.456680
28	1	0	1.793047	-0.882986	-1.522207
29	6	0	2.744463	-3.173594	0.784740
30	1	0	2.165199	-3.133604	2.857080
31	6	0	0.677989	-1.021462	3.065592
32	8	0	0.546747	-1.726697	4.049879
33	8	0	0.290977	0.266340	3.041968
34	1	0	-0.105819	0.464314	3.908505
35	6	0	3.316398	-3.022936	-1.708004
36	8	0	3.581250	-2.247073	-2.631724
37	7	0	3.625260	-4.341919	-1.750973
38	6	0	4.262702	-4.935612	-2.916574
39	1	0	4.671200	-5.908025	-2.639256
40	1	0	3.556484	-5.069102	-3.744370
41	1	0	5.072225	-4.288244	-3.257909
42	1	0	3.237443	-4.962071	-1.055894
43	6	0	-4.930327	-2.966346	-0.295158
44	1	0	-5.415740	-3.899204	0.006244
45	1	0	-4.750563	-3.021179	-1.375082
46	6	0	-4.177240	2.144809	-0.839239
47	6	0	-5.504863	1.674099	-1.434434
48	6	0	-3.610513	-2.773429	0.451548
49	1	0	-6.193725	2.515785	-1.551207
50	1	0	-5.340313	1.243036	-2.428553
51	1	0	-4.369096	2.789848	0.029341
52	1	0	-3.632826	2.762504	-1.559020
53	1	0	-2.886848	-3.546726	0.174524
54	1	0	-3.790297	-2.889301	1.529628
55	1	0	-7.059321	0.235494	-0.981285
56	1	0	-6.408199	1.060496	0.437960
57	6	0	3.896513	3.873089	-0.094207
58	6	0	3.201875	1.386197	0.923171
59	6	0	4.863086	3.108390	0.539337
60	1	0	5.879044	3.476377	0.645399
61	6	0	4.498894	1.862573	1.063965
62	1	0	5.228565	1.259329	1.596084
63	1	0	4.120679	4.858576	-0.487897
64	1	0	2.964582	0.433579	1.368738
65	1	0	3.363371	-4.056202	0.905612

164aaa_Rh_Julolidin5COOH_naft1-2H_B3LYP631dp_PCMw.log

Standard orientation:

Center Number	Atomic Number	Atomic Type	Coordinates (Angstroms) X	Y	Z
1	6	0	-3.049814	-1.368454	0.252271
2	6	0	-1.693747	-1.162223	0.395566
3	6	0	-1.080139	0.092745	0.175485
4	6	0	-1.937542	1.128781	-0.239993
5	6	0	-3.310993	0.985364	-0.419078
6	6	0	-3.891084	-0.284178	-0.163510
7	6	0	0.323571	0.359049	0.364071
8	6	0	-0.088034	2.628596	-0.417537
9	6	0	0.821207	1.640811	0.112271
10	6	0	2.206954	2.104621	0.306131
11	6	0	2.609536	3.368563	-0.209551
12	6	0	1.648886	4.293652	-0.869438
13	6	0	0.283811	3.864544	-0.883912
14	1	0	-1.077129	-1.997086	0.708815
15	1	0	-0.469221	4.522481	-1.303436
16	8	0	-1.423347	2.361679	-0.523618
17	7	0	-5.241847	-0.480437	-0.345260
18	6	0	-5.884193	-1.734937	0.048241
19	1	0	-6.804109	-1.830621	-0.536756
20	1	0	-6.179380	-1.700969	1.109583
21	6	0	-6.128812	0.661573	-0.564627
22	8	0	2.021975	5.387281	-1.347482
23	6	0	1.195116	-0.843994	0.575734
24	6	0	1.319613	-1.516722	1.808603
25	6	0	1.833712	-1.360306	-0.557777
26	6	0	2.092915	-2.682039	1.861099
27	6	0	2.602169	-2.528147	-0.496335
28	1	0	1.741524	-0.855131	-1.513396
29	6	0	2.734502	-3.187133	0.734028
30	1	0	2.181855	-3.175130	2.822987
31	6	0	0.688312	-0.973746	3.101657
32	8	0	0.689543	-1.748242	4.092093
33	8	0	0.245017	0.204809	3.040458
34	6	0	3.261734	-2.994543	-1.763797
35	8	0	3.486021	-2.218962	-2.700869
36	7	0	3.603252	-4.308232	-1.821355
37	6	0	4.217304	-4.882824	-3.007534
38	1	0	4.645917	-5.852461	-2.750440
39	1	0	3.493662	-5.019366	-3.820250
40	1	0	5.009804	-4.222830	-3.365126
41	1	0	3.244608	-4.938787	-1.120514
42	6	0	-4.967219	-2.927763	-0.199466
43	1	0	-5.467116	-3.844238	0.128008
44	1	0	-4.777542	-3.021270	-1.275146
45	6	0	-4.147972	2.159177	-0.882688
46	6	0	-5.474696	1.689488	-1.481294
47	6	0	-3.652506	-2.725516	0.554178
48	1	0	-6.152667	2.535965	-1.625419
49	1	0	-5.304402	1.233511	-2.463371
50	1	0	-4.342642	2.832387	-0.036202
51	1	0	-3.587017	2.749486	-1.612942
52	1	0	-2.934043	-3.513520	0.305889
53	1	0	-3.844099	-2.810923	1.633161
54	1	0	-7.052583	0.282010	-1.010930
55	1	0	-6.401160	1.128200	0.395919
56	6	0	3.932870	3.812894	-0.054662
57	6	0	3.171799	1.376335	1.040014
58	6	0	4.871247	3.055188	0.627558
59	1	0	5.891253	3.409547	0.743143
60	6	0	4.472756	1.836511	1.192832
61	1	0	5.179831	1.245091	1.767666
62	1	0	4.182830	4.780501	-0.476673
63	1	0	2.893142	0.450867	1.518832
64	1	0	3.357589	-4.071737	0.827003

164bba_Rh_Julolidin5COOH_naft1-2H+carb_B3LYP631dp_PCMw_ring.log

Standard orientation:

Center Number	Atomic Number	Atomic Type	Coordinates (Angstroms) X	Y	Z
1	6	0	3.172934	0.914228	0.719777
2	6	0	1.808132	0.816359	0.941485
3	6	0	1.032900	-0.268214	0.501173
4	6	0	1.706532	-1.268732	-0.197451
5	6	0	3.081412	-1.219815	-0.475579
6	6	0	3.832399	-0.117856	-0.008790
7	6	0	-0.443242	-0.376693	0.817232
8	6	0	-0.307748	-2.507486	-0.513186
9	6	0	-1.084420	-1.578158	0.193469
10	6	0	-2.497410	-1.891230	0.298376
11	6	0	-3.029910	-3.057400	-0.345730
12	6	0	-2.176817	-3.991246	-1.115919
13	6	0	-0.802279	-3.649786	-1.142809
14	1	0	1.328515	1.620542	1.492080
15	1	0	-0.105112	-4.287577	-1.677152
16	8	0	1.056816	-2.370988	-0.675962
17	7	0	5.192988	-0.020595	-0.294030
18	6	0	6.004925	0.969852	0.407612
19	1	0	6.936546	1.091829	-0.154581
20	1	0	6.282105	0.615998	1.416254
21	6	0	5.901867	-1.208968	-0.758441
22	8	0	-2.663117	-5.009940	-1.689630
23	6	0	-1.128940	0.968295	0.607802
24	6	0	-1.392349	1.580551	1.829053
25	6	0	-1.442268	1.610235	-0.582284
26	6	0	-1.976143	2.842875	1.909654
27	6	0	-2.018209	2.887850	-0.525101
28	1	0	-1.259591	1.150952	-1.547361
29	6	0	-2.285817	3.495300	0.717774
30	1	0	-2.193526	3.294830	2.871888
31	6	0	-1.011265	0.649170	2.917847
32	8	0	-1.109079	0.814301	4.121349
33	8	0	-0.531144	-0.482521	2.366864
34	6	0	-2.361743	3.543913	-1.838223
35	8	0	-2.578548	2.870818	-2.851359
36	7	0	-2.423739	4.898929	-1.841367
37	6	0	-2.716863	5.641673	-3.057523
38	1	0	-2.955820	6.672241	-2.792603
39	1	0	-1.868433	5.640052	-3.751867
40	1	0	-3.573327	5.192398	-3.563693
41	1	0	-2.068331	5.409587	-1.047333
42	6	0	5.265620	2.300186	0.512832
43	1	0	5.897187	3.033415	1.024139
44	1	0	5.069023	2.680742	-0.496456
45	6	0	3.725773	-2.345586	-1.259782
46	6	0	5.086638	-1.932672	-1.824906
47	6	0	3.951238	2.095418	1.267998
48	1	0	5.636476	-2.809622	-2.180644
49	1	0	4.951519	-1.261202	-2.680916
50	1	0	3.846475	-3.225314	-0.611774
51	1	0	3.058633	-2.663298	-2.066939
52	1	0	3.334408	2.999925	1.225851
53	1	0	4.175287	1.928131	2.331335
54	1	0	6.864721	-0.884380	-1.165772
55	1	0	6.119958	-1.894685	0.079043
56	6	0	-4.401854	-3.360985	-0.248640
57	6	0	-3.426777	-1.108434	1.041166
58	6	0	-5.278384	-2.567485	0.468501
59	1	0	-6.333613	-2.817661	0.533883
60	6	0	-4.770496	-1.430870	1.121931
61	1	0	-5.433185	-0.794914	1.703607
62	1	0	-4.735360	-4.256756	-0.763445
63	1	0	-3.092522	-0.227678	1.567293

64	1	0	-2.770814	4.465016	0.756938

171aaa_Rh_Julolidin5COOH_naft2_B3LYP631dp_PCMw.log

Standard orientation:

Center Number	Atomic Number	Atomic Type	Coordinates (Angstroms) X	Y	Z
1	6	0	-0.322001	3.324732	0.245912
2	6	0	-0.806080	2.055256	0.377832
3	6	0	0.020618	0.901250	0.229382
4	6	0	1.396818	1.144296	-0.072982
5	6	0	1.939573	2.403086	-0.245190
6	6	0	1.077864	3.530208	-0.086828
7	6	0	-0.428395	-0.413192	0.369585
8	6	0	1.830695	-1.191371	-0.089857
9	6	0	0.487084	-1.491460	0.197257
10	6	0	0.118788	-2.873097	0.304492
11	1	0	-0.913614	-3.120505	0.520305
12	6	0	1.041828	-3.862595	0.137475
13	6	0	2.412674	-3.560910	-0.150159
14	6	0	2.818774	-2.193776	-0.267301
15	1	0	-1.853928	1.912951	0.617067
16	1	0	0.746303	-4.903407	0.221686
17	8	0	2.247914	0.089478	-0.211749
18	7	0	1.556741	4.786158	-0.246033
19	6	0	0.721978	5.985196	-0.064017
20	1	0	1.115447	6.755791	-0.731690
21	1	0	0.840746	6.353108	0.964830
22	6	0	2.994512	5.033772	-0.437814
23	6	0	-1.880473	-0.697401	0.601562
24	6	0	-2.470395	-0.912885	1.868677
25	6	0	-2.689615	-0.759028	-0.537351
26	6	0	-3.845014	-1.175736	1.945323
27	6	0	-4.056322	-1.043482	-0.454194
28	1	0	-2.260278	-0.585551	-1.517726
29	6	0	-4.635002	-1.243367	0.804282
30	1	0	-4.281927	-1.320311	2.926267
31	6	0	-1.744454	-0.873286	3.176364
32	8	0	-2.280308	-1.076738	4.250301
33	8	0	-0.434697	-0.590179	3.064919
34	1	0	-0.058603	-0.593348	3.962652
35	6	0	-4.830966	-1.080424	-1.747873
36	8	0	-4.431346	-0.469097	-2.743553
37	7	0	-5.973261	-1.808179	-1.746158
38	6	0	-6.786616	-1.946893	-2.945176
39	1	0	-7.762489	-2.344451	-2.664653
40	1	0	-6.916312	-0.970043	-3.414513
41	1	0	-6.323321	-2.620642	-3.675197
42	1	0	-6.164497	-2.424984	-0.970969
43	6	0	-0.743829	5.710224	-0.367358
44	1	0	-1.328847	6.605100	-0.138683
45	1	0	-0.865602	5.499082	-1.435602
46	6	0	3.400999	2.572363	-0.596363
47	6	0	3.643214	3.927934	-1.259207
48	6	0	-1.218435	4.520739	0.464589
49	1	0	4.714600	4.126785	-1.347868
50	1	0	3.221580	3.934776	-2.270323
51	1	0	4.011681	2.486786	0.312273
52	1	0	3.718511	1.762564	-1.258558
53	1	0	-2.252553	4.255074	0.228174
54	1	0	-1.198569	4.794937	1.528420
55	1	0	3.095333	5.998590	-0.939508
56	1	0	3.478337	5.122092	0.544845
57	6	0	4.177963	-1.894191	-0.550563
58	6	0	5.096675	-2.902001	-0.711695
59	1	0	6.137236	-2.685491	-0.927073
60	6	0	3.372402	-4.578372	-0.321250
61	6	0	4.694013	-4.257490	-0.596867

62	1	0	3.067043	-5.617344	-0.234409
63	1	0	4.488764	-0.859878	-0.636157
64	8	0	5.664730	-5.185524	-0.769883
65	1	0	5.296672	-6.076202	-0.670949
66	1	0	-5.699660	-1.423468	0.908295

172aaa_Rh_Julolidin5COOH_naft2-H+carb_B3LYP631dp_PCMw.log

Standard orientation:

Center Number	Atomic Number	Atomic Type	Coordinates (Angstroms) X	Y	Z
1	6	0	-0.288235	3.334253	0.247121
2	6	0	-0.780566	2.069028	0.426072
3	6	0	0.030924	0.907171	0.312372
4	6	0	1.397848	1.125113	-0.008057
5	6	0	1.950862	2.378236	-0.227027
6	6	0	1.105214	3.516089	-0.096014
7	6	0	-0.437576	-0.403926	0.539581
8	6	0	1.804332	-1.212809	0.001596
9	6	0	0.471371	-1.493723	0.321798
10	6	0	0.088585	-2.868129	0.441151
11	1	0	-0.940352	-3.095952	0.693026
12	6	0	0.988966	-3.874893	0.245362
13	6	0	2.353227	-3.593435	-0.083337
14	6	0	2.773541	-2.232293	-0.209058
15	1	0	-1.827263	1.942328	0.680682
16	1	0	0.679773	-4.911106	0.339796
17	8	0	2.245914	0.062276	-0.134489
18	7	0	1.598578	4.768719	-0.304103
19	6	0	0.780313	5.975024	-0.123504
20	1	0	1.178393	6.741097	-0.794702
21	1	0	0.897862	6.350988	0.903661
22	6	0	3.037611	4.990091	-0.490122
23	6	0	-1.906073	-0.670431	0.652614
24	6	0	-2.514511	-0.847398	1.904541
25	6	0	-2.675737	-0.749134	-0.511710
26	6	0	-3.886321	-1.098162	1.971261
27	6	0	-4.047862	-1.021009	-0.443206
28	1	0	-2.225491	-0.603648	-1.487922
29	6	0	-4.652339	-1.187512	0.812000
30	1	0	-4.335313	-1.212413	2.952153
31	6	0	-1.652754	-0.745363	3.156827
32	8	0	-2.201723	-0.887396	4.271036
33	8	0	-0.425055	-0.519057	2.918907
34	6	0	-4.802330	-1.089418	-1.743046
35	8	0	-4.382856	-0.527255	-2.761142
36	7	0	-5.962655	-1.793735	-1.734332
37	6	0	-6.757286	-1.962719	-2.940940
38	1	0	-7.742415	-2.340610	-2.664634
39	1	0	-6.869460	-1.000797	-3.444864
40	1	0	-6.291461	-2.664429	-3.643013
41	1	0	-6.173230	-2.382544	-0.942834
42	6	0	-0.689027	5.711460	-0.425007
43	1	0	-1.265838	6.616868	-0.216308
44	1	0	-0.808984	5.480680	-1.489647
45	6	0	3.410568	2.519268	-0.601122
46	6	0	3.669724	3.858707	-1.290962
47	6	0	-1.179551	4.542641	0.427640
48	1	0	4.743839	4.039462	-1.388700
49	1	0	3.243622	3.851731	-2.300449
50	1	0	4.033762	2.438291	0.299711
51	1	0	3.706976	1.692850	-1.253014
52	1	0	-2.212860	4.279253	0.183208
53	1	0	-1.174637	4.843541	1.484551
54	1	0	3.158670	5.944075	-1.009245
55	1	0	3.526682	5.087404	0.490292
56	6	0	4.126694	-1.952300	-0.534974
57	6	0	5.026617	-2.972394	-0.728964

58	1	0	6.062566	-2.768677	-0.977428
59	6	0	3.294867	-4.624734	-0.288796
60	6	0	4.609616	-4.322252	-0.605722
61	1	0	2.977634	-5.659842	-0.195183
62	1	0	4.448959	-0.922017	-0.627654
63	8	0	5.562884	-5.265781	-0.814160
64	1	0	5.181674	-6.149583	-0.706164
65	1	0	-5.722148	-1.354436	0.892307

172baa_Rh_Julolidin5COOH_naft2-H+carb_B3LYP631dp_PCMw_ring.log

Standard orientation:

Center Number	Atomic Number	Atomic Type	Coordinates (Angstroms) X	Y	Z
1	6	0	-0.738319	3.311297	0.366074
2	6	0	-1.027003	2.005039	0.724180
3	6	0	-0.092686	0.962627	0.627380
4	6	0	1.170051	1.296605	0.133963
5	6	0	1.520077	2.591526	-0.267774
6	6	0	0.557105	3.622265	-0.145276
7	6	0	-0.405147	-0.428741	1.103197
8	6	0	1.894818	-0.962731	0.234400
9	6	0	0.680710	-1.405827	0.726422
10	6	0	0.508803	-2.803239	0.936133
11	1	0	-0.440197	-3.157689	1.325359
12	6	0	1.504792	-3.703248	0.660232
13	6	0	2.760124	-3.260711	0.151021
14	6	0	2.962447	-1.862908	-0.068995
15	1	0	-2.019751	1.785626	1.107969
16	1	0	1.347574	-4.764243	0.828264
17	8	0	2.165569	0.353621	-0.006888
18	7	0	0.857321	4.914111	-0.557046
19	6	0	-0.044492	6.015406	-0.226972
20	1	0	0.184518	6.843661	-0.905254
21	1	0	0.135893	6.380283	0.798803
22	6	0	2.249114	5.279443	-0.804473
23	6	0	-1.807456	-0.900681	0.751805
24	6	0	-2.544560	-1.104817	1.912582
25	6	0	-2.376503	-1.124480	-0.495169
26	6	0	-3.869714	-1.534303	1.877693
27	6	0	-3.702065	-1.577500	-0.552619
28	1	0	-1.831244	-0.959726	-1.417712
29	6	0	-4.443008	-1.770744	0.630538
30	1	0	-4.434991	-1.671361	2.793404
31	6	0	-1.690731	-0.784849	3.077343
32	8	0	-1.959236	-0.830519	4.261017
33	8	0	-0.471436	-0.404083	2.618220
34	6	0	-4.287738	-1.805389	-1.923935
35	8	0	-3.845250	-1.209961	-2.911546
36	7	0	-5.315512	-2.685981	-2.004144
37	6	0	-5.941728	-3.010749	-3.276844
38	1	0	-6.898401	-3.498300	-3.085550
39	1	0	-6.111374	-2.093891	-3.843930
40	1	0	-5.316678	-3.678461	-3.881312
41	1	0	-5.521695	-3.275063	-1.211464
42	6	0	-1.501839	5.590891	-0.376563
43	1	0	-2.156396	6.432231	-0.129135
44	1	0	-1.691903	5.321410	-1.422123
45	6	0	2.905949	2.861370	-0.818861
46	6	0	2.957600	4.181685	-1.590449
47	6	0	-1.782385	4.397398	0.537364
48	1	0	3.995081	4.472742	-1.781262
49	1	0	2.464394	4.069335	-2.562902
50	1	0	3.632871	2.885873	0.004984
51	1	0	3.214775	2.033629	-1.464615
52	1	0	-2.779750	3.986876	0.346356
53	1	0	-1.787494	4.741326	1.581436
54	1	0	2.251245	6.217069	-1.368821

55	1	0	2.781882	5.471324	0.142778
56	6	0	4.212285	-1.420015	-0.576220
57	6	0	5.217182	-2.315810	-0.855361
58	1	0	6.174405	-1.983657	-1.243401
59	6	0	3.809992	-4.164973	-0.145893
60	6	0	5.015339	-3.702949	-0.639775
61	1	0	3.656036	-5.228338	0.019817
62	1	0	4.371055	-0.360994	-0.741363
63	8	0	6.063218	-4.518497	-0.945952
64	1	0	5.820637	-5.438878	-0.768948
65	1	0	-5.482153	-2.077517	0.577247

173aaa_Rh_Julolidin5COOH_naft2-H+_oh_B3LYP631dp_PCMw.log

Standard orientation:

Center Number	Atomic Number	Atomic Type	Coordinates (Angstroms) X	Y	Z
1	6	0	0.506263	3.298114	0.081142
2	6	0	-0.270320	2.177736	0.252704
3	6	0	0.255862	0.864063	0.169195
4	6	0	1.639348	0.758887	-0.122141
5	6	0	2.470873	1.850533	-0.329837
6	6	0	1.907663	3.153341	-0.222198
7	6	0	-0.500263	-0.321427	0.355630
8	6	0	1.517748	-1.624865	-0.052883
9	6	0	0.119043	-1.570366	0.232916
10	6	0	-0.564148	-2.829187	0.397302
11	1	0	-1.625063	-2.814524	0.618592
12	6	0	0.095929	-4.010205	0.279360
13	6	0	1.509713	-4.069900	-0.011267
14	6	0	2.224302	-2.821870	-0.177700
15	1	0	-1.325917	2.303365	0.469717
16	1	0	-0.438939	-4.946982	0.406739
17	8	0	2.224544	-0.473557	-0.213923
18	7	0	2.686114	4.260478	-0.419155
19	6	0	2.165463	5.618061	-0.225649
20	1	0	2.755414	6.287276	-0.858445
21	1	0	2.323095	5.935368	0.816542
22	6	0	4.141144	4.139621	-0.548883
23	6	0	-1.976187	-0.228593	0.595350
24	6	0	-2.582505	-0.045745	1.860252
25	6	0	-2.797885	-0.306233	-0.534394
26	6	0	-3.978638	0.061149	1.941685
27	6	0	-4.190638	-0.222459	-0.443875
28	1	0	-2.353229	-0.431998	-1.515153
29	6	0	-4.779977	-0.026101	0.810669
30	1	0	-4.421357	0.218722	2.918054
31	6	0	-1.855833	0.050400	3.165036
32	8	0	-2.403163	0.317768	4.220206
33	8	0	-0.537041	-0.196552	3.083240
34	1	0	-0.172407	-0.105653	3.981155
35	6	0	-4.974694	-0.313672	-1.728492
36	8	0	-4.456386	-0.027490	-2.812364
37	7	0	-6.264055	-0.715805	-1.619081
38	6	0	-7.114002	-0.873196	-2.789669
39	1	0	-8.153861	-0.929433	-2.465651
40	1	0	-6.987751	-0.013777	-3.450426
41	1	0	-6.867254	-1.780090	-3.353961
42	1	0	-6.587675	-1.099868	-0.744105
43	6	0	0.687662	5.707540	-0.585290
44	1	0	0.328289	6.720580	-0.383151
45	1	0	0.561254	5.519524	-1.657623
46	6	0	3.933984	1.647996	-0.660279
47	6	0	4.520739	2.889376	-1.332985
48	6	0	-0.095824	4.678608	0.228289
49	1	0	5.610240	2.814847	-1.393794
50	1	0	4.139464	2.979030	-2.356461
51	1	0	4.493582	1.423014	0.257968

52	1	0	4.049507	0.774205	-1.307809
53	1	0	-1.148034	4.656566	-0.071564
54	1	0	-0.079795	4.972588	1.287148
55	1	0	4.501772	5.037067	-1.058398
56	1	0	4.606902	4.122508	0.448312
57	6	0	3.629148	-2.877367	-0.461294
58	6	0	4.278578	-4.069541	-0.574347
59	1	0	5.343476	-4.102585	-0.789339
60	6	0	2.183395	-5.273594	-0.130183
61	6	0	3.596363	-5.356074	-0.416568
62	1	0	1.642557	-6.208333	-0.004774
63	1	0	4.171793	-1.945449	-0.583691
64	8	0	4.212020	-6.447114	-0.525256
65	1	0	-5.854106	0.086690	0.912983

174aaa_Rh_Julolidin5COOH_naft2-2H+_oh_B3LYP631dp_PCMw.log

Standard orientation:

Center Number	Atomic Number	Atomic Type	Coordinates (Angstroms) X	Y	Z
1	6	0	0.356743	3.314813	0.153577
2	6	0	-0.364338	2.158814	0.340655
3	6	0	0.217474	0.871231	0.247835
4	6	0	1.596826	0.828905	-0.062760
5	6	0	2.375424	1.959274	-0.286025
6	6	0	1.756484	3.233376	-0.171601
7	6	0	-0.487982	-0.345666	0.462219
8	6	0	1.580202	-1.554566	-0.014200
9	6	0	0.190763	-1.565061	0.290445
10	6	0	-0.434464	-2.854147	0.435862
11	1	0	-1.491322	-2.885145	0.674734
12	6	0	0.271122	-4.005832	0.281597
13	6	0	1.680588	-4.000406	-0.030634
14	6	0	2.337254	-2.720571	-0.178508
15	1	0	-1.419796	2.232226	0.581110
16	1	0	-0.223354	-4.966502	0.396047
17	8	0	2.240112	-0.373519	-0.165939
18	7	0	2.482165	4.377172	-0.388620
19	6	0	1.906669	5.706523	-0.168306
20	1	0	2.456758	6.410323	-0.800042
21	1	0	2.062978	6.019553	0.875994
22	6	0	3.939214	4.318527	-0.522000
23	6	0	-1.974546	-0.323777	0.640509
24	6	0	-2.597593	-0.251922	1.901407
25	6	0	-2.760723	-0.371744	-0.517071
26	6	0	-3.995550	-0.226559	1.958790
27	6	0	-4.158359	-0.363107	-0.449349
28	1	0	-2.292917	-0.418322	-1.494843
29	6	0	-4.774923	-0.281027	0.807675
30	1	0	-4.449193	-0.155937	2.941431
31	6	0	-1.782834	-0.198376	3.202685
32	8	0	-2.435341	-0.132192	4.274469
33	8	0	-0.529364	-0.226847	3.061085
34	6	0	-4.917510	-0.416489	-1.745732
35	8	0	-4.400470	-0.067709	-2.813778
36	7	0	-6.199195	-0.861070	-1.678864
37	6	0	-7.023076	-0.990404	-2.870073
38	1	0	-8.064611	-1.109615	-2.568401
39	1	0	-6.926682	-0.091457	-3.481896
40	1	0	-6.729758	-1.852672	-3.480942
41	1	0	-6.521125	-1.301450	-0.830537
42	6	0	0.420869	5.734121	-0.506658
43	1	0	0.020056	6.729356	-0.293448
44	1	0	0.288431	5.547021	-1.578558
45	6	0	3.840532	1.820456	-0.641392
46	6	0	4.366307	3.088780	-1.314654
47	6	0	-0.306197	4.666808	0.310454
48	1	0	5.457469	3.061609	-1.387039

49	1	0	3.971062	3.165916	-2.333968
50	1	0	4.424282	1.612307	0.265879
51	1	0	3.982093	0.956030	-1.296599
52	1	0	-1.359163	4.597882	0.019738
53	1	0	-0.295154	4.959301	1.369879
54	1	0	4.261495	5.232715	-1.028018
55	1	0	4.411715	4.314123	0.472799
56	6	0	3.735941	-2.710212	-0.487027
57	6	0	4.433631	-3.872204	-0.639151
58	1	0	5.495236	-3.853528	-0.873142
59	6	0	2.404769	-5.173382	-0.190520
60	6	0	3.812122	-5.190392	-0.500605
61	1	0	1.903732	-6.132280	-0.079214
62	1	0	4.237441	-1.753785	-0.597072
63	8	0	4.475913	-6.251785	-0.645745
64	1	0	-5.856294	-0.228689	0.893096

174bba_Rh_Julolidin5COOH_naft2-2H+carb_B3LYP631dp_PCMw_ring.log

Standard orientation:

Center Number	Atomic Number	Atomic Type	Coordinates (Angstroms) X	Y	Z
1	6	0	0.229927	3.369529	0.384849
2	6	0	-0.406374	2.193766	0.747830
3	6	0	0.194517	0.931360	0.631282
4	6	0	1.491721	0.896423	0.113811
5	6	0	2.180911	2.047412	-0.294604
6	6	0	1.549377	3.305565	-0.153120
7	6	0	-0.490389	-0.319415	1.105014
8	6	0	1.565047	-1.480861	0.225910
9	6	0	0.277523	-1.554576	0.740406
10	6	0	-0.263104	-2.852785	0.978567
11	1	0	-1.267154	-2.929814	1.385973
12	6	0	0.446919	-3.992669	0.711389
13	6	0	1.776202	-3.935544	0.175105
14	6	0	2.338171	-2.633070	-0.074039
15	1	0	-1.413132	2.258844	1.152534
16	1	0	0.004779	-4.966681	0.905241
17	8	0	2.180798	-0.280983	-0.043139
18	7	0	2.193208	4.465492	-0.573848
19	6	0	1.650293	5.770383	-0.204865
20	1	0	2.094695	6.513950	-0.874441
21	1	0	1.944078	6.047011	0.822772
22	6	0	3.628794	4.424190	-0.835240
23	6	0	-1.968367	-0.381674	0.752360
24	6	0	-2.743708	-0.358224	1.905594
25	6	0	-2.568434	-0.454249	-0.498414
26	6	0	-4.135949	-0.403032	1.859231
27	6	0	-3.967009	-0.519692	-0.567873
28	1	0	-1.989821	-0.462164	-1.415342
29	6	0	-4.743003	-0.484823	0.608324
30	1	0	-4.724313	-0.366965	2.770077
31	6	0	-1.844335	-0.274769	3.078569
32	8	0	-2.135758	-0.232004	4.259279
33	8	0	-0.567309	-0.251193	2.636628
34	6	0	-4.579689	-0.592386	-1.943765
35	8	0	-3.980339	-0.155155	-2.931556
36	7	0	-5.811381	-1.153976	-2.030604
37	6	0	-6.484842	-1.312249	-3.310631
38	1	0	-7.545612	-1.493689	-3.133171
39	1	0	-6.367494	-0.399447	-3.896771
40	1	0	-6.072906	-2.147633	-3.889015
41	1	0	-6.179765	-1.656287	-1.236904
42	6	0	0.130007	5.781770	-0.328277
43	1	0	-0.253480	6.770973	-0.059627
44	1	0	-0.146030	5.593137	-1.372444
45	6	0	3.576684	1.919764	-0.871486
46	6	0	3.987787	3.178585	-1.638180

47	6	0	-0.463919	4.703942	0.579665
48	1	0	5.062317	3.167128	-1.845607
49	1	0	3.467964	3.219082	-2.602441
50	1	0	4.295025	1.729085	-0.061832
51	1	0	3.627299	1.043711	-1.525398
52	1	0	-1.538893	4.593421	0.399807
53	1	0	-0.360477	5.023842	1.626359
54	1	0	3.891591	5.329017	-1.392199
55	1	0	4.204332	4.445582	0.106499
56	6	0	3.657391	-2.568219	-0.611219
57	6	0	4.369322	-3.707233	-0.881320
58	1	0	5.375405	-3.638296	-1.291521
59	6	0	2.523742	-5.088228	-0.110612
60	6	0	3.847676	-5.052421	-0.647890
61	1	0	2.081590	-6.064313	0.083225
62	1	0	4.096563	-1.593670	-0.802642
63	8	0	4.538790	-6.086336	-0.912003
64	1	0	-5.826161	-0.490475	0.546127

References

1 C. Xu and W. W. Webb, *J. Opt. Soc. Am. B*, 1996, **13**, 481–491.

2 N. S. Makarov, M. Drobizhev and A. Rebane, *Opt. Express*, 2008, **16**, 4029–4047.

3 M. J. Frisch, G. W. Trucks, H. B. Schlegel, G. E. Scuseria, M. A. Robb, J. R. Cheeseman, G. Scalmani, V. Barone, B. Mennucci, G. A. Petersson, H. Nakatsuji, M. Caricato, X. Li, H. P. Hratchian, A. F. Izmaylov, J. Bloino, G. Zheng, J. L. Sonnenberg, M. Hada, M. Ehara, K. Toyota, R. Fukuda, J. Hasegawa, M. Ishida, T. Nakajima, Y. Honda, O. Kitao, H. Nakai, T. Vreven, J. A. Montgomery Jr., J. E. Peralta, F. Ogliaro, M. Bearpark, J. J. Heyd, E. Brothers, K. N. Kudin, V. N. Staroverov, R. Kobayashi, J. Normand, K. Raghavachari, A. Rendell, J. C. Burant, S. S. Iyengar, J. Tomasi, M. Cossi, N. Rega, J. M. Millam, M. Klene, J. E. Knox, J. B. Cross, V. Bakken, C. Adamo, J. Jaramillo, R. Gomperts, R. E. Stratmann, O. Yazyev, A. J. Austin, R. Cammi, C. Pomelli, J. W. Ochterski, R. L. Martin, K. Morokuma, V. G. Zakrzewski, G. A. Voth, P. Salvador, J. J. Dannenberg, S. Dapprich, A. D. Daniels, Ö. Farkas, J. B. Foresman, J. V Ortiz, J. Cioslowski and D. J. Fox, *Gaussian Inc Wallingford CT*, 2016.

4 A. D. Becke, *J. Chem. Phys.*, 1993, **98**, 5648–5652.

5 J. Tomasi, B. Mennucci and R. Cammi, *Chem. Rev.*, 2005, **105**, 2999–3094.

6 G. Grabner, K. Rechthaler, B. Mayer, G. Köhler and K. Rotkiewicz, *J. Phys. Chem. A*, 2000, **104**, 1365–1376.

7 C. Bahou, D. A. Richards, A. Maruani, E. A. Love, F. Javaid, S. Caddick, J. R. Baker and V. Chudasama, *Org. Biomol. Chem.*, 2018, **16**, 1359–1366.
